# Freshwater faces a warmer and saltier future from headwaters to coasts: climate risks, saltwater intrusion, and biogeochemical chain reactions

**DOI:** 10.1007/s10533-025-01219-6

**Published:** 2025-03-10

**Authors:** Sujay S. Kaushal, Sydney A. Shelton, Paul M. Mayer, Bennett Kellmayer, Ryan M. Utz, Jenna E. Reimer, Jenna Baljunas, Shantanu V. Bhide, Ashley Mon, Bianca M. Rodriguez-Cardona, Stanley B. Grant, Tamara A. Newcomer-Johnson, Joseph T. Malin, Ruth R. Shatkay, Daniel C. Collison, Kyriaki Papageorgiou, Jazmin Escobar, Megan A. Rippy, Gene E. Likens, Raymond G. Najjar, Alfonso I. Mejia, Allison Lassiter, Ming Li, Robert J. Chant

**Affiliations:** 1 Department of Geology & Earth System Science Interdisciplinary Center, University of Maryland, College Park, MD USA; 2 Pacific Ecological Systems Division, US Environmental Protection Agency, Office of Research and Development, Center for Public Health and Environmental Assessment, Corvallis, OR USA; 3 Chatham University, Gibsonia, PA USA; 4 Department of Soil & Water Sciences, University of Florida, Gainesville, FL USA; 5 The Charles E. Via Jr Department of Civil and Environmental Engineering, Occoquan Watershed Monitoring Laboratory, Virginia Tech, Manassas, VA USA; 6 Groupe de Recherche Interuniversitaire en Limnologie (GRIL), Université du Québec à Montréal, Montréal, Canada; 7 Watershed and Ecosystem Characterization Division, US Environmental Protection Agency, Office of Research and Development, Center for Environmental Measurement and Modeling, Cincinnati, OH USA; 8 Cary Institute of Ecosystem Studies, Millbrook, NY USA; 9 University of Connecticut, Storrs, CT USA; 10 Department of Meteorology and Atmospheric Science, The Pennsylvania State University, University Park, PA USA; 11 Civil and Environmental Engineering, The Pennsylvania State University, University Park, PA USA; 12 University of Pennsylvania Weitzman School of Design, Philadelphia, PA USA; 13 Horn Point Laboratory, University of Maryland Center for Environmental Science, Cambridge, MD USA; 14 Institute of Marine and Coastal Science, Rutgers, The State University of New Jersey, New Brunswick, NJ USA

**Keywords:** Anthropogenic salt cycle, Global biogeochemical cycles, Carbon cycle, Nitrogen cycle, Metals, Climate change

## Abstract

Alongside global climate change, many freshwater ecosystems are experiencing substantial shifts in the concentrations and compositions of salt ions coming from both land and sea. We synthesize a risk framework for anticipating how climate change and increasing salt pollution coming from both land and saltwater intrusion will trigger chain reactions extending from headwaters to tidal waters. Salt ions trigger ‘chain reactions,’ where chemical products from one biogeochemical reaction influence subsequent reactions and ecosystem responses. Different chain reactions impact drinking water quality, ecosystems, infrastructure, and energy and food production. Risk factors for chain reactions include shifts in salinity sources due to global climate change and amplification of salinity pulses due to the interaction of precipitation variability and human activities. Depending on climate and other factors, salt retention can range from 2 to 90% across watersheds globally. Salt retained in ecosystems interacts with many global biogeochemical cycles along flowpaths and contributes to ‘fast’ and ‘slow’ chain reactions associated with temporary acidification and long-term alkalinization of freshwaters, impacts on nutrient cycling, CO_2_, CH_4_, N_2_O, and greenhouse gases, corrosion, fouling, and scaling of infrastructure, deoxygenation, and contaminant mobilization along the freshwater-marine continuum. Salt also impacts the carbon cycle and the quantity and quality of organic matter transported from headwaters to coasts. We identify the double impact of salt pollution from land and saltwater intrusion on a wide range of ecosystem services. Our salinization risk framework is based on analyses of: (1) increasing temporal trends in salinization of tributaries and tidal freshwaters of the Chesapeake Bay and freshening of the Chesapeake Bay mainstem over 40 years due to changes in streamflow, sea level rise, and watershed salt pollution; (2) increasing long-term trends in concentrations and loads of major ions in rivers along the Eastern U.S. and increased riverine exports of major ions to coastal waters sometimes over 100-fold greater than forest reference conditions; (3) varying salt ion concentration-discharge relationships at U.S. Geological Survey (USGS) sites across the U.S.; (4) empirical relationships between specific conductance and Na^+^, Cl^−^, SO_4_^2−^, Ca^2+^, Mg^2+^, K^+^, and N at USGS sites across the U.S.; (5) changes in relationships between concentrations of dissolved organic carbon (DOC) and different salt ions at USGS sites across the U.S.; and (6) original salinization experiments demonstrating changes in organic matter composition, mobilization of nutrients and metals, acidification and alkalinization, changes in oxidation–reduction potentials, and deoxygenation in non-tidal and tidal waters. The interaction of human activities and climate change is altering sources, transport, storage, and reactivity of salt ions and chain reactions along the entire freshwater-marine continuum. Our salinization risk framework helps anticipate, prevent, and manage the growing double impact of salt ions from both land and sea on drinking water, human health, ecosystems, aquatic life, infrastructure, agriculture, and energy production.

**Supplementary Information:**

The online version contains supplementary material available at 10.1007/s10533-025-01219-6.

## Introduction

Freshwater salinization is increasing in many regions of the world and the anthropogenic salt cycle is now a driver of global change across diverse Earth systems (Williams 1999; Kaushal et al. 2005, 2023a; Cañedo-Argüelles et al. 2013). The world’s freshwaters face a salty future due to: increasing land-use change (Williams 1999), road salt use (Kaushal et al. 2005; Dugan et al. 2017; Hintz and Relyea 2019), wastewater (Bhide et al. 2021; Grant et al. 2022), resource extraction (Kaushal et al. 2021, 2023a, b, c), groundwater pumping (Kaushal et al. 2024), irrigation (Cañedo-Argüelles et al. 2013; Thorslund et al. 2021), climate change and sea level rise (Herbert et al. 2015), human-accelerated weathering (Kaushal et al. 2021, 2023b), resource extraction (Kaushal et al. 2024), mineral fertilizers containing chloride and sulfate in agricultural areas (Kaushal et al. 2024), and other factors (Cunillera-Montcusí et al. 2022). Concentrations and mixtures of salt ions and alkalinity in freshwaters have been altered across regional and global scales (Raymond et al. 2008; Kaushal et al. 2013, 2017, 2018a, b, 2019, 2023a, b). Increased freshwater salinization and alkalinization is occurring simultaneously with rising temperatures in streams, rivers, and estuaries (Kaushal et al. 2010; Van Vliet et al. 2011, 2023; Tassone et al. 2022; Hinson et al. 2022). The convergence of salt pollution from land and saltwater intrusion places both non-tidal and tidal freshwaters ecosystems at risk for declines and shifts in ecosystem services and functions sensitive to salinity thresholds (Herbert et al. 2015; Tully et al. 2019a, b; Lassiter 2021, 2024; Little et al. 2022; Bernhardt 2022; Valle-Levinson and Li 2023; O’Donnell et al. 2024). Here, we synthesize a framework for anticipating how climate change, rising salt pollution, and saltwater intrusion can alter salinity sources, transport, retention, and reactivity from headwaters to tidal waters, with implications for drinking water, aquatic life, agriculture, and infrastructure.

Freshwater Salinization Syndrome (FSS) refers to the interrelated suite of physical, chemical, and biological impacts of salt ions that degrade the environment, impact infrastructure, and disrupt ecosystem services (e.g., Kaushal et al. 2018a, b, 2019, 2020, 2021, 2022, 2023b, 2024). Salinization, acidification, alkalinization, corrosivity, and water hardness can be chemical indicators of FSS depending on environmental factors. Briefly, salinization refers to an increase in salt ions such as sodium (Na^+^), potassium (K^+^), calcium (Ca^2+^), magnesium (Mg^2+^), and chloride (Cl^−^), bicarbonate (HCO_3_^−^), carbonate (CO_3_^2−^) and sulfate (SO_4_^2−^) in soils, waters, and air (Kaushal et al. 2017, 2023b, 2024). Corrosivity is related to an increase in the chloride to sulfate mass ratio (Edwards and Triantafyllidou 2007; Stets et al. 2018), alkalinity (Edwards et al. 1996), and dissolved oxygen, temperature, and pH; neutral waters are not particularly corrosive but acidic (pH < 6.5) and alkaline (pH > 7.5) waters can be corrosive when alkalinity is low. As an important secondary effect of freshwater salinization, many biogeochemical processes associated with FSS can also mobilize diverse chemical cocktails, which refer to distinct elemental mixtures with shared sources or pathways of transport or transformation in the environment (Kaushal et al. 2018a, b, 2019, 2020, 2021, 2024; Shelton et al. 2024). The chemical cocktails of FSS can be linked to ‘chain reactions,’ where chemical products from one biogeochemical reaction influence subsequent reactions, chemical mixtures, and ecosystem responses in the environment. More work is needed to predict how climate change and variability will alter the spread and severity of diverse FSS impacts, chemical cocktails, and chain reactions across natural, engineered, and socio-ecological systems.

## Why do we need to anticipate the double impact of salinization from land and sea?

**Fig. 1:**
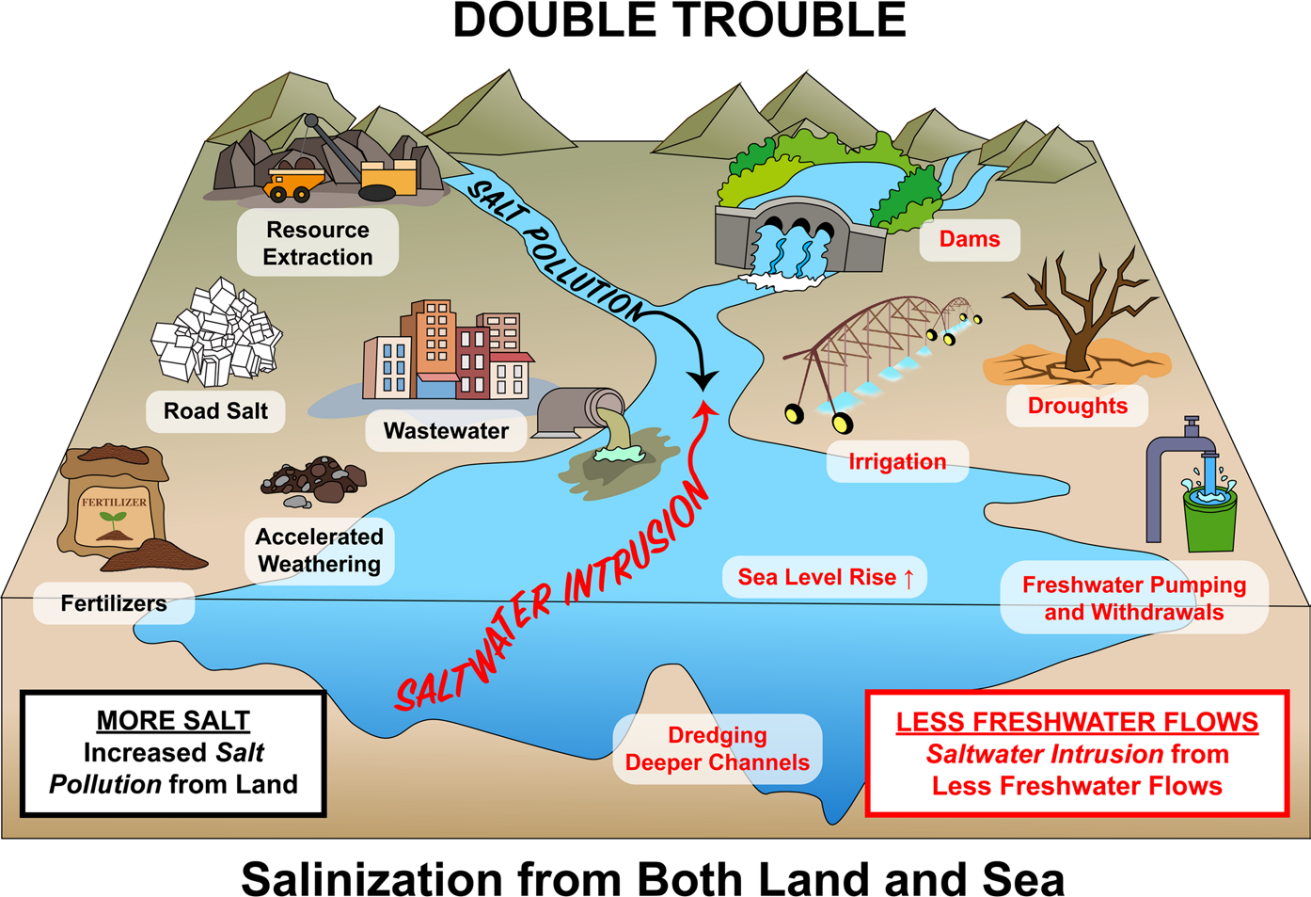
Salinization exerts a growing double impact on freshwaters from both land and sea. Salt pollution from land is increasing concentration of multiple salt ions in rivers worldwide (Kaushal et al. 2019, 2023a, b, 2024). At the same, decreased freshwater flows, droughts, and drying rivers can also increase saltwater intrusion risks. Variability in the location of the salt front of rivers and estuaries likely depends on: tides, winds, waves and storm surges, increased precipitation, bathymetry, dredging deeper channels, sea level rise, degree of mixing, upriver freshwater withdrawals for agriculture, power, and water consumption, and other complex factors (Najjar et al. 2010; Ralston and Geyer 2019; Tian 2019; Lassiter 2021; Valle-Levinson and Li 2023). Tidal freshwaters and low salinity zones along streams, rivers, estuaries, and wetlands are the most at risk from the double impact of salt from land and sea. Graphics modified from IAN Symbol Library and Canva

There have been advancements in our knowledge of the causes and consequences of freshwater salinization, but there are major knowledge gaps regarding the double impact of salinization from land and sea on freshwaters that we address in this paper. One emerging question is related to the complex effects of climate change: how will the interaction between climate change and human activities alter the sources, fluxes, storage, and flowpaths of different salt ions from headwaters to coastal waters? Although less explored, the effects of climate change can have opposing or synergistic forces on salinity along rivers. Increases in rain and floods can reduce saltwater intrusion whereas droughts can enhance saltwater intrusion into tidal freshwater zones (Fig. 1). Warming temperatures can reduce salinity in streams and rivers affected by road salt pollution during winter seasons, but they can also increase salinity due to evaporative concentration during summer seasons. The balance of opposing, reinforcing, and interactive forces of climate change on salinity across space and time along the freshwater-marine continuum is not well known. There may be cases where freshwater in rivers is becoming saltier due to increased watershed salt pollution and saltwater intrusion into tidal freshwater zones (Fig. 1), but saltwater ecosystems further downriver along estuaries and coastal waters are becoming fresher due to increased floods and dilution. The complex effects of climate change and the role of river discharge on influencing salinity risks represents an emerging knowledge gap for many world regions (Fig. 1).

**Fig. 2:**
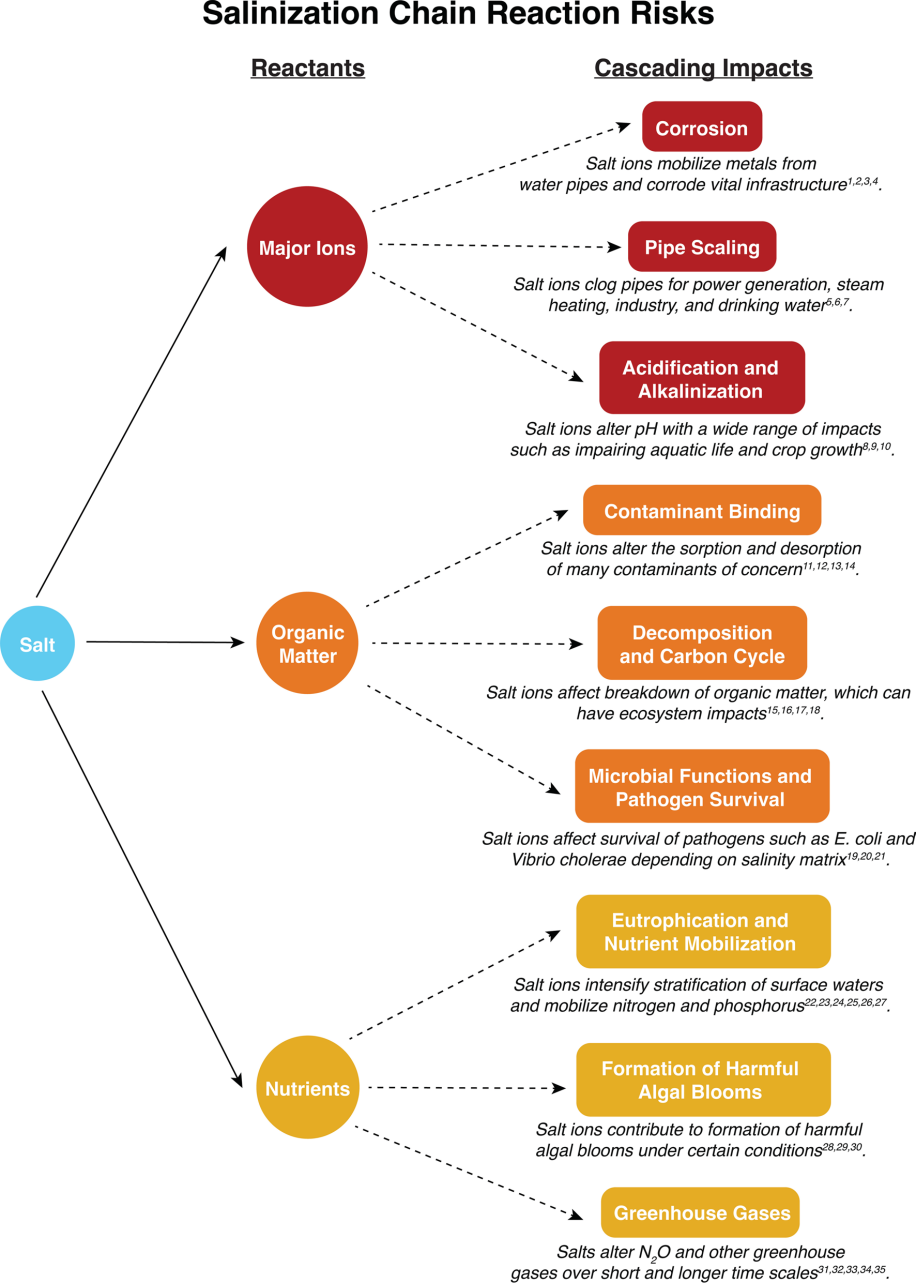
Examples of different chain reactions triggered by salt as part of Freshwater Salinization Syndrome, which can influence the role of major ions, organic matter, and nutrients in degrading ecosystem services and causing water quality issues. Superscripts in the figure correspond to literature references for specific types of chain reactions that are provided in this caption. For example, impacts on corrosion can be found in Pieper et al. (2018)^1^; Stets et al. (2018)^2^; Zhou et al. (2021)^3^; Kaushal (2016)^4^. Impacts on pipe scaling can be found in Li et al. (2022a, b)^5^; MacAdam and Jarvis (2015)^6^; Cao et al. (2022)^7^. Impacts on acidification and alkalinization can be found in Bui (2017)^8^; Zalizniak et al. (2009)^9^; Zhao et al. (2022)^10^. Impacts on contaminant binding can be found in Navarro et al. (2022)^11^; Rodríguez-Liébana et al. (2010)^12^; Yin et al. (2022)^13^; Acosta et al. (2011)^14^. Impacts on decomposition and carbon cycling can be found in Oliveira et al. (2021)^15^; Connolly et al. (2014)^16^; Weston et al. (2011)^17^; Almeida Júnior et al. (2020)^18^. Impacts on microbial functions and pathogen survival can be found in Huq et al. (1984)^19^; DeVilbiss et al. (2021)^20^; Van Gray and Ayayee (2024)^21^. Impacts on eutrophication and nutrient mobilization can be found in Lind et al. 2018^22^; Radosavljevic et al. (2022); Salcedo et al. (2024)^24^; Galella et al. (2023a, b)^25^; MacLeod et al. (2011)^26^; Steinmuller and Chambers (2018)^27^. Impacts on algal blooms can be found in Osburn et al. (2023)^28^; Yu et al. (2022)^29^; Duval et al. (2018)^30^. Impacts on greenhouse gases can be found in Ardón et al. (2018)^31^; Neubauer et al. (2013)^32^; Dang et al. (2019)^33^; Xie et al. (2020)^34^; Weston et al. (2014)^35^

We do not completely understand whether there are similar effects of salinization from both land and sea on chemical, biological, and physical processes. For example, why does salinization lead to acidification or alkalinization in certain ecosystems, and is there a difference in responses across time scales and along non-tidal and tidal waters? What are the site specific conditions influencing the trajectory of acid–base status in response to salinization along the freshwater-marine continuum? In addition, what are the effects of salinization on the carbon cycle? How does salinization affect dissolved inorganic carbon (DIC) and river alkalinization? How can salinization alter the absorption or efflux of atmospheric carbon dioxide (CO_2_) along rivers? Why does salinization sometimes cause an increase in dissolved organic carbon (DOC) concentrations and quality (increases in protein-like and reactive fractions) but cause decreases in DOC concentrations and quality (increases in more recalcitrant humic fractions) in other cases? In this paper, we define connections between salinization and changes in acids and bases, metals, and carbon and nutrient cycles, and we also synthesize salinization’s direct, indirect, and interactive effects on many biogeochemical cycles. We propose the new concept of salinization ‘chain reactions’ extending from elemental interactions to sequences of alterations in organisms, ecosystems, infrastructure, and Earth’s biogeochemical cycles (Fig. 2).

Until now, salinization of inland waters and saltwater intrusion impacts have been typically studied and managed separately due to disciplinary divisions and boundaries among hydrology, stream ecology, soil science, limnology, estuarine science, engineering, planning, and oceanography. Here, we investigate how salinization exerts a growing double impact on freshwaters from both land and sea due to increased salt pollution, decreased freshwater flows along river systems, and saltwater intrusion (Fig. 1). The causes and consequences of salinization have not been compared from headwaters to coastal waters, even within the same geographic regions of the world. This lack of connection in our understanding of salinization between inland and coastal waters opens up a new research frontier and question: How will the combination of freshwater salinization from land and saltwater intrusion from the ocean impact ecosystems, infrastructure, water, energy, and food production, and global biogeochemical cycles from headwaters to coasts? We make new connections among salt and different biogeochemical chain reactions and their emerging risks. We discuss the double impact of salt from both land and sea on multiple elemental cycles together, which is not always considered. We demonstrate that there is an added value of putting all of these elements together to anticipate a more holistic and comprehensive sequence of cascading impacts on water quality and ecosystem services from headwaters to coasts.

## A salinization risk framework from headwaters to coasts in a changing climate

Here, we synthesize and conceptualize a salinization risk assessment framework from headwaters to coasts. Risks can be defined in many different ways. We consider salinity risks as the intersection of: (1) hazards, (2) probability, (3) salt exposure history, and (4) vulnerability (e.g., a community is at risk if they are exposed to a hazard or are more vulnerable to that hazard). These types of conceptual frameworks have been widely used for analyzing risks and vulnerability associated with climate change and flooding (Brooks 2003). We explore how the frequency and magnitude of salinization events has been shifting spatially and temporally from headwaters to estuaries. We explore three major questions for guiding future predictions, mitigation, and management: (1) how will salt sources, transport, and storage change in watersheds in response to climate change and variability?, (2) how will salinization trigger chain reactions and cascading impacts from headwaters to tidal waters?, and (3) and how will saltwater intrusion due to sea level rise shift ecosystem functions and services? We illustrate how salinization risks can be associated with ‘fast’ processes, which occur over shorter timescales from hours to days and ‘slow’ processes, which occur over longer time periods from years to decades (e.g., Michael et al. 2005, 2017; Kirwan and Gedan 2019; Tully et al. 2019a, b). Anticipating changes in salinity sources, salt retention, biogeochemical chain reactions, and saltwater intrusion from headwaters to coastal zones will improve FSS monitoring, modeling, and management strategies globally (Figs. 1 and 2).

In our salinization risk framework, we synthesize 10 interactive risks based on data from: (1) original field and lab experiments, (2) water quality monitoring across space and time, and (3) case studies from the global literature. More details on all data sources are available in Supporting Information. Each risk that we identify in this paper also represents an emerging frontier of salinization research for further exploration. Although we highlight examples from the intensively monitored Chesapeake Bay region and regions throughout the U.S., categories of predictions may be applicable to other regions globally. We predict that climate change and variability will increase salinization, which is already occurring along both ends of the freshwater-marine continuum (e.g., Herbert et al. 2015; Kaushal et al. 2018a, 2022, 2023b, c; Little et al. 2022; Maas et al. 2023; Shelton et al. 2024). In addition, we show that salinization triggers chain reactions along the freshwater-marine continuum with implications for different ecosystem functions and ecosystem services (Fig. 2). We propose the need for better identification and anticipation of diverse salinity risks and collaborative partnerships in regions where salinity monitoring is difficult. Overall, we synthesize a set of interactive risks regarding how climate change, pollution, and saltwater intrusion will cause cascading effects affecting water quality, infrastructure, and ecosystems extending from headwaters to tidal waters.

### Part 1: anticipating changes in watershed salt sources, transport, and storage

*Interactive risk 1: Watershed sources and transport of salt will shift with global warming and droughts.*

Climate change alters many direct and indirect salinization risks. More work is needed to fully predict the effects of global warming and intensification of the hydrological cycle on freshwater salinization, but some large-scale impacts have emerged such as changing regional ocean salinity; some areas of Earth’s oceans are becoming saltier and other areas are becoming fresher due to regional changes in rainfall and precipitation patterns and increasing glacial meltwater (Durack et al. 2012). Analysis of long-term changes in ocean salinity imply that there could be a 16–24% amplification of the global water cycle and evaporation and precipitation in a 2–3 C° warmer world (Durack et al. 2012). Some semi-arid and arid regions are expected to become drier, and some humid regions may become wetter (Zaitchik et al. 2023), which could amplify or counteract the effects of salinization based on changes in sources, storage, and transport (Lintern et al. 2021, 2023). The effects of warming and droughts on salinization has been a growing topic of concern primarily in dry environments (Jeppesen et al. 2020; Lintern et al. 2023). Droughts can increase freshwater salinization through evaporative concentration of salt ions (Kaushal et al. 2023b), losses in plant cover causing losses in regulation of the hydrologic cycle (Perri et al. 2020), decreases in dilution capacity (Lintern et al. 2023), changes in human water uses, increased irrigation impacts on salinization during drier conditions (Thorslund et al. 2021), and complex feedbacks leading to aridity or desertification (D’Odorico et al. 2013; Perri et al. 2020).

In response to global warming and droughts, there are also other important human feedbacks on salinity in watersheds. For example, per capita water consumption can increase with temperature, particularly due to increased use of water for irrigation for home lawns, swimming pools, and luxury uses by affluent communities (Balling et al. 2008); these water uses represent 60–75% of residential water use in some regions (Balling et al. 2008). There can be increased seasonal variability of wastewater discharges in response to climate change (Khalkhali and Mo 2020), and wastewater has been shown to be a major source of salinity in watersheds (Bhide et al. 2021; Grant et al. 2022). Socioecological feedbacks can increase salinization of source waters in at least two ways: (1) facilitating the evapotranspiration of irrigated soils (based on water withdrawals) in green space in urban settings (thereby offsetting salt dilution) (Qiu et al. 2017); and (2) increasing the rate at which water passes through homes, where salty chemicals (e.g., clothing detergents and household products) are added to waste streams and subsequently discharged back to freshwaters (Rippy et al. 2024). Understanding the feedback between salinity and changes in human water consumption and management in response to climate change presents a challenge (Fig. 1).

In humid climates, we anticipate that warming temperatures and changes in precipitation will also interact with underlying geology to increase major ion concentrations and/or fluxes through human-accelerated weathering (Kaushal et al. 2013, 2017; Raymond 2017; Kopáček et al. 2017a, b; Crawford et al. 2019). Under future climate scenarios, river water temperatures are predicted to increase on average by approximately 0.8–1.6 °C for 2071–2100 relative to 1971–2000 (Van Vliet et al. 2013). Warming temperatures influence physical properties of water including mineral solubility and dissolution rates (Kaushal et al. 2010; Raymond 2017; Li et al. 2024a). Increased salinity also modestly reduces the specific heat capacity of water, which allows water to increase in temperature more quickly and cool off more slowly (Millero et al. 1973; Sharqawy et al. 2010). Human-accelerated weathering increases concentrations of alkalinity, SO_4_^2−^, HCO_3_^−^ and CO_3_^2−^, Ca^2+^, and other ions, which all can contribute to rising salinization and alkalinization trends (Kaushal et al. 2013, 2017, 2023a, b, c, 2024).

**Fig. 3:**
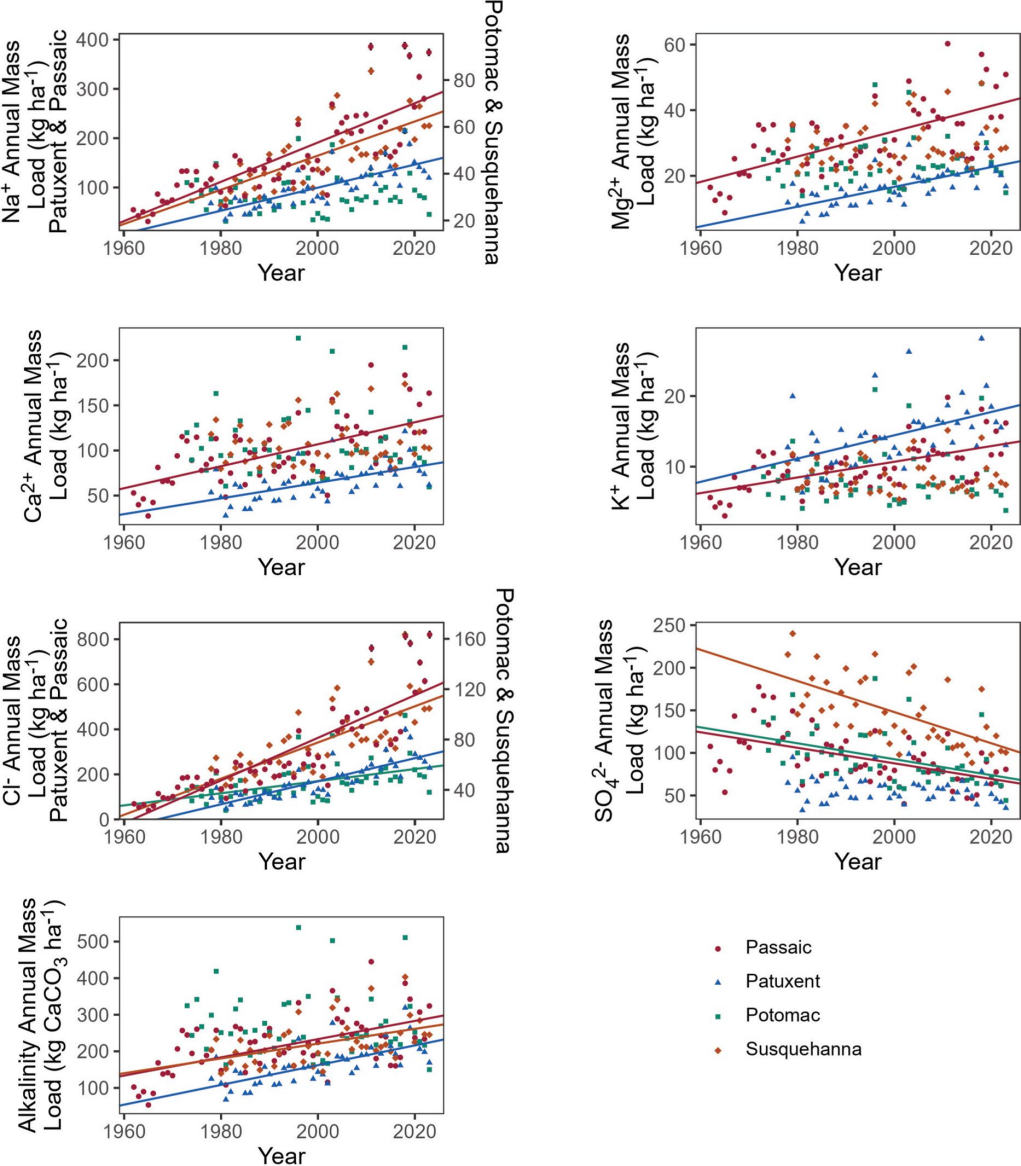
Increasing long-term annual exports of major ions in kg/ha/yr (mass transport) in Susquehanna, Potomac, Patuxent, and Passaic Rivers along the eastern U.S. Annual exports of sodium, chloride, potassium, and alkalinity show increasing patterns over decades whereas sulfate shows a decreasing pattern over decades. There have been increased pulses in annual exports of major ions due to increasing climate variability over recent decades. Information on methods for salt ion load estimates can be found in the Supporting Information

Human activities and climate change are accelerating geological processes, which are influencing the concentrations and compositions of major ions in streams and rivers, in addition to changes in pollution sources. Annual watershed fluxes of major ions (Na^+^, Cl^−^, Ca^2+^, Mg^2+^, K^+^, and alkalinity) have been significantly increasing in some major tributaries of the Chesapeake Bay and other rivers draining the U.S. East Coast over previous decades (Fig. 3) due to human-accelerated weathering, increased ion exchange from salt pollution, and changes in atmospheric acid deposition (Kaushal et al. 2013, 2017, 2018a, b). Annual riverine fluxes of major ions per unit watershed area in Fig. 3 are sometimes over 100 times greater than small forest reference watersheds (Likens et al. 1967; Watmough and Dillon 2004), but within the range of other human-impacted watersheds and rivers across the U.S. (Barco et al. 2013). Annual riverine fluxes of SO_4_^2−^ show a significant decrease in some watersheds over recent decades because of acid rain regulations and decreased SO_2_ emission from coal fired power plants (Fig. 3). In Europe, the SO_4_^2−^ content has also decreased significantly in agricultural fertilizers, due to the reduction in the use of (NH_4_)_2_SO_4_, which acidifies soils, and the reduction in the use of H_2_SO_4_ (and the increasing use of H_3_PO_4_) in the production of P fertilizers (Kopáček et al. 2014a, b).

Weathering of geologic materials is accelerated by rising temperatures in watersheds and rivers (Kaushal et al. 2010, 2023b; Raymond 2017), decreased ice cover and exposure of rocks to weathering agents (Kaushal et al. 2013, 2017; Drake et al. 2018; Crawford et al. 2019), and changes in precipitation, temperature, and freeze thaw cycles (Kopáček et al. 2017a). Easily weathered construction materials and agricultural lime are affected disproportionately thereby contributing to enhanced weathering fluxes from human-dominated watersheds (Barnes and Raymond 2009; Kaushal et al. 2017; Moore et al. 2017). In some regions, urban watersheds can export almost 800% more dissolved inorganic carbon (DIC) than forested watersheds and 200% more DIC than agricultural watersheds (Barnes and Raymond 2009). In the future, freshwater salinization could increase due to climate and land use change, accelerated physical and chemical weathering, and increased mineral solubility.

**Fig. 4:**
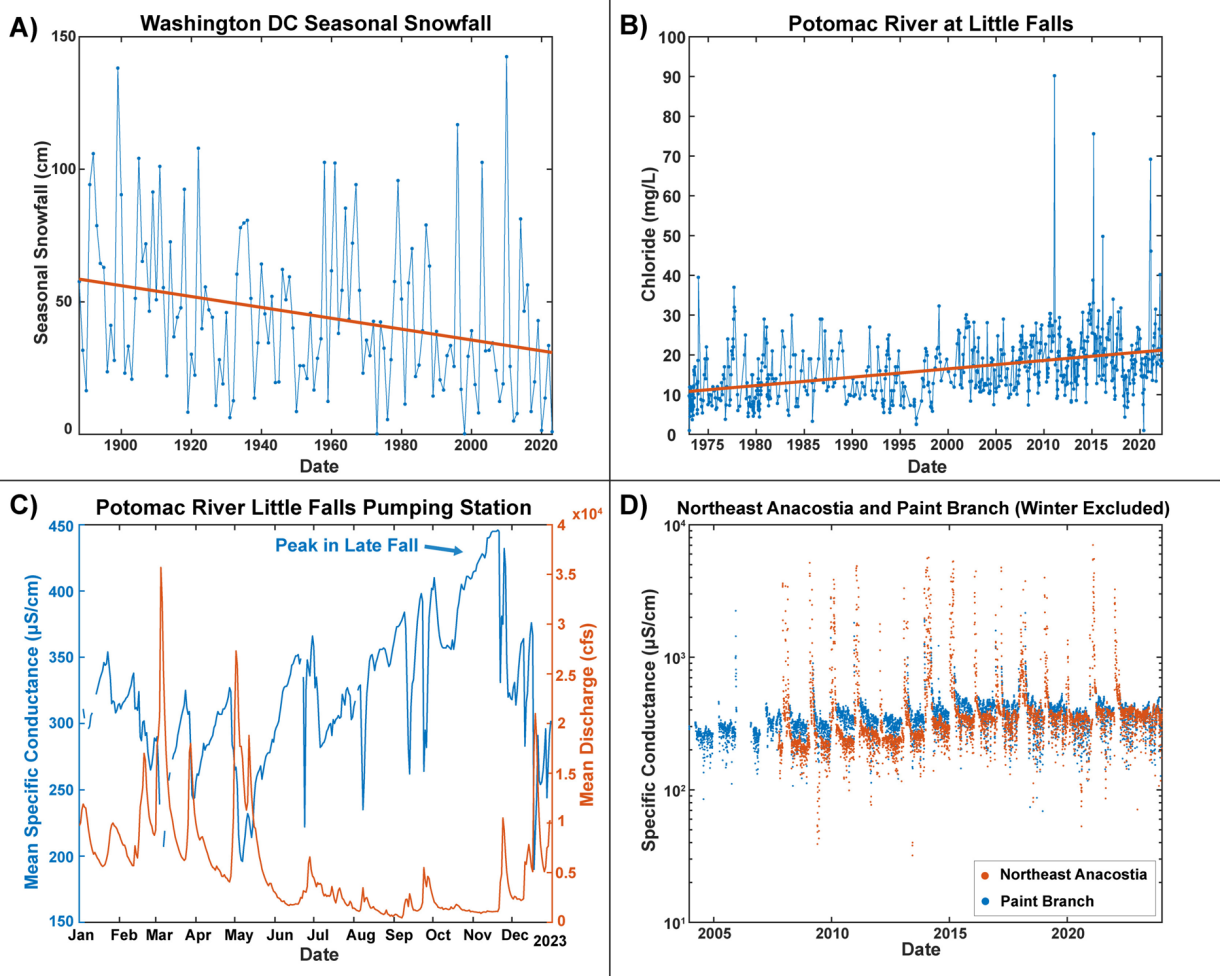
(Panel A) Decreasing snowfall trend in Washington D.C. over 100 years from National Oceanic and Atmospheric Administration (NOAA) data. (Panel B) Increasing long-term trends in chloride concentrations with extreme pulses during recent years in the Potomac River at Little Falls Pumping Station near Washington D.C. from U.S. Geological Survey (USGS) data. (Panel C) Increasing specific conductance during drought conditions near the drinking water intake for the Potomac River at the Little Falls Pumping Station revealed by USGS high-frequency sensor data during 2023. (Panel D) Increasing baseflow concentrations of specific conductance with winters removed in the Northeast Branch of the Anacostia River in the Potomac Watershed

In wetter and colder climates, warming temperatures may decrease the perceived need for road salt use in the future (Fig. 4A), but urbanization is also simultaneously increasing thereby leading to more roadways that will require salting (Kaushal et al. 2005; Rossi et al. 2022). Despite warming temperatures, annual fluxes of Na^+^ and Cl^−^ ions have significantly increased over the past 40 years in major tributaries of the Chesapeake Bay and other regions in the northeastern U.S. (Fig. 3). In some cases, warmer temperatures have been shown to decrease salinization from road salt (Arvidsson et al. 2012; Stirpe et al. 2017; Kaushal et al. 2022), but there have been only relatively short-term annual salinity reductions or substantial lags in trends due to variations in hydrogeology and retention of salt ions in soils and groundwater (Novotny and Stefan 2010). If road salt applications were discontinued, it is projected that it would still take some surface waters 10–30 years before chloride concentrations would return to natural levels (Novotny and Stefan 2010). It is likely that long-term increases in urban impervious surface cover (increasing surface area of roads and parking lots requiring deicers) can overwhelm impacts of warming winters to sustain long-term salinization trends in some regions.

Salinization is still increasing in regions experiencing a decrease in snow. In the Washington D.C. region, there has been decreased snowfall over the past century (Fig. 4A) (Kocin and Uccellini 2016). From 1965 to 2005, mean, minimum, and maximum temperatures were increasing at rates ranging from 0.42 to 0.46 °C per decade in the northeastern U.S., and the fastest rates of warming were during the winter months (Burakowski et al. 2008). Yet, there have still been more intense snow events over shorter time periods during recent decades leading to increased salinity peaks in many streams and rivers including the Potomac River (Fig. 4B) (Kocin and Uccellini 2016). Interestingly, there has also been increasing salinization during drought years with minimal or no snow due to decreased dilution from runoff (Fig. 4C). Overall, there has been increased salinization of freshwaters with increased variability of salinity pulses from winter snowstorms in the northeastern U.S., including increasing baseline specific conductance during non-winter months due to the steady accumulation of road salt in soils and ground water (Kaushal et al. 2005) (Fig. 4D). In addition, an increase in rain on snow events may also enhance transport of salt pollution to freshwaters. Approximately 53% of the contiguous U.S. is impacted by rain on snow events with the highest frequency in the northeastern U.S. and western mountains with greater than 3 rain on snow events per year (Seybold et al. 2022). If road salt is applied during snow events followed shortly thereafter by heavy rains (or snowmelt), it could change pathways by which salt enters streams and rivers. Thus, there can be both synergistic and reinforcing or opposing and counteracting forces that determine the net rates of salinization in the future that should be considered holistically.

*Interactive risk 2: More intense precipitation patterns will amplify watershed salinity pulses.*

Salinity could become increasingly pulsed in some regions due to changes in precipitation and these pulses could be amplified by the interaction between climate variability and human activities (Daley et al. 2009; Kaushal et al. 2014). Based on long-term data analyses, the frequency of both extreme high and low-flow discharge events in streams and rivers has increased by approximately 100% from historical conditions across many regions of North America (Dethier et al. 2020). Pulses are large increases in concentrations or fluxes over relatively short periods of time (Kaushal et al. 2008, 2014). In some regions, rainfall could decrease but intensity of rainfall could increase over shorter time scales such as extreme storm events (Asadieh and Krakauer 2017; Naz et al. 2018). In coastal waters, high water levels associated with extreme storms and king tides (i.e., the highest high tide of the year) may serve as a predictor for future freshwater saltwater intrusion events and help us know what the “new normal” will look like for coastal areas impacted by sea level rise and land subsidence. In addition to storms, droughts can also lead to increases in specific conductance and salinity and vulnerability to saltwater intrusion events (e.g., Fig. 4C).

Streamflow varies regionally and profoundly affects salinity risks, and contributions of low streamflow to river discharge is increasing in some regions whereas low flow is decreasing in other regions (Rice and Hirsch 2012; Rice et al. 2017). For example, some rivers in the northeastern U.S. have experienced increasing trends in streamflow due to increased precipitation (Zhang et al. 2010a; Rice et al. 2017). During periods of decreased streamflow, low flow in tidal rivers can induce the upstream encroachment of salt fronts (Tian 2019); for example, there was an increase in specific conductance in the Potomac River upstream of its estuary, as streamflow decreased during a regional drought (Fig. 4C). Thus, in addition to salinity pulses from land to coasts based on road salt applications (Bubeck et al. 1971), there may be salinity pulses from the coast towards the land based on droughts and saltwater intrusion (Li et al. 2024b). Changes in runoff would not only influence dilution capacity and attenuation of salty inputs but also affect the sources, timing, fluxes, and flowpaths of salt ions transported in watersheds.

There could also be more extreme winter salinity pulses from road salt application due to shifts in the frequency and magnitude of both snow events and rain on snow events in colder regions. In the eastern U.S., there was an increase in extreme snowfall events in the 1950’s and 1990’s with concomitant pulses in chloride concentrations in the Potomac River during the corresponding period of measurement in the 1990’s (Fig. 4B) (Kocin and Uccellini 2016). El Niño and La Niña events can also influence snowfall in the Mid-Atlantic U.S. and elsewhere. With increasing climate change and global warming, there is increased water vapor in the atmosphere contributing to climate variability. For example, the dew point has been increasing over time in many regions of the U.S. (Wu and Wang 2021). When air can hold more moisture, it rises and cools due to adiabatic cooling, and then drops more precipitation as either rain, snow, or sleet depending on atmospheric temperatures. Changes in the timing, duration, and magnitude of precipitation events affect flushing and dilution of salts from watersheds, which could alter pulses in concentrations and fluxes of different salt ions and associated chemical cocktails.

Land use change can further interact with winter precipitation variability to amplify salinity pulses (Kaushal et al. 2014). Impervious surface cover is strongly related to salinity in urban streams (Kaushal et al. 2005; Baker et al. 2019) and impervious surfaces can efficiently convey roadway chemicals to waterways during precipitation events. In some cases, freshwater ecosystems may become more adapted to lower salinity levels during warmer years with minimal snow and road salt applications and then are exposed to extreme salinity events (sensu DeLaune et al. 2021). More extremes in salinity and temperature could influence water quality and cause stress in organisms and/or their ability for osmoregulation and adaptation to a more variable environment (Van Meter et al. 2011; Duan and Kaushal 2013, 2015; Walker et al. 2020; Garcia et al. 2024). It may become harder for cities and municipalities to manage, plan, and budget for extreme snow events leading to pulses in road salt application rates across years (Matthews et al. 2017). We anticipate that variability in road salt application rates across dry and snowy winters could have lingering biogeochemical consequences and impact water quality spanning over multiple years (Novotny and Stefan 2010) (Figs. 3 and 4).

*Interactive risk 3: Salt retention within watersheds will increase on regional, continental, and global scales.*

**Fig. 5:**
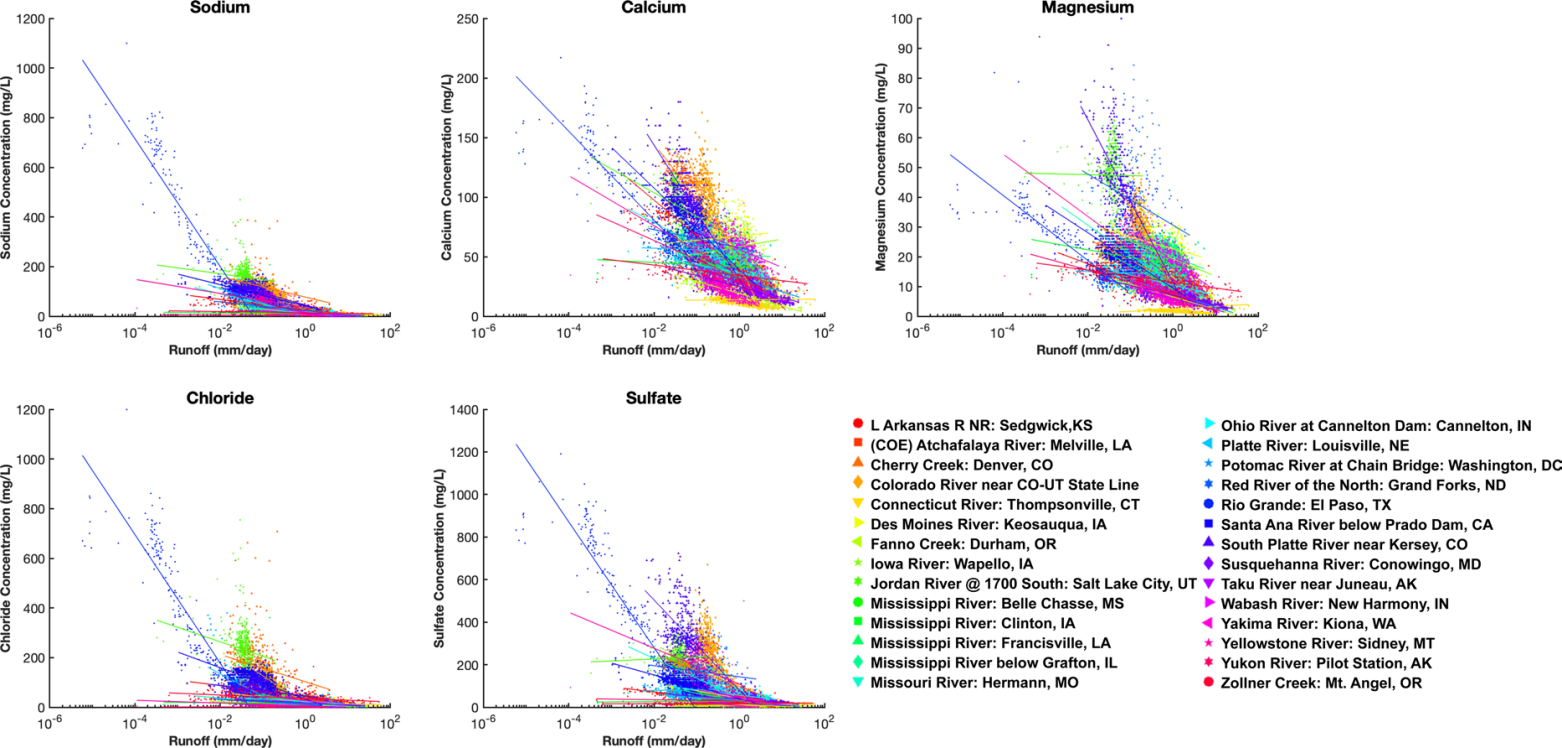
Relationships between streamflow and concentrations of sodium, calcium, magnesium, chloride, sulfate in streams and rivers across the U.S. monitored by the U.S. Geological Survey (USGS). Concentrations of major ions generally decrease with increases in runoff, but there appears to be a stabilization of concentrations (plateau) for many major ions at the highest levels of runoff. Information on USGS stream and river sites can be found in the Supporting Information

The limited capacity of watersheds to flush out salts during precipitation events can lead to long-term storage and rising salinity trends in streams, rivers, and lakes (Kaushal et al. 2005; Kelly et al. 2008; Lintern et al. 2023; Van Meter and Ceisel 2024). Across the U.S., there are varying relationships between streamflow and concentrations of Na^+^, Ca^2+^, Mg^2+^, Cl^−^, and SO_4_^2−^in streams and rivers (Fig. 5). Concentrations of Na^+^, Cl^−^, and SO_4_^2−^ decrease with increasing streamflow due to dilution, but there appears to be stabilization of concentrations (plateaus) for these major ions at the highest levels of streamflow (Fig. 5). However, if salt accumulation in catchments increases during dry years, salt ion concentrations in receiving waters may actually increase with increasing runoff in wet years, as observed in Europe (Kopáček et al. 2017b). Interestingly, concentrations of Ca^2+^ and Mg^2+^ are less controlled by streamflow and hydrology, as compared to the more mobile ions of Na^+^, Cl^−^, and SO_4_^2−^ (Fig. 5). Thus, the potential flushing rates of salt ions from watersheds likely depends upon climate, geology, human activities, flowpaths, and time (Lintern et al. 2023; Kaushal et al. 2023b), and also the different types of salt ions (Fig. 5). Changes in agricultural fertilization and drainage of farmlands are also other important factors. Precipitation and streamflow are increasing in some regions, which could flush and dilute salt ions and contribute to decreasing long-term trends (Murphy and Sprague 2019). However, road salt is accumulating in groundwater and soils at faster rates than it can be flushed out (Cooper et al. 2014), which is contributing to long-term increasing chloride and sodium trends in some watersheds and their receiving waters (Kaushal et al. 2005; Daley et al. 2009; Kelly et al. 2008; Van Meter and Ceisel 2024) (Table 1). Although NaCl has been commonly considered to be inert and mobile, it can be retained up to decades in watersheds (Shaw et al. 2012).

**Table 1:** Examples of retention of sodium and chloride in watersheds around the world

% Cl- retained	Location	Period	Watershed area (km^2^)	References
36%	Chicago, Illinois, USA	30 years (1990–2020)	18,600	Van Meter and Ceisel (2024)
35%	Lake Constance Catchment, Switzerland Germany and Austria	1 year (2006)	11,000	Müller and Gächter (2012)
72%	Minneapolis/St Paul MN, USA	5 years (2000–2005)	4150	Novotny et al. (2009)
40–90%	Southern Ontario, Canada	Each water year 2007–2011	40.5–406	Oswald et al. (2019)
28–45%	Chicago, Illinois, USA	5 months (Nov 1972 to April 1973)	376.5	Wulkowicz and Saleem (1974)
32%	New York, USA	1 year (Nov 1971- Nov 1972)	396	Diment et al. (1973)
52%	New York, USA	1 year (Nov 1970- Nov 1971)	396	Diment et al. (1973)
11–40%	Vermont, USA	1 year (1970)	111.2	Kunkle (1972)
50–65%	Helsinki, Finland	1.5 years (July 1998–Dec 1999)	1.7–24.4	Ruth (2003)
35%	Boston Metro, Massachusetts, USA	4 months (December 1969–March 1970)	168.4	Huling and Hollocher (1972)
55%	Toronto Metro, Ontario, Canada	2 years (1989–1990)	104	Howard and Haynes (1993)
59%	Rochester, New York, USA	1 year (1969–1970)	435	Bubeck et al. (1971)
34–69%	New York, USA	1 year (2012–2014)	1000	Gutchess et al. (2016)
10.8–23.5%	Ontario, Canada	1 year (2004–2005)	27	Meriano et al. (2009)
10–47%	New Hampshire, USA	1 year (Oct 2006–Sept 2007)	1.42–78.5	Trowbridge et al. (2010)
53%	Alberta, Canada	Each water year for 2010–2017	12,971	Laceby et al. (2019)
40%	City of Toronto, Ontario, Canada	Each water year for 2004–2008	100	Perera et al. (2013)
2–62%	Pennsylvania, USA	Annually for 2011–2018	73.9–934	Rossi et al. (2022)

A biogeochemical salt budget for the entire continental U.S. showed that a substantial fraction of anthropogenic salt input is retained in watersheds before reaching streams and rivers (Kaushal et al. 2023a). Salt retention is an important process in other watersheds globally (Table 1). Over a 30-year period, approximately 36% of total inputs of chloride have been retained in watersheds of the Chicago Metropolitan Area, and Cl^−^ is accumulating in groundwater at a rate of 480 kilotons per year (Van Meter and Ceisel 2024). It is important to note that accumulation in groundwater may not always be considered permanently ‘retaining’ salt, rather just redistributing it to a slower moving pool relative to surface water. Over a one-year period, 35% of chloride inputs were retained in the Lake Constance watershed in Europe with only 65% of chloride from anthropogenic sources reaching the lake (Müller and Gächter 2012). An annual chloride budget revealed that 77% of chloride from road salt was retained within a large watershed draining the Minneapolis-Saint Paul metropolitan area in Minnesota, USA, and there was an average annual chloride retention of 72% in 10 of the subwatersheds (Novotny et al. 2009). Chloride retention in a metropolitan region of Canada ranged from 40–90% and was related to urban land use patterns (Oswald et al. 2019). A growing body of work supports the growing importance of quantifying salt storage and retention within soils and groundwater in the future (sensu Shanley 1994) (Table 1).

Although less considered from the perspective of global climate change, impacts on the salt cycle, biological formation of organochlorines can represent an important mechanism for watershed chloride retention and transformation (Kaushal et al. 2023a), in addition to biological uptake and adsorption of Cl^−^ and other anions on Al and Fe oxyhydroxides. Biological organochlorine formation can be enhanced by warming temperatures and faster reaction kinetics. Kopáček et al. (2014a, b) found that on average 14% chloride was retained in agricultural soils, and this percentage was consistent with organochlorine formation in soils. Similarly, Bastviken et al. (2007, 2009) showed that there is chloride retention in Swedish forest soils which ranges from 4 to 40% as a function of temperature (e.g., with warmer soils potentially forming more organochlorines). The effects of climate change and warming temperatures on organochlorine formation warrant further investigation as part of the anthropogenic salt cycle (Kaushal et al. 2023a). Organochlorine formation and salt storage in soils and groundwater can lead to salt retention in watersheds globally (Table 1).

### Part 2: anticipating chain reactions from headwaters to coastal waters from salinization

*Interactive risk 4: Salinization exhibits ‘pulsed, and episodic’ versus ‘sustained and cumulative’ effects due to changing climate that drive chain reactions and formation of harmful chemical cocktails.*

**Fig. 6:**
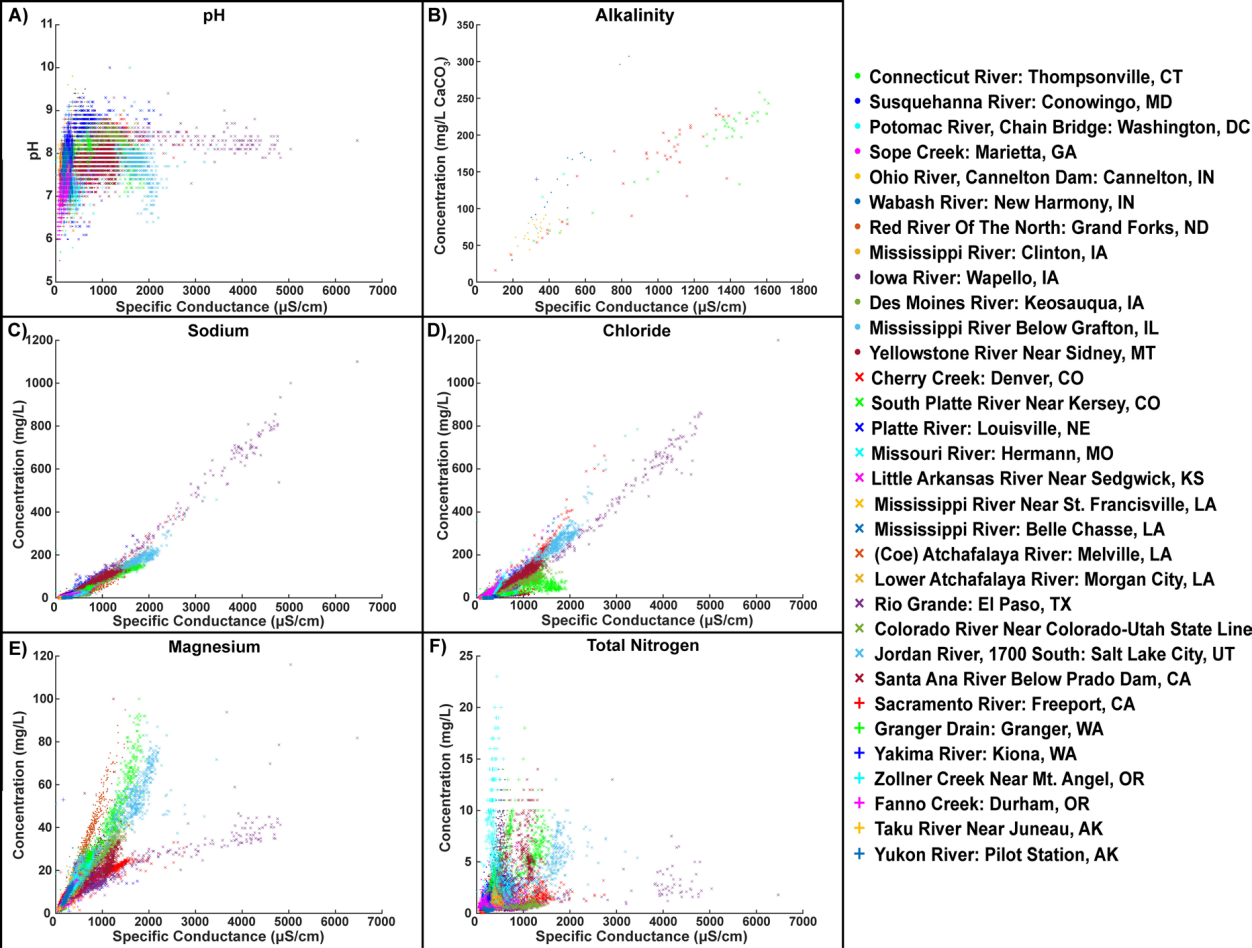
There are positive relationships between specific conductance and concentrations of salt ions and nutrients and dissolved inorganic carbon (as represented by alkalinity) in streams and rivers across the U.S. monitored by the U.S. Geological Survey (USGS). These positive relationships demonstrate that many different elements are either co-mobilized or transported along with salt ions in watersheds. Specific conductance is a surrogate or proxy for many ions (Kaushal et al. 2018b, 2019, 2020, 2021). Information on USGS sites can be found in the Supporting Information

Salt is a strong driver of chain reactions along flowpaths, which can lead to mobilization of secondary contaminants and changes in acidity and alkalinity (Kaushal et al. 2018b, 2019, 2020, 2022; Haq et al. 2018; Galella et al. 2021). For example, there are strong positive relationships between specific conductance and concentrations of salt ions and nutrients in streams and rivers across the U.S. (Fig. 6). Many different elements are either co-mobilized (through biogeochemical reactions) or transported along with salt ions to maintain charge balances in watersheds (Kaushal et al. 2018b, 2019, 2020, 2024) (Fig. 6). There are also plateau or threshold relationships between specific conductance and pH due to influence of salt ion mixtures and alkalinity on acid buffering capacity (Fig. 6), which have been described in previous work linking salinization and alkalinization in streams and rivers across the U.S. (Kaushal et al. 2013, 2018a). Thus, salt has the potential to trigger chain reactions among many different elements, which may be either both short-term (pulses) or sustained and cumulative changes.

**Fig. 7:**
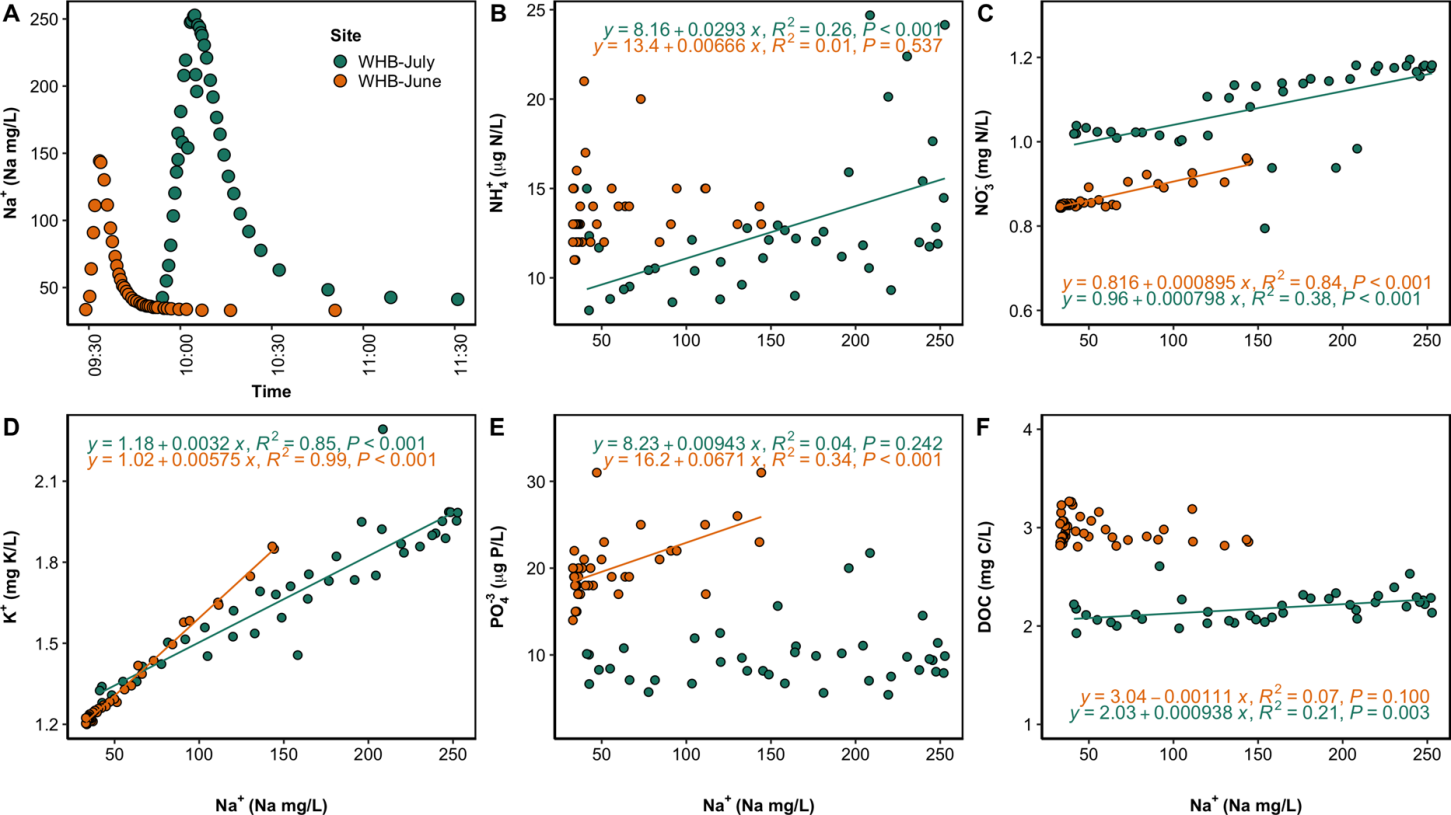
Experimental NaCl tracer additions in a suburban New Hampshire stream (WHB) trigger “fast” mobilization of dissolved organic carbon (DOC), nitrogen (N), phosphorus (P), and potassium (K). A) Break-through curve of Na^+^ concentrations from the added NaCl at WHB; Regressions between added Na^+^ concentrations and ambient concentrations of B) ammonium (NH_4_^+^), C) nitrate (NO_3_^−^), D) potassium (K^+^), E) phosphate (PO_4_^3−^), and F) dissolved organic carbon (DOC). Green points are from July 2015 and yellow points are from June 2014. Each point represents a single sample, and linear regressions were only included for statistically significant (p < 0.05) relationships. The effects of experimental stream salinization may vary with streamflow and season. Information on tracer addition methods can be found in the Supporting Information

Changes in the frequency and magnitude of salinity pulses from winter road salt events, irrigation return flows, agricultural runoff, saltwater intrusion events, droughts, and other climatic factors have the potential to trigger mobilization of secondary contaminant pulses (Ardón et al. 2013; Kaushal et al. 2018b, 2019; Galella et al. 2023a, b a,b). For example, salinity pulses can lead to the sequential extraction of elements from soils and sediments in freshwaters (e.g., adsorption and solubility changes with ionic strength and pH, ion exchange, mineral dissolution, redox effects, changes in alkalinity, hardness and toxicity) (Kaushal et al. 2019, 2021, 2024) (Fig. 6). In addition, there can be ion pairing during salinity pulses leading to temporary bonds that allow Na^+^ and Cl^−^ ions to “pull” many other ions such as nitrate, phosphate, sulfate and others within ground and surface waters during pulsed winter road salt, fertilizer runoff, and saltwater intrusion events (Kaushal et al. 2024). Salt tracer experiments in suburban stream ecosystems can result in mobilization of ammonium (NH_4_^+^), nitrate (NO_3_^−^), potassium (K^+^), phosphate (PO_4_^3−^), and dissolved organic carbon (DOC) and significant linear relationships between these chemicals and added Na^+^ concentrations (Fig. 7). However, the effects of NaCl on mobilization of DOC and nutrients in stream likely varies across streamflow, seasons, and land use (Fig. 7). Salinization can also trigger episodic pulses of toxic metals and nutrients from sediments to streams and rivers (Kaushal et al. 2019, 2022).

In addition to pulsed and episodic effects, we also need to anticipate sustained and cumulative effects from chronic salinization due to legacy salt retention in watersheds during dry conditions or prolonged saltwater intrusion. Sustained effects from chronic salinization are related to storage of salt and chain reactions in soils, groundwater, streams, rivers, reservoirs, and estuaries (sensu slow changes described in Michael et al. 2005, 2017). Groundwater storage of salt contributes to corrosivity and mobilization of radium, radionuclides, and metals (McNaboe et al. 2017; Lazur et al. 2020; Kaushal et al. 2024). Slow effects of salinization can alter major cycles of carbon, nitrogen, phosphorus, sulfur, iron, and silica, which control the productivity, functioning, and biodiversity of non-tidal and tidal freshwater ecosystems (Herbert et al. 2015; Luo et al. 2019) (e.g., Fig. 2). In coastal rivers and wetlands, chronically elevated salt ion concentrations from repeated and frequent saltwater intrusion can reduce the solubility of gasses including dissolved O_2_ (Supporting Information Fig. S1), which could eventually trigger changes in redox potentials (Supporting Information Fig. S2) and alter reactions in sediments or stratified bottom waters affected by hypoxia or anoxia (Luo et al. 2019). The combination of deoxygenation and ion exchange from salinization can also mobilize redox-sensitive metals such as Mn, Fe, and Cu from sediments into solution (Supporting Information Fig. S3), which has implications for mobilization of other metals and contaminants bound to Mn and Fe oxyhydroxides. Future predictions need to anticipate both pulsed and episodic *vs.* sustained and cumulative biogeochemical reactions associated with salinization.

*Interactive risk 5: Salt pollution pulses and saltwater intrusion events will contribute to “fast” pulses of temporary acidification but “slow” and sustained long-term alkalinization.*

Over time, pulsed Na^+^, Ca^2+^, Mg^2+^, and K^+^ inputs from winter road salt, other salt pollution sources, and saltwater intrusion can contribute to continual alkalinization chain reactions from the displacement of H^+^ ions from soil and sediment exchange sites by base cations and episodic acidification (Kaushal et al. 2018a, b; Kaushal et al. 2024). The input of sea salts onto coastal soils during atmospheric deposition events can also be important for episodic acidification and the mobilization of H^+^ (and other ions) in acidic soils (Wright et al. 1988). On an ion basis, inputs of Mg^2+^ and Ca^2+^ ions have the potential to displace more H^+^ ions than Na^+^ based on their size and charge (Hussein and Rabenhorst 2001). Initially, this would lead to ‘fast’ temporary acidification and pH depression (Kaushal et al. 2022; Ury et al. 2023), and then, sustained and cumulative long-term alkalinization as H^+^ becomes depleted on soil ion exchange sites. In areas affected by saltwater intrusion, soil alkalinization is controlled by the accumulation of sodium and other exchangeable base cations, in addition to the displacement of H^+^ ions (Hussein and Rabenhorst 2001; Arslan and Demir 2013).

There have been increasing alkalinity trends in streams, rivers, and seas across diverse world regions (Raymond et al. 2008; Kaushal et al. 2013,2017, 2018a; Stets et al. 2014; Drake et al. 2018; Müller et al. 2016; Najjar et al. 2020) due to accelerated weathering, decreases in atmospheric acid deposition, increasing production and use of alkaline salts, and cumulative depletion of H^+^ from soil ion exchange sites from increased Na^+^ and salinization (Kaushal et al. 2013, 2017, 2023a). Another important pattern of changing climate is the increased movement of dust (salts, base cations, and P) from deserts to aquatic ecosystems (Brahney et al. 2014). The increase in alkalinity and acid neutralizing capacity has increased pH. Specifically, there have been rising trends in pH in 66% of streams and rivers draining the continental U.S. (Kaushal et al. 2018a), including the Mississippi River and Chesapeake Bay tributaries (Turner 2021; Waldbusser et al. 2011). Human activities are now regulating alkalinity and pH trends and pulses (both increases and decreases) on a global scale. Increased pH may decrease carbon dioxide (CO_2_) evasion and cause some alkaline streams and rivers to become sinks for CO_2_ (Dubois et al. 2010). On the other hand, increasing DOC and decomposition may contribute to acidification in some cases and lead to increases in pCO_2_ (Couturier et al. 2022). In a wetter or more variable climate, increases in the delivery of alkalinity loads in rivers could contribute to variability in coastal ecosystem responses to ocean acidification and alkalinization of estuaries and coastal waters (e.g., Fig. 3). While an increase in alkalinity and pH is beneficial to streams recovering from acidic precipitation in response to the Clean Air Act Amendments in the U.S. (Likens et al. 1996), it can also alter changes in absorption of CO_2_ from the atmosphere, changes in ammonia toxicity, phosphorus sorption or desorption from sediments, changes in organic matter solubility and carbon cycling, and have effects on primary productivity, aquatic life, and food webs (Kaushal et al. 2013). Thus, a future challenge will be to better understand the potential connections between salinization and alkalinization of inland waters and estuaries across time and space from increases in ion exchange, alkalinity generation from chemical weathering, and other biogeochemical processes.

*Interactive risk 6: Salt pollution and saltwater intrusion will alter the quantity and quality of organic carbon in freshwaters.*

**Fig. 8:**
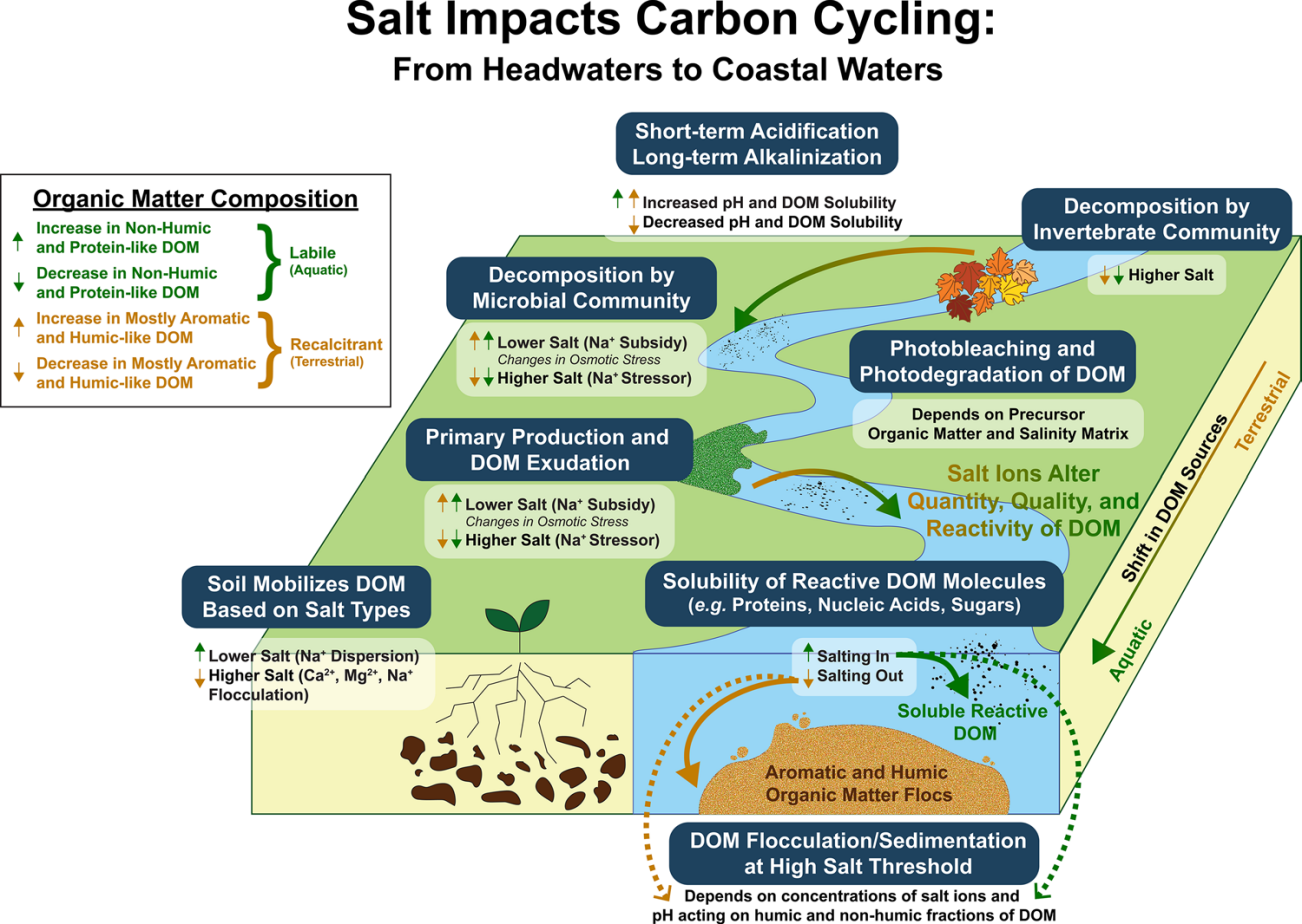
Salt impacts carbon cycling and bulk dissolved organic matter (DOM) concentrations, as well as different fractions of DOM through physical, biological, and chemical processes. These processes can result in net increases or decreases in DOM concentrations or changes in DOM quality from headwaters to coastal waters. Here, we summarize how changes in salinity impact: (1) bulk DOM; (2) recalcitrant DOM, which is often considered to be aromatic, high molecular weight humic-like substances typically terrestrial and soil derived (shown in brown arrows as absolute amount; Hansen et al. 2016); and (3) labile DOM, which is often considered to be less complex aliphatic, protein-like substances typically microbial and plant leachate derived (shown in green arrows as absolute amount; Hansen et al. 2016). Initially, pulses in salinity cause rapid decreases in pH (Kaushal et al. 2022), making DOM, including humic fractions, less soluble in soil, water, and streams (Green et al. 2009; Duan and Kaushal 2015). Over longer time scales, elevated salinity can cause alkalinization through repeated H^+^ depletion on soil exchange sites, enhancing solubility of DOM, particularly aromatic and humic fractions (Green et al. 2008, 2009; Duan and Kaushal 2015; Haq et al. 2018). In soils and sediments, sodium dispersion can mobilize organic matter through the destruction of soil aggregates, and it can increase bulk DOM, as well as aromatic and non-humic fractions up to a threshold before flocculation (Amrhein et al. 1992, Green et al. 2008b, 2009, Duan and Kaushal 2015). Along riparian zones and streams, invertebrate decomposers are impacted by salinity thresholds, which alter decomposition rates and concentrations of fine particulate organic matter, bulk DOM, and changes in DOM quality (Entrekin et al. 2019; Berger et al. 2019). Within streams and rivers, elevated salinity can pose a subsidy-stress relationship to primary producers and heterotrophic microbial communities. Low levels of added salinity initially cause decreases in osmotic stress, leading to increases in primary production and decomposition rates (Entrekin et al. 2019; DeVilbiss et al. 2024), which could increase bulk DOM and protein-like and microbially derived fractions of organic matter through exudation. As salinity thresholds are exceeded, increases in osmotic stress can lead to decreases in primary production and decomposition rates, leading to decreases in overall DOM concentrations and larger relative contributions of recalcitrant material (Entrekin et al. 2019). Cell death due to desiccation or cell lysis caused by osmotic stress can increase the contribution of aquatically sourced DOM in streams (Duan and Kaushal 2015; Kaushal et al. 2022). Along river flowpaths, salinity gradients along the freshwater-marine continuum can affect whether different fractions of DOM are degraded, oxidized, or mineralized by sunlight, UV radiation, and photobleaching based on upon the chemical composition of organic matter and the salt ion matrix (Minor et al. 2006; Schafer et al. 2021). Along the entire freshwater-marine continuum, increasing ionic strength also increases the solubility of proteins up to a threshold, which depends upon salt ion compositions and DOM substrate composition (salting-in), after which hydrogen bond locations are taken up and solubility decreases (salting out) (Kaushal et al. 2022; Hyde et al 2017). Across increased salinity levels, Ca^2+^, Mg^2+^, and Na^+^, can lead to flocculation of organic matter; flocculation can remove aromatic and humic fractions preferentially and decrease bulk DOC concentrations (Abolfazli and Strom 2021; Duan and Kaushal 2015). Overall, salinity affects the cycling of carbon from headwaters to coastal waters in many environmentally significant ways based on: organic substrate composition, concentrations and compositions of the salt ion matrix, previous salt exposure histories at sites, microbial communities and adaptations to osmotic stress, and other site-specific factors

The future impacts of salinization on the concentration, composition, and structure of dissolved organic carbon (DOC) also warrant attention. The cumulative effects of different salt ions on the quantity and quality of organic matter and DOC have not been synthesized to our knowledge (Fig. 8); this knowledge gap connecting salinization with the cycling of DOC extends from headwaters to coastal waters (Fig. 8). Although not fully understood yet, we propose that impacts of salinity on organic matter and DOC depends upon six primary factors: (1) salinity ranges, (2) pH ranges, (3) ion mixtures dominated by Na^+^ (dispersant of colloids at certain concentrations) or Ca^2+^ (coagulant of colloids), (4) the composition of organic substrates exposed to salt (Duan and Kaushal 2015; Haq et al 2018), (5) salt exposure history and microbial communities at sites (Ury et al. 2023), and (6) dissolved oxygen and redox conditions and redox-sensitive metals with changing salinity (Supporting Information Figs. S1–S3). The relative importance of these six primary factors can be site specific (Fig. 8). There may even be subsidy stress responses related to Na^+^ where organic matter decomposition rates are slower in the presence of Na^+^ (Tyree et al. 2016; Gruntz et al. 2022; DeVilbiss et al. 2024), which cause nonlinear effects of increasing salinization on decomposition. Ion mixtures may also affect decomposition rates (Martínez et al. 2020) (Fig. 8). Some of the changes (or lack thereof) in DOM composition or concentrations could be due to an absence or reduction in certain groups/taxa of microbes with different sensitivities to salt, and future work needs to link salinization with changes in organic matter amounts and quality and microbial communities.

**Fig. 9:**
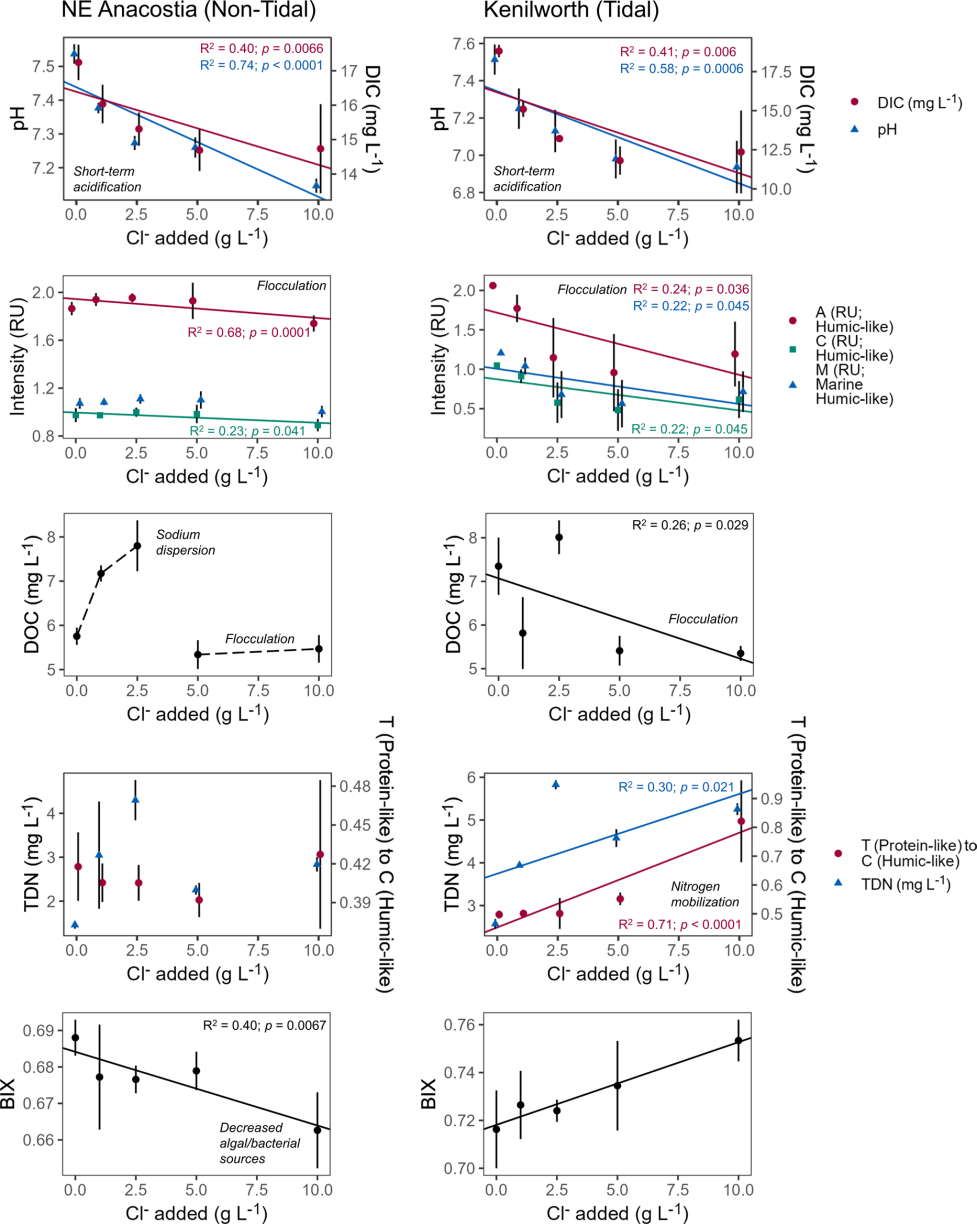
Experimental NaCl impacts on pH, dissolved inorganic carbon (DIC), total dissolved nitrogen (TDN), and humic fractions of dissolved organic matter (DOM) in sediments from non-tidal and tidal freshwater sites along the Anacostia River. Results are from an original experiment with further details provided in Supporting Information. Organic matter indices were identified using staRdom in R (Pucher et al. 2019), with a higher value of BIX representing a larger contribution of recent autochthonous material (Huguet et al. 2009) and higher values of Coble’s Peaks (A, C, M, and T) representing larger amounts of protein-like and humic-like organic matter, as associated with each peak given in parentheses (Coble 1996). RU denotes Raman Units, which is followed by the description of the peak (Coble 1996). T (Protein-like) to C (Humic-Like) is the ratio between Coble’s Peak T to Coble’s Peak C, with higher values indicating a larger relative contribution of protein-like material. Experimental methods and additional results are in the Supporting Information

Distinct fractions of dissolved organic matter (DOM) respond differently to discrete salt ion pulses and mixtures across varying pH ranges (Figs. 8 and 9, Supporting Information Figure S4). Increased Na^+^ from winter road salt pulses and saltwater intrusion events can cause dispersion of DOM and then flocculation at higher salinities (Duan and Kaushal 2015; Haq et al. 2018) (Figs. 8 and 9). For example, NaCl pulses can enhance mobilization of DOM across certain ranges of concentrations, dissolved organic nitrogen (DON), and protein-like fractions in roadside soils and urban stream sediments due to a combination of increases in soil pH and increased solubility, denaturing of proteins, and dispersion of organic matter (Amrhein et al. 1992; Green et al. 2008; Duan and Kaushal 2015) (Fig. 8). Conversely, Ca^2+^ and Mg^2+^ form a bridge between mineral surfaces and organic matter, which decreases DOC solubility and increases DOC flocculation. The phases and concentrations of Fe are also important in governing DOC behavior in this context as well. There can also be decreased solubility and increased ‘salting out’ of DOM and also some metals at higher concentrations of salt; this has important implications for DOM reactivity and bioavailability and also contaminant partitioning between dissolved and particulate phases (Turner 2003). Changes in salt concentrations and pH alter optical properties of organic matter and the rate and proportion of DOC that is photochemically oxidized and broken down by solar radiation to respective C-oxides and lower molecular weight DOC (Fig. 8); these forms of carbon can be more available for microbial degradation (e.g., Kopáček et al. 2003). Thus, salinity interacts with site-specific chemical mixtures to alter the amounts, forms, and chemical and biological reactivity of dissolved organic matter from headwaters to coastal waters (Figs. 8 and 9).

**Fig. 10:**
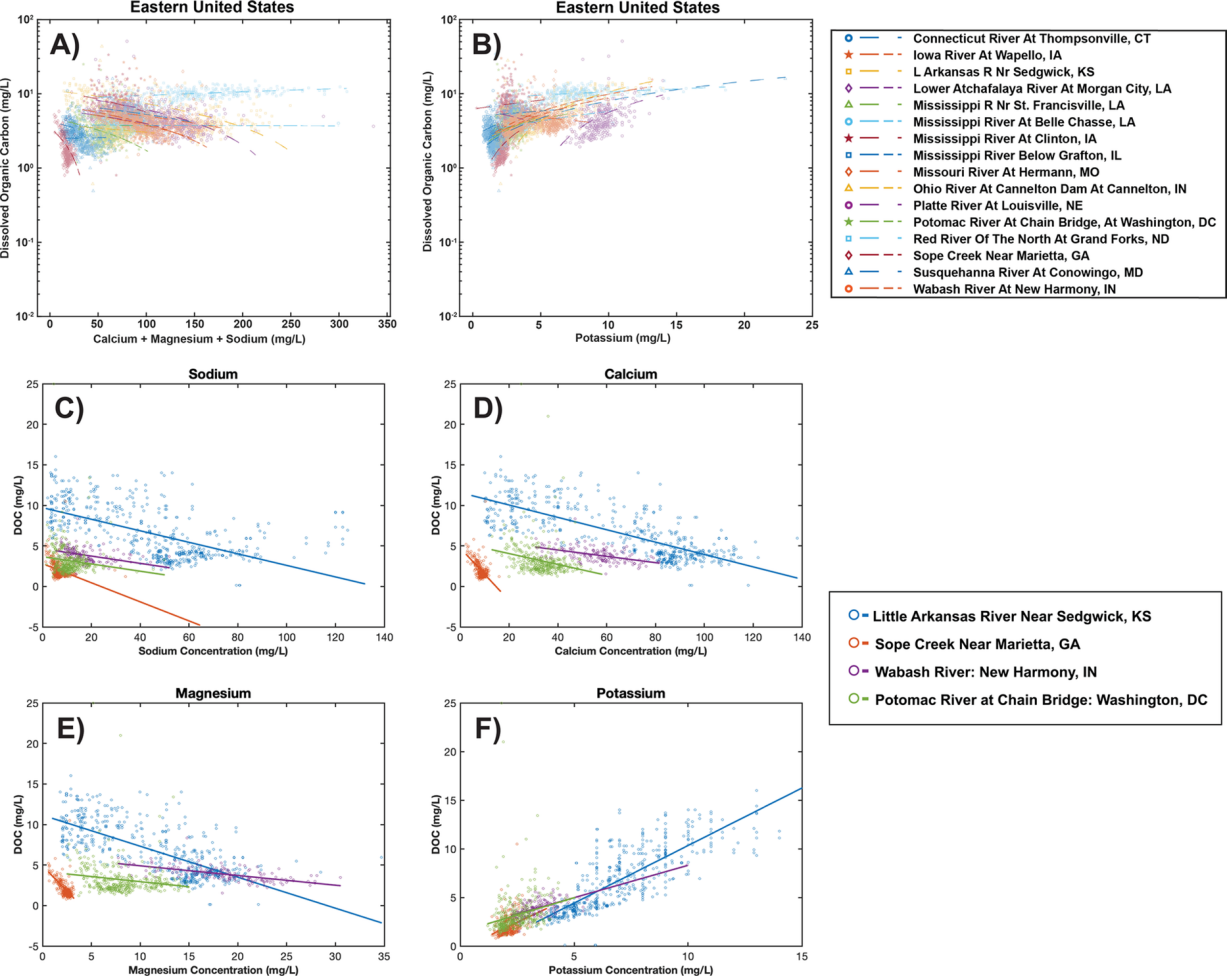
Relationships between Na^+^, Ca^2+^, and Mg^2+^ (total sum of these three base cations), and K^+^ and dissolved organic carbon concentrations in streams and rivers across the U.S. In addition, we present examples of relationships between dissolved organic carbon (DOC) and individual ions at specific sites. Concentrations of DOC appear to increase initially with elevated concentrations of Na^+^, Ca^2+^, and Mg^2+^ (sum of these base cations) across sites in the Eastern U.S., but then decrease likely as ionic strength and coagulation increases. However, there are typically declining relationships between DOC concentrations and concentrations of Na^+^, Ca^2+^, and Mg^2+^ at individual local sites. Typically, K^+^ concentrations show a strong positive relationship with DOC concentrations, which is likely because of the importance of biological controls on cycling of potassium; potassium is a limiting nutrient in terrestrial systems (Tripler et al. 2006). Information on U.S. Geological Survey (USGS) sites can be found in Supporting Information Table S1

Further insights into the effects of salinity on concentrations of DOC can be gained from analyzing broader patterns across sites and analyzing relationships between DOC and salt ions at individual sites. Across the eastern U.S., there are decreasing relationships between DOC concentrations and concentrations of Na^+^, Ca^2+^, and Mg^2+^ at some U.S. Geological Survey stream sites; this can be related to the effects of increasing ionic strength on coagulation and flocculation of DOC or a general inverse relationship between DOC and base cations due to a shift in their sources across shallow vs. deep flowpaths (Fig. 10). However, a slightly different pattern emerges across all sites. Concentrations of DOC appear to increase initially with elevated concentrations of Na^+^, Ca^2+^, and Mg^2+^ potentially due to sodium dispersion effects on organic matter in soils at sites that represent the lower range of salinity, but then decrease as ionic strength and coagulation and flocculation generally increase (Fig. 10). There may be competing effects of different salt ions and pH on enhancing solubility within certain ranges in salt ion concentrations and compositions *versus* enhancing flocculation within other ion concentration and composition ranges. Typically, K^+^ shows a strong positive linear relationship with DOC across U.S. Geological Survey sites (Fig. 10), which is likely because of its biological importance as a limiting nutrient in terrestrial systems (Tripler et al. 2006). Overall, our analysis raises new questions regarding the relationships between different salt ions and the quantity, quality, and reactivity of DOC transported along streams, rivers, and estuaries.

In some cases, particularly when there is lower pH or temporary fast acidification (Fig. 9), salt pollution and saltwater intrusion can lead to DOC decreases likely due to changes in solubility or flocculation (Duan and Kaushal 2015; Ardón et al. 2016; Haq et al. 2018; Ury et al. 2023) (Figs. 8 and 9). For example, salt pulses at very high salinities can lead to changes in particle size distribution, flocculation of organic matter and inorganic colloids, and sedimentation in streams and lead to streambed clogging, which impact benthic habitats (Abolfazli and Strom 2021). At lower levels of salinity, salinization can actually lead to DOC increases due to dispersion from Na^+^ at higher pH ranges (Amrhein et al. 1992; Green et al. 2008; Duan and Kaushal 2015; Haq et al. 2018) (Figs. 8 and 9). Thus, the long-term impacts of salinization on DOM concentrations and quality depend on the balance of various processes such as short-term acidification, long-term pH increase, sodium dispersion, and coagulation and flocculation of DOM due to higher ionic strength and/or increasing calcium and magnesium concentrations (Green et al. 2008; Hruska et al. 2009; Abolfazli and Strom 2021) (Figs. 8 and 9). In particular, the effects of different ion mixtures and chemical cocktails containing Na^+^, Ca^2+^, Mg^2+^, and K^+^ on organic carbon quantity, quality, and microbial decomposition warrants consideration in a changing climate, particularly in freshwaters experiencing alkalinization and higher pH (Fig. 8).

*Interactive risk 7: Salinity pulses from changing weather, salt pollution, and saltwater intrusion will alter nitrogen cycling from headwaters to tidal waters.*

From a watershed perspective, salinization has the potential to extract N and other nutrients from soils and sediments and increase N transport to ground and surface waters under some conditions and seasons (Duan and Kaushal 2015; Haq et al. 2018; Kaushal et al. 2019; Kinsman-Costello et al. 2023). Winter road salt mobilizes ammonium from soil exchange sites near roads and increases soil pH in acidic soils by displacing H^+^ ions (Green and Cresser 2008) (discussed in prediction 5). The increase in pH enhances mineralization of organic N and nitrification in these roadside soils, which may increase N transport to ground and surface waters (Green and Cresser 2008). Winter salinity pulses from road salt can suppress denitrification in some cases (Hale and Groffman 2006) and enhance dissimilatory nitrate reduction to ammonium (Inamdar et al. 2024). Interestingly, the potential for added NaCl to significantly mobilize nitrogen from soils and sediments may also be related to an increase in mobilization of biologically labile organic matter and organic N that may be rapidly mineralized to inorganic N (Fig. 9). Although less considered from a climate change perspective, salinization can alter the magnitude and timing of nitrogen delivery to receiving waters during winter months when road salt is applied (Galella et al. 2023b). From a watershed perspective, salinization influences the cycling of N and multiple bioreactive elements together (Duan and Kaushal 2015).

In response to sea level rise and saltwater intrusion, tidal freshwaters become less of an N sink and more of an N source when considering fast biogeochemical responses (Osborne et al. 2015). For example, drought can lead to saltwater intrusion and fast N mobilization from coastal agricultural fields (Ardón et al. 2013). Slow and chronic saltwater intrusion can also lead to large mobilization of inorganic N to tidal freshwater marshes (Widney et al. 2019). In addition, saltwater intrusion can also enhance mobilization of NH_4_^+^ from sediments to the water column of estuaries through pairing of NH_4_^+^ with base cations from sea salts (Gardner et al. 1991); the process of ion pairing refers to the association of oppositely charged ions in solution by electrostatic Coulombic forces without forming covalent bonds, which also influences movement of ions through watersheds (Kaushal et al. 2024). Shifts in microbial community composition and function from salt stress can also shift the predominant N cycling pathways, water quality, and ecosystem services in estuaries (Franklin et al. 2017; Dang et al. 2019; Neubauer et al. 2019; Batanero et al. 2022; Feng et al. 2023). Nitrate (NO_3_^−^) is a major N form in estuaries, and it can be assimilated into biomass or transformed via two dissimilatory pathways: denitrification and dissimilatory nitrate reduction to ammonia (DNRA). Denitrification sequentially converts NO_3_^−^ to NO_2_^−^, NO, N_2_O, and N_2_. Denitrification has the potential for permanently removing N from the ecosystem as N_2_ gas, but saltwater intrusion can lead to incomplete denitrification and production of the greenhouse gas N_2_O (Jiang et al. 2023). In contrast, DNRA reduces NO_3_^−^ to ammonium (NH_4_^+^), conserving N in the ecosystem.

Previous studies have shown that denitrification is the dominant pathway in freshwater and intermediate salinities and generates alkalinity (biologically mediated alkalinization). The relative importance of DNRA increases in more saline waters, which could alter the fate of N and decrease N removal via denitrification in estuaries (Gardner et al. 2006; Seo et al. 2008; Jiang et al. 2023; Gervasio et al. 2023; Huang et al. 2024). For example, DNRA is more important in C-rich systems with high C:N ratios like sediments in estuaries. The potential for significant DNRA exists in most soils also, but is more important under anoxic conditions in microsites rich in low molecular weight C sources, and at high soil bioavailable DOC to NO_3_^−^ ratios (Rütting et al. 2011). Over both fast and slow time scales, there can be greater mobilization of inorganic N from sediments in saltier and more alkaline tidal rivers and marshes in response to saltwater intrusion events and sea level rise.

*Interactive risk 8: Salinity pulses from climate change will amplify corrosion risks and reactions with infrastructure from headwaters to coastal waters.*

Infrastructure corrosion costs billions of dollars per year, representing approximately 3% of the gross domestic product of the U.S. (Koch et al. 2005) and 1–6% of the gross domestic product of South Korea (Kim et al. 2011). Most of the costs are associated with sectors such as drinking water and sewer systems, transportation, and defense, which can be affected by salinization of groundwater, inland waters, and coastal tidal waters (Koch et al. 2005). Corrosivity is commonly estimated by the ratios of the concentrations of chloride and sulfate ions to the concentrations of bicarbonate and carbonate ions (alkalinity) (e.g., Edwards and Triantafyllidou 2007; Stets et al. 2018; Edwards et al. 1996). Concentrations and mixtures of major ions influencing corrosion are shifting across local, regional, continental, and global scales (Kaushal et al. 2005, 2013, 2018a, b, 2019, 2021, 2023a), which could influence corrosion potential of freshwaters across “fast” and “slow” time scales.

As one example, pulses of Cl^−^ have been increasing in streams and rivers from road salt pollution and climate variability (Figs. 3 and 4), and there have been long-term decreases in SO_4_^2−^ loads in rivers from acid precipitation regulations and changes in application rate or composition of synthetic fertilizers (Fig. 3). Long-term diverging trends and pulsed changes in the Cl^−^ to SO_4_^2−^ mass ratio (Fig. 3) can trigger fast corrosion events, which can affect the mobilization of Pb, Cu, and other metals from drinking water pipes in the absence of adequate corrosion inhibitors (Pieper et al. 2017, 2018). As highlighted in Risk 5, pulsed salinity events can occur during periods of temporary acidification and reduced alkalinity during storms or road salt events (Kaushal et al. 2018a, b, 2022), which could increase corrosion risks into the future because of more saline conditions. A notable example of the ‘fast’ effects of chloride contamination (from road salts) on mobilization of metals occurred in the drinking water supply of Flint, Michigan, U.S.A. when the city failed to add proper amounts of corrosion inhibitors and test for elevated concentrations of lead and copper in drinking water (Pieper et al. 2017, 2018). Cascade events are also possible, where salinity pulses in a watershed release nitrate to drinking water (e.g., Galella et al. 2023b), which in turn, can mobilize lead in finished drinking water (Lopez et al. 2022).

In addition, climate variability can influence moisture and salinity exposure in concrete structures through acute fast extreme weather events and slow, long-term, and prolonged exposure to moisture and salt via rain, snow, and road salt applications. Chloride-induced corrosion is a major deterioration mechanism of concrete (e.g., reinforced concrete used in buildings, parking garages, etc*.*) and steel structures (e.g., bridges), and is a motivation for infrastructure design strategies and planning for infrastructure life cycles based on environmental exposure conditions (Ahmad 2003). Concrete is porous, and there is a need to better consider penetration of water contaminated with chlorides when predicting corrosion of reinforced steel structures supporting concrete bridges, tunnels, and roadways (Aldea et al. 1999). There are different mixtures of concrete used for different applications and some concrete mixtures are more resistant to salinity and moisture than others (Yildrim et al. 2011). Typically, concrete mixtures and infrastructure designs are based on current climate conditions, but designs do not always consider future changes in moisture, salinity, and pH (Stewart et al. 2011); these future changes can manifest as both fast pulses and slow trends over time. Overall, changes in salinization will affect the service life of infrastructure exposed to pulsed salinity events and understanding how those changes will affect infrastructure design, maintenance and inspection, financing, and failure risks poses a looming conundrum (Stewart et al. 2011).

### Part 3: anticipating double trouble: ecosystem transitions where salt pollution from land meets saltwater intrusion

*Interactive risk 9: Climate-driven changes in streamflow, human activities, and sea level rise will interact to alter saltwater intrusion and ecosystem transitions.*

Effects of freshwater salinization have been studied separately in non-tidal and tidal waters. More work on understanding impacts of climate change on salinization have focused on soils, groundwater, or coastal forests and wetlands (e.g., Kirwan and Gedan 2019; Tully et al. 2019a, b; White et al. 2022; Mondal et al. 2023). Relatively less work has focused on the effects of climate change on salinization and alkalinization of tidal rivers and estuaries because of disciplinary divides among scientists along nontidal and tidal boundaries (but see Hall et al. 2023). Tidal freshwater areas or low salinity zones are the nexus of freshwater and marine waters, and tidal freshwater habitats may be most at risk from salinization across space and time. Tidal freshwaters are likely more sensitive to shifts in salinity due to their previous exposure history to low salinity conditions, the rapid encroachment of salt fronts during droughts, and the combined impacts of increased watershed salt pollution to estuaries.

During periods of warmer temperatures and droughts, water withdrawals and pumping are expected to increase (Van Vliet et al. 2023) and can increase saltwater intrusion rates and decrease dilution of salinity in tidal rivers (Barlow and Reichard 2010; Roehl et al. 2013). A greater frequency and magnitude of droughts is predicted in the future with warmer temperatures (Cook et al. 2018), which may increase upstream freshwater demand and withdrawals. Flash droughts, which develop more suddenly than prolonged droughts, are increasing over 74% of global regions identified by the Intergovernmental Panel on Climate Change (Yuan et al. 2023), and flash droughts could impact the magnitude, timing, and extent of saltwater intrusion events along tidal rivers. Runoff in rivers influences the landward encroachment of the salt front in tidal rivers and estuaries (Tian 2019), and decreasing river discharges to coastal zones can be further reduced by upstream dams, human water use, and hydrologic alterations.

**Fig. 11:**
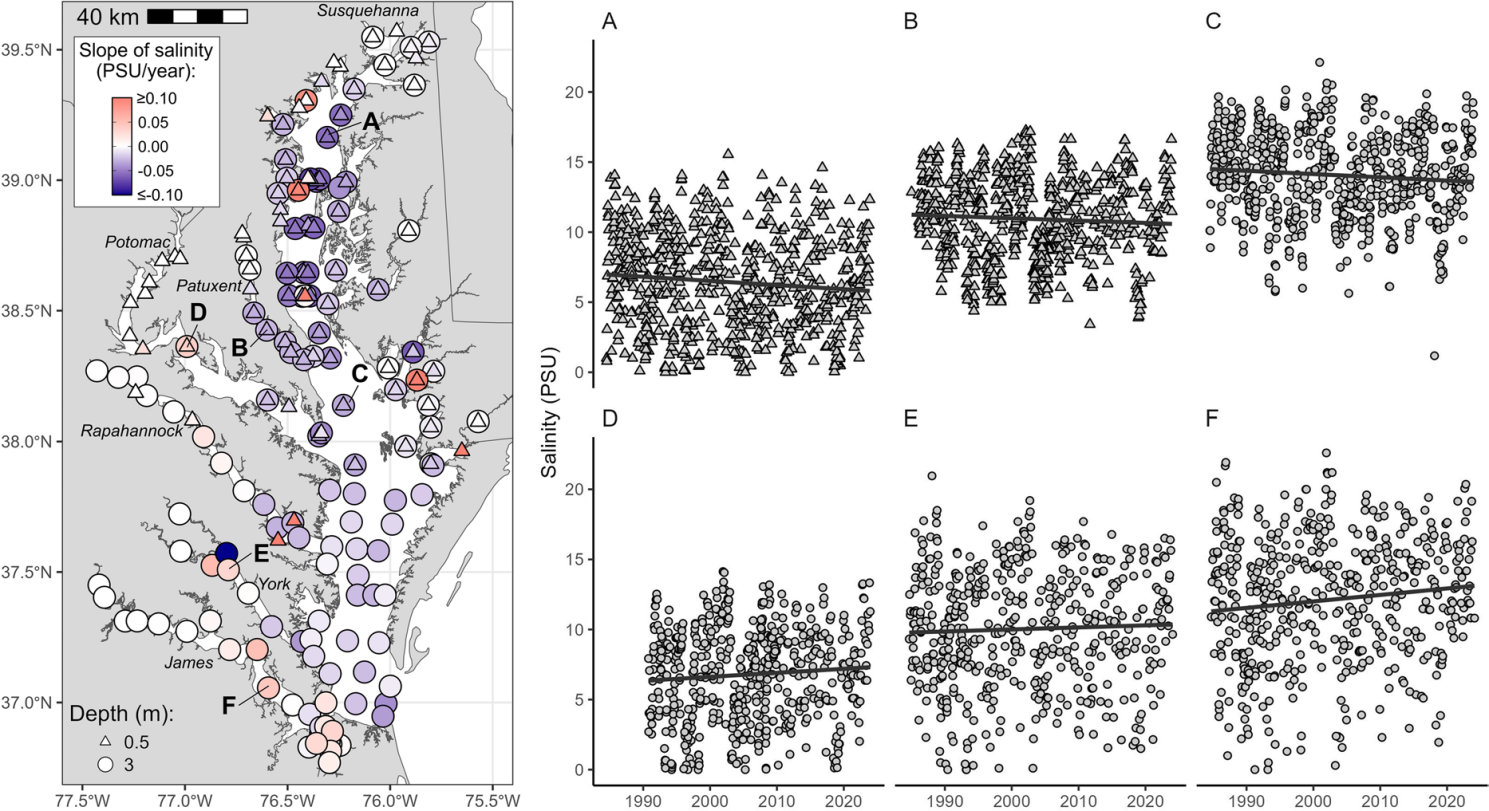
A long-term trend analysis of salinity for tidal sites within the EPA Chesapeake Bay Program water quality monitoring network. Long-term trends at sites were only assessed if sites had 10 + years of data and 50 + observations. Theil-Sen slopes are shown for the trends at specific stations as examples; in addition, the colors of points on the map represent ranges in Theil-Sen slopes for individual stations within the tidal monitoring network. Trends in figures A-F are statistically significant and PSU in the figure legend refers to practical salinity unit. (Panels A, B, and C) Long-term trends in salinity throughout the Chesapeake Bay. The mainstem of the Chesapeake Bay shows long-term “freshening” or decreasing salinity trends due to increased streamflow from the Susquehanna River over recent decades. In contrast, we observed increasing salinity trends in many of the tidal rivers flowing into the Chesapeake Bay during the same time period. (Panels D, E, F) There have been increasing long-term salinity trends along the Potomac, Rapahannock, York, and James Rivers; while examples of time series are only shown for a subset of these rivers, all monitoring stations with increasing salinity trends can be seen in shades of red on the map. The names of some of the major tributaries flowing into Chesapeake Bay are labeled on the map for geographic reference. Information about trends at each station (e.g., years of record, sample sizes, slopes, p values, and confidence intervals) can be found in Supporting Information Table S2

Changes in streamflow can have profound impacts on salinization versus freshening responses in estuaries from days to decades. As one example, we have observed increasing freshening trends over decades throughout the mainstem of the Chesapeake Bay (Fig. 11) due to increasing precipitation and streamflow in the Susquehanna River (e.g., Zhang et al. 2010a). These long-term freshening trends throughout the mainstem of the Chesapeake Bay suggest the importance of understanding regional balances between opposing forces of sea level rise and streamflow when anticipating net effects of saltwater intrusion risks along estuaries and tidal rivers. The freshening trends are strongest in the Upper Chesapeake Bay due to the proximity of the Susquehanna River (Fig. 11A–C), and observations of increasing streamflow in rivers draining the northeastern U.S. (Rice et al. 2017). In contrast, we observed increasing salinity trends in many of the tidal rivers flowing into the Chesapeake Bay during the same time period as freshening trends along the mainstem of the Chesapeake Bay. For example, we documented increasing long-term salinity trends in stations along the Potomac, Rapahannock, York, and James Rivers (e.g., Fig. 11D–F and any other stations on the map shown in shades of red). Interestingly, the number of stations with increasing salinity trends increases in a southward direction with the James River showing the most stations experiencing salinization. The increasing salinity trends in tributaries of the Chesapeake Bay, particularly rivers in Virginia, are likely driven by decreasing streamflow in the southeastern U.S. (Rice and Hirsch 2012; Rice et al. 2017).

**Fig. 12:**
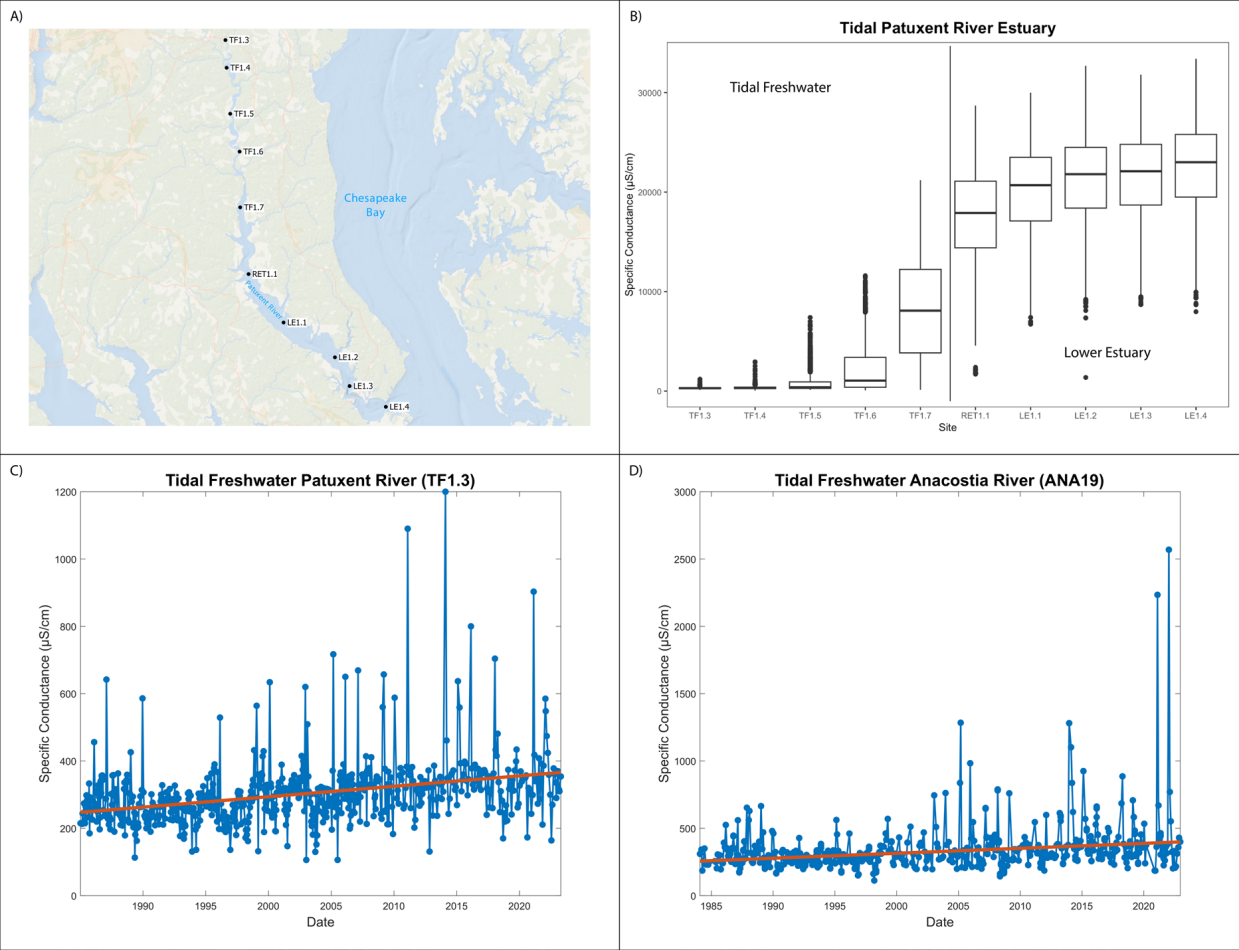
Salinization is impacting tidal freshwater rivers of the Chesapeake Bay according to an analysis of data from the EPA Chesapeake Bay Program. Tidal freshwaters of the Patuxent River estuary are becoming more salty, whereas salty portions of the lower estuary are becoming fresher. (Panels A and B) There is an increase in salinity pulses in tidal freshwaters, and there is an increase in pulses of fresher water in the saltier lower estuary. (Panels A and B) There are extreme outliers in very high specific conductance in tidal freshwater reaches, and there are extreme outliers in low specific conductance in the lower estuary due to floods and dilution events. (Panels C and D) There have been increasing long-term trends in specific conductance in the tidal freshwater Patuxent River and tidal freshwater Anacostia River. (Panels C and D) These increasing long-term trends in specific conductance are characterized by an increase in winter pulses when road salt is applied

Salinization is also impacting tidal freshwater rivers in the northern portion of Chesapeake Bay (Fig. 12). For example, tidal freshwaters of the Patuxent River estuary are becoming more salty, whereas salty portions of the lower estuary are becoming fresher (Fig. 12A and B). When examining the longitudinal pattern of specific conductance along the Patuxent River, there is an increase in salinity pulses in tidal freshwaters due to road salts and watershed pollution and also increased pulses of fresher water in the lower estuary due to floods (Fig. 12A and B). For example, there are extreme outliers in very high specific conductance in tidal freshwater reaches coinciding with winter road salt events, and there are extreme outliers in low specific conductance in the saltier lower estuary due to floods and dilution events (Fig. 12C and D). There have been increasing trends in specific conductance in the tidal freshwater Anacostia and Patuxent Rivers over approximately the last four decades (Fig. 12C and D). These increasing long-term trends in specific conductance are characterized by an increase in strong pulses, particularly during winter months when road salt is applied (Fig. 12C and D). A future challenge will be to understand and anticipate the impacts of shifting salinity along tidal rivers and estuaries (Najjar et al. 2010; Lassiter 2021), given the growing implications for irrigation and agriculture, oyster and shellfish production, power generation, drinking water supplies, and industry within this region.

From the perspective of global implications, there could be a disappearance or displacement of tidal freshwater areas in the future due to watershed salt pollution and saltwater intrusion (Tully et al. 2019a; Little et al. 2022; Bernhardt 2022), which could influence ecosystem services, functions, and biogeochemical reactions. There are varying approaches of how a salt front can be defined in estuaries depending on geography (sensu Cook et al. 2023). In some estuaries, average saltwater intrusion and the location of the daily average of the tidal salt front is generally inversely related to river discharge (Zhang et al. 2010b), but there can be variability in these relationships with river discharge. The relationship between saltwater intrusion and river discharge varies over time, and there are interannual seasonal and annual changes in the relationships between river flow and the location of the salt front (Tian 2019; Cook et al. 2023). Variability in the location of the salt front depends on tides, winds, waves and storm surges (likely minor effects), increased precipitation, bathymetry, sea level rise, degree of mixing, upriver freshwater withdrawals for agriculture, power, and consumption (withdrawals are typically much smaller than river flows), and other factors (Najjar et al. 2010; Ralston and Geyer 2019; Tian 2019; Valle-Levinson and Li 2023). Thus, predicting the location of the salt line and saltwater intrusion is a moving target subject to environmental change and anticipating changes will be important for harnessing the many ecosystem services of tidal rivers and wetlands (Lassiter 2021, 2024; Little et al. 2022; Bernhardt 2022; Valle-Levinson and Li 2023; O’Donnell et al. 2024).

Although underappreciated, the depth and shape of the river channel (and estuary) affects potential saltwater intrusion length (Chant et al. 2011; Tian 2019; Ralston and Geyer 2019). For example, the deeper the river channel, the greater the potential for saltwater intrusion from saltier, denser, and deeper water layers (Chant et al. 2011; Ralston and Geyer 2019). Dredging for navigation significantly deepens river channels, and sea level rise can also affect the length of saltwater intrusion further up rivers (Chant et al. 2011). There are lingering questions about whether sea level rise always leads to increasing water depth along tidal rivers and estuaries (average depth may increase but there can also be spatial variations). Channel depth can mitigate or exacerbate the effects of sea level rise and depends on the sediment supply of the rivers (and whether the sediment supply is low or high) (Chant et al. 2011; Tian 2019). Sedimentation may be able to keep up with sea level rise in some estuaries but not in many others (Ensign et al. 2023), and this is certainly complicated by engineering activities such as dredging. The geomorphological response of estuaries and tidal rivers and large-scale impacts of dredging and channel engineering is largely unknown. Although more traditional emphasis is placed on sea level rise from climate change as a driver for saltwater intrusion (Najjar et al. 2010; Ross et al. 2015), other factors such as river channel dredging and sediment transport should also be considered when anticipating saltwater intrusion risks.

*Interactive risk 10: Saltwater intrusion will alter diverse ecosystem services such as habitat for aquatic life, provisioning drinking water, supporting agriculture, and power generation along tidal rivers and estuaries.*

As mentioned throughout this paper, the ecosystem scale impacts of increasing saltwater intrusion risks and biogeochemical chain reactions along tidal freshwater segments of rivers has been less studied, although they are often the link between land and sea and provide vital ecosystem services. Below, we explore impacts of salinization on different types of ecosystem functions and services along the freshwater-marine continuum.

A. Impacts along tidal rivers from organisms to ecosystems

Saltwater intrusion is altering ecosystem and biogeochemical transition zones between uplands, marshes, and open water. In initial stages of saltwater intrusion, there can be loss of uplands and gains in marsh areas with opportunities for increased carbon sequestration (e.g., Kirwan and Gedan 2019; Tully et al. 2019a, b; Guimond and Michael 2021; de la Reguera and Tully 2021). As inundation from sea level rise continues, there can be eventual loss of wetlands to open water and losses in carbon sequestration (e.g., Kirwan and Gedan 2019; Tully et al. 2019a, b; Guimond and Michael 2021; de la Reguera and Tully 2021). The frequency and extent of saltwater intrusion influences transition zones for biogeochemical reactions by altering spatial and temporal evolution of increasing salinity; decreasing dissolved oxygen; shifting groundwater salinity dynamics; changing hydraulic conductivity of soils; and causing temporary acidification and long-term alkalinization (examples of different processes are in Tully et al. 2019a, b; Kaushal et al. 2021, 2023b, 2024). All of these physical and chemical changes triggered by ‘fast’ saltwater intrusion from storms and ‘slow’ saltwater intrusion from sea level rise will influence the distribution, abundance, stability, and diversity of organisms and ecosystem services.

Along with shifting ecosystem and biogeochemical transitions, saltwater intrusion will have ripple effects from organisms to ecosystems along tidal rivers (Love et al. 2008; Osborne et al. 2015). Some organisms such as early stage amphibians and mussels are more susceptible to acute and chronic changes in salinity (Venâncio et al. 2022). There can be elevated salinity concentrations during low flow periods, when river discharge either cannot push the salt front seaward or there are increased shallow saline groundwater contributions (Sadat-Noori et al. 2015). Some forms of submerged aquatic vegetation and their organic matter are susceptible to elevated salinity (Connolly et al. 2014), and submerged aquatic vegetation can provide valuable habitat and can influence ecological impacts of hypoxia (Miranda and Hodges 2000). More work is necessary to understand and identify thresholds influencing impacts on organisms, ecosystems, and biogeochemical functions (Bachman and Rand 2008; Osborne et al. 2015). There can be losses of sensitive species at certain thresholds and changes in the geographic abundance and distribution of organisms, which represent shifts in habitat over time (Love et al. 2008; Pettit et al. 2016).

Even episodic saltwater intrusion can be problematic, with tidal rivers and marshes experiencing the most risk. Plants and macrophytes in tidal marshes are important for carbon sequestration, their organic matter inputs contribute to denitrification, and they are susceptible due to inundation (Connolly et al. 2014; Pettit et al. 2016). Plants in tidal marshes are adapted to different salinity zones influenced by microtopography and the depth and duration of inundation. For example, some plant species may be intolerant of higher salinity in marshes at lower topographic elevation near tidal rivers (Pennings et al. 2005; Pettit et al. 2016); this could result in conversion of some tidal wetlands to mudflats and altered biogeochemical reactions (Barendregt and Swarth 2013). Tidal marsh vegetation composition could also shift over time in response to salinity changes (Sutter et al. 2015), but there may be differences in fast and slow effects on vegetation dynamics (Li et al. 2022a).

Although underappreciated, salinity has the potential to influence the location, timing, and magnitude of algal blooms and harmful algal blooms (Xu et al. 2017; Li et al. 2020; McClymont et al. 2023). For example, salinity can influence transient stratification, where algal cells may be suspended in the light longer. In addition, salinity can trigger algal blooms through increased mortality of zooplankton grazers and trophic cascades in freshwaters (Hintz et al. 2017). There can be cascading effects of salinity contributing to the formation, intensity, and persistence of harmful algal blooms and interactions with warming (McClymont et al. 2023). For example, at 500 mg/L of chloride, there was a large increase in cyanobacteria concentrations whereas temperature played a smaller role (McClymont et al. 2023). This response to salinity was due to chloride reducing zooplankton biomass and richness suggesting that disturbance to the food web made conditions favorable to cyanobacteria (McClymont et al. 2023). In addition, hypersaline conditions (> 60 ppt) due to drought and loss of zooplankton and grazer communities contributed to formation of brown tides in the Laguna Madre of Texas (Buskey et al. 1997). Other factors such as nutrient availability, temperature, hydraulic flushing, and stratification are important in coastal waters, but the interactive impacts of salinity warrant further consideration in tidal ecosystems (Röthig et al. 2023).

There are many open questions regarding how changes in saltwater intrusion will impact aquatic food webs and the distribution and abundance of filter feeders and predatory fishes in tidal rivers and marshes (Romañach et al. 2019). Fish are mobile, but they may be more active and/or migrate during certain seasons or in response to saltwater intrusion with implications for altering predator–prey dynamics (Schwartz 1998; Love et al. 2008; Mohamed and Hameed 2019). Overall, direct and indirect impacts of saltwater intrusion and ecosystem transitions on fisheries and altered aquatic food webs can influence both coastal ecosystems and economies (Al-zewar and Ahmed 2020).

B. Impacts on coastal drinking water supplies

Many major drinking water supplies are located at the boundary of saltwater and freshwater interfaces (Martínez et al. 2007; Lassiter 2021, 2024). Saltwater intrusion can directly or indirectly contaminate coastal drinking water supplies, through direct salinization or indirect mobilization of chemicals by enhanced biogeochemical reactions (Moore and Joye 2021). In addition, saltwater intrusion is linked to hypertensive disorders, developmental delays in children, and other human health impacts beyond the impacts of corrosion to drinking water infrastructure (please see Kaushal et al. 2024 for an extensive review on human health implications of salinization). Even modest levels of sea level rise will have serious impacts on drinking water depending on where intakes for drinking water facilities are located and coastal water supplies may be most vulnerable (Roehl et al. 2013; Garcés-Vargas et al. 2020). Saltwater intrusion can affect multiple sources of drinking water including groundwater aquifers and surface waters in estuaries. Groundwater can also be contaminated from recharge from saltwater if wells are overpumped in coastal areas (Moore and Joye 2021; Langevin and Zygnerski 2013). It is important to emphasize that the tidal freshwater portions of estuaries are a viable drinking water source without desalinization, and tidal freshwaters are at the most risk of salinization from both watershed pollution and saltwater intrusion. Here, we consider the brackish front moving upstream into areas that were previously either tidal freshwaters or non-tidal freshwaters and contaminating viable drinking water sources.

Predicting regional impacts of saltwater intrusion on drinking water can be difficult to anticipate due to heterogeneity in local environmental factors, which include river discharge and the ability to push away the encroaching salt front, geomorphology, and channel deepening from dredging. Most previous work has focused on saltwater intrusion of aquifers and associated hydrogeologic parameters and human activities (e.g., groundwater pumping) that make them susceptible to saltwater intrusion (Klassen and Allen 2017). For an extensive review of hydrogeologic factors contributing to groundwater salinization and the biogeochemical and human health effects of groundwater salinization in coastal areas, see Kaushal et al. (2024). Much less is known regarding salinization risks along the world’s many tidal rivers. Local factors influencing the physical transport of saltwater upstream to drinking water sources include increases in channel depth, subsidence, changes in river flow, decreases in vertical mixing, increases in gravitational circulation, changes in baroclinic pressure gradients, and changes in tidal ranges, which also depend upon length and geometry (e.g., Chant et al. 2011; Ralston and Geyer 2019; Tian 2019; Cook et al. 2023; Valle-Levinson and Li 2023). Thus, there is an increasing need for customized models and decision support tools to be downscaled from global modeling assessments (e.g., Ross et al. 2021; Valle-Levinson and Li 2023) to better anticipate the effects of saltwater intrusion on drinking water sources based on local and unique conditions.

In order to better anticipate the risks of saltwater intrusion on drinking water intakes, regional models are needed to project how much salinity can migrate upstream, particularly during dry years and droughts as a research frontier (Lassiter 2021, 2024). Regional models with different local and regional boundary conditions are needed to predict how often and how long salinity thresholds are exceeded. Information regarding threshold exceedances can guide water managers on when to limit withdrawal frequency or developing advisories on whether the quality of water may present certain risks (sensu Jones and van Vliet 2018). Furthermore, regional models and plans can be developed to inform where to move intakes further into the future and identify and predict spatial regions where tidal rivers will be most affected by salinity upstream of the mouth. This information is needed to better anticipate adaptation and mitigation strategies (Lassiter 2021, 2024).

C. Saltwater intrusion impacts along tidal rivers on agriculture

During dry weather, crops demand more irrigation and streamflow is naturally lower, which increases vulnerability to saltwater intrusion risks along the freshwater-marine continuum (Tarolli et al. 2023). There may also be increasing instances when farmers cannot reliably use irrigation water from their intakes along the river because farmers either cannot use the water for irrigation or are forced to irrigate with saltier water or need to switch to growing alternative salt-tolerant crops because the water becomes too salty for conventional crop growth and health (de la Reguera et al. 2021; van Aalst et al. 2023; Mondal et al. 2023). Saltwater intrusion leads to serious economic costs (Mondal et al. 2023), and substantial losses of nitrogen and phosphorus from agricultural lands (Weissman et al. 2021). Saltwater intrusion also contributes to losses of culture and history, as homes and fields are threatened by inundation from increased flood waters from marine environments (Tully et al. 2019b).

Saltwater intrusion alters spatial and temporal transitions in biogeochemical reactions relevant to agriculture and water quality along the freshwater-marine continuum (Tully et al. 2019a; Weissman and Tully 2020). Saltwater intrusion can cause sulfate to bind with iron to form sulfides, preventing phosphorus (P) from binding to iron oxyhydroxides (*i.e.*, P stays in solution) (Tully et al. 2019a; Weissman and Tully 2020). This increases P mobilization from agricultural soils and can negatively impact coastal water quality (Tully et al. 2019a, b a,b, Weissman and Tully 2020). However, carbon can accumulate in inundated soil aggregates experiencing anoxic conditions and anaerobic metabolism (Tully et al. 2019a, b). Decreases in the SO_4_^2−^ to Cl^−^ ratio in soils and soil waters have indicated the importance of increased sulfate reduction in response to saltwater intrusion. There can be stabilization of soil organic matter through chemical sorption of organic matter onto iron and aluminum oxyhydroxides (Tully et al. 2019a, b). As the impacts of saltwater intrusion increase and soil fertility declines, there may be a need to switch to more salt tolerant crops such as barley and sorghum, and plant crops such as switchgrass for restoration and remediation of mobilized nutrients; otherwise, farmers may experience serious economic losses (de la Reguera and Tully 2021; Mondal et al. 2023).

D. Saltwater intrusion impacts on power generation, infrastructure, and cooling waters

There can be increasing operational costs of electricity generation from increasing saltwater intrusion due to sea level rise and channel dredging and deepening (Shirazi et al. 2019). The energy industry wants to draw the cleanest water possible to reduce corrosion (lower Cl^−^), scaling (lower Ca^2+^, Mg^2+^, and carbonates), and fouling and biofouling for steam generation and cooling, when generating electricity (Pan et al. 2018). However, due to environmental and ecological concerns for waste heat discharges, locations for power plants are often located along large rivers and estuaries (Lin et al. 2021). Therefore, the potential for using river water as a coolant in energy production and industry may be impaired by warmer and saltier water with lower specific heat that cools off more slowly (sensu Millero et al. 1973, Stewart et al. 2013); thermal pollution from coolants may also have further impacts if discharged back to rivers (particularly rivers already showing rising river temperatures sensu Kaushal et al. 2010, Stewart et al. 2013). Even if the slower cooling of water doesn’t pose a problem to rivers, there may be more costs required to remove the additional salts from the water actually used for cooling to prevent the precipitation of Ca(Mg)CO_3_ and CaSO_4_ and to reduce either the corrosive or scaling potential of the cooling water within the power plants.

Freshwater alkalinization and water hardness could also impact the efficiency of transmission of steam and water in piped distribution systems. Scaling from ions contributing to carbonate alkalinity and water hardness can be a major problem for water supply pipelines (Li et al. 2022b). Buildup of scale in pipes can increase the resistance of water flow and pressure within water supply pipes and can contribute to wasted energy or deterioration of pipes (Li et al. 2022b). Scale in the pipes can be enriched in toxic metals or pathogens in biofilms, which can also be a potential source of secondary contamination of drinking water supplies (Li et al. 2022b). Ultimately, both salinization and alkalinization along tidal rivers can impact the industrial uses of water and degradation of piped infrastructure.

## Future directions

Based on our predictions of a saltier and alkaline future for freshwaters, there is a growing role for ecologists, watershed hydrologists, oceanographers, landscape architects and planners, geochemists, epidemiologists, and engineers to address the future of FSS. General interdisciplinary knowledge gaps are relevant to: (1) planning—developing strategies for anticipating changes in source water quality, and then using these data to inform long-term planning; (2) technology—developing low-cost, low-energy modular technologies for removing salt from water; these facilities can be scaled up based on increasing salinity concentrations and risks; and (3) institutions—developing strategies for strong partnerships among coastal freshwater users and reallocating freshwater sources as needed to ensure basic water security. These future strategies will require communication and collaboration among diverse groups to tackle the complex problem of stopping or slowing FSS.

More work in the future is also necessary identifying all relevant stakeholders (including underrepresented groups) for managing water needs pertaining to changes in salinity, alkalinity, and pH and potential interactions with temperature from headwaters to coastal waters. There may be underrepresented groups affected by saltwater intrusion along tidal rivers and estuaries. For example, rural communities may not be a key stakeholder in terms of use, but they can be more strongly impacted and have less resources for anticipating and managing salinity risks. Understanding the risks and limitations of saline and alkaline freshwaters requires interviewing different users and decision makers for acceptable water uses across different salinities, pH, alkalinity, and salt ion concentrations (sensu Higgins et al. 2002; Dutta et al. 2022) and public perceptions (sensu Dolnicar et al. 2011). If changes in the salinity, alkalinity, and pH of water can be anticipated or predicted, it can help guide decisions regarding how to use that water better for drinking, agriculture, power generation, industry, etc*.*; for example, there may be certain recommended limits and thresholds for salinity, alkalinity, and pH of water for different uses and applications. For example, high salinity water that is very alkaline and hard is not best for steam transmission in pipes and avoiding scaling of pipes. On the other hand, high salinity water that is more acidic may affect uses of water and steam that could cause corrosion risks. There is also a need to anticipate potential complications from different salinity mixtures and chemical cocktails (Kaushal et al. 2018b, 2019, 2020), which need to be considered not only from an ecological and health perspective but also for water treatment and industrial processes (Bhide et al. 2021; Grant et al. 2022).

Predicting the future scope and magnitude of FSS along inland and coastal waters will be limited by the availability of high temporal and spatial resolution data from monitoring with sensors and high-resolution spatial monitoring (sensu Kaushal et al. 2023a, b, c, Shelton et al. 2024). High salinity or high pH events can easily be missed based on weekly or monthly sampling or limited sampling locations (Tassone et al. 2022). Further work should also focus on developing proxies with conductivity sensors, pH, and ion specific probes to expand the temporal and spatial resolution of salt ion concentrations and related chemical cocktails (Kaushal et al. 2018b, 2019, 2020, 2021; Morel et al. 2020; Galella et al. 2021). FSS will likely impact many non-tidal and tidal freshwaters around the world, which remain unmonitored due to economic or sociopolitical challenges, offering opportunities for collaborations (Krabbenhoft et al 2022).

Alternative future management scenarios, tradeoffs, and restoration strategies can be anticipated and modeled, but are typically limited by high resolution monitoring data across space and time (Sanford and Pope 2010; Dey and Prakash 2020). With improved high-resolution data, models can be developed to: (1) predict alternative scenarios for water allocations among groups, (2) develop adaptation, mitigation, and restoration strategies based on salinity, alkalinity, and pH, and (3) compare analyses of tradeoffs. Models could evaluate the potential for nature-based solutions to FSS before costly engineering solutions are constructed and implemented. Traditional engineered solutions for saltwater intrusion involve barriers, diversions, dilution from upstream reservoirs, setting new minimum flow requirements, and increasing water use efficiency (Motallebian et al. 2019). Despite potential limitations and trade-offs in salinity mitigation and attenuation associated with conservation and restoration (Kaushal et al. 2022, 2023c; Maas et al. 2023; Malin et al. 2024; Shelton et al. 2024; Long et al. 2025), nature-based solutions can also provide other ecosystem services; for example, restoration of coastal tidal wetlands may serve as future salinity barriers preventing saltwater intrusion (depending on location), while also increasing recreational opportunities and ecological habitats (White and Kaplan 2017). It is important to recognize that some of these nature-based solutions are experimental approaches and may have mixed effectiveness based on their settings (White and Kaplan 2017).

## Conclusion

Our synthesis shows that climate change, pollution, and saltwater intrusion can alter the sources, storage, reactivity, and transport of salt ions causing cascading impacts on water quality and ecosystems extending from headwaters to coastal waters. More monitoring is needed in tidal freshwaters where variability is high due to pulsed inputs from both salt pollution on land and saltwater intrusion from the sea. Understanding changes in the different compositions of salt ions, biogeochemical reactions, and mobilized chemical cocktails could provide breakthroughs for anticipating impacts of saltier and more alkaline freshwater on ecosystem services. Salinization interacts with many biogeochemical cycles and can exacerbate contemporary water quality problems as a multiple stressor along with rising temperatures and chemical cocktails from other anthropogenic sources. There will be fast and slow effects of climate change on salinity risks spreading along both ends of the freshwater-marine continuum. Our synthesis showed that salt sources, transport, and storage is changing in watersheds in response to climate change and variability. We illustrated how salinization is triggering different chain reactions with cascading impacts from headwaters to tidal waters. Finally, we showed how combined salinization from land and saltwater intrusion due to sea level rise will alter ecosystem services such as habitat for aquatic life, provisioning drinking water, supporting agriculture, and power generation along the freshwater-marine continuum.

## Supplementary Information

Below is the link to the electronic supplementary material.

Supplementary file1 (PDF 4077 KB)

Supplementary file2 (XLSX 824 KB)

## Data Availability

Many of the datasets generated during and/or analyzed during the current study are publicly available from the U.S. Geological Survey and U.S. EPA Chesapeake Bay Program. Data from individual salinization experiments are available from the corresponding author on reasonable request.

## References

[CR1] Abolfazli E, Strom K (2021) Deicing road salts may contribute to impairment of streambeds through alterations to sedimentation processes. ACS ES&T Water 2(1):148–155

[CR2] Acosta JA, Jansen B, Kalbitz K, Faz A, Martínez-Martínez S (2011) Salinity increases mobility of heavy metals in soils. Chemosphere 85(8):1318–1324 21862104 10.1016/j.chemosphere.2011.07.046

[CR3] Ahmad S (2003) Reinforcement corrosion in concrete structures, its monitoring and service life prediction––a review. Cement Concr Compos 25(4–5):459–471

[CR4] Aldea CM, Shah SP, Karr A (1999) Effect of cracking on water and chloride permeability of concrete. J Mater Civ Eng 11(3):181–187

[CR5] Almeida Júnior ES, Martínez A, Gonçalves AL, Canhoto C (2020) Combined effects of freshwater salinization and leaf traits on litter decomposition. Hydrobiologia 847:3427–3435

[CR6] Al-zewar J, Ahmed A (2020) Socio-economic impact of the saltwater intrusion in the Shatt al-Arab River on fish farmers in Al-Mashab marshes, Southern Iraq. Mediterranean Fisheries Aquaculture Res 3(2):83–91

[CR7] Amrhein C, Strong JE, Mosher PA (1992) Effect of deicing salts on metal and organic matter mobilization in roadside soils. Environ Sci Technol 26(4):703–709

[CR8] Ardón M, Morse JL, Colman BP, Bernhardt ES (2013) Drought-induced saltwater incursion leads to increased wetland nitrogen export. Glob Change Biol 19(10):2976–2985 10.1111/gcb.12287 23749653

[CR9] Ardón M, Helton AM, Bernhardt ES (2016) Drought and saltwater incursion synergistically reduce dissolved organic carbon export from coastal freshwater wetlands. Biogeochemistry 127:411–426

[CR10] Ardón M, Helton AM, Bernhardt ES (2018) Salinity effects on greenhouse gas emissions from wetland soils are contingent upon hydrologic setting: a microcosm experiment. Biogeochemistry 140:217–232

[CR11] Arslan H, Demir Y (2013) Impacts of seawater intrusion on soil salinity and alkalinity in Bafra Plain. Turkey Environ Monitor Assess 185(2):1027–1040 10.1007/s10661-012-2611-3 22488661

[CR12] Arvidsson A, Blomqvist GÖRAN, Öberg G (2012) Impact of climate change on use of anti-icing and deicing salt in Sweden. Winter maintenance and surface transportation weather: international conference on winter maintenance and surface transportation weather, vol 30. Coralville, IA, pp 3–10

[CR13] Asadieh B, Krakauer NY (2017) Global change in streamflow extremes under climate change over the 21st century. Hydrol Earth Syst Sci 21(11):5863–5874

[CR14] Bachman PM, Rand GM (2008) Effects of salinity on native estuarine fish species in South Florida. Ecotoxicology 17:591–597 18642076 10.1007/s10646-008-0244-7

[CR15] Baker ME, Schley ML, Sexton JO (2019) Impacts of expanding impervious surface on specific conductance in urbanizing streams. Water Resour Res 55(8):6482–6498

[CR16] Balling RC Jr, Gober P, Jones N (2008) Sensitivity of residential water consumption to variations in climate: an intraurban analysis of Phoenix, Arizona. Water Resour Res. 10.1029/2007WR006722

[CR17] Barco J, Gunawan S, Hogue TS (2013) Seasonal controls on stream chemical export across diverse coastal watersheds in the USA. Hydrol Process 27(10):1440–1453

[CR18] Barendregt A, Swarth CW (2013) Tidal freshwater wetlands: variation and changes. Estuaries Coasts 36(3):445–456

[CR19] Barlow PM, Reichard EG (2010) Saltwater intrusion in coastal regions of North America. Hydrogeol J 18(1):247

[CR20] Barnes RT, Raymond PA (2009) The contribution of agricultural and urban activities to inorganic carbon fluxes within temperate watersheds. Chem Geol 266(3–4):318–327

[CR21] Bastviken D, Thomsen F, Svensson T, Karlsson S, Sandén P, Shaw G, Matucha M, Öberg G (2007) Chloride retention in forest soil by microbial uptake and by natural chlorination of organic matter. Geochim Cosmochim Acta 71(13):3182–3192

[CR22] Bastviken D, Svensson T, Karlsson S, Sanden P, Oberg G (2009) Temperature sensitivity indicates that chlorination of organic matter in forest soil is primarily biotic. Environ Sci Technol 43(10):3569–3573 19544856 10.1021/es8035779

[CR23] Batanero GL, Green AJ, Amat JA, Vittecoq M, Suttle CA, Reche I (2022) Patterns of microbial abundance and heterotrophic activity along nitrogen and salinity gradients in coastal wetlands. Aquat Sci 84(2):22

[CR24] Berger E, Frör O, Schäfer RB (2019) Salinity impacts on river ecosystem processes: a critical mini-review. Philos Trans R Soc B 374(1764):20180010 10.1098/rstb.2018.0010 PMC6283965 30509912

[CR25] Bernhardt E (2022) Coastal freshwater wetlands squeezed between migrating salt marshes and working lands. Sci Adv 8(26):eadd1628 35767608 10.1126/sciadv.add1628 PMC11094673

[CR26] Bhide SV, Grant SB, Parker EA, Rippy MA, Godrej AN, Kaushal S, Prelewicz G, Saji N, Curtis S, Vikesland P, Maile-Moskowitz A (2021) Addressing the contribution of indirect potable reuse to inland freshwater salinization. Nat Sustain 4(8):699–707

[CR27] Brahney J, Ballantyne AP, Kociolek P, Spaulding S, Otu M, Porwoll T, Neff JC (2014) Dust mediated transfer of phosphorus to alpine lake ecosystems of the Wind River Range, Wyoming, USA. Biogeochemistry 120:259–278

[CR28] Brooks N (2003) Vulnerability, risk and adaptation: a conceptual framework. Tyndall Centre Clim Change Res Working Paper 38(38):1–16

[CR29] Bubeck RC, Diment WH, Deck BL, Baldwin AL, Lipton SD (1971) Runoff of deicing salt: effect on irondequoit Bay, Rochester, New Yok. Science 172(3988):1128–1132 17839819 10.1126/science.172.3988.1128

[CR30] Bui EN (2017) Causes of soil salinization, sodification, and alkalinization. In: Oxford research encyclopedia of environmental science.

[CR31] Burakowski EA, Wake CP, Braswell B, Brown DP (2008) Trends in wintertime climate in the northeastern United States: 1965–2005. J Geophys Res: Atmos. 10.1029/2008JD009870

[CR32] Buskey EJ, Montagna PA, Amos AF, Whitledge TE (1997) Disruption of grazer populations as a contributing factor to the initiation of the Texas brown tide algal bloom. Limnol Oceanogr 42:1215–1222

[CR33] Cañedo-Argüelles M, Kefford BJ, Piscart C, Prat N, Schäfer RB, Schulz CJ (2013) Salinisation of rivers: an urgent ecological issue. Environ Pollut 173:157–167 23202646 10.1016/j.envpol.2012.10.011

[CR34] Cao Z, Hu Y, Zhao H, Cao B, Zhang P (2022) Sulfate mineral scaling: from fundamental mechanisms to control strategies. Water Res 222:118945 35963137 10.1016/j.watres.2022.118945

[CR35] Chant RJ, Fugate D, Garvey E (2011) The shaping of an estuarine superfund site: roles of evolving dynamics and geomorphology. Estuaries Coasts 34:90–105

[CR36] Coble PG (1996) Characterization of marine and terrestrial DOM in seawater using excitation-emission matrix spectroscopy. Mar Chem 51(4):325–346

[CR37] Connolly CT, Sobczak WV, Findlay SE (2014) Salinity effects on Phragmites decomposition dynamics among the Hudson River’s freshwater tidal wetlands. Wetlands 34:575–582

[CR38] Cook BI, Mankin JS, Anchukaitis KJ (2018) Climate change and drought: from past to future. Curr Clim Change Rep 4:164–179

[CR39] Cook SE, Warner JC, Russell KL (2023) A numerical investigation of the mechanisms controlling salt intrusion in the Delaware Bay estuary. Estuar Coast Shelf Sci 283:108257

[CR40] Cooper CA, Mayer PM, Faulkner BR (2014) Effects of road salts on groundwater and surface water dynamics of sodium and chloride in an urban restored stream. Biogeochemistry 121:149–166

[CR41] Couturier M, Prairie YT, Paterson AM, Emilson EJS, del Giorgio PA (2022) Long-term trends in pCO2 in lake surface water following rebrowning. Geophys Res Lett 49(11):e2022GL097973

[CR42] Crawford JT, Hinckley ELS, Litaor MI, Brahney J, Neff JC (2019) Evidence for accelerated weathering and sulfate export in high alpine environments. Environ Res Lett 14(12):124092

[CR43] Cunillera-Montcusí D, Beklioğlu M, Cañedo-Argüelles M, Jeppesen E, Ptacnik R, Amorim CA, Arnott SE, Berger SA, Brucet S, Dugan HA, Gerhard M (2022) Freshwater salinisation: a research agenda for a saltier world. Trends Ecol Evol 37(5):440–453 35058082 10.1016/j.tree.2021.12.005

[CR44] D’Odorico P, Bhattachan A, Davis KF, Ravi S, Runyan CW (2013) Global desertification: drivers and feedbacks. Adv Water Resour 51:326–344

[CR45] Daley ML, Potter JD, McDowell WH (2009) Salinization of urbanizing New Hampshire streams and groundwater: effects of road salt and hydrologic variability. J N Am Benthol Soc 28(4):929–940

[CR46] Dang C, Morrissey EM, Neubauer SC, Franklin RB (2019) Novel microbial community composition and carbon biogeochemistry emerge over time following saltwater intrusion in wetlands. Glob Change Biol 25(2):549–561 10.1111/gcb.14486 30537235

[CR47] de la Reguera E, Tully KL (2021) Farming carbon: the link between saltwater intrusion and carbon storage in coastal agricultural fields. Agric Ecosyst Environ 314:107416

[CR48] Delaune KD, Nesich D, Goos JM, Relyea RA (2021) Impacts of salinization on aquatic communities: abrupt vs gradual exposures. Environ Pollut 285:117636 34380226 10.1016/j.envpol.2021.117636

[CR49] Dethier EN, Sartain SL, Renshaw CE, Magilligan FJ (2020) Spatially coherent regional changes in seasonal extreme streamflow events in the United States and Canada since 1950. Sci Adv 6(49):e5939 10.1126/sciadv.aba5939 PMC7717913 33277243

[CR50] DeVilbiss SE, Steele MK, Krometis LAH, Badgley BD (2021) Freshwater salinization increases survival of *Escherichia coli* and risk of bacterial impairment. Water Res 191:116812 33461082 10.1016/j.watres.2021.116812

[CR51] DeVilbiss SE, Badgley BD, Hotchkiss ER, Steele MK (2024) Subsidy-stress responses of ecosystem functions along experimental freshwater salinity gradients. Biogeochemistry 167:1–15

[CR52] Dey S, Prakash O (2020) Management of saltwater intrusion in coastal aquifers: an overview of recent advances. Environ Process Manage Tools Practices, 321–344

[CR53] Diment WH, Bubeck RC, Deck BL (1973) Some effects of de-icing salts on irondequoit bay and its drainage basin. Highway Res Rec 425:23–35

[CR54] Dolnicar S, Hurlimann A, Grün B (2011) What affects public acceptance of recycled and desalinated water? Water Res 45(2):933–943 20950834 10.1016/j.watres.2010.09.030 PMC3020276

[CR55] Drake TW, Tank SE, Zhulidov AV, Holmes RM, Gurtovaya T, Spencer RG (2018) Increasing alkalinity export from large Russian Arctic rivers. Environ Sci Technol 52(15):8302–8308 29947507 10.1021/acs.est.8b01051

[CR56] Duan SW, Kaushal SS (2013) Warming increases carbon and nutrient fluxes from sediments in streams across land use. Biogeosciences 10(2):1193–1207

[CR57] Duan S, Kaushal SS (2015) Salinization alters fluxes of bioreactive elements from stream ecosystems across land use. Biogeosciences 12(23):7331–7347

[CR58] Dubois KD, Lee D, Veizer J (2010) Isotopic constraints on alkalinity, dissolved organic carbon, and atmospheric carbon dioxide fluxes in the Mississippi River. J Geophys Res: Biogeosci. 10.1029/2009JG001102

[CR59] Dugan HA, Bartlett SL, Burke SM, Doubek JP, Krivak-Tetley FE, Skaff NK, Summers JC, Farrell KJ, McCullough IM, Morales-Williams AM, Roberts DC (2017) Salting our freshwater lakes. Proc Natl Acad Sci 114(17):4453–4458 28396392 10.1073/pnas.1620211114 PMC5410852

[CR60] Durack PJ, Wijffels SE, Matear RJ (2012) Ocean salinities reveal strong global water cycle intensification during 1950 to 2000. Science 336(6080):455–458 22539717 10.1126/science.1212222

[CR61] Dutta N, Thakur BK, Nurujjaman M, Debnath K, Bal DP (2022) An assessment of the water quality index (WQI) of drinking water in the Eastern Himalayas of South Sikkim. India Groundwater Sustain Deve 17:100735

[CR62] Duval C, Thomazeau S, Drelin Y, Yepremian C, Bouvy M, Couloux A, Troussellier M, Rousseau F, Bernard C (2018) Phylogeny and salt-tolerance of freshwater Nostocales strains: contribution to their systematics and evolution. Harmful Algae 73:58–71 29602507 10.1016/j.hal.2018.01.008

[CR63] Edwards M, Triantafyllidou S (2007) Chloride-to-sulfate mass ratio and lead leaching to water. J Am Water Works Assoc 99(7):96–109

[CR64] Edwards M, Schock MR, Meyer TE (1996) Alkalinity, pH, and copper corrosion by-product release. J Am Water Works Assoc 88(3):81–94

[CR65] Ensign SH, Halls JN, Peck EK (2023) Watershed sediment cannot offset sea level rise in most US tidal wetlands. Science 382(6675):1191–1195 38060655 10.1126/science.adj0513

[CR66] Entrekin SA, Clay NA, Mogilevski A, Howard-Parker B, Evans-White MA (2019) Multiple riparian–stream connections are predicted to change in response to salinization. Philos Trans R Soc B 374(1764):20180042 10.1098/rstb.2018.0042 PMC6283969 30509922

[CR67] Feng L, Zhang Z, Yang G, Wu G, Yang Q, Chen Q (2023) Microbial communities and sediment nitrogen cycle in a coastal eutrophic lake with salinity and nutrients shifted by seawater intrusion. Environ Res 225:115590 36863651 10.1016/j.envres.2023.115590

[CR68] Franklin RB, Morrissey EM, Morina JC (2017) Changes in abundance and community structure of nitrate-reducing bacteria along a salinity gradient in tidal wetlands. Pedobiologia 60:21–26

[CR69] Galella JG, Kaushal SS, Wood KL, Reimer JE, Mayer PM (2021) Sensors track mobilization of ‘chemical cocktails’ in streams impacted by road salts in the Chesapeake Bay watershed. Environ Res Lett 16(3):035017 34017359 10.1088/1748-9326/abe48f PMC8128710

[CR70] Galella JG, Kaushal SS, Mayer PM, Maas CM, Shatkay RR, Stutzke RA (2023a) Stormwater best management practices: experimental evaluation of chemical cocktails mobilized by freshwater salinization syndrome. Front Environ Sci 11:1020914 10.3389/fenvs.2023.1020914 PMC10208307 37234950

[CR71] Galella JG, Kaushal SS, Mayer PM, Maas CM, Shatkay RR, Inamdar S, Belt KT (2023b) Freshwater salinization syndrome alters nitrogen transport in urban watersheds. Water 15(22):3956 10.3390/w15223956 PMC10831318 38313692

[CR72] Garcés-Vargas J, Schneider W, Pinochet A, Piñones A, Olguin F, Brieva D, Wan Y (2020) Tidally forced saltwater intrusions might impact the quality of drinking water, the Valdivia River (40° S), Chile Estuary case. Water 12(9):2387 10.3390/w12092387 PMC9127748 35615208

[CR73] García G, Pérez J, Boyero L, Alonso A, Tuñon A, Pérez E, Cornejo A (2024) Joint effects of warming and salinization on instream leaf litter decomposition assessed through a microcosm experiment. Hydrobiologia 851:1–12

[CR74] Gardner WS, Seitzinger SP, Malczyk JM (1991) The effects of sea salts on the forms of nitrogen released from estuarine and freshwater sediments: does ion pairing affect ammonium flux? Estuaries 14:157–166

[CR75] Gardner WS, McCarthy MJ, An S, Sobolev D, Sell KS, Brock D (2006) Nitrogen fixation and dissimilatory nitrate reduction to ammonium (DNRA) support nitrogen dynamics in Texas estuaries. Limnol Oceanogr 51:558–568

[CR76] Gervasio MP, Soana E, Vincenzi F, Magri M, Castaldelli G (2023) Drought-induced salinity intrusion affects nitrogen removal in a deltaic ecosystem (Po River Delta, Northern Italy). Water 15(13):2405

[CR77] Grant SB, Rippy MA, Birkland TA, Schenk T, Rowles K, Misra S, Aminpour P, Kaushal S, Vikesland P, Berglund E, Gomez-Velez JD (2022) Can common pool resource theory catalyze stakeholder-driven solutions to the freshwater salinization syndrome? Environ Sci Technol 56(19):13517–13527 36103712 10.1021/acs.est.2c01555 PMC9536470

[CR78] Green SM, Cresser MS (2008) Nitrogen cycle disruption through the application of de-icing salts on upland highways. Water Air Soil Pollut 188:139–153

[CR79] Green SM, Machin R, Cresser MS (2008) Long-term road salting effects on dispersion of organic matter from roadside soils into drainage water. Chem Ecol 24(3):221–231

[CR80] Green SM, Machin R, Cresser MS (2009) Does road salting induce or ameliorate DOC mobilisation from roadside soils to surface waters in the long term? Environ Monit Assess 153:435–448 18566904 10.1007/s10661-008-0369-4

[CR81] Gruntz CP, Entrekin SA, Evans-White MA, Clay NA (2022) Too much of a good thing: evidence of sodium stress in an inland subtropical riparian detrital system. Appl Soil Ecol 169:104194

[CR82] Guimond JA, Michael HA (2021) Effects of marsh migration on flooding, saltwater intrusion, and crop yield in coastal agricultural land subject to storm surge inundation. Water Resour Res 57(2):e2020WR028326

[CR83] Gutchess K, Jin L, Lautz L, Shaw SB, Zhou X, Lu Z (2016) Chloride sources in urban and rural headwater catchments, central New York. Sci Total Environ 565:462–472 27183460 10.1016/j.scitotenv.2016.04.181

[CR84] Hale RL, Groffman PM (2006) Chloride effects on nitrogen dynamics in forested and suburban stream debris dams. J Environ Qual 35(6):2425–2432 17071914 10.2134/jeq2006.0164

[CR85] Hall N, Testa J, Li M, Paerl H (2023) Assessing drivers of estuarine pH: a comparative analysis of the continental USA’s two largest estuaries. Limnol Oceanogr 68(10):2227–2244

[CR86] Hansen AM, Kraus TEC, Pellerin BA, Fleck JA, Downing BD, Bergamaschi BA (2016) Optical Properties of dissolved organic matter (DOM): effects of biological and photolytic degradation. Limnol Oceanogr 61(3):1015–1032

[CR87] Haq S, Kaushal SS, Duan S (2018) Episodic salinization and freshwater salinization syndrome mobilize base cations, carbon, and nutrients to streams across urban regions. Biogeochemistry 141:463–486

[CR88] Herbert ER, Boon P, Burgin AJ, Neubauer SC, Franklin RB, Ardón M, Hopfensperger KN, Lamers LP, Gell P (2015) A global perspective on wetland salinization: ecological consequences of a growing threat to freshwater wetlands. Ecosphere 6(10):1–43

[CR89] Higgins J, Warnken J, Sherman PP, Teasdale PR (2002) Survey of users and providers of recycled water: quality concerns and directions for applied research. Water Res 36(20):5045–5056 12448553 10.1016/s0043-1354(02)00158-6

[CR90] Hinson KE, Friedrichs MA, St-Laurent P, Da F, Najjar RG (2022) Extent and causes of Chesapeake Bay warming. J Am Water Resour Assoc 58(6):805–825

[CR91] Hintz WD, Relyea RA (2019) A review of the species, community, and ecosystem impacts of road salt salinisation in fresh waters. Freshw Biol 64(6):1081–1097

[CR92] Hintz WD, Mattes BM, Schuler MS, Jones DK, Stoler AB, Lind L, Relyea RA (2017) Salinization triggers a trophic cascade in experimental freshwater communities with varying food-chain length. Ecol Appl 27(3):833–844 27992971 10.1002/eap.1487

[CR93] Howard KWF, Haynes J (1993) Groundwater contamination due to road de-icing chemicals—salt balance implications. *Geoscience Canada*, 20(1). https://journals.lib.unb.ca/index.php/GC/article/view/3784. Accessed 24 July 2024

[CR94] Hruska J, Krám P, McDowell WH, Oulehle F (2009) Increased dissolved organic carbon (DOC) in Central European streams is driven by reductions in ionic strength rather than climate change or decreasing acidity. Environ Sci Technol 43(12):4320–4326 19603641 10.1021/es803645w

[CR95] Huang Y, Song B, Zhang Q, Park Y, Wilson SJ, Tobias CR, An S (2024) Seawater intrusion effects on nitrogen cycling in the regulated Nakdong River Estuary, South Korea. Front Marine Sci 11:1369421

[CR96] Huguet A, Vacher L, Relexans S, Saubusse S, Froidefond JM, Parlanti E (2009) Properties of fluorescent dissolved organic matter in the Gironde Estuary. Org Geochem 40(6):706–719

[CR97] Huling EE, Hollocher TC (1972) Groundwater contamination by road salt: steady-state concentrations in east central Massachusetts. Science 176:288–290 5019780 10.1126/science.176.4032.288

[CR98] Huq A, West PA, Small EB, Huq MI, Colwell RR (1984) Influence of water temperature, salinity, and pH on survival and growth of toxigenic Vibrio cholerae serovar 01 associated with live copepods in laboratory microcosms. Appl Environ Microbiol 48(2):420–424 6486784 10.1128/aem.48.2.420-424.1984 PMC241529

[CR99] Hussein AH, Rabenhorst MC (2001) Tidal inundation of transgressive coastal areas: pedogenesis of salinization and alkalinization. Soil Sci Soc Am J 65(2):536–544

[CR100] Hyde AM, Zultanski SL, Waldman JH, Zhong Y-L, Shevlin M, Peng F (2017) General principles and strategies for salting-out informed by the Hofmeister series. Org Process Res Dev 21:1355–1370

[CR101] Inamdar S, Peipoch M, Sena M, Joshi B, Rahman MM, Kan J, Peck EK, Gold A, Trammell TL, Groffman PM (2024) Riparian groundwater nitrogen (N) isotopes reveal human imprints of dams and road salt salinization. Geophys Res Lett 51(5):e2023GL106888

[CR102] Jeppesen E, Beklioğlu M, Özkan K, Akyürek Z (2020) Salinization increase due to climate change will have substantial negative effects on inland waters: a call for multifaceted research at the local and global scale. Innovation 1(2):100030 34557708 10.1016/j.xinn.2020.100030 PMC8454634

[CR103] Jiang Y, Yin G, Li Y, Hou L, Liu M, Chen C, Zheng D, Wu H, Gao D, Zheng Y, Han P (2023) Saltwater incursion regulates N2O emission pathways and potential nitrification and denitrification in intertidal wetland. Biol Fertil Soils 59(5):541–553

[CR104] Jones E, van Vliet MT (2018) Drought impacts on river salinity in the southern US: Implications for water scarcity. Sci Total Environ 644:844–853 30743882 10.1016/j.scitotenv.2018.06.373

[CR105] Kaushal SS (2016) Increased salinization decreases safe drinking water. Environ Sci Technol 50(6):2765–2766 26903048 10.1021/acs.est.6b00679

[CR106] Kaushal SS, Groffman PM, Likens GE, Belt KT, Stack WP, Kelly VR, Band LE, Fisher GT (2005) Increased salinization of fresh water in the northeastern United States. Proc Natl Acad Sci 102(38):13517–13520 16157871 10.1073/pnas.0506414102 PMC1224654

[CR107] Kaushal SS, Groffman PM, Band LE, Shields CA, Morgan RP, Palmer MA, Belt KT, Swan CM, Findlay SE, Fisher GT (2008) Interaction between urbanization and climate variability amplifies watershed nitrate export in Maryland. Environ Sci Technol 42(16):5872–5878 18767638 10.1021/es800264f

[CR108] Kaushal SS, Likens GE, Jaworski NA, Pace ML, Sides AM, Seekell D, Belt KT, Secor DH, Wingate RL (2010) Rising stream and river temperatures in the United States. Front Ecol Environ 8(9):461–466

[CR109] Kaushal SS, Likens GE, Utz RM, Pace ML, Grese M, Yepsen M (2013) Increased river alkalinization in the Eastern US. Environ Sci Technol 47(18):10302–10311 23883395 10.1021/es401046s

[CR110] Kaushal SS, Mayer PM, Vidon PG, Smith RM, Pennino MJ, Newcomer TA, Duan S, Welty C, Belt KT (2014) Land use and climate variability amplify carbon, nutrient, and contaminant pulses: a review with management implications. J Am Water Resour Assoc 50(3):585–614

[CR111] Kaushal SS, Duan S, Doody TR, Haq S, Smith RM, Johnson TAN, Newcomb KD, Gorman J, Bowman N, Mayer PM, Wood KL (2017) Human-accelerated weathering increases salinization, major ions, and alkalinization in fresh water across land use. Appl Geochem 83:121–135 30220785 10.1016/j.apgeochem.2017.02.006 PMC6134868

[CR112] Kaushal SS, Likens GE, Pace ML, Utz RM, Haq S, Gorman J, Grese M (2018a) Freshwater salinization syndrome on a continental scale. Proc Natl Acad Sci 115(4):E574–E583 29311318 10.1073/pnas.1711234115 PMC5789913

[CR113] Kaushal SS, Gold AJ, Bernal S, Johnson TAN, Addy K, Burgin A, Burns DA, Coble AA, Hood E, Lu Y, Mayer P (2018b) Watershed ‘chemical cocktails’: forming novel elemental combinations in Anthropocene fresh waters. Biogeochemistry 141:281–305 31427837 10.1007/s10533-018-0502-6 PMC6699637

[CR114] Kaushal SS, Likens GE, Pace ML, Haq S, Wood KL, Galella JG, Morel C, Doody TR, Wessel B, Kortelainen P, Räike A (2019) Novel ‘chemical cocktails’ in inland waters are a consequence of the freshwater salinization syndrome. Philos Trans R Soc B 374(1764):20180017 10.1098/rstb.2018.0017 PMC6283973 30509916

[CR115] Kaushal SS, Wood KL, Galella JG, Gion AM, Haq S, Goodling PJ, Haviland KA, Reimer JE, Morel CJ, Wessel B, Nguyen W (2020) Making ‘chemical cocktails’—evolution of urban geochemical processes across the periodic table of elements. Appl Geochem 119:104632 10.1016/j.apgeochem.2020.104632 PMC7970522 33746355

[CR116] Kaushal SS, Likens GE, Pace ML, Reimer JE, Maas CM, Galella JG, Utz RM, Duan S, Kryger JR, Yaculak AM, Boger WL (2021) Freshwater salinization syndrome: from emerging global problem to managing risks. Biogeochemistry 154:255–292

[CR117] Kaushal SS, Reimer JE, Mayer PM, Shatkay RR, Maas CM, Nguyen WD, Boger WL, Yaculak AM, Doody TR, Pennino MJ, Bailey NW (2022) Freshwater salinization syndrome alters retention and release of chemical cocktails along flowpaths: from stormwater management to urban streams. Freshwater Sci 41(3):420–441 10.1086/721469 PMC9533665 36213200

[CR118] Kaushal SS, Likens GE, Mayer PM, Shatkay RR, Shelton SA, Grant SB, Utz RM, Yaculak AM, Maas CM, Reimer JE, Bhide SV (2023a) The anthropogenic salt cycle. Nat Rev Earth Environ 4(11):770–784 38515734 10.1038/s43017-023-00485-y PMC10953805

[CR119] Kaushal SS, Mayer PM, Likens GE, Reimer JE, Maas CM, Rippy MA, Grant SB, Hart I, Utz RM, Shatkay RR, Wessel BM (2023b) Five state factors control progressive stages of freshwater salinization syndrome. Limnol Oceanogr Lett 8(1):190–211 37539375 10.1002/lol2.10248 PMC10395323

[CR120] Kaushal SS, Maas CM, Mayer PM, Newcomer-Johnson TA, Grant SB, Rippy MA, Shatkay RR, Leathers J, Gold AJ, Smith C, McMullen EC (2023c) Longitudinal stream synoptic monitoring tracks chemicals along watershed continuums: a typology of trends. Front Environ Sci 11:1122485 10.3389/fenvs.2023.1122485 PMC10355011 37475839

[CR121] Kaushal SS, Mayer PM, Shatkey RR, Maas CM, Cañedo-Argüelles M, Hintz WD, Wessel BM, Tully KG, Rippy MA, Grant SB (2024) Salinization of inland waters, 3rd edition of Treatise on Geochemistry. Elsevier

[CR122] Kelly VR, Lovett GM, Weathers KC, Findlay SE, Strayer DL, Burns DJ, Likens GE (2008) Long-term sodium chloride retention in a rural watershed: legacy effects of road salt on streamwater concentration. Environ Sci Technol 42(2):410–415 18284139 10.1021/es071391l

[CR123] Khalkhali M, Mo W (2020) The energy implication of climate change on urban wastewater systems. J Clean Prod 267:121905

[CR124] Kim YS, Lim HK, Kim JJ, Hwang WS, Park YS (2011) Corrosion cost and corrosion map of Korea-based on the data from 2005 to 2010. Corros Sci Technol 10(2):52–59

[CR125] Kinsman-Costello L, Bean E, Goeckner A, Matthews JW, O’Driscoll M, Palta MM, Peralta AL, Reisinger AJ, Reyes GJ, Smyth AR, Stofan M (2023) Mud in the city: effects of freshwater salinization on inland urban wetland nitrogen and phosphorus availability and export. Limnol Oceanogr Lett 8(1):112–130

[CR126] Kirwan ML, Gedan KB (2019) Sea-level driven land conversion and the formation of ghost forests. Nat Clim Chang 9(6):450–457

[CR127] Klassen J, Allen DM (2017) Assessing the risk of saltwater intrusion in coastal aquifers. J Hydrol 551:730–745

[CR128] Koch GH, Brongers MP, Thompson NG, Virmani YP, Payer JH (2005) Cost of corrosion in the United States. Handbook of environmental degradation of materials. William Andrew Publishing, pp 3–24

[CR129] Kocin PJ, Uccellini L (2016) Snowstorms along the Northeastern Coast of the United States: 1955 to 1985. Springer

[CR130] Kopáček J, Hejzlar J, Kaňa J, Porcal P, Klementová Š (2003) Photochemical, chemical, and biological transformations of dissolved organic carbon and its effect on alkalinity production in acidified lakes Ji. Limnol Oceanogr 48(1):106–117

[CR131] Kopáček J, Hejzlar J, Porcal P, Posch M (2014a) A mass-balance study on chloride fluxes in a large central European catchment during 1900–2010. Biogeochemistry 120:319–335

[CR132] Kopáček J, Hejzlar J, Porcal P, Posch M (2014b) Sulphate leaching from diffuse agricultural and forest sources in a large central European catchment during 1900–2010. Sci Total Environ 470:543–550 24176702 10.1016/j.scitotenv.2013.10.013

[CR133] Kopáček J, Kana J, Bičárová S, Fernandez IJ, Hejzlar J, Kahounová M, Norton SA, Stuchlik E (2017a) Climate change increasing calcium and magnesium leaching from granitic alpine catchments. Environ Sci Technol 51(1):159–166 27997122 10.1021/acs.est.6b03575

[CR134] Kopáček J, Hejzlar J, Porcal P, Posch M (2017b) Trends in riverine element fluxes: a chronicle of regional socio-economic changes. Water Res 125:374–383 28881213 10.1016/j.watres.2017.08.067

[CR135] Krabbenhoft CA, Allen GH, Lin P, Godsey SE, Allen DC, Burrows RM et al (2022) Assessing placement bias of the global river gauge network. Nat Sustain 5(7):586–592 36213515 10.1038/s41893-022-00873-0 PMC9534037

[CR136] Kunkle SH (1972) Effects of road salt on a Vermont stream. J Am Water Works Ass 64(5):290–295

[CR137] Laceby JP, Kerr JG, Zhu D, Chung C, Situ Q, Abbasi S, Orwin JF (2019) Chloride inputs to the North Saskatchewan River watershed: the role of road salts as a potential driver of salinization downstream of North America’s northern most major city (Edmonton, Canada). Sci Total Environ 688:1056–1068 31726537 10.1016/j.scitotenv.2019.06.208

[CR138] Langevin CD, Zygnerski M (2013) Effect of sea-level rise on salt water intrusion near a coastal well field in Southeastern Florida. Groundwater 51(5):781–803 10.1111/j.1745-6584.2012.01008.x 23145832

[CR139] Lassiter A (2021) Rising seas, changing salt lines, and drinking water salinization. Curr Opin Environ Sustain 50:208–214

[CR140] Lassiter A (2024) Planning for drinking water salinization in the US Atlantic and Gulf Coast regions. J Am Plan Assoc 90:1–16

[CR141] Lazur A, VanDerwerker T, Koepenick K (2020) Review of implications of road salt use on groundwater quality—corrosivity and mobilization of heavy metals and radionuclides. Water Air Soil Pollut 231(9):474

[CR142] Li M, Ni W, Zhang F, Glibert PM, Lin CHM (2020) Climate-induced interannual variability and projected change of two harmful algal bloom taxa in Chesapeake Bay, USA. Sci Total Environ 744:140947 32721680 10.1016/j.scitotenv.2020.140947

[CR143] Li F, Angelini C, Byers JE, Craft C, Pennings SC (2022a) Responses of a tidal freshwater marsh plant community to chronic and pulsed saline intrusion. J Ecol 110(7):1508–1524

[CR144] Li CG, Liu C, Xu WH, Shan MG, Wu HX (2022b) Formation mechanisms and supervisory prediction of scaling in water supply pipelines: a review. Water Res 222:118922 35932708 10.1016/j.watres.2022.118922

[CR145] Li L, Knapp JL, Lintern A, Ng GHC, Perdrial J, Sullivan PL, Zhi W (2024a) River water quality shaped by land–river connectivity in a changing climate. Nat Clim Change 14:1–13

[CR146] Li D, Liu B, Lu Y, Fu J (2024b) The characteristic of compound drought and saltwater intrusion events in the several major river estuaries worldwide. J Environ Manage 350:119659 38029500 10.1016/j.jenvman.2023.119659

[CR147] Likens GE, Bormann FH, Johnson NM, Pierce RS (1967) The calcium, magnesium, potassium, and sodium budgets for a small forested ecosystem. Ecology 48(5):772–785 34493004 10.2307/1933735

[CR148] Likens GE, Driscoll CT, Buso DC (1996) Long-term effects of acid rain: response and recovery of a forest ecosystem. Science 272(5259):244–246

[CR149] Lin J, Zou X, Huang F (2021) Quantitative analysis of the factors influencing the dispersion of thermal pollution caused by coastal power plants. Water Res 1(188):116558 10.1016/j.watres.2020.116558 33157473

[CR151] Lintern A, Liu S, Minaudo C, Dupas R, Guo D, Zhang K, Bende-Michl U, Duvert C (2021) The influence of climate on water chemistry states and dynamics in rivers across Australia. Hydrol Process 35(12):e14423

[CR152] Lintern A, Kho N, Peterson T, Guo D (2023) Shifts in stream salt loads during and after prolonged droughts. Hydrol Process 37(6):e14901

[CR153] Little S, Lewis JP, Pietkiewicz H (2022) Defining estuarine squeeze: the loss of upper estuarine transitional zones against in-channel barriers through saline intrusion. Estuar Coast Shelf Sci 278:108107

[CR154] Long S, Rippy MA, Krauss L, Stacey M, Fausey K (2025) The impact of deicer and anti-icer use on plant communities in stormwater detention basins: characterizing salt stress and phytoremediation potential. Sci Total Environ. 10.1016/j.scitotenv.2024.178310 10.1016/j.scitotenv.2024.178310 39818486

[CR155] Lopez KG, Xiao J, Crockett C, Lytle C, Grubbs H, Edwards M (2022) Seasonal fluctuations in nitrate levels can trigger lead solder corrosion problems in drinking water. Environ Sci Technol Lett 10(1):21–26 36643386 10.1021/acs.estlett.2c00581 PMC9835880

[CR156] Love JW, Gill J, Newhard JJ (2008) Saltwater intrusion impacts fish diversity and distribution in the Blackwater River drainage (Chesapeake Bay watershed). Wetlands 28:967–974

[CR157] Luo M, Huang JF, Zhu WF, Tong C (2019) Impacts of increasing salinity and inundation on rates and pathways of organic carbon mineralization in tidal wetlands: a review. Hydrobiologia 827:31–49

[CR158] Maas CM, Kaushal SS, Rippy MA, Mayer PM, Grant SB, Shatkay RR, Malin JT, Bhide SV, Vikesland P, Krauss L, Reimer JE (2023) Freshwater salinization syndrome limits management efforts to improve water quality. Front Environ Sci 11:1106581 10.3389/fenvs.2023.1106581 PMC10568995 37841559

[CR159] MacAdam J, Jarvis P (2015) Water-formed scales and deposits: types, characteristics, and relevant industries. Mineral scales and deposits. Elsevier, pp 3–23

[CR160] MacLeod A, Sibert R, Snyder C, Koretsky CM (2011) Eutrophication and salinization of urban and rural kettle lakes in Kalamazoo and Barry Counties, Michigan, USA. Appl Geochem 26:S214–S217

[CR161] Malin JT, Kaushal SS, Mayer PM, Maas CM, Hohman SP, Rippy MA (2024) Longitudinal stream synoptic (LSS) monitoring to evaluate water quality in restored streams. Environ Monit Assess 196(5):437 38592553 10.1007/s10661-024-12570-w PMC11069387

[CR162] Martínez ML, Intralawan A, Vázquez G, Pérez-Maqueo O, Sutton P, Landgrave R (2007) The coasts of our world: ecological, economic and social importance. Ecol Econ 63(2–3):254–272

[CR163] Martínez A, Barros J, Gonçalves AL, Canhoto C (2020) Salinisation effects on leaf litter decomposition in fresh waters: Does the ionic composition of salt matter? Freshw Biol 65(8):1475–1483

[CR164] Matthews L, Andrey J, Picketts I (2017) Planning for winter road maintenance in the context of climate change. Weather, Climate, and Society 9(3):521–532

[CR165] McClymont A, Arnott SE, Rusak JA (2023) Interactive effects of increasing chloride concentration and warming on freshwater plankton communities. Limnol Oceanogr Lett 8(1):56–64

[CR166] McNaboe LA, Robbins GA, Dietz ME (2017) Mobilization of radium and radon by deicing salt contamination of groundwater. Water Air Soil Pollut 228:1–929225380

[CR167] Meriano M, Eyles N, Howard KWF (2009) Hydrogeological impacts of road salt from Canada’s busiest highway on a Lake Ontario watershed (Frenchman’s Bay) and lagoon, City of Pickering. J Contam Hydrol 107:66–81 19464750 10.1016/j.jconhyd.2009.04.002

[CR168] Michael HA, Mulligan AE, Harvey CF (2005) Seasonal oscillations in water exchange between aquifers and the coastal ocean. Nature 436(7054):1145–1148 16121178 10.1038/nature03935

[CR169] Michael HA, Post VE, Wilson AM, Werner AD (2017) Science, society, and the coastal groundwater squeeze. Water Resour Res 53(4):2610–2617

[CR170] Millero FJ, Perron G, Desnoyers JE (1973) Heat capacity of seawater solutions from 5 to 35 C and 0.5 to 22‰ chlorinity. J Geophys Res 78(21):4499–4507

[CR171] Minor EC, Pothen J, Dalzell BJ, Abdulla H, Mopper K (2006) Effects of salinity changes on the photodegradation and ultraviolet—visible absorbance of terrestrial dissolved organic matter. Limnol Oceanogr 51(5):2181–2186

[CR172] Miranda LE, Hodges KB (2000) Role of aquatic vegetation coverage on hypoxia and sunfish abundance in bays of a eutrophic reservoir. Hydrobiologia 427(1):51–57

[CR173] Mohamed ARM, Hameed EK (2019) Impacts of saltwater intrusion on the fish assemblage in the middle part of Shatt Al-Arab River, Iraq. Asian J Appl Sci. 10.24203/ajas.v7i5.5917

[CR174] Mondal P, Walter M, Miller J, Epanchin-Niell R, Gedan K, Yawatkar V, Nguyen E, Tully KL (2023) The spread and cost of saltwater intrusion in the US Mid-Atlantic. Nat Sustain 6(11):1352–1362

[CR175] Moore WS, Joye SB (2021) Saltwater intrusion and submarine groundwater discharge: acceleration of biogeochemical reactions in changing coastal aquifers. Front Earth Sci 9:600710

[CR176] Moore J, Bird DL, Dobbis SK, Woodward G (2017) Nonpoint source contributions drive elevated major ion and dissolved inorganic carbon concentrations in urban watersheds. Environ Sci Technol Lett 4(6):198–204

[CR177] Morel CJ, Kaushal SS, Tan ML, Belt KT (2020) Developing sensor proxies for “chemical cocktails” of trace metals in urban streams. Water 12(10):2864

[CR178] Motallebian M, Ahmadi H, Raoof A, Cartwright N (2019) An alternative approach to control saltwater intrusion in coastal aquifers using a freshwater surface recharge canal. J Contam Hydrol 222:56–64 30837160 10.1016/j.jconhyd.2019.02.007

[CR179] Müller B, Gächter R (2012) Increasing chloride concentrations in Lake Constance: characterization of sources and estimation of loads. Aquat Sci 74:101–112

[CR180] Müller JD, Schneider B, Rehder G (2016) Long-term alkalinity trends in the Baltic Sea and their implications for CO_2_-induced acidification. Limnol Oceanogr 61(6):1984–2002

[CR181] Murphy J, Sprague L (2019) Water-quality trends in US rivers: exploring effects from streamflow trends and changes in watershed management. Sci Total Environ 656:645–658 30529968 10.1016/j.scitotenv.2018.11.255

[CR182] Najjar RG, Pyke CR, Adams MB, Breitburg D, Hershner C, Kemp M, Howarth R, Mulholland MR, Paolisso M, Secor D, Sellner K (2010) Potential climate-change impacts on the Chesapeake Bay. Estuar Coast Shelf Sci 86(1):1–20

[CR183] Najjar RG, Herrmann M, Cintrón Del Valle SM, Friedman JR, Friedrichs MA, Harris LA, Shadwick EH, Stets EG, Woodland RJ (2020) Alkalinity in tidal tributaries of the Chesapeake Bay. J Geophys Res: Oceans 125(1):e2019JC015597 10.1029/2019JC015610 PMC7380316 32728507

[CR184] Navarro DA, Oliver DP, Simpson SL, Kookana RS (2022) Organic carbon and salinity affect desorption of PFAS from estuarine sediments. J Soils Sediments 22(4):1302–1314

[CR185] Naz BS, Kao SC, Ashfaq M, Gao H, Rastogi D, Gangrade S (2018) Effects of climate change on streamflow extremes and implications for reservoir inflow in the United States. J Hydrol 556:359–370

[CR186] Neubauer SC, Franklin RB, Berrier DJ (2013) Saltwater intrusion into tidal freshwater marshes alters the biogeochemical processing of organic carbon. Biogeosciences 10(12):8171–8183

[CR187] Neubauer SC, Piehler MF, Smyth AR, Franklin RB (2019) Saltwater intrusion modifies microbial community structure and decreases denitrification in tidal freshwater marshes. Ecosystems 22:912–928

[CR188] Novotny EV, Sander AR, Mohseni O, Stefan HG (2009) Chloride ion transport and mass balance in a metropolitan area using road salt. *Water Resour Res*, *45*(12).

[CR189] Novotny EV, Stefan HG (2010) Projections of chloride concentrations in urban lakes receiving road de-icing salt. Water Air Soil Pollut 211:261–271

[CR190] O’Donnell KL, Bernhardt ES, Yang X, Emanuel RE, Ardón M, Lerdau MT, Manda AK, Braswell AE, BenDor TK, Edwards EC, Frankenberg E (2024) Saltwater intrusion and sea level rise threatens US rural coastal landscapes and communities. Anthropocene, 100427

[CR191] Oliveira R, Martínez A, Gonçalves AL, Júnior ESA, Canhoto C (2021) Salt pulses effects on in-stream litter processing and recovery capacity depend on substrata quality. Sci Total Environ 783:147013 33872895 10.1016/j.scitotenv.2021.147013

[CR192] Osborne RI, Bernot MJ, Findlay SE (2015) Changes in nitrogen cycling processes along a salinity gradient in tidal wetlands of the Hudson River, New York, USA. Wetlands 35:323–334

[CR193] Osburn FS, Wagner ND, Taylor RB, Chambliss CK, Brooks BW, Scott JT (2023) The effects of salinity and N: P on N-rich toxins by both an N-fixing and non-N-fixing cyanobacteria. Limnol Oceanogr Lett 8(1):162–172 36777312 10.1002/lol2.10234 PMC9915339

[CR194] Oswald CJ, Giberson G, Nicholls E, Wellen C, Oni S (2019) Spatial distribution and extent of urban land cover control watershed-scale chloride retention. Sci Total Environ 652:278–288 30366328 10.1016/j.scitotenv.2018.10.242

[CR195] Pan SY, Snyder SW, Packman AI, Lin YJ, Chiang PC (2018) Cooling water use in thermoelectric power generation and its associated challenges for addressing water-energy nexus. Water-Energy Nexus 1(1):26–41

[CR196] Pennings SC, Grant MB, Bertness MD (2005) Plant zonation in low-latitude salt marshes: disentangling the roles of flooding, salinity and competition. J Ecol: 159–167

[CR197] Perera N, Gharabaghi B, Howard K (2013) Groundwater chloride response in the Highland Creek watershed due to road salt application: a re-assessment after 20 years. J Hydrol 479:159–168

[CR198] Perri S, Suweis S, Holmes A, Marpu PR, Entekhabi D, Molini A (2020) River basin salinization as a form of aridity. Proc Natl Acad Sci 117(30):17635–17642 32651272 10.1073/pnas.2005925117 PMC7395538

[CR199] Pettit NE, Bayliss P, Bartolo R (2016) Dynamics of plant communities and the impact of saltwater intrusion on the floodplains of Kakadu National Park. Mar Freshw Res 69(7):1124–1133

[CR200] Pieper KJ, Tang M, Edwards MA (2017) Flint water crisis caused by interrupted corrosion control: investigating “ground zero” home. Environ Sci Technol 51(4):2007–2014 28145123 10.1021/acs.est.6b04034

[CR201] Pieper KJ, Martin R, Tang M, Walters L, Parks J, Roy S, Devine C, Edwards MA (2018) Evaluating water lead levels during the Flint water crisis. Environ Sci Technol 52(15):8124–8132 29932326 10.1021/acs.est.8b00791

[CR202] Pucher M, Wünsch U, Weigelhofer G, Murphy K, Hein T, Graeber D (2019) staRdom: versatile software for analyzing spectroscopic data of dissolved organic matter in R. Water 11(11):2366

[CR203] Qiu GY, Zou Z, Li X, Li H, Guo Q, Yan C, Tan S (2017) Experimental studies on the effects of green space and evapotranspiration on urban heat island in a subtropical megacity in China. Habitat Int 68:30–42

[CR204] Radosavljevic J, Slowinski S, Shafii M, Akbarzadeh Z, Rezanezhad F, Parsons CT, Withers W, Van Cappellen P (2022) Salinization as a driver of eutrophication symptoms in an urban lake (Lake Wilcox, Ontario, Canada). Sci Total Environ 846:157336 35863566 10.1016/j.scitotenv.2022.157336

[CR205] Ralston DK, Geyer WR (2019) Response to channel deepening of the salinity intrusion, estuarine circulation, and stratification in an urbanized estuary. J Geophys Res: Oceans 124(7):4784–4802

[CR206] Raymond PA (2017) Temperature versus hydrologic controls of chemical weathering fluxes from United States forests. Chem Geol 458:1–13

[CR207] Raymond PA, Oh NH, Turner RE, Broussard W (2008) Anthropogenically enhanced fluxes of water and carbon from the Mississippi River. Nature 451(7177):449–452 18216851 10.1038/nature06505

[CR208] Rice KC, Moyer DL, Mills AL (2017) Riverine discharges to Chesapeake Bay: analysis of long-term (1927–2014) records and implications for future flows in the Chesapeake Bay basin. J Environ Manage 204:246–254 28888206 10.1016/j.jenvman.2017.08.057

[CR209] Rice KC, Hirsch RM (2012) Spatial and temporal trends in runoff at long-term streamgages within and near the Chesapeake Bay watershed (No. 2012–5151). US Geological Survey.

[CR210] Rippy MA, Roston BH, Berglund EZ, Aminpour P, Krauss L, Bhide SV, Schenk T, Rowles K, Misra S, Birkland TA, Kaushal SS (2024) Characterizing the social-ecological system for inland freshwater salinization using fuzzy cognitive maps: implications for collective management. Ecol Soc 29(4).

[CR211] Rodríguez-Liébana JA, Mingorance MD, Peña A (2010) Sorption of hydrophobic pesticides on a Mediterranean soil affected by wastewater, dissolved organic matter and salts. J Environ Manage 92(3):650–654 20980092 10.1016/j.jenvman.2010.10.009

[CR212] Roehl EA Jr, Daamen RC, Cook JB (2013) Estimating seawater intrusion impacts on coastal intakes as a result of climate change. J Am Water Works Assoc 105(11):E642–E650

[CR213] Romañach SS, Beerens JM, Patton BA, Chapman JP, Hanson MR (2019) Impacts of saltwater intrusion on wetland prey production and composition in a historically freshwater marsh. Estuaries Coasts 42(6):1600–1611

[CR214] Ross AC, Najjar RG, Li M, Mann ME, Ford SE, Katz B (2015) Sea-level rise and other influences on decadal-scale salinity variability in a coastal plain estuary. Estuar Coast Shelf Sci 157:79–92

[CR215] Ross AC, Najjar RG, Li M (2021) A metamodel-based analysis of the sensitivity and uncertainty of the response of Chesapeake Bay salinity and circulation to projected climate change. Estuaries Coasts 44:70–87

[CR216] Rossi ML, Kremer P, Cravotta CA, Scheirer KE, Goldsmith ST (2022) Long-term impacts of impervious surface cover change and roadway deicing agent application on chloride concentrations in exurban and suburban watersheds. Sci Total Environ 851:157933 35987233 10.1016/j.scitotenv.2022.157933

[CR217] Röthig T, Trevathan-Tackett SM, Voolstra CR, Ross C, Chaffron S, Durack PJ, Warmuth LM, Sweet M (2023) Human-induced salinity changes impact marine organisms and ecosystems. Glob Change Biol 29(17):4731–4749 10.1111/gcb.16859 37435759

[CR218] Ruth O (2003) The effects of de-icing in Helsinki urban streams, Southern Finland. Water Sci Technol 48:33–43 14703137

[CR219] Rütting T, Boeckx P, Müller C, Klemedtsson L (2011) Assessment of the importance of dissimilatory nitrate reduction to ammonium for the terrestrial nitrogen cycle. Biogeosciences 8(7):1779–1791

[CR220] Sadat-Noori M, Santos IR, Sanders CJ, Sanders LM, Maher DT (2015) Groundwater discharge into an estuary using spatially distributed radon time series and radium isotopes. J Hydrol 528:703–719

[CR221] Salcedo AJM, Prat N, Bertrans-Tubau L, Piñero-Fernandez M, Cunillera-Montcusí D, López-Doval JC, Abril M, Proia L, Cañedo-Argüelles M (2024) What happens when salinization meets eutrophication? A test using stream microcosms. Sci Total Environ 912:168824 38030007 10.1016/j.scitotenv.2023.168824

[CR222] Sanford WE, Pope JP (2010) Current challenges using models to forecast seawater intrusion: lessons from the Eastern Shore of Virginia, USA. Hydrogeol J 18(1):73

[CR223] Schafer T, Powers L, Gonsior M, Reddy KR, Osborne TZ (2021) Contrasting responses of DOM leachates to photodegradation observed in plant species collected along an estuarine salinity gradient. Biogeochemistry 152:291–307

[CR224] Schwartz FJ (1998) Fishes affected by freshwater inflows and/or marine intrusions in North Carolina. J Elisha Mitchell Sci Soc: 173–189

[CR225] Seo DC, Yu K, Delaune RD (2008) Influence of salinity level on sediment denitrification in a Louisiana estuary receiving diverted Mississippi River water. Arch Agron Soil Sci 54(3):249–257

[CR226] Seybold EC, Dwivedi R, Musselman KN, Kincaid DW, Schroth AW, Classen AT, Perdrial JN, Adair EC (2022) Winter runoff events pose an unquantified continental-scale risk of high wintertime nutrient export. Environ Res Lett 17(10):104044

[CR227] Shanley JB (1994) Effects of ion exchange on stream solute fluxes in a basin receiving highway deicing salts. J Environ Qual 23(5):977–986 34872214 10.2134/jeq1994.00472425002300050019x

[CR228] Sharqawy MH, Lienhard JH, Zubair SM (2010) Thermophysical properties of seawater: a review of existing correlations and data. Desalin Water Treat 16(1–3):354–380

[CR229] Shaw SB, Marjerison RD, Bouldin DR, Parlange JY, Walter MT (2012) Simple model of changes in stream chloride levels attributable to road salt applications. J Environ Eng 138(1):112–118

[CR230] Shelton SA, Kaushal SS, Mayer PM, Shatkay RR, Rippy MA, Grant SB, Newcomer-Johnson TA (2024) Salty chemical cocktails as water quality signatures: Longitudinal trends and breakpoints along different US streams. Sci Total Environ: 172777 10.1016/j.scitotenv.2024.172777 PMC11371123 38670384

[CR231] Shirazi YA, Carr EW, Parsons GR, Hoagland P, Ralston DK, Chen J (2019) Increased operational costs of electricity generation in the Delaware River and Estuary from salinity increases due to sea-level rise and a deepened channel. J Environ Manage 244:228–234 31125873 10.1016/j.jenvman.2019.04.056

[CR232] Steinmuller HE, Chambers LG (2018) Can saltwater intrusion accelerate nutrient export from freshwater wetland soils? An experimental approach. Soil Sci Soc Am J 82(1):283–292

[CR233] Stets EG, Kelly VJ, Crawford CG (2014) Long-term trends in alkalinity in large rivers of the conterminous US in relation to acidification, agriculture, and hydrologic modification. Sci Total Environ 488:280–289 24836138 10.1016/j.scitotenv.2014.04.054

[CR234] Stets EG, Lee CJ, Lytle DA, Schock MR (2018) Increasing chloride in rivers of the conterminous US and linkages to potential corrosivity and lead action level exceedances in drinking water. Sci Total Environ 613:1498–1509 28797521 10.1016/j.scitotenv.2017.07.119 PMC7390064

[CR235] Stewart MG, Wang X, Nguyen MN (2011) Climate change impact and risks of concrete infrastructure deterioration. Eng Struct 33(4):1326–1337

[CR236] Stewart RJ, Wollheim WM, Miara A, Vörösmarty CJ, Fekete B, Lammers RB, Rosenzweig B (2013) Horizontal cooling towers: riverine ecosystem services and the fate of thermoelectric heat in the contemporary Northeast US. Environ Res Lett 8(2):025010

[CR237] Stirpe CR, Cunningham MA, Menking KM (2017) How will climate change affect road salt export from watersheds? Water Air Soil Pollut 228:1–1529225380

[CR238] Sutter LA, Chambers RM, Perry Iii JE (2015) Seawater intrusion mediates species transition in low salinity, tidal marsh vegetation. Aquat Bot 122:32–39

[CR239] Tarolli P, Luo J, Straffelini E, Liou YA, Nguyen KA, Laurenti R, Masin R, D’Agostino V (2023) Saltwater intrusion and climate change impact on coastal agriculture. Plos Water 2(4):e0000121

[CR240] Tassone SJ, Besterman AF, Buelo CD, Walter JA, Pace ML (2022) Co-occurrence of aquatic heatwaves with atmospheric heatwaves, low dissolved oxygen, and low pH events in estuarine ecosystems. Estuaries Coasts 45(3):707–720

[CR241] Thorslund J, Bierkens MF, Oude Essink GH, Sutanudjaja EH, van Vliet MT (2021) Common irrigation drivers of freshwater salinisation in river basins worldwide. Nat Commun 12(1):4232 34244500 10.1038/s41467-021-24281-8 PMC8270903

[CR242] Tian R (2019) Factors controlling saltwater intrusion across multi-time scales in estuaries, Chester River, Chesapeake Bay. Estuar Coast Shelf Sci 223:61–73

[CR243] Tripler CE, Kaushal SS, Likens GE, Todd Walter M (2006) Patterns in potassium dynamics in forest ecosystems. Ecol Lett 9(4):451–466 16623731 10.1111/j.1461-0248.2006.00891.x

[CR244] Trowbridge PR, Kahl JS, Sassan DA, Heath DL, Walsh EM (2010) Relating road salt to exceedances of the water quality standard for chloride in New Hampshire streams. Environ Sci Technol 44:4903–4909 20545352 10.1021/es100325j

[CR245] Tully KL, Weissman D, Wyner WJ, Miller J, Jordan T (2019a) Soils in transition: saltwater intrusion alters soil chemistry in agricultural fields. Biogeochemistry 142:339–356

[CR246] Tully K, Gedan K, Epanchin-Niell R, Strong A, Bernhardt ES, BenDor T, Mitchell M, Kominoski J, Jordan TE, Neubauer SC, Weston NB (2019b) The invisible flood: The chemistry, ecology, and social implications of coastal saltwater intrusion. Bioscience 69(5):368–378

[CR247] Turner A (2003) Salting out of chemicals in estuaries: implications for contaminant partitioning and modelling. Sci Total Environ 314:599–612 14499553 10.1016/s0048-9697(03)00076-7

[CR248] Turner RE (2021) Declining bacteria, lead, and sulphate, and rising pH and oxygen in the lower Mississippi River. Ambio 50(9):1731–1738 33550571 10.1007/s13280-020-01499-2 PMC7868078

[CR249] Tyree M, Clay N, Polaskey S, Entrekin S (2016) Salt in our streams: even small sodium additions can have negative effects on detritivores. Hydrobiologia 775:109–122

[CR251] Ury EA, Ardón M, Wright JP, Bernhardt ES (2023) Restored forested wetland surprisingly resistant to experimental salinization. PLoS ONE 18(12):e0296128 38128024 10.1371/journal.pone.0296128 PMC10734931

[CR252] Valle-Levinson A, Li M (2023) Climate change and saltwater intrusion in estuaries. Climate change and estuaries. CRC Press, pp 99–112

[CR253] van Aalst MA, Koomen E, de Groot HLF (2023) Vulnerability and resilience to drought and saltwater intrusion of rice farming households in the Mekong delta, Vietnam. Econ Disasters Clim Change 7(3):407–430

[CR254] Van Gray JB, Ayayee P (2024) Examining the impacts of salt specificity on freshwater microbial community and functional potential following salinization. Environ Microbiol 26(5):e16628 38757470 10.1111/1462-2920.16628

[CR255] Van Meter KJ, Ceisel E (2024) Road salt legacies: quantifying fluxes of chloride to groundwater and surface water across the Chicago Metropolitan statistical area. Water Resour Res 60(2):e2023WR035103

[CR256] Van Meter RJ, Swan CM, Leips J, Snodgrass JW (2011) Road salt stress induces novel food web structure and interactions. Wetlands 31:843–851

[CR257] Van Vliet MTH, Ludwig F, Zwolsman JJG, Weedon GP, Kabat P (2011) Global river temperatures and sensitivity to atmospheric warming and changes in river flow. Water Resour Res. 10.1029/2010WR009198

[CR258] Van Vliet MT, Franssen WH, Yearsley JR, Ludwig F, Haddeland I, Lettenmaier DP, Kabat P (2013) Global river discharge and water temperature under climate change. Glob Environ Change 23(2):450–464

[CR259] Van Vliet MT, Thorslund J, Strokal M, Hofstra N, Flörke M, Ehalt Macedo H, Nkwasa A, Tang T, Kaushal SS, Kumar R, Van Griensven A (2023) Global river water quality under climate change and hydroclimatic extremes. Nat Rev Earth Environ 4(10):687–702

[CR260] Venâncio C, Ribeiro R, Lopes I (2022) Seawater intrusion: an appraisal of taxa at most risk and safe salinity levels. Biol Rev 97(1):361–382 34626061 10.1111/brv.12803

[CR261] Waldbusser GG, Voigt EP, Bergschneider H, Green MA, Newell RI (2011) Biocalcification in the eastern oyster (Crassostrea virginica) in relation to long-term trends in Chesapeake Bay pH. Estuaries Coasts 34:221–231

[CR262] Walker RH, Smith GD, Hudson SB, French SS, Walters AW (2020) Warmer temperatures interact with salinity to weaken physiological facilitation to stress in freshwater fishes. Conserv Physiol 8(1):coaa107 33365130 10.1093/conphys/coaa107 PMC7745714

[CR263] Watmough SA, Dillon PJ (2004) Major element fluxes from a coniferous catchment in central Ontario, 1983–1999. Biogeochemistry 67:369–399

[CR264] Weissman DS, Tully KL (2020) Saltwater intrusion affects nutrient concentrations in soil porewater and surface waters of coastal habitats. Ecosphere 11(2):e03041

[CR265] Weissman D, Ouyang T, Tully KL (2021) Saltwater intrusion affects nitrogen, phosphorus and iron transformations under oxic and anoxic conditions: an incubation experiment. Biogeochemistry 154(3):451–469

[CR266] Weston NB, Vile MA, Neubauer SC, Velinsky DJ (2011) Accelerated microbial organic matter mineralization following salt-water intrusion into tidal freshwater marsh soils. Biogeochemistry 102:135–151

[CR267] Weston NB, Neubauer SC, Velinsky DJ, Vile MA (2014) Net ecosystem carbon exchange and the greenhouse gas balance of tidal marshes along an estuarine salinity gradient. Biogeochemistry 120:163–189

[CR268] White E, Kaplan D (2017) Restore or retreat? Saltwater intrusion and water management in coastal wetlands. Ecosyst Health Sustain 3(1):e01258

[CR269] White EE Jr, Ury EA, Bernhardt ES, Yang X (2022) Climate change driving widespread loss of coastal forested wetlands throughout the North American coastal plain. Ecosystems 25(4):812–827

[CR270] Widney SE, Smith D, Herbert ER, Schubauer-Berigan JP, Li F, Pennings SC, Craft CB (2019) Chronic but not acute saltwater intrusion leads to large release of inorganic N in a tidal freshwater marsh. Sci Total Environ 695:133779 31412302 10.1016/j.scitotenv.2019.133779

[CR271] Williams WD (1999) Salinisation: a major threat to water resources in the arid and semi-arid regions of the world. Lakes Reserv Res Manag 4(3–4):85–91

[CR272] Wright RF, Norton SA, Brakke DF, Frogner T (1988) Experimental verification of episodic acidification of freshwaters by sea salts. Nature 334(6181):422–424

[CR273] Wu G, Wang K (2021) Observed response of precipitation intensity to dew point temperature over the contiguous US. Theoret Appl Climatol 144:1349–1362

[CR274] Wulkowicz GM, Saleem ZA (1974) Chloride balance of an urban basin in the Chicago area. Water Resour Res 10:974–982

[CR275] Xie R, Rao P, Pang Y, Shi C, Li J, Shen D (2020) Salt intrusion alters nitrogen cycling in tidal reaches as determined in field and laboratory investigations. Sci Total Environ 729:138803 32361438 10.1016/j.scitotenv.2020.138803

[CR276] Xu N, Huang B, Hu Z, Tang Y, Duan S, Zhang C (2017) Effects of temperature, salinity, and irradiance on the growth of harmful algal bloom species Phaeocystis globosa Scherffel (Prymnesiophyceae) isolated from the South China Sea. Chin J Oceanol Limnol 35(3):557–565

[CR277] Yildirim H, Ilica T, Sengul O (2011) Effect of cement type on the resistance of concrete against chloride penetration. Constr Build Mater 25(3):1282–1288

[CR278] Yin C, Pan CG, Xiao SK, Wu Q, Tan HM, Yu K (2022) Insights into the effects of salinity on the sorption and desorption of legacy and emerging per-and polyfluoroalkyl substances (PFASs) on marine sediments. Environ Pollut 300:118957 35124123 10.1016/j.envpol.2022.118957

[CR279] Yu J, Zhu H, Shutes B, Wang X (2022) Salt-alkalization may potentially promote Microcystis aeruginosa blooms and the production of microcystin-LR. Environ Pollut 301:118971 35167928 10.1016/j.envpol.2022.118971

[CR280] Yuan X, Wang Y, Ji P, Wu P, Sheffield J, Otkin JA (2023) A global transition to flash droughts under climate change. Science 380(6641):187–191 37053316 10.1126/science.abn6301

[CR281] Zaitchik BF, Rodell M, Biasutti M, Seneviratne SI (2023) Wetting and drying trends under climate change. Nat Water 1:1–12

[CR282] Zalizniak L, Kefford BJ, Nugegoda D (2009) Effects of pH on salinity tolerance of selected freshwater invertebrates. Aquat Ecol 43:135–144

[CR283] Zhang Z, Dehoff AD, Pody RD, Balay JW (2010a) Detection of streamflow change in the Susquehanna River Basin. Water Resour Manage 24:1947–1964

[CR284] Zhang Z, Cui B, Zhao H, Fan X, Zhang H (2010b) Discharge-salinity relationships in Modaomen waterway, Pearl River estuary. Procedia Environ Sci 2:1235–1245

[CR286] Zhao Y, Wang G, Zhao M, Wang M, Liu B, Jiang M (2022) How soil salinization and alkalinization drive vegetation change in salt-affected inland wetlands. Plant Soil 480(1):571–581

[CR287] Zhou J, Yin S, Fu Q, Wang Q, Huang Q, Wang J (2021) Microbial-induced concrete corrosion under high-salt conditions: microbial community composition and environmental multivariate association analysis. Int Biodeterior Biodegrad 164:105287

