# Supplementary material for: Freshwater faces a warmer and saltier future from headwaters to coasts: climate risks, saltwater intrusion, and biogeochemical chain reactions
Source: Biogeochemistry. 2025 Mar 10;168(2):31. doi: 10.1007/s10533-025-01219-6 (PMC11893707; doi:10.1007/s10533-025-01219-6)
Supplement: Supplementary file 1 — Supplementary file1 (PDF 4077 KB) [file 10533_2025_1219_MOESM1_ESM.pdf]

Freshwater Faces a Warmer and Saltier Future from Headwaters to Coasts: Climate Risks, Saltwater Intrusion, and Biogeochemical Chain Reactions

Published in *Biogeochemistry*

Sujay S. Kaushal[1*], Sydney A. Shelton[1], Paul M. Mayer[2], Bennett Kellmayer[1], Ryan M. Utz[3], Jenna E. Reimer[4], Jenna Baljunas[3], Shantanu V. Bhide[5], Ashley Mon[1], Bianca M. Rodriguez-Cardona[6], Stanley B. Grant[5], Tamara A. Newcomer-Johnson[7], Joseph T. Malin[1], Ruth R. Shatkay[1], Daniel C. Collison[1], Kyriaki Papageorgiou[1], Jazmin Escobar[1], Megan A. Rippy[5], Gene E. Likens[8,9], Raymond G. Najjar[10], Alfonso I. Mejia[11], Allison Lassiter[12], Ming Li[13], and Robert J. Chant[14]

[1]Department of Geology & Earth System Science Interdisciplinary Center, University of Maryland, College Park, MD, USA

[2]US Environmental Protection Agency, Office of Research and Development, Center for Public Health and Environmental Assessment, Pacific Ecological Systems Division, Corvallis OR, USA

[3]Chattham University, Gibsonia, PA, USA

[4]Department of Soil & Water Sciences, University of Florida, Gainesville, FL, USA

[5]Occoquan Watershed Monitoring Laboratory, The Charles E. Via Jr Department of Civil and Environmental Engineering, Virginia Tech, Manassas, VA, USA

[6]Université du Québec à Montréal, Montréal, Canada, Groupe de Recherche Interuniversitaire en Limnologie (GRIL), Montréal, Canada

[7]US Environmental Protection Agency, Office of Research and Development, Center for Environmental Measurement and Modeling, Watershed and Ecosystem Characterization Division, Cincinnati, OH, USA

[8]Cary Institute of Ecosystem Studies, Millbrook, NY, USA

[9]University of Connecticut, Storrs, CT, USA

[10]Department of Meteorology and Atmospheric Science, The Pennsylvania State University, University Park, PA, USA

[11]Civil and Environmental Engineering, The Pennsylvania State University, University Park, PA, USA

[12]University of Pennsylvania Weitzman School of Design, Philadelphia, PA, USA

[13]University of Maryland Center for Environmental Science, Horn Point Laboratory, Cambridge, MD, USA

[14]Institute of Marine and Coastal Science, Rutgers, The State University of New Jersey, New Brunswick, NJ, USA

*Corresponding author: skaushal@umd.edu

Brief Description of Supporting Information

Many freshwaters are experiencing substantial shifts in the concentrations and compositions of salt ions coming from both land and sea. We synthesized a risk framework for anticipating how climate change and increasing salt pollution coming from both land and saltwater intrusion will trigger biogeochemical chain reactions extending from headwaters to tidal waters. Our supplementary information provides information regarding the data sets, sites, and methods that we used to develop our risk framework. For example, we provide methods regarding our analysis of increasing long-term trends in concentrations and loads of major ions in rivers along the eastern U.S. We also provide information for U.S. Geological Survey (USGS) sites across the U.S. where we analyzed: (1) varying salt ion concentration-discharge relationships; (2) relationships between specific conductance and Na^{+}, Cl^{-}, SO_4^{2-}, Ca^{2+}, Mg^{2+}, K^{+}, and N concentrations; (3) changes in relationships between concentrations of dissolved organic carbon (DOC) and different salt ions. We also provide further information regarding literature reviews synthesizing information on our current state of the knowledge regarding the effects of salinization on the carbon cycle and the effects of salinity on biogeochemical processes. We provide supporting data analyses and methods associated with our original salinization experiments demonstrating changes in organic matter composition, mobilization of nutrients and metals, acidification and alkalinization, changes in oxidation-reduction potentials, and deoxygenation in non-tidal and tidal waters. Finally, we provide information about an analysis of long-term salinity trends throughout the Chesapeake Bay and its tidal tributaries using data from EPA Chesapeake Bay Program sites; specifically, we provide information on rates of change in salinity over years at these Chesapeake Bay Program sites, p values, intercepts, confidence intervals and other parameters. Overall, all of these supplementary materials support our synthesis using a combination of both observational and experimental data on the diverse causes, consequences, and risks of salinization from headwaters to coastal waters.

Supporting Information

Methods for Annual River Flux and Load Calculations

Annual fluxes were calculated using loadflex in R (Appling et al 2015) based on data from United States Geological Survey Water Data for the Nation (USGS 2016). Data were obtained using the dataRetrieval package in R (De Cicco et al 2024) for the Passaic River (USGS gage: 01389500), Patuxent River (01594440), Potomac River (01646580 for chemistry, 01646500 for dicharge), and Susquehanna River (01578310). For concentration data, we queried from the start of the time series to December 31, 2023 for the following parameter codes: 00915 for calcium; 00930 for sodium; 00925 for magnesium; 00935 for potassium; 00945 for sulfate; and 00940 for chloride. For alkalinity, parameter codes 39086, 29801, 90410, and 00410 were compiled into a singular time series for the duration of available data. Any duplicate measurements and measurements from the same day were averaged. The concentration data were combined with daily flow data at each site and then daily load was estimated using the *predictSolute* command in USGS software package loadflex (Appling et al., 2015). The total annual mass load was calculated by summing all of the daily values for the year. The standard error for these annual loads was calculated by taking the square root of the summed daily sum of squares. Both were normalized to basin area for plotting purposes. Each fitted model is available in the supporting information.

References

Appling, A. P., M. C. Leon, and W. H. McDowell. 2015. Reducing bias and quantifying uncertainty in watershed flux estimates: the R package loadflex. Ecosphere 6(12):269. http://dx.doi.org/10.1890/ES14-00517.1

De Cicco, L.A., Hirsch, R.M., Lorenz, D., Watkins, W.D., Johnson, M., 2024, dataRetrieval: R packages for discovering and retrieving water data available from Federal hydrologic web services, v.2.7.15, doi:10.5066/P9X4L3GE

U.S. Geological Survey, 2016, National Water Information System data available on the World Wide Web (USGS Water Data for the Nation), accessed [May 31, 2024], at URL [http://waterdata.usgs.gov/nwis/].

Methods for Analysis of Concentrations at U.S. Geological Survey sites

We analyzed relationships between streamflow and concentrations of major ions and relationships between specific conductance and concentrations of solutes at all of the following 32 USGS sites (below). The 32 sites that were analyzed in this study were chosen based on three criteria: 1) sites that had relevant water quality data (elemental concentrations and ion data) in the USGS National Water Information system; 2) sites that were active until September 2023; and 3) sites that had more than 25 years of data available. Finally, we also analyzed relationships between concentrations of dissolved organic carbon (DOC) and concentrations of Na, Ca, Mg and relationships between DOC and K; for this analysis we only focused only on a subset of the U.S. Geological Survey sites in the eastern U.S. (east of the Mississippi River).

Table S1. Gage numbers and names of U.S. Geological Survey sites that were analyzed for relationships among major ions, discharge, and specific conductance. A subset of sites in the eastern U.S. (East of the Mississippi River) were used for the analysis of relationships between DOC and major ions to focus on a major region.

USGS Gage #	Site Name
01184000	CONNECTICUT RIVER AT THOMPSONVILLE, CT
01578310	SUSQUEHANNA RIVER AT CONOWINGO, MD
01646580	POTOMAC RIVER AT CHAIN BRIDGE, AT WASHINGTON, DC
02335870	SOPE CREEK NEAR MARIETTA, GA
03303280	OHIO RIVER AT CANNELTON DAM AT CANNELTON, IN
03378500	WABASH RIVER AT NEW HARMONY, IN
05082500	RED RIVER OF THE NORTH AT GRAND FORKS, ND
05420500	Mississippi River at Clinton, IA
05465500	Iowa River at Wapello, IA
05490500	Des Moines River at Keosauqua, IA
05587455	MISSISSIPPI RIVER BELOW GRAFTON, IL
06329500	Yellowstone River near Sidney MT
06713500	CHERRY CREEK AT DENVER, CO.
06754000	SOUTH PLATTE RIVER NEAR KERSEY, CO
06805500	Platte River at Louisville, Nebraska
06934500	Missouri River at Hermann, MO
07144100	L ARKANSAS R NR SEDGWICK, KS
07373420	MISSISSIPPI R NR ST. FRANCISVILLE, LA
07374525	Mississippi River at Belle Chasse, LA
07381495	(COE) Atchafalaya River at Melville, LA
07381600	Lower Atchafalaya River at Morgan City, LA
08364000	RIO GRANDE AT EL PASO, TX
09163500	COLORADO RIVER NEAR COLORADO-UTAH STATE LINE
10171000	JORDAN RIVER @ 1700 SOUTH @ SALT LAKE CITY, UT
11074000	SANTA ANA R BL PRADO DAM CA
11447650	SACRAMENTO R A FREEPORT CA
12505450	GRANGER DRAIN AT GRANGER, WA
12510500	YAKIMA RIVER AT KIONA, WA
14201300	ZOLLNER CREEK NEAR MT ANGEL, OR
14206950	FANNO CREEK AT DURHAM, OR
15041200	TAKU R NR JUNEAU AK
15565447	YUKON R AT PILOT STATION AK

Methods for Salt Pulse Tracer Additions in Streams

Salt pulse additions were conducted at Wednesday Hill Brook (WHB) in southeastern New Hampshire in June 2014 and July 2015. Salt, in the form of NaCl, was fully mixed with stream water where 1000g of NaCl were added in June 2014 and 1761g of NaCl were added in

July 2015. The NaCl solutions were released (instantaneously) at the top of the experimental reach near a riffle to facilitate mixing. Samples were collected at a fixed point 100m downstream of the input. The change in NaCl over time created a break-through curve (BTC) where sample were collected in the rising limb, peak, and falling limb.

Samples were filtered through pre-combusted Whatman GF/F glass fiber filters and collected in acid washed bottles and kept frozen until analyzed. We analyzed samples for DOC using high temperature catalytic oxidation in a Shimadzu TOC-V CPH/TNM. Major dissolved cations and anions (Na^+, Cl^-, K^+, $NO_3^{-,}$ SO_4^{2-}, Mg^{2+}, and Ca^2) were analyzed with ion chromatography using a Dionex ICS-1000 with AS40 autosampler. NH_4^+ and PO_4^{3-} analyses were conducted on a SmartChem 200 discrete automated colorimetric analyzer using the alkaline phenate (NH_4^+) and molybdate (PO_4^{3-}) standard methods. There were small amounts of SO_4^{2-}, K^+, Mg^{2+}, and Ca^{2+} in the NaCl salt used for tracer experiments but each is less than 1%.

Additional References Supporting Development of the Conceptual Model: Salt Affects Physics, Chemistry, and Biology of Dissolved Organic Matter (DOM)

Processes
Sodium dispersion of soil aggregates[1]
Cell lysis[2]
Changes in organic matter decomposition and leaching[3]
Flocculation[4]
Salting-in and salting-out[5]
Long-term alkalinization[6]
Short-term acidification[7]

1- Amrhein et al., 1992; Bui, 2017; Green et al., 2008a, 2008b
2- Kinsman-Costello et al., 2022; Schimel et al., 2007; Wood, 2015
3- Almeida Júnior et al., 2020; Canhoto et al., 2017; Gómez et al., 2016; Gonçalves et al., 2019; Herrmann et al., 2022; Martínez et al., 2020; McGuire and Judd, 2020; Setia et al., 2011; Steele and Aitkenhead-Peterson, 2013; Van Meter et al., 2012; Weston et al., 2006; Yang et al., 2020
4- Bui, 2017; Sholkovitz, 1976
5- Hyde et al., 2017; Kaushal et al., 2022; Monteith et al., 2023
6- Curtin et al., 2016; Green et al., 2008b; Tavakkoli et al., 2015
7- Bäckström et al., 2004; Green et al., 2008b

Almeida Júnior, E.S., Martínez, A., Gonçalves, A.L., Canhoto, C., 2020. Combined effects of freshwater salinization and leaf traits on litter decomposition. Hydrobiologia 847, 3427–3435. https://doi.org/10.1007/s10750-020-04348-1

Amrhein, C., Strong, J.E., Mosher, P.A., 1992. Effect of Deicing Salts on Metal and Orgaic Matter Mobilization in Roadside Soils. Environ. Sci. Technol. 26, 703–709.

Bäckström, M., Karlsson, S., Bäckman, L., Folkeson, L., Lind, B., 2004. Mobilisation of heavy metals by deicing salts in a roadside environment. Water Research 38, 720–732. https://doi.org/10.1016/j.watres.2003.11.006

Bui, E.N., 2017. Causes of Soil Salinization, Sodification, and Alkalinization, in: Oxford Research Encyclopedia of Environmental Science. https://doi.org/10.1093/acrefore/9780199389414.013.264

Canhoto, C., Simões, S., Gonçalves, A.L., Guilhermino, L., Bärlocher, F., 2017. Stream salinization and fungal-mediated leaf decomposition: A microcosm study. Science of The Total Environment 599–600, 1638–1645. https://doi.org/10.1016/j.scitotenv.2017.05.101

Curtin, D., Peterson, M.E., Anderson, C.R., 2016. pH-dependence of organic matter solubility: Base type effects on dissolved organic C, N, P, and S in soils with contrasting mineralogy. Geoderma 271, 161–172. https://doi.org/10.1016/j.geoderma.2016.02.009

Gómez, R., Asencio, A.D., Picón, J.M., Del Campo, R., Arce, M.I., del Mar Sánchez-Montoya, M., Suárez, M.L., Vidal-Abarca, M.R., 2016. The effect of water salinity on wood breakdown in semiarid Mediterranean streams. Science of The Total Environment 541, 491–501. https://doi.org/10.1016/j.scitotenv.2015.09.040

Gonçalves, A.L., Simões, S., Bärlocher, F., Canhoto, C., 2019. Leaf litter microbial decomposition in salinized streams under intermittency. Science of The Total Environment 653, 1204–1212. https://doi.org/10.1016/j.scitotenv.2018.11.050

Green, S.M., Machin, R., Cresser, M.S., 2008a. Effect of long-term changes in soil chemistry induced by road salt applications on N-transformations in roadside soils. Environmental Pollution 152, 20–31. https://doi.org/10.1016/j.envpol.2007.06.005

Green, S.M., Machin, R., Cresser, M.S., 2008b. Does road salting induce or ameliorate DOC mobilisation from roadside soils to surface waters in the long term? Environ Monit Assess 153, 435. https://doi.org/10.1007/s10661-008-0369-4

Herrmann, M.C., Entrekin, S.A., Evans-White, M.A., Clay, N.A., 2022. Salty water and salty leaf litter alters riparian detrital processes: Evidence from sodium-addition laboratory mesocosm experiments. Science of The Total Environment 806, 151392. https://doi.org/10.1016/j.scitotenv.2021.151392

Hyde, A.M., Zultanski, S.L., Waldman, J.H., Zhong, Y.-L., Shevlin, M., Peng, F., 2017. General Principles and Strategies for Salting-Out Informed by the Hofmeister Series. Org. Process Res. Dev. 21, 1355–1370. https://doi.org/10.1021/acs.oprd.7b00197

Kaushal, S.S., Reimer, J.E., Mayer, P.M., Shatkay, R.R., Maas, C.M., Nguyen, W.D., Boger, W.L., Yaculak, A.M., Doody, T.R., Pennino, M.J., Bailey, N.W., Galella, J.G., Weingrad, A., Collison, D.C., Wood, K.L., Haq, S., Newcomer-Johnson, T.A., Duan, S., Belt, K.T., 2022. Freshwater salinization syndrome alters retention and release of chemical cocktails along flowpaths: From stormwater management to urban streams. Freshwater Science 41, 420–441. https://doi.org/10.1086/721469

Kinsman-Costello, L., Bean, E., Goeckner, A., Matthews, J.W., O'Driscoll, M., Palta, M.M., Peralta, A.L., Reisinger, A.J., Reyes, G.J., Smyth, A.R., Stofan, M., 2022. Mud in the city: Effects of freshwater salinization on inland urban wetland nitrogen and phosphorus availability and export. Limnol Oceanogr Letters lol2.10273. https://doi.org/10.1002/lol2.10273

Martínez, A., Barros, J., Gonçalves, A.L., Canhoto, C., 2020. Salinisation effects on leaf litter decomposition in fresh waters: Does the ionic composition of salt matter? Freshwater Biology 65, 1475–1483. https://doi.org/10.1111/fwb.13514

McGuire, K.M., Judd, K.E., 2020. Road salt chloride retention in wetland soils and effects on dissolved organic carbon export. Chemistry and Ecology 36, 342–359. https://doi.org/10.1080/02757540.2020.1735376

Monteith, D.T., Henrys, P.A., Hruška, J., de Wit, H.A., Krám, P., Moldan, F., Posch, M., Räike, A., Stoddard, J.L., Shilland, E.M., Pereira, M.G., Evans, C.D., 2023. Long-term rise in riverine dissolved organic carbon concentration is predicted by electrolyte solubility theory. Science Advances 9, eade3491. https://doi.org/10.1126/sciadv.ade3491

Schimel, J., Balser, T.C., Wallenstein, M., 2007. Microbial Stress-Response Physiology and Its Implications for Ecosystem Function. Ecology 88, 1386–1394. https://doi.org/10.1890/06-0219

Setia, R., Marschner, P., Baldock, J., Chittleborough, D., Smith, P., Smith, J., 2011. Salinity effects on carbon mineralization in soils of varying texture. Soil Biology and Biochemistry, 19th International Symposium on Environmental Biogeochemistry 43, 1908–1916. https://doi.org/10.1016/j.soilbio.2011.05.013

Sholkovitz, E.R., 1976. Flocculation of dissolved organic and inorganic matter during the mixing of river water and seawater. Geochimica et Cosmochimica Acta 40, 831–845. https://doi.org/10.1016/0016-7037(76)90035-1

Steele, M.K., Aitkenhead-Peterson, J.A., 2013. Salt impacts on organic carbon and nitrogen leaching from senesced vegetation. Biogeochemistry 112, 245–259. https://doi.org/10.1007/s10533-012-9722-3

Tavakkoli, E., Rengasamy, P., Smith, E., McDonald, G.K., 2015. The effect of cation–anion interactions on soil pH and solubility of organic carbon. European Journal of Soil Science 66, 1054–1062. https://doi.org/10.1111/ejss.12294

Van Meter, R.J., Swan, C.M., Trossen, C.A., 2012. Effects of road deicer (NaCl) and amphibian grazers on detritus processing in pond mesocosms. Environmental Toxicology and Chemistry 31, 2306–2310. https://doi.org/10.1002/etc.1949

Weston, N.B., Dixon, R.E., Joye, S.B., 2006. Ramifications of increased salinity in tidal freshwater sediments: Geochemistry and microbial pathways of organic matter mineralization. Journal of Geophysical Research: Biogeosciences 111. https://doi.org/10.1029/2005JG000071

Wood, J.M., 2015. Bacterial responses to osmotic challenges. J Gen Physiol 145, 381–388. https://doi.org/10.1085/jgp.201411296

Yang, C., Wang, X., Miao, F., Li, Z., Tang, W., Sun, J., 2020. Assessing the effect of soil salinization on soil microbial respiration and diversities under incubation conditions. Applied Soil Ecology 155, 103671. https://doi.org/10.1016/j.apsoil.2020.103671

Methods for Laboratory Salinization Incubation Experiments

Incubation experiments were based on Duan and Kaushal 2015, Haq et al 2018, and Gallela et al 2023. For the incubation experiments, streamwater and sediment (from the streambed or floodplain) were collected at Campus Creek (38.99403, -76.95014), Paint Branch (38.99104, -76.93539), Rock Creek (38.9725, -77.04), the Northeast Branch of the Anacostia River (38.95990, -76.92620), and Kenilworth Aquatic Gardens near Watts Branch (38.90559, −76.94851) in the Washington, DC metropolitan area. Sediment samples were then homogenized for particle size by sieving with a 4 mm sieve, using the fine fraction for the incubation. pH, conductivity, temperature, dissolved oxygen, and oxidation-reduction potential were measured in situ using a YSI ProPlus Multiparameter Probe (Yellow Springs Instruments [YSI], Ohio, U.S.A.). Streamwater was then dosed with NaCl to have 1g $Cl^- L^{-1}$, 2.5g $Cl^- L^{-1}$, 5g $Cl^- L^{-1}$, and 10g $Cl^- L^{-1}$ of additional salt. Once dosed, another measurement was taken using the multiparameter probe to have before incubation values for pH, conductivity, temperature, dissolved oxygen, and oxidation-reduction potential. Each of these treatments were replicated in triplicates. For each replicate, 60 ± 0.1 g of homogenized sediment were placed in acid-washed 125 mL Erlenmeyer flasks. Then, 120 mL of water was pipetted onto the sediment in each of the flasks. For controls, streamwater + sediment without added salt was incubated in triplicate, and plain streamwater and sediment + deionized water were incubated in duplicate. The flasks were then capped loosely with aluminum foil to limit evaporation but allow for air exchange to simulate open system conditions. The flasks were incubated on a shaking table at slow mode in the dark for 24 h at room temperature (20°C; temperature is approximate). After the incubation, water was immediately removed from the flask using a pipette to avoid any disturbance to the sediment. A final measurement was taken with the multiparameter probe to have after incubation values for pH, conductivity, temperature, dissolved oxygen, and oxidation-reduction potential. The water was then filtered through ashed Whatman GF/F filters. Samples were then refrigerated at 4°C awaiting further analysis; if analysis was not completed within two weeks the samples were then frozen. Dissolved organic carbon, dissolved inorganic carbon, and total dissolved nitrogen was analyzed using a Shimadzu Total Organic Carbon analyzer (TOC-L, Shimadzu, Columbia, Maryland, U.S.A). Organic matter optical properties were analyzed using a Horiba Aqualog spectrofluorometer (Horiba, Piscataway, New Jersey, U.S.A.) and the indices and Coble's peaks were calculated using staRdom in R (Pucher et al 2019). Briefly, organic matter indices were identified using staRdom in R (Pucher et al. 2019), with a higher value of BIX representing a larger contribution of recent autochthonous material (Huguet et al. 2009) and higher values of Coble's Peaks (A, C, M, and T) representing larger amounts of protein-like and humic-like organic matter, as associated with each peak given in parentheses (Coble, 1996). Descriptions of optical properties, indices for dissolved organic matter quality, and methods for analyses on the fluorometer can all be found in Kaushal et al. (2023).

References

Duan, S. and Kaushal, S.S., 2015. Salinization alters fluxes of bioreactive elements from stream ecosystems across land use. *Biogeosciences*, 12(23), pp.7331-7347.

Galella, J.G., Kaushal, S.S., Mayer, P.M., Maas, C.M., Shatkay, R.R. and Stutzke, R.A., 2023a. Stormwater best management practices: Experimental evaluation of chemical cocktails mobilized by freshwater salinization syndrome. *Frontiers in Environmental Science*, *11*, p.1020914.

Haq, S., Kaushal, S.S. and Duan, S., 2018. Episodic salinization and freshwater salinization syndrome mobilize base cations, carbon, and nutrients to streams across urban regions. *Biogeochemistry*, *141*, pp.463-486.

Kaushal, S.S., Maas, C.M., Mayer, P.M., Newcomer-Johnson, T.A., Grant, S.B., Rippy, M.A., Shatkay, R.R., Leathers, J., Gold, A.J., Smith, C. and McMullen, E.C., 2023. Longitudinal stream synoptic monitoring tracks chemicals along watershed continuums: a typology of trends. *Frontiers in environmental science*, *11*, p.1122485.

Maas, C.M., Kaushal, S.S., Rippy, M.A., Mayer, P.M., Grant, S.B., Shatkay, R.R., Malin, J.T., Bhide, S.V., Vikesland, P., Krauss, L. and Reimer, J.E., 2023. Freshwater salinization syndrome limits management efforts to improve water quality. *Frontiers in Environmental Science*, 11, p.1106581.

Pucher, Matthias, Urban Wünsch, Gabriele Weigelhofer, Kathleen Murphy, Thomas Hein, and Daniel Graeber. 2019. “staRdom: Versatile Software for Analyzing Spectroscopic Data of Dissolved Organic Matter in R.” *Water* 11 (11): 2366. https://doi.org/10.3390/w11112366.

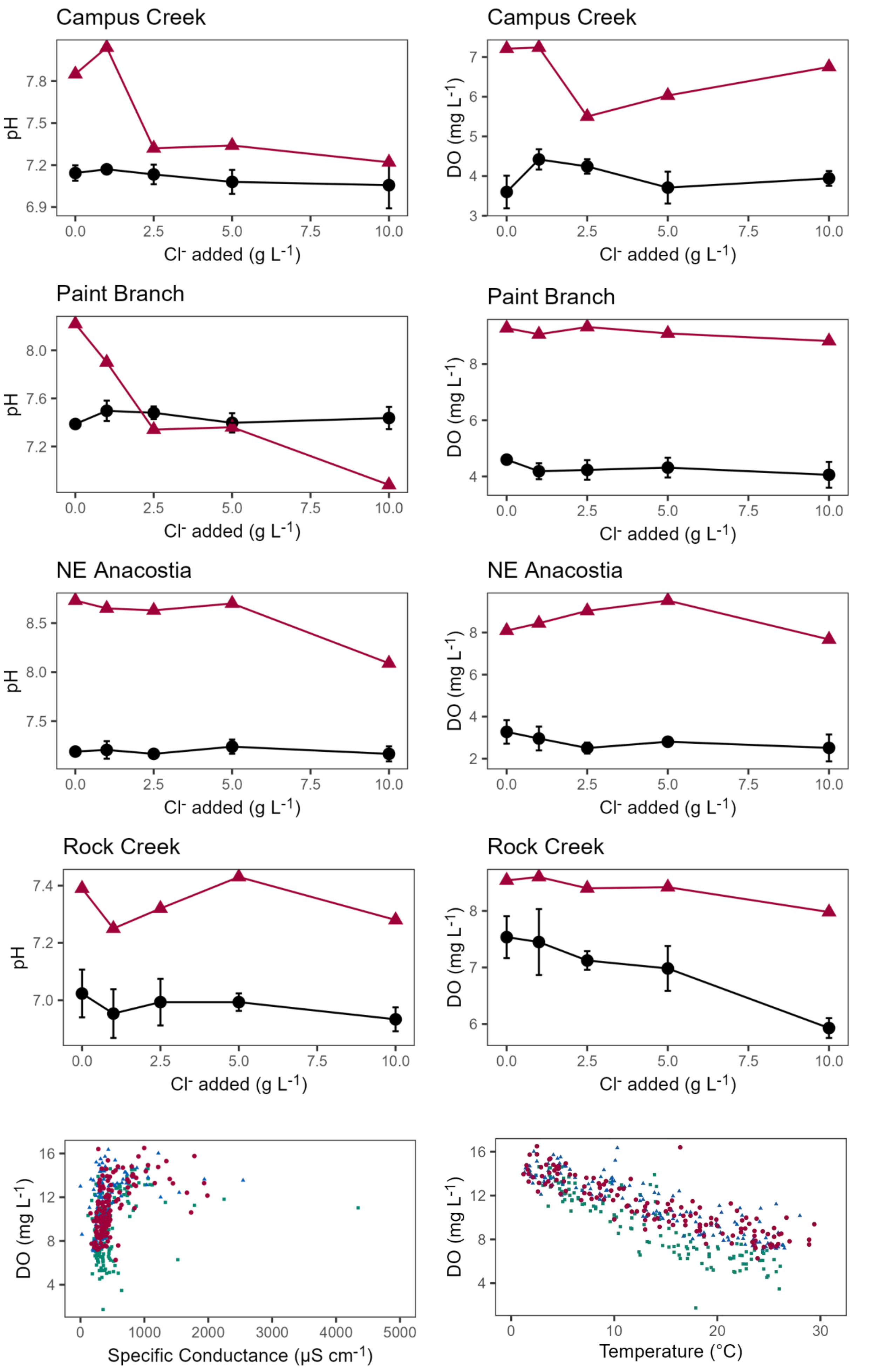

Figure S1. Changes in pH and dissolved oxygen (DO) before and after experimental salt incubations with sediments and streamwater from non-tidal and tidal streams and rivers. Relationships between DO with specific conductance and temperature from weekly monitoring at a subset of the study sites.

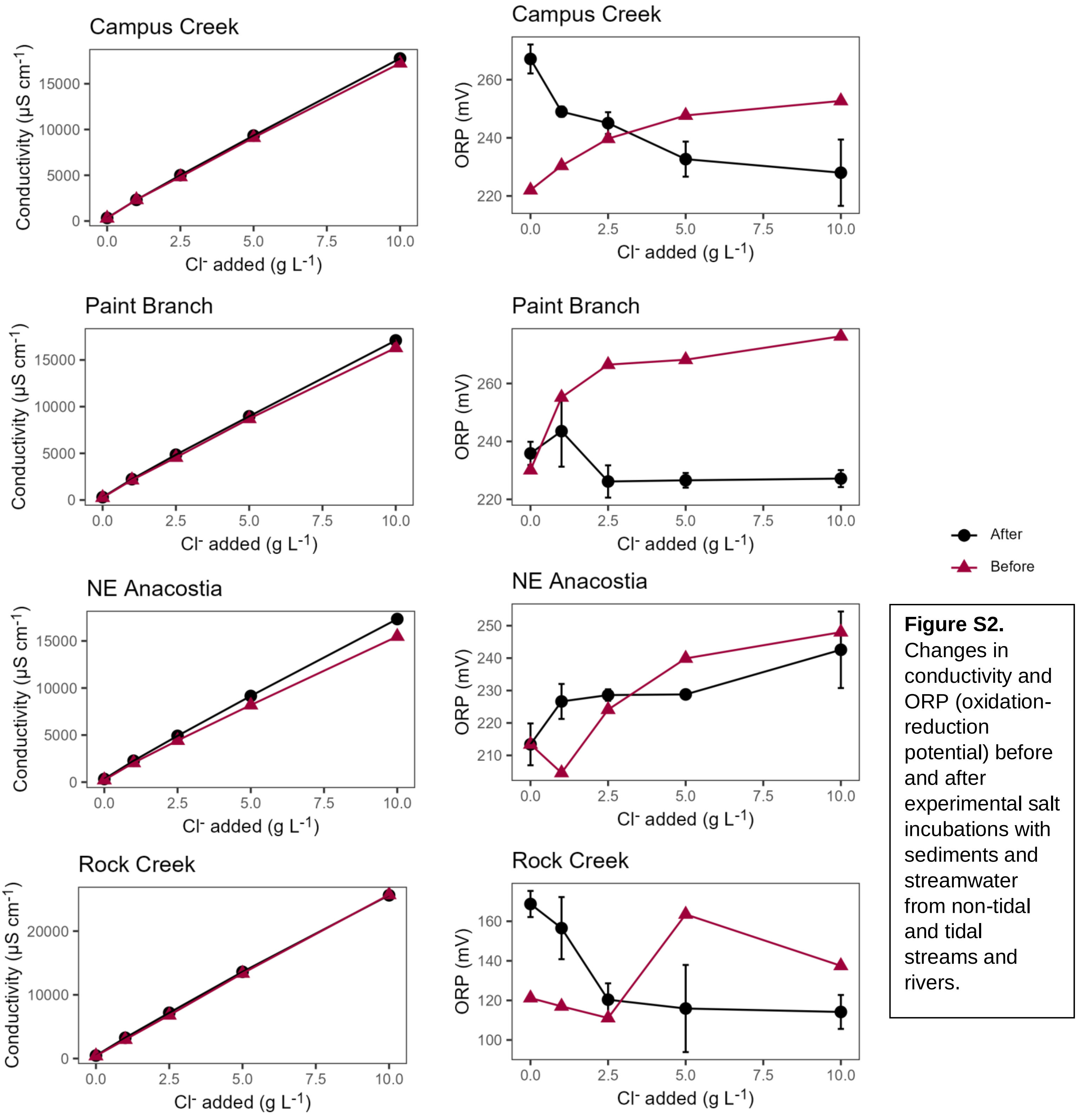

Figure S2. Changes in conductivity and ORP (oxidation-reduction potential) before and after experimental salt incubations with sediments and streamwater from non-tidal and tidal streams and rivers.

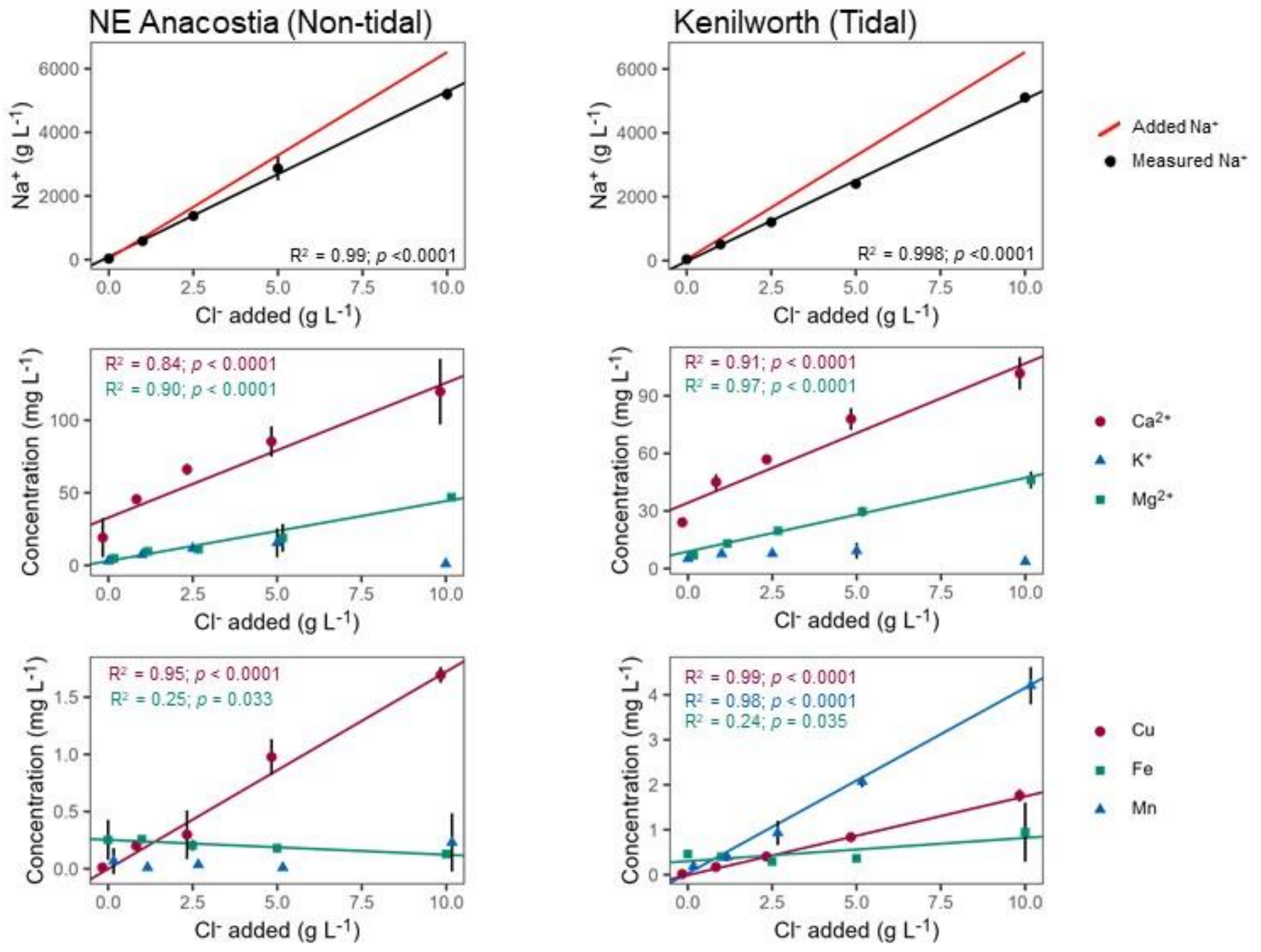

Figure S3. Changes in metals (base cations) and redox-sensitive metals in response to salt incubations. At increased salinity levels, there is mobilization of these metals in response to ion exchange and deoxygenation, particularly in the tidal freshwater Anacostia River. The difference between added Na^+ and measured Na^+ represents Na^+ retention on sediment ion exchange sites.

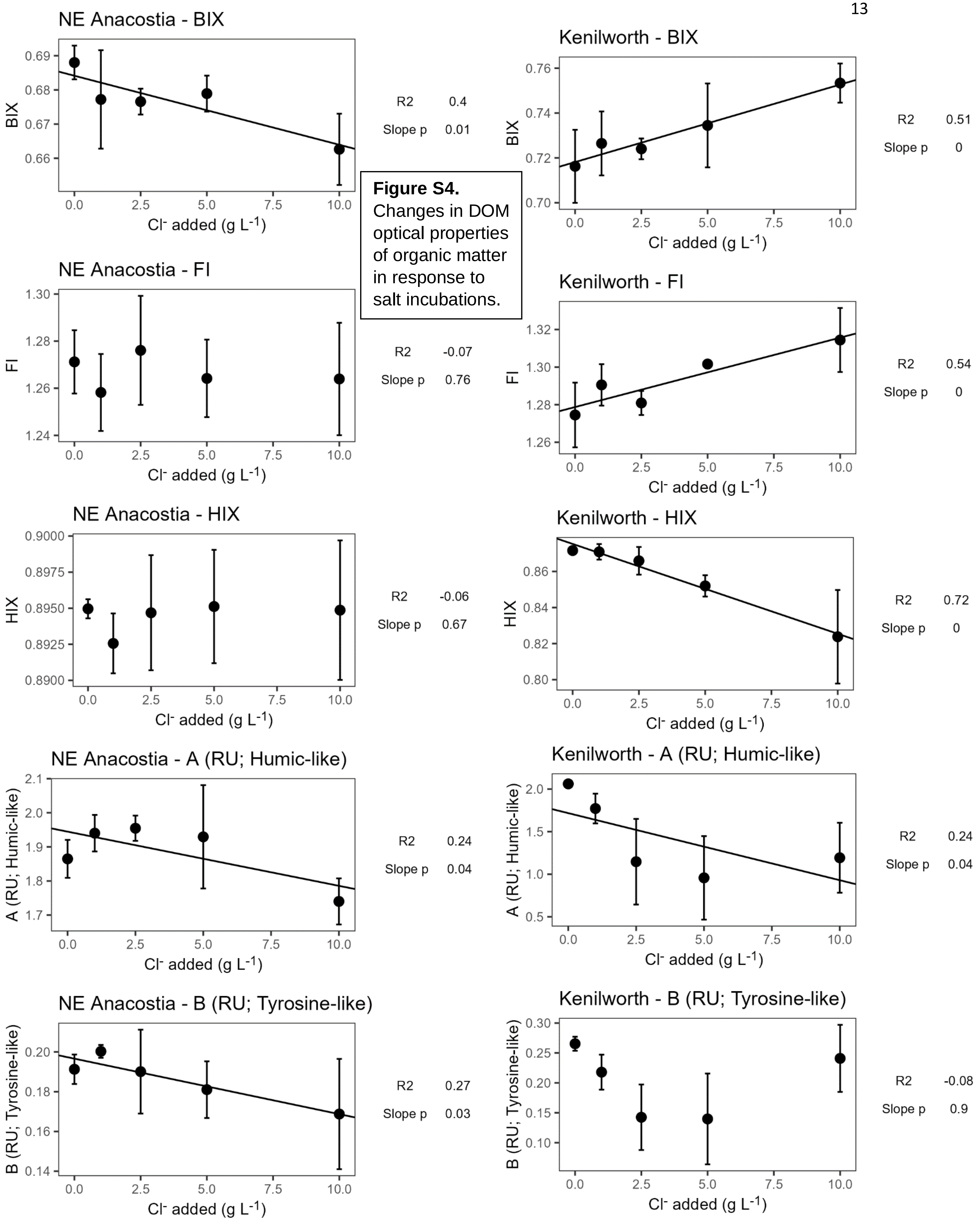

Figure S4. Changes in DOM optical properties of organic matter in response to salt incubations.

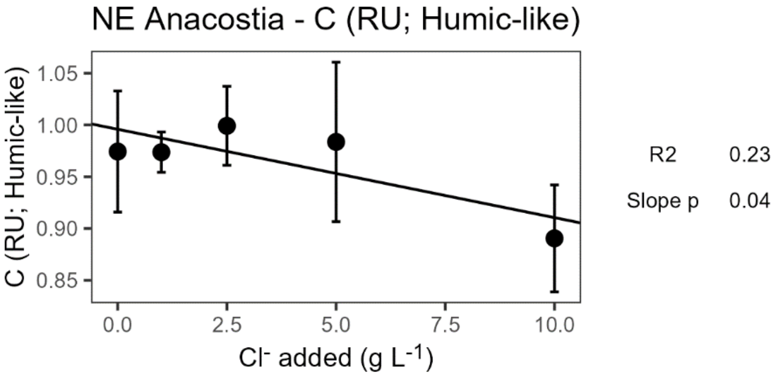

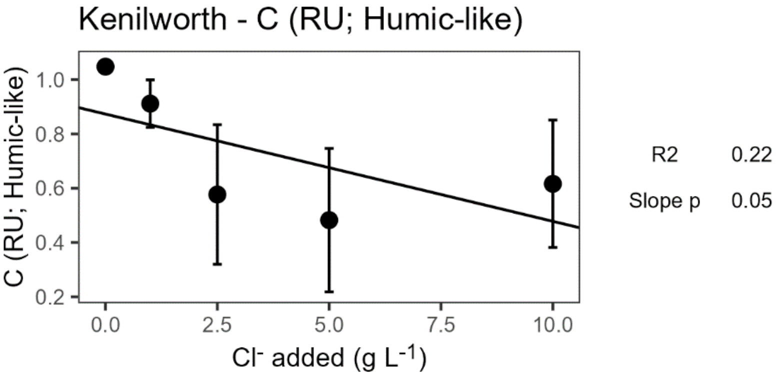

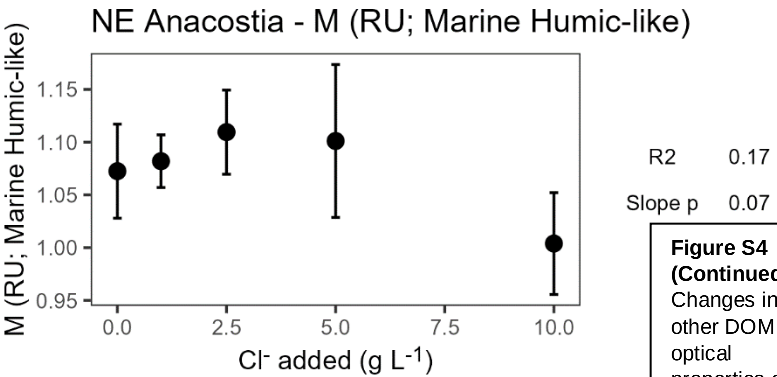

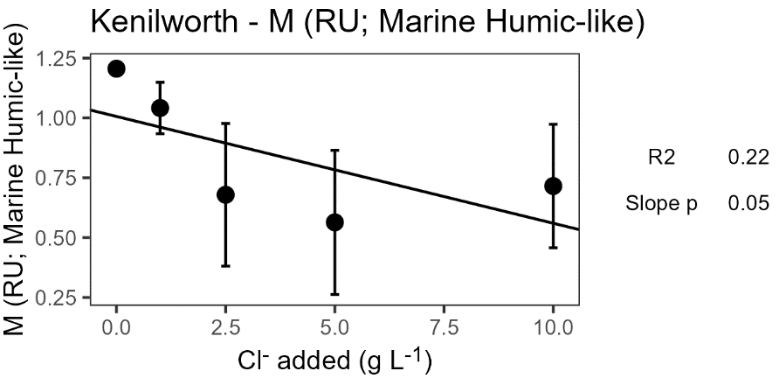

Figure S4 (Continued) Changes in other DOM optical properties of organic matter in response to salt.incu-bations.

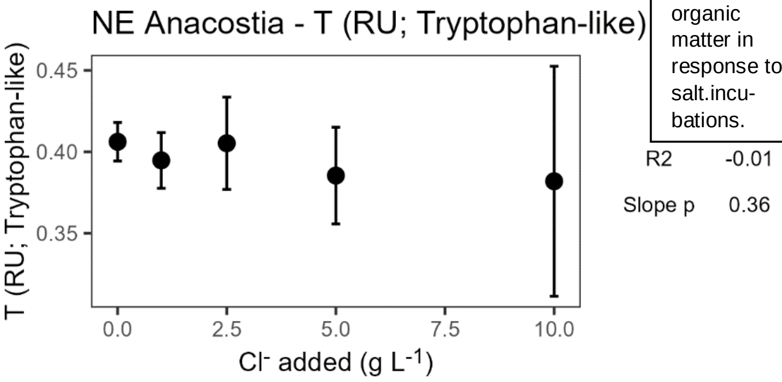

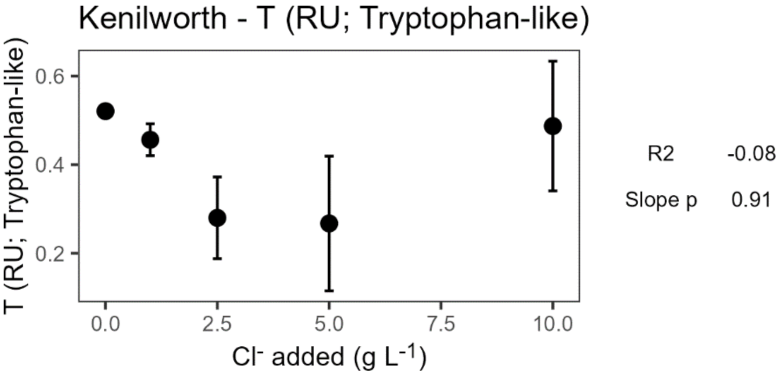

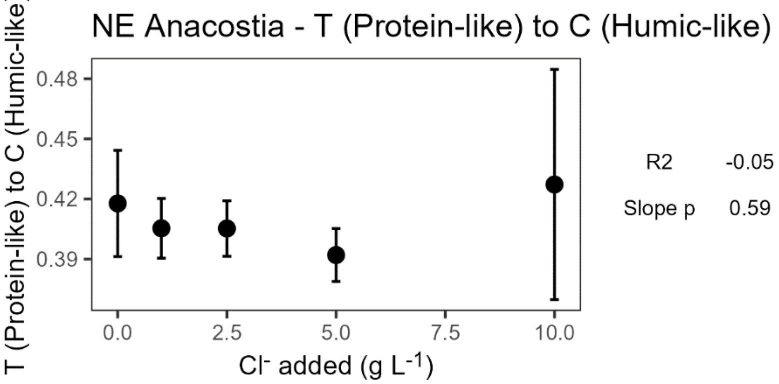

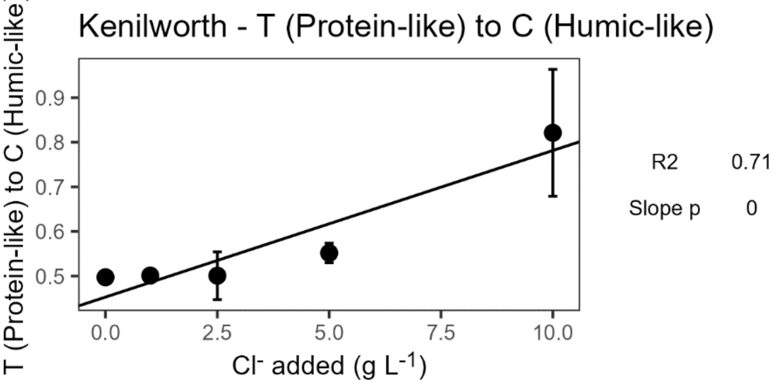

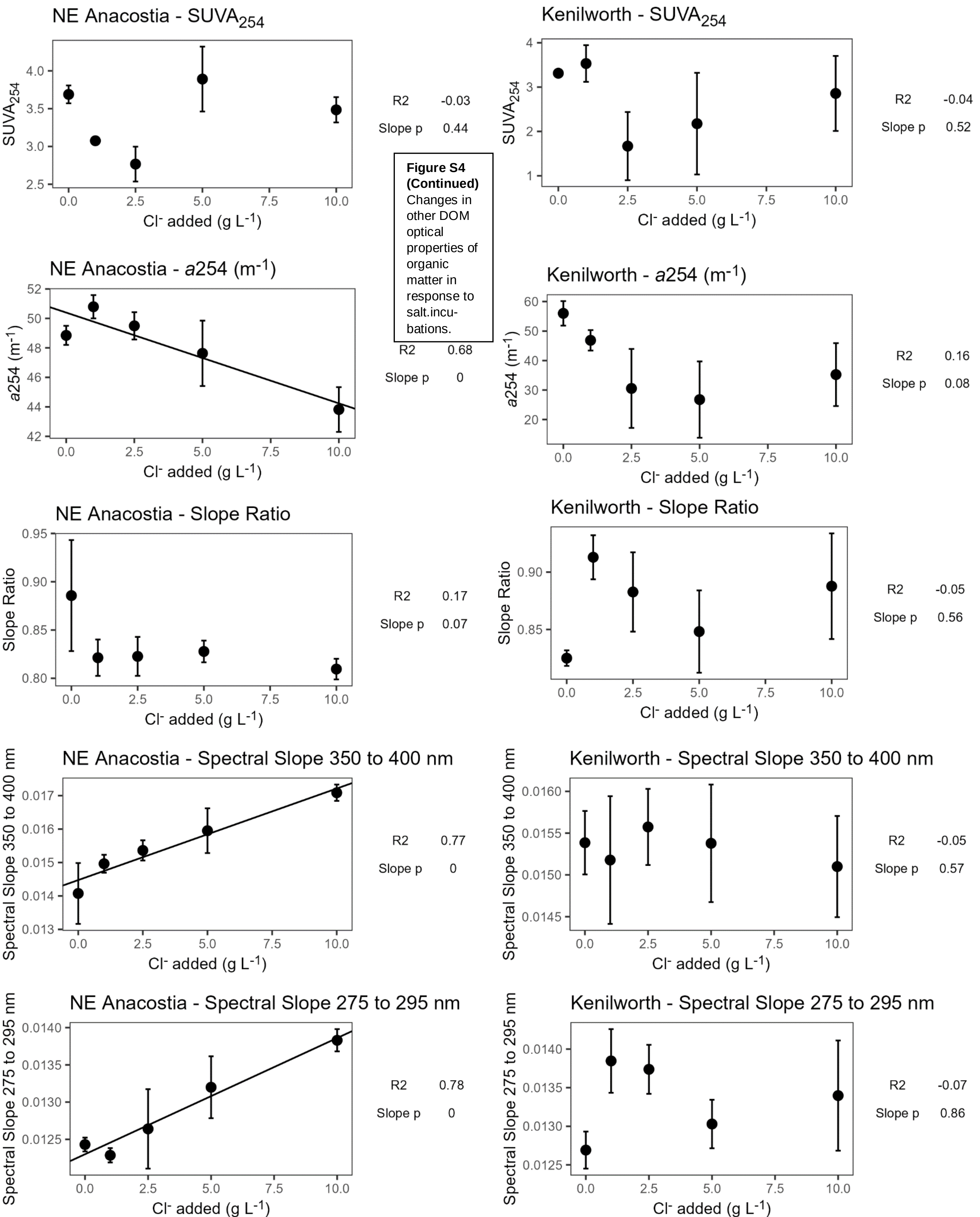

Figure S4 (Continued) Changes in other DOM optical properties of organic matter in response to salt.incu-bations.

Model parameters for changing salinity in Chesapeake Bay sites

The following table of metadata corresponds to the parameters listed in Supplementary Table S2 (starting on the next page), which provides site-specific models for the analysis of long-term salinity trends in the Chesapeake Bay and its tidal tributaries presented in Figure 11. Data is from the EPA Chesapeake Bay Program (CBP) water quality monitoring network.

Term	Description	Units
site	Site number assigned by CPB	
station	Station identity assignment by CPB	
lat	Site latitude	Decimal degrees
lon	Site longitude	Decimal degrees
parameter	Parameter measured by CPB	
depth	Depth of measurement	Meters
n	Sample size at depth and location	
maxDate	Latest date recorded at corresponding site and depth	
yrsRecord	Length of record at corresponding site and depth	Years
slope	Slope estimate from model	Salinity ppt/year
uCI	Upper 95% confidence interval of slope	
lCI	Lower 95% confidence interval of slope	
intercept	Intercept from model	

Table S2. Statistical information regarding analysis of long-term salinity trends at stations throughout Chesapeake Bay and its tidal tributaries.

site	station	Lat	lon	parameter	depth	N	minDate	maxDate	yrsRecord	slope	uCI	lCI	intercept
1178	CB6.2	37.4868	-76.1563	SALINITY	0	25	6/8/1988	3/18/1992	3.778082	-0.94019	0.641772	-2.81121	23.01139
1178	CB6.2	37.4868	-76.1563	SALINITY	1	594	7/11/1984	2/21/2024	39.6411	-0.01581	-0.01052	-0.02633	17.41462
1178	CB6.2	37.4868	-76.1563	SALINITY	10	330	6/27/1984	2/21/2024	39.67945	-0.02513	-0.01877	-0.04199	22.59942
1178	CB6.2	37.4868	-76.1563	SALINITY	11	46	8/7/1984	2/18/2020	35.55616	-0.11715	-0.05053	-0.26149	23.41772
1178	CB6.2	37.4868	-76.1563	SALINITY	2	551	6/27/1984	2/21/2024	39.67945	-0.00977	-0.00411	-0.02125	17.39698
1178	CB6.2	37.4868	-76.1563	SALINITY	3	588	7/11/1984	2/21/2024	39.6411	0.077249	0.08378	0.065378	15.01714
1178	CB6.2	37.4868	-76.1563	SALINITY	4	553	6/27/1984	2/21/2024	39.67945	-0.01678	-0.00765	-0.02474	17.75885
1178	CB6.2	37.4868	-76.1563	SALINITY	5	586	7/11/1984	2/21/2024	39.6411	-0.03549	-0.02378	-0.03978	18.44079
1178	CB6.2	37.4868	-76.1563	SALINITY	6	554	6/27/1984	2/21/2024	39.67945	-0.02907	-0.01515	-0.03341	19.10479
1178	CB6.2	37.4868	-76.1563	SALINITY	7	588	7/11/1984	2/21/2024	39.6411	-0.02461	-0.01633	-0.03416	20.59618
1178	CB6.2	37.4868	-76.1563	SALINITY	8	554	6/27/1984	2/21/2024	39.67945	-0.01837	-0.00571	-0.02414	21.35466
1178	CB6.2	37.4868	-76.1563	SALINITY	9	610	7/11/1984	2/21/2024	39.6411	-0.01346	-0.00413	-0.01894	21.94947
1029	1AACO014.57	38.81123	-77.2303	SALINITY	0.3	45	2/6/2008	8/1/2012	4.487671	-0.00911	0.000563	-0.02343	0.194127
1032	1ACAX004.57	39.25458	-77.5762	SALINITY	0.3	13	11/5/2008	8/1/2011	2.736986	0.003136	0.009652	0.002781	0.095015
1062	2-DPC005.20	37.28389	-77.8683	SALINITY	0	8	5/25/2006	10/23/2008	2.416438	0	NA	NA	0
1062	2-DPC005.20	37.28389	-77.8683	SALINITY	0.3	12	1/6/2004	7/15/2009	5.526027	0	NA	NA	0
1065	2-JMS117.35	37.56158	-77.5438	SALINITY	0.3	12	2/2/2004	4/20/2006	2.213699	0	NA	NA	0
1080	302031	38.84972	-75.6733	SALINITY	0	212	10/12/2011	9/25/2023	11.96164	0	0.013309	0.000766	0.1
1081	304191	38.72917	-75.5614	SALINITY	0	270	10/12/2011	9/25/2023	11.96164	0	-0.00153	-0.00153	0.1
1082	7-DRN010.48	37.63361	-76.6958	SALINITY	0.3	15	1/20/2004	3/17/2005	1.156164	0	NA	NA	0
1085	8-LTL009.54	37.87292	-77.5133	SALINITY	0	10	5/25/2006	11/18/2010	4.487671	0	NA	NA	0
1085	8-LTL009.54	37.87292	-77.5133	SALINITY	0.3	20	1/21/2004	7/15/2009	5.484932	0	NA	NA	0
1088	8-NAR005.42	37.85014	-77.4278	SALINITY	0.3	13	1/21/2004	11/27/2007	3.852055	0	NA	NA	0
1093	ANA0082	38.93893	-76.9434	SALINITY	0	439	1/7/1986	12/6/2023	37.93699	0	0.023316	0.002004	0
1094	ANA01	38.91817	-76.9416	SALINITY	0.1	33	3/29/2010	11/15/2016	6.638356	-0.00984	-0.00362	-0.01732	0.201966
1094	ANA01	38.91817	-76.9416	SALINITY	1	11	4/26/2010	3/23/2015	4.909589	-0.0141	0.034292	-0.02843	0.203825
1094	ANA01	38.91817	-76.9416	SALINITY	2	3	9/21/2010	5/7/2013	2.627397	0.044008	0.057483	-0.00586	0.04775
1098	ANA05	38.90928	-76.9558	SALINITY	0.1	17	5/11/2010	11/15/2016	6.520548	-0.01576	-0.00518	-0.02681	0.199063
1098	ANA05	38.90928	-76.9558	SALINITY	0.44	3	3/8/2016	4/12/2016	0.09589	-1.79929	-1.72489	-1.8737	10.88868
1098	ANA05	38.90928	-76.9558	SALINITY	1	5	3/13/2012	8/14/2012	0.421918	-0.36314	-0.20485	-0.4681	0.956071
1098	ANA05	38.90928	-76.9558	SALINITY	2	4	4/10/2012	4/12/2016	4.008219	0.010549	0.011569	-0.06876	0.163855
1101	ANA08	38.89872	-76.9622	SALINITY	0.1	24	3/16/2010	3/10/2015	4.986301	-0.00849	0.004691	-0.01961	0.20221
1101	ANA08	38.89872	-76.9622	SALINITY	1	9	6/22/2010	4/12/2016	5.810959	-0.04226	-0.01854	-0.09639	0.26766
1101	ANA08	38.89872	-76.9622	SALINITY	2	9	6/22/2010	3/8/2016	5.715068	-0.04226	0.005349	-0.09639	0.26766
1101	ANA08	38.89872	-76.9622	SALINITY	3	5	6/22/2010	4/12/2016	5.810959	-0.01702	0.003939	-0.03852	0.198815
1104	ANA11	38.884	-76.9689	SALINITY	0.1	18	5/11/2010	11/15/2016	6.520548	-0.01871	-0.00654	-0.02504	0.198771
1104	ANA11	38.884	-76.9689	SALINITY	1	4	4/10/2012	8/14/2012	0.345205	-0.14691	-0.14484	-0.23542	0.441746
1104	ANA11	38.884	-76.9689	SALINITY	2	4	6/19/2012	4/12/2016	3.816438	-0.13036	-0.05297	-0.22813	0.405
1107	ANA14	38.87734	-76.9755	SALINITY	0.1	41	3/16/2010	11/15/2016	6.673973	-0.01881	-0.00837	-0.02337	0.187094
1107	ANA14	38.87734	-76.9755	SALINITY	1	23	4/26/2010	9/13/2016	6.389041	-0.01887	-0.01183	-0.02662	0.183172
1107	ANA14	38.87734	-76.9755	SALINITY	1.02	3	3/21/2016	8/21/2016	0.419178	-0.40804	-0.37023	-0.4338	2.752763

1154	CB3.3C	38.99596	-76.3597	SALINITY	17.5	4	6/2/1987	10/5/1988	1.345205	-0.99367	1.21707	-1.2017	17.44633
1154	CB3.3C	38.99596	-76.3597	SALINITY	18	169	7/12/1984	8/8/2023	39.09863	-0.0213	-0.0069	-0.03227	15.77211
1154	CB3.3C	38.99596	-76.3597	SALINITY	19	660	7/12/1984	12/13/2023	39.44658	-0.01288	-0.0051	-0.01861	17.12023
1154	CB3.3C	38.99596	-76.3597	SALINITY	2	577	7/12/1984	12/13/2023	39.44658	0	0.011002	-0.01365	9.012607
1154	CB3.3C	38.99596	-76.3597	SALINITY	20	84	7/12/1984	8/8/2023	39.09863	-0.03484	-0.00639	-0.04792	16.51324
1154	CB3.3C	38.99596	-76.3597	SALINITY	21	654	8/7/1984	12/13/2023	39.37534	-0.00919	-0.0035	-0.01665	17.2214
1154	CB3.3C	38.99596	-76.3597	SALINITY	22	77	9/11/1984	8/8/2023	38.93151	-0.00166	0.021278	-0.02206	16.94997
1154	CB3.3C	38.99596	-76.3597	SALINITY	23	578	12/12/1984	12/13/2023	39.0274	-0.01438	-0.00287	-0.01834	17.34155
1154	CB3.3C	38.99596	-76.3597	SALINITY	24	303	7/9/1985	4/11/2023	37.78082	-0.01794	-0.00652	-0.03074	17.51997
1154	CB3.3C	38.99596	-76.3597	SALINITY	25	52	2/25/1987	1/14/2020	32.90685	0.012063	0.054058	-0.02025	16.0314
1154	CB3.3C	38.99596	-76.3597	SALINITY	26	4	9/28/1993	11/18/2019	26.15616	-0.03902	0.010524	-0.05065	18.22343
1154	CB3.3C	38.99596	-76.3597	SALINITY	3	722	7/12/1984	12/13/2023	39.44658	-0.05598	-0.0384	-0.05759	10.82884
1154	CB3.3C	38.99596	-76.3597	SALINITY	4	488	7/12/1984	12/13/2023	39.44658	-0.04903	-0.02996	-0.0551	10.83036
1154	CB3.3C	38.99596	-76.3597	SALINITY	4.6	60	4/8/1986	1/15/1997	10.78082	-0.12744	0.113103	-0.28034	12.44198
1154	CB3.3C	38.99596	-76.3597	SALINITY	5	668	7/12/1984	12/13/2023	39.44658	-0.05383	-0.0382	-0.05804	11.63226
1154	CB3.3C	38.99596	-76.3597	SALINITY	6	569	7/12/1984	12/13/2023	39.44658	-0.04111	-0.03301	-0.05382	12.14157
1154	CB3.3C	38.99596	-76.3597	SALINITY	6.1	34	4/8/1986	1/15/1997	10.78082	-0.39472	-0.1603	-1.37206	13.51175
1154	CB3.3C	38.99596	-76.3597	SALINITY	7	677	7/12/1984	12/13/2023	39.44658	-0.04467	-0.03707	-0.054	13.02781
1154	CB3.3C	38.99596	-76.3597	SALINITY	7.6	32	4/8/1986	1/15/1997	10.78082	-0.70927	-0.32842	-1.71574	14.7379
1154	CB3.3C	38.99596	-76.3597	SALINITY	8	578	7/12/1984	12/13/2023	39.44658	-0.04255	-0.02802	-0.04775	13.48449
1154	CB3.3C	38.99596	-76.3597	SALINITY	9	670	7/12/1984	12/13/2023	39.44658	-0.04326	-0.03408	-0.05116	14.1944
1154	CB3.3C	38.99596	-76.3597	SALINITY	9.1	52	4/8/1986	1/15/1997	10.78082	-0.08623	0.069321	-0.36447	14.3759
1155	CB3.3E	39.00412	-76.3452	SALINITY	0.5	511	7/12/1984	10/5/2023	39.25753	-0.04868	-0.02923	-0.05378	9.695174
1155	CB3.3E	39.00412	-76.3452	SALINITY	1	506	7/12/1984	10/5/2023	39.25753	-0.04979	-0.03364	-0.05798	9.941409
1155	CB3.3E	39.00412	-76.3452	SALINITY	2	370	7/12/1984	10/5/2023	39.25753	-0.02757	-0.00693	-0.03996	9.810001
1155	CB3.3E	39.00412	-76.3452	SALINITY	3	506	7/12/1984	10/5/2023	39.25753	-0.06069	-0.0416	-0.06522	10.77963
1155	CB3.3E	39.00412	-76.3452	SALINITY	4	346	7/25/1984	10/5/2023	39.22192	-0.0554	-0.03675	-0.06714	11.19396
1155	CB3.3E	39.00412	-76.3452	SALINITY	5	492	7/25/1984	10/5/2023	39.22192	-0.0658	-0.04854	-0.07119	11.81552
1155	CB3.3E	39.00412	-76.3452	SALINITY	6	378	7/12/1984	10/5/2023	39.25753	-0.06047	-0.04473	-0.06867	12.68732
1155	CB3.3E	39.00412	-76.3452	SALINITY	7	442	7/12/1984	10/5/2023	39.25753	-0.06175	-0.04685	-0.06916	13.06757
1155	CB3.3E	39.00412	-76.3452	SALINITY	8	74	7/12/1984	7/27/2022	38.06575	-0.05873	-0.0228	-0.15122	13.59776
1155	CB3.3E	39.00412	-76.3452	SALINITY	9	17	7/12/1984	5/24/2000	15.87671	0.285756	0.612861	0.025913	12.41761
1156	CB3.3W	39.00462	-76.3881	SALINITY	0.5	509	7/12/1984	10/5/2023	39.25753	-0.05371	-0.03901	-0.06171	9.423558
1156	CB3.3W	39.00462	-76.3881	SALINITY	1	506	7/12/1984	10/5/2023	39.25753	-0.05755	-0.04247	-0.06565	9.566966
1156	CB3.3W	39.00462	-76.3881	SALINITY	2	355	7/12/1984	10/5/2023	39.25753	-0.01935	0.000427	-0.03157	8.96899
1156	CB3.3W	39.00462	-76.3881	SALINITY	3	506	7/12/1984	10/5/2023	39.25753	-0.07457	-0.05086	-0.07423	10.34626
1156	CB3.3W	39.00462	-76.3881	SALINITY	4	313	7/12/1984	10/5/2023	39.25753	-0.03239	-0.01093	-0.04351	9.615131
1156	CB3.3W	39.00462	-76.3881	SALINITY	5	491	7/12/1984	10/5/2023	39.25753	-0.07594	-0.05815	-0.08096	11.36172
1156	CB3.3W	39.00462	-76.3881	SALINITY	6	337	7/12/1984	10/5/2023	39.25753	-0.07093	-0.05002	-0.07886	12.202
1156	CB3.3W	39.00462	-76.3881	SALINITY	7	501	7/12/1984	10/5/2023	39.25753	-0.05956	-0.05417	-0.07708	13.1793
1156	CB3.3W	39.00462	-76.3881	SALINITY	8	350	7/12/1984	7/27/2022	38.06575	-0.04034	-0.02739	-0.0565	13.66974
1156	CB3.3W	39.00462	-76.3881	SALINITY	9	23	4/9/1985	4/24/2018	33.06301	-0.20131	-0.07627	-0.43399	16.18578

1168	CB4.3W	38.55728	-76.494	SALINITY	1	499	7/10/1984	10/5/2023	39.26301	-0.05282	-0.0334	-0.0551	12.40795
1168	CB4.3W	38.55728	-76.494	SALINITY	2	322	7/10/1984	10/5/2023	39.26301	-0.00516	0.014166	-0.01908	11.39757
1168	CB4.3W	38.55728	-76.494	SALINITY	3	499	7/10/1984	10/5/2023	39.26301	-0.05005	-0.03307	-0.05437	12.4501
1168	CB4.3W	38.55728	-76.494	SALINITY	4	160	7/24/1984	10/5/2023	39.22466	0.008396	0.039429	-0.00628	11.51011
1168	CB4.3W	38.55728	-76.494	SALINITY	5	482	7/24/1984	10/5/2023	39.22466	-0.03765	-0.02448	-0.04637	12.43748
1168	CB4.3W	38.55728	-76.494	SALINITY	6	191	7/10/1984	10/5/2023	39.26301	-0.03331	-0.01098	-0.04363	12.84158
1168	CB4.3W	38.55728	-76.494	SALINITY	7	488	7/10/1984	10/5/2023	39.26301	-0.03938	-0.02771	-0.04745	13.01871
1168	CB4.3W	38.55728	-76.494	SALINITY	8	313	7/10/1984	10/5/2023	39.26301	-0.05329	-0.03756	-0.0595	13.94121
1168	CB4.3W	38.55728	-76.494	SALINITY	8.5	7	7/24/1984	11/18/1986	2.320548	7.169643	10.87206	3.307987	10.41869
1168	CB4.3W	38.55728	-76.494	SALINITY	9	347	9/10/1984	10/5/2023	39.09315	-0.0132	0.003194	-0.02417	12.89873
1169	CB4.4	38.41457	-76.3457	SALINITY	0.5	683	7/10/1984	12/12/2023	39.44932	-0.03332	-0.01669	-0.03565	13.18958
1169	CB4.4	38.41457	-76.3457	SALINITY	1	661	7/10/1984	12/12/2023	39.44932	-0.03678	-0.01971	-0.03809	13.309
1169	CB4.4	38.41457	-76.3457	SALINITY	10	490	7/10/1984	12/12/2023	39.44932	-0.02823	-0.01462	-0.03283	15.36711
1169	CB4.4	38.41457	-76.3457	SALINITY	11	649	7/10/1984	12/12/2023	39.44932	-0.03285	-0.02149	-0.03721	16.32512
1169	CB4.4	38.41457	-76.3457	SALINITY	12	509	7/10/1984	12/12/2023	39.44932	-0.03483	-0.02413	-0.04183	16.63761
1169	CB4.4	38.41457	-76.3457	SALINITY	13	649	7/10/1984	12/12/2023	39.44932	-0.02554	-0.02013	-0.03554	17.48762
1169	CB4.4	38.41457	-76.3457	SALINITY	14	479	7/10/1984	12/12/2023	39.44932	-0.02203	-0.01164	-0.03011	17.62351
1169	CB4.4	38.41457	-76.3457	SALINITY	15	660	7/10/1984	12/12/2023	39.44932	-0.02025	-0.01645	-0.03063	18.31217
1169	CB4.4	38.41457	-76.3457	SALINITY	16	404	7/10/1984	12/12/2023	39.44932	-0.01833	-0.00274	-0.02329	18.39365
1169	CB4.4	38.41457	-76.3457	SALINITY	17	644	7/24/1984	12/12/2023	39.41096	-0.02091	-0.01452	-0.02814	18.97493
1169	CB4.4	38.41457	-76.3457	SALINITY	18	286	7/10/1984	12/12/2023	39.44932	-0.03586	-0.0185	-0.04169	18.79261
1169	CB4.4	38.41457	-76.3457	SALINITY	19	629	9/10/1984	12/12/2023	39.27945	-0.01533	-0.00958	-0.02396	19.29476
1169	CB4.4	38.41457	-76.3457	SALINITY	2	468	7/10/1984	12/12/2023	39.44932	0.035158	0.042869	0.015686	11.77153
1169	CB4.4	38.41457	-76.3457	SALINITY	20	155	9/10/1984	12/12/2023	39.27945	-0.00463	0.013737	-0.01454	18.48648
1169	CB4.4	38.41457	-76.3457	SALINITY	21	660	7/10/1984	12/12/2023	39.44932	-0.01948	-0.01381	-0.02699	19.74684
1169	CB4.4	38.41457	-76.3457	SALINITY	22	87	11/12/1985	8/7/2023	37.7589	0.026099	0.046611	-0.00531	18.18692
1169	CB4.4	38.41457	-76.3457	SALINITY	23	625	11/12/1985	12/12/2023	38.10685	-0.01822	-0.00729	-0.02185	19.86086
1169	CB4.4	38.41457	-76.3457	SALINITY	24	91	7/10/1984	7/27/2023	39.07123	-0.05975	-0.04781	-0.08312	20.08195
1169	CB4.4	38.41457	-76.3457	SALINITY	25	627	8/30/1984	12/12/2023	39.30959	-0.01242	-0.00378	-0.01762	19.97125
1169	CB4.4	38.41457	-76.3457	SALINITY	26	44	3/19/1985	7/27/2023	38.38082	0.00827	0.026246	-0.03348	18.65745
1169	CB4.4	38.41457	-76.3457	SALINITY	27	651	7/10/1984	12/12/2023	39.44932	-0.00997	-0.00441	-0.01793	20.04511
1169	CB4.4	38.41457	-76.3457	SALINITY	28	97	7/24/1984	4/10/2023	38.73699	-0.07909	-0.06234	-0.12711	21.07928
1169	CB4.4	38.41457	-76.3457	SALINITY	29	545	7/10/1984	12/12/2023	39.44932	0	0.016062	-0.00027	19.77324
1169	CB4.4	38.41457	-76.3457	SALINITY	3	660	7/10/1984	12/12/2023	39.44932	-0.04161	-0.02597	-0.04401	13.46463
1169	CB4.4	38.41457	-76.3457	SALINITY	30	129	1/14/1985	4/10/2023	38.26027	0.044281	0.065337	0.03357	18.6116
1169	CB4.4	38.41457	-76.3457	SALINITY	31	247	1/5/1987	12/12/2023	36.9589	0.043417	0.056803	0.018832	18.84989
1169	CB4.4	38.41457	-76.3457	SALINITY	32	46	2/10/1986	12/12/2023	37.86027	0.126913	0.154261	0.060252	14.91226
1169	CB4.4	38.41457	-76.3457	SALINITY	4	200	8/6/1984	12/12/2023	39.37534	0.025512	0.039765	0.000826	11.31044
1169	CB4.4	38.41457	-76.3457	SALINITY	5	635	8/6/1984	12/12/2023	39.37534	-0.03828	-0.02211	-0.04016	13.67959
1169	CB4.4	38.41457	-76.3457	SALINITY	6	342	7/10/1984	12/12/2023	39.44932	-0.01261	0.001256	-0.02545	12.96694
1169	CB4.4	38.41457	-76.3457	SALINITY	7	639	7/10/1984	12/12/2023	39.44932	-0.03615	-0.02092	-0.03778	14.34661
1169	CB4.4	38.41457	-76.3457	SALINITY	8	421	7/10/1984	12/12/2023	39.44932	-0.0113	-0.00476	-0.0259	13.698

1176	CB5.5	37.6918	-76.1897	SALINITY	19	134	8/30/1984	10/22/2013	29.16438	-0.00509	0.017615	-0.0538	21.41954
1176	CB5.5	37.6918	-76.1897	SALINITY	2	541	6/29/1984	2/20/2024	39.67123	-0.00518	0.002857	-0.01505	15.99148
1176	CB5.5	37.6918	-76.1897	SALINITY	20	45	11/26/1984	1/11/2007	22.13973	-0.02533	0.02848	-0.12214	22.0594
1176	CB5.5	37.6918	-76.1897	SALINITY	21	28	8/30/1984	9/19/2006	22.06849	0.157553	0.205926	-0.00031	19.94474
1176	CB5.5	37.6918	-76.1897	SALINITY	3	581	7/12/1984	2/20/2024	39.63562	0.078918	0.08957	0.071155	13.9036
1176	CB5.5	37.6918	-76.1897	SALINITY	4	545	6/29/1984	2/20/2024	39.67123	-0.0117	-0.00117	-0.01775	16.4879
1176	CB5.5	37.6918	-76.1897	SALINITY	5	581	7/12/1984	2/20/2024	39.63562	-0.01502	-0.00712	-0.02214	17.00499
1176	CB5.5	37.6918	-76.1897	SALINITY	6	543	6/29/1984	2/20/2024	39.67123	-0.01077	-0.00161	-0.01762	17.32925
1176	CB5.5	37.6918	-76.1897	SALINITY	7	581	7/12/1984	2/20/2024	39.63562	-0.00976	-0.00278	-0.01716	17.70046
1176	CB5.5	37.6918	-76.1897	SALINITY	8	543	6/29/1984	2/20/2024	39.67123	-0.00135	0.00725	-0.00873	17.90978
1176	CB5.5	37.6918	-76.1897	SALINITY	9	581	7/12/1984	2/20/2024	39.63562	-0.00544	0.002236	-0.01363	18.47083
1177	CB6.1	37.58847	-76.1622	SALINITY	0	24	6/7/1988	3/17/1992	3.778082	0.272743	0.656502	-2.19915	18.08281
1177	CB6.1	37.58847	-76.1622	SALINITY	1	599	7/12/1984	2/20/2024	39.63562	-0.01195	-0.00541	-0.02117	16.61867
1177	CB6.1	37.58847	-76.1622	SALINITY	10	556	6/27/1984	2/20/2024	39.67671	0.002877	0.008575	-0.00717	21.0336
1177	CB6.1	37.58847	-76.1622	SALINITY	11	606	7/12/1984	2/20/2024	39.63562	-0.00398	0.003991	-0.00924	21.73889
1177	CB6.1	37.58847	-76.1622	SALINITY	12	322	6/27/1984	2/20/2024	39.67671	-0.01298	-0.0066	-0.03031	21.98406
1177	CB6.1	37.58847	-76.1622	SALINITY	13	45	7/12/1984	9/19/2013	29.20822	-0.00221	0.066804	-0.09525	22.17943
1177	CB6.1	37.58847	-76.1622	SALINITY	2	556	6/27/1984	2/20/2024	39.67671	-0.00448	0.002791	-0.0142	16.56391
1177	CB6.1	37.58847	-76.1622	SALINITY	3	593	7/12/1984	2/20/2024	39.63562	0.084226	0.090025	0.07244	14.31618
1177	CB6.1	37.58847	-76.1622	SALINITY	4	556	6/27/1984	2/20/2024	39.67671	-0.00564	0.002914	-0.01316	16.98141
1177	CB6.1	37.58847	-76.1622	SALINITY	5	589	7/12/1984	2/20/2024	39.63562	-0.01575	-0.00747	-0.02276	17.43934
1177	CB6.1	37.58847	-76.1622	SALINITY	6	554	6/27/1984	2/20/2024	39.67671	-0.01131	0.000235	-0.01689	17.67891
1177	CB6.1	37.58847	-76.1622	SALINITY	7	593	7/12/1984	2/20/2024	39.63562	-0.01541	-0.00569	-0.02189	18.69633
1177	CB6.1	37.58847	-76.1622	SALINITY	8	560	6/27/1984	2/20/2024	39.67671	-0.00965	0.002708	-0.01423	19.40095
1177	CB6.1	37.58847	-76.1622	SALINITY	9	593	7/12/1984	2/20/2024	39.63562	-0.0107	-0.00402	-0.01957	20.53658
1178	CB6.2	37.4868	-76.1563	SALINITY	0	25	6/8/1988	3/18/1992	3.778082	-0.94019	0.641772	-2.81121	23.01139
1178	CB6.2	37.4868	-76.1563	SALINITY	1	594	7/11/1984	2/21/2024	39.6411	-0.01581	-0.01052	-0.02633	17.41462
1178	CB6.2	37.4868	-76.1563	SALINITY	10	330	6/27/1984	2/21/2024	39.67945	-0.02513	-0.01877	-0.04199	22.59942
1178	CB6.2	37.4868	-76.1563	SALINITY	11	46	8/7/1984	2/18/2020	35.55616	-0.11715	-0.05053	-0.26149	23.41772
1178	CB6.2	37.4868	-76.1563	SALINITY	2	551	6/27/1984	2/21/2024	39.67945	-0.00977	-0.00411	-0.02125	17.39698
1178	CB6.2	37.4868	-76.1563	SALINITY	3	588	7/11/1984	2/21/2024	39.6411	0.077249	0.08378	0.065378	15.01714
1178	CB6.2	37.4868	-76.1563	SALINITY	4	553	6/27/1984	2/21/2024	39.67945	-0.01678	-0.00765	-0.02474	17.75885
1178	CB6.2	37.4868	-76.1563	SALINITY	5	586	7/11/1984	2/21/2024	39.6411	-0.03549	-0.02378	-0.03978	18.44079
1178	CB6.2	37.4868	-76.1563	SALINITY	6	554	6/27/1984	2/21/2024	39.67945	-0.02907	-0.01515	-0.03341	19.10479
1178	CB6.2	37.4868	-76.1563	SALINITY	7	588	7/11/1984	2/21/2024	39.6411	-0.02461	-0.01633	-0.03416	20.59618
1178	CB6.2	37.4868	-76.1563	SALINITY	8	554	6/27/1984	2/21/2024	39.67945	-0.01837	-0.00571	-0.02414	21.35466
1178	CB6.2	37.4868	-76.1563	SALINITY	9	610	7/11/1984	2/21/2024	39.6411	-0.01346	-0.00413	-0.01894	21.94947
1179	CB6.3	37.41153	-76.1597	SALINITY	0	25	6/8/1988	3/18/1992	3.778082	-2.16007	-0.75019	-4.7047	31.25322
1179	CB6.3	37.41153	-76.1597	SALINITY	1	601	7/11/1984	2/21/2024	39.6411	-0.02181	-0.01147	-0.02727	18.03845
1179	CB6.3	37.41153	-76.1597	SALINITY	10	455	6/27/1984	1/18/2024	39.5863	0.014298	0.016443	0.001271	21.94681
1179	CB6.3	37.41153	-76.1597	SALINITY	11	348	8/29/1984	1/18/2024	39.4137	0.007969	0.008265	-0.01423	22.24307
1179	CB6.3	37.41153	-76.1597	SALINITY	12	142	7/11/1984	2/9/2021	36.60822	-0.05235	-0.04096	-0.09957	22.54061

1187	CB7.3E	37.22875	-76.0538	SALINITY	18.5	7	7/23/1984	3/13/1991	6.641096	-0.20735	0.223343	-0.77732	27.21282
1187	CB7.3E	37.22875	-76.0538	SALINITY	19	249	11/16/1984	2/20/2024	39.28767	-0.01493	-0.0059	-0.02999	27.05647
1187	CB7.3E	37.22875	-76.0538	SALINITY	2	554	10/28/1986	2/20/2024	37.33973	7.79E-05	0.012033	-0.00503	22.78874
1187	CB7.3E	37.22875	-76.0538	SALINITY	20	72	9/10/1985	1/22/2024	38.39178	-0.06239	-0.00315	-0.08519	28.99289
1187	CB7.3E	37.22875	-76.0538	SALINITY	21	92	11/16/1984	2/20/2024	39.28767	0.000947	0.014604	-0.02935	27.1472
1187	CB7.3E	37.22875	-76.0538	SALINITY	22	22	10/28/1986	10/14/2019	32.98356	0.019803	0.102352	-0.06605	26.88348
1187	CB7.3E	37.22875	-76.0538	SALINITY	22.5	3	3/5/1990	12/9/1991	1.764384	0.756958	2.074861	0.269053	22.31789
1187	CB7.3E	37.22875	-76.0538	SALINITY	23	25	3/26/1985	6/26/2018	33.27397	-0.1043	-0.06281	-0.14301	28.93919
1187	CB7.3E	37.22875	-76.0538	SALINITY	24	8	3/26/1985	8/12/2013	28.4	-0.1347	-0.05913	-0.27924	30.82948
1187	CB7.3E	37.22875	-76.0538	SALINITY	25	6	11/16/1987	7/24/2006	18.69863	0.095829	0.479812	-0.4417	28.07523
1187	CB7.3E	37.22875	-76.0538	SALINITY	3	598	8/7/1984	2/20/2024	39.56438	0.02978	0.041072	0.023886	22.68159
1187	CB7.3E	37.22875	-76.0538	SALINITY	4	554	10/28/1986	2/20/2024	37.33973	0.004982	0.01316	-0.00246	23.68372
1187	CB7.3E	37.22875	-76.0538	SALINITY	5	598	8/7/1984	2/20/2024	39.56438	-0.01037	0.000103	-0.01441	24.26286
1187	CB7.3E	37.22875	-76.0538	SALINITY	6	554	10/28/1986	2/20/2024	37.33973	-0.00701	0.003654	-0.01214	24.43823
1187	CB7.3E	37.22875	-76.0538	SALINITY	7	596	8/7/1984	2/20/2024	39.56438	-0.0174	-0.00688	-0.02087	24.84763
1187	CB7.3E	37.22875	-76.0538	SALINITY	8	552	10/28/1986	2/20/2024	37.33973	-0.01075	-0.00229	-0.01786	24.91301
1187	CB7.3E	37.22875	-76.0538	SALINITY	9	594	8/7/1984	2/20/2024	39.56438	-0.01897	-0.00887	-0.023	25.18894
1188	CB7.4	36.9957	-76.0205	SALINITY	1	673	6/27/1984	2/22/2024	39.68219	-0.06447	-0.04462	-0.06321	26.02373
1188	CB7.4	36.9957	-76.0205	SALINITY	10	544	3/25/1986	2/22/2024	37.93973	-0.00926	-0.00467	-0.01477	29.78944
1188	CB7.4	36.9957	-76.0205	SALINITY	11	576	8/7/1984	2/22/2024	39.56986	-0.00714	-0.00386	-0.01353	29.89087
1188	CB7.4	36.9957	-76.0205	SALINITY	11.5	4	9/23/1986	8/24/1992	5.923288	-0.08806	0.013272	-0.13506	30.79735
1188	CB7.4	36.9957	-76.0205	SALINITY	12	507	6/3/1985	2/22/2024	38.74795	-0.00261	-0.00058	-0.01107	29.92169
1188	CB7.4	36.9957	-76.0205	SALINITY	12.5	7	12/5/1988	7/25/1994	5.638356	0.264207	0.459492	-0.66528	29.2638
1188	CB7.4	36.9957	-76.0205	SALINITY	13	476	8/7/1984	2/22/2024	39.56986	0.003643	0.004956	-0.00553	29.87353
1188	CB7.4	36.9957	-76.0205	SALINITY	13.5	10	11/7/1988	8/10/1992	3.758904	0.368936	0.656964	-0.36931	28.37253
1188	CB7.4	36.9957	-76.0205	SALINITY	14	315	8/27/1984	2/22/2024	39.51507	0.017633	0.020122	0.007048	29.55702
1188	CB7.4	36.9957	-76.0205	SALINITY	14.5	5	1/24/1985	7/20/1992	7.490411	-0.2048	7.494927	-0.38026	31.51313
1188	CB7.4	36.9957	-76.0205	SALINITY	15	96	3/26/1985	2/22/2024	38.93699	-0.01231	-0.00576	-0.03389	30.45072
1188	CB7.4	36.9957	-76.0205	SALINITY	16	7	3/22/1988	12/10/2007	19.73151	-0.32722	0.118961	-0.58881	34.88537
1188	CB7.4	36.9957	-76.0205	SALINITY	2	545	10/28/1986	2/22/2024	37.34521	-0.04071	-0.02591	-0.04657	26.50926
1188	CB7.4	36.9957	-76.0205	SALINITY	3	590	8/7/1984	2/22/2024	39.56986	-0.00297	0.017399	-0.00217	26.6484
1188	CB7.4	36.9957	-76.0205	SALINITY	4	546	8/11/1986	2/22/2024	37.5589	-0.03591	-0.02263	-0.03998	28.06311
1188	CB7.4	36.9957	-76.0205	SALINITY	5	588	8/7/1984	2/22/2024	39.56986	-0.0275	-0.02128	-0.03529	28.62973
1188	CB7.4	36.9957	-76.0205	SALINITY	6	545	10/28/1986	2/22/2024	37.34521	-0.02162	-0.01373	-0.02784	28.87389
1188	CB7.4	36.9957	-76.0205	SALINITY	7	590	8/7/1984	2/22/2024	39.56986	-0.01702	-0.01219	-0.02341	29.21929
1188	CB7.4	36.9957	-76.0205	SALINITY	8	545	10/28/1986	2/22/2024	37.34521	-0.01391	-0.00973	-0.0209	29.5414
1188	CB7.4	36.9957	-76.0205	SALINITY	9	586	8/7/1984	2/22/2024	39.56986	-0.01327	-0.01008	-0.02057	29.71228
1189	CB7.4N	37.06237	-75.9994	SALINITY	1	592	6/27/1984	2/22/2024	39.68219	-0.01917	-0.0073	-0.02604	27.95411
1189	CB7.4N	37.06237	-75.9994	SALINITY	10	460	9/10/1985	2/22/2024	38.47671	-0.00066	0.008551	-0.00691	29.70259
1189	CB7.4N	37.06237	-75.9994	SALINITY	10.5	3	4/25/1988	1/16/1989	0.728767	4.597917	10.61953	-2.72838	13.57771
1189	CB7.4N	37.06237	-75.9994	SALINITY	11	300	8/7/1984	5/29/2020	35.83288	0.000954	0.016356	-0.01157	29.80249
1189	CB7.4N	37.06237	-75.9994	SALINITY	11.5	5	6/27/1984	8/24/1992	8.164384	-0.27447	0.08605	-4.66632	27.11539

1231	EE1.1	38.88	-76.2515	SALINITY	11.5	69	10/9/1984	7/12/2023	38.78082	-0.00818	0.022905	-0.01945	15.20265
1231	EE1.1	38.88	-76.2515	SALINITY	11.6	37	12/10/1985	3/15/2022	36.28493	0.058835	0.092683	0.011299	13.49798
1231	EE1.1	38.88	-76.2515	SALINITY	11.7	53	8/7/1985	8/10/2023	38.03288	-0.02874	0.01806	-0.04072	14.85807
1231	EE1.1	38.88	-76.2515	SALINITY	11.8	50	6/19/1985	11/13/2023	38.4274	-0.0245	0.011901	-0.07054	14.76773
1231	EE1.1	38.88	-76.2515	SALINITY	11.9	35	5/5/1987	6/8/2023	36.11781	-0.02278	0.042464	-0.04061	14.38035
1231	EE1.1	38.88	-76.2515	SALINITY	12	227	10/23/1984	3/13/2023	38.41096	-0.01603	0.006455	-0.02516	15.21899
1231	EE1.1	38.88	-76.2515	SALINITY	12.1	18	8/20/1985	5/16/2022	36.76164	-0.12509	-0.10062	-0.18636	17.19832
1231	EE1.1	38.88	-76.2515	SALINITY	12.2	24	9/23/1986	10/12/2022	36.07671	0.096154	0.151524	0.024743	12.82374
1231	EE1.1	38.88	-76.2515	SALINITY	12.3	15	7/29/1986	7/7/2020	33.96438	-0.02452	-0.00665	-0.05691	15.46682
1231	EE1.1	38.88	-76.2515	SALINITY	12.4	13	3/9/1987	3/13/2023	36.03562	-0.08742	0.134342	-0.12028	15.18946
1231	EE1.1	38.88	-76.2515	SALINITY	12.5	9	10/23/1984	6/24/2010	25.68493	0.063307	0.185793	-0.02962	14.48207
1231	EE1.1	38.88	-76.2515	SALINITY	12.6	4	6/25/1997	7/11/2017	20.05753	-0.05875	-0.04351	-0.074	15.27571
1231	EE1.1	38.88	-76.2515	SALINITY	12.7	6	8/5/2003	7/26/2005	1.975342	2.493169	4.01132	2.003902	-38.8037
1231	EE1.1	38.88	-76.2515	SALINITY	13	13	11/15/1984	12/16/2014	30.10411	0.07442	0.152837	0.043193	13.71892
1231	EE1.1	38.88	-76.2515	SALINITY	2	572	10/9/1984	12/14/2023	39.20548	-0.03168	-0.01637	-0.03435	12.77394
1231	EE1.1	38.88	-76.2515	SALINITY	3	587	10/9/1984	12/14/2023	39.20548	-0.03123	-0.01751	-0.0353	12.81839
1231	EE1.1	38.88	-76.2515	SALINITY	4	526	10/9/1984	12/14/2023	39.20548	-0.03413	-0.01801	-0.03638	12.71065
1231	EE1.1	38.88	-76.2515	SALINITY	5	579	10/9/1984	12/14/2023	39.20548	-0.02639	-0.01316	-0.03092	12.83501
1231	EE1.1	38.88	-76.2515	SALINITY	6	531	10/9/1984	12/14/2023	39.20548	-0.03287	-0.01828	-0.03621	12.84564
1231	EE1.1	38.88	-76.2515	SALINITY	7	579	10/9/1984	12/14/2023	39.20548	-0.02149	-0.01074	-0.02776	13.11729
1231	EE1.1	38.88	-76.2515	SALINITY	8	534	10/9/1984	12/14/2023	39.20548	-0.01968	-0.01151	-0.02988	13.39553
1231	EE1.1	38.88	-76.2515	SALINITY	9	582	10/9/1984	12/14/2023	39.20548	-0.00787	-0.00277	-0.02093	13.76274
1232	EE2.1	38.6549	-76.2643	SALINITY	0.3	15	4/23/1985	12/10/1985	0.632877	8.922139	9.600614	7.835268	5.458483
1232	EE2.1	38.6549	-76.2643	SALINITY	0.5	568	8/2/1984	12/13/2023	39.38904	-0.02046	-0.00211	-0.02074	12.49023
1232	EE2.1	38.6549	-76.2643	SALINITY	1	577	8/2/1984	12/13/2023	39.38904	-0.02484	-0.00909	-0.02702	12.67263
1232	EE2.1	38.6549	-76.2643	SALINITY	2	576	8/2/1984	12/13/2023	39.38904	-0.02353	-0.00874	-0.02702	12.74104
1232	EE2.1	38.6549	-76.2643	SALINITY	3	577	8/2/1984	12/13/2023	39.38904	-0.02509	-0.00902	-0.02738	12.86117
1232	EE2.1	38.6549	-76.2643	SALINITY	4	575	8/2/1984	12/13/2023	39.38904	-0.02427	-0.00843	-0.02715	12.92683
1232	EE2.1	38.6549	-76.2643	SALINITY	5	575	8/2/1984	12/13/2023	39.38904	-0.02336	-0.0079	-0.02566	13.09976
1232	EE2.1	38.6549	-76.2643	SALINITY	5.9	4	9/6/1995	3/12/2002	6.517808	0.183748	0.856676	-0.13331	12.71252
1232	EE2.1	38.6549	-76.2643	SALINITY	6	549	8/2/1984	12/13/2023	39.38904	-0.02539	-0.01005	-0.02773	13.23413
1232	EE2.1	38.6549	-76.2643	SALINITY	6.1	10	8/10/1993	10/16/2012	19.19726	0.033362	0.058334	-0.23741	13.47394
1232	EE2.1	38.6549	-76.2643	SALINITY	6.2	16	4/4/1989	4/27/1999	10.06849	-0.43934	-0.24248	-0.74624	15.78307
1232	EE2.1	38.6549	-76.2643	SALINITY	6.3	20	3/25/1986	5/14/2013	27.15616	-0.09574	-0.0667	-0.30486	12.15607
1232	EE2.1	38.6549	-76.2643	SALINITY	6.4	22	10/6/1987	3/15/2022	34.46301	-0.01471	0.052318	-0.09177	12.96073
1232	EE2.1	38.6549	-76.2643	SALINITY	6.5	48	8/23/1984	6/13/2017	32.8274	-0.00584	0.037436	-0.09086	14.41914
1232	EE2.1	38.6549	-76.2643	SALINITY	6.6	48	6/4/1985	8/4/2015	30.1863	-0.03056	0.010178	-0.07673	12.60483
1232	EE2.1	38.6549	-76.2643	SALINITY	6.7	50	7/23/1985	10/16/2018	33.25479	-0.05923	-0.00812	-0.10514	14.13014
1232	EE2.1	38.6549	-76.2643	SALINITY	6.8	57	5/8/1985	9/12/2023	38.3726	0.0356	0.062108	0.013155	12.76492
1232	EE2.1	38.6549	-76.2643	SALINITY	6.9	39	7/9/1985	10/17/2023	38.29863	-0.0421	0.019985	-0.07607	14.11741
1232	EE2.1	38.6549	-76.2643	SALINITY	7	266	8/2/1984	12/13/2023	39.38904	-0.01384	-0.00707	-0.03469	13.97955
1232	EE2.1	38.6549	-76.2643	SALINITY	7.1	24	5/13/1986	4/13/2021	34.94247	-0.06123	-0.0025	-0.09714	14.09342

1235	EE3.1	38.19685	-75.9732	SALINITY	6	519	10/17/1984	12/13/2023	39.18082	-0.02262	-0.01449	-0.02887	15.46416
1235	EE3.1	38.19685	-75.9732	SALINITY	7	556	10/17/1984	12/13/2023	39.18082	-0.01794	-0.01232	-0.0267	15.54952
1235	EE3.1	38.19685	-75.9732	SALINITY	8	520	10/17/1984	12/13/2023	39.18082	-0.02266	-0.0149	-0.02984	15.75041
1235	EE3.1	38.19685	-75.9732	SALINITY	9	561	10/17/1984	12/13/2023	39.18082	-0.01726	-0.01225	-0.02637	15.69594
1236	EE3.2	37.98139	-75.9242	SALINITY	0.5	532	3/11/1986	12/12/2023	37.78082	-0.01036	-0.00095	-0.01637	16.74762
1236	EE3.2	37.98139	-75.9242	SALINITY	1	528	3/11/1986	12/12/2023	37.78082	-0.01003	-0.00093	-0.01661	16.73383
1236	EE3.2	37.98139	-75.9242	SALINITY	10	39	4/8/1986	10/21/2022	36.56164	0.007782	0.040669	-0.01959	17.16333
1236	EE3.2	37.98139	-75.9242	SALINITY	11	526	3/11/1986	12/12/2023	37.78082	-0.00529	0.000649	-0.01471	17.23779
1236	EE3.2	37.98139	-75.9242	SALINITY	12	38	4/8/1986	10/21/2022	36.56164	0.030929	0.066846	-0.01181	17.54213
1236	EE3.2	37.98139	-75.9242	SALINITY	13	527	3/11/1986	12/12/2023	37.78082	-0.00782	-0.0005	-0.01644	17.3367
1236	EE3.2	37.98139	-75.9242	SALINITY	14	35	4/8/1986	10/21/2022	36.56164	0.022376	0.074582	-0.00637	18.01484
1236	EE3.2	37.98139	-75.9242	SALINITY	15	527	3/11/1986	12/12/2023	37.78082	-0.00456	0.00074	-0.0147	17.45
1236	EE3.2	37.98139	-75.9242	SALINITY	16	41	4/8/1986	10/21/2022	36.56164	0.00289	0.057022	-0.01969	18.2925
1236	EE3.2	37.98139	-75.9242	SALINITY	17	525	4/8/1986	12/12/2023	37.70411	-0.00593	0.001829	-0.01368	17.60466
1236	EE3.2	37.98139	-75.9242	SALINITY	18	411	4/8/1986	12/12/2023	37.70411	-0.00895	0.003046	-0.01556	17.79132
1236	EE3.2	37.98139	-75.9242	SALINITY	19	522	4/8/1986	12/12/2023	37.70411	-0.00433	0.002769	-0.01327	17.6727
1236	EE3.2	37.98139	-75.9242	SALINITY	2	386	3/11/1986	12/12/2023	37.78082	-0.00091	0.002832	-0.01333	16.47359
1236	EE3.2	37.98139	-75.9242	SALINITY	20	29	3/11/1986	10/21/2022	36.63836	0.072473	0.140159	0.031203	18.36236
1236	EE3.2	37.98139	-75.9242	SALINITY	21	521	4/30/1986	12/12/2023	37.64384	-0.00768	0.001117	-0.01542	17.82745
1236	EE3.2	37.98139	-75.9242	SALINITY	22	30	4/30/1986	10/21/2022	36.50137	0.012627	0.087987	-0.0108	18.54333
1236	EE3.2	37.98139	-75.9242	SALINITY	23	515	4/30/1986	12/12/2023	37.64384	-0.00759	0.001507	-0.01507	17.88258
1236	EE3.2	37.98139	-75.9242	SALINITY	23.5	5	6/24/1986	11/23/1992	6.421918	2.398083	2.562374	1.117422	16.16288
1236	EE3.2	37.98139	-75.9242	SALINITY	23.7	4	9/8/1986	5/17/2018	31.70959	0.017946	0.027166	-0.07157	15.12638
1236	EE3.2	37.98139	-75.9242	SALINITY	24	32	4/30/1986	10/21/2022	36.50137	-0.0561	-0.00776	-0.13568	19.13407
1236	EE3.2	37.98139	-75.9242	SALINITY	24.5	3	6/15/1987	8/20/1998	11.18904	1.061221	1.238748	-0.0676	2.589258
1236	EE3.2	37.98139	-75.9242	SALINITY	24.7	3	6/9/1986	11/18/1997	11.45205	0.120668	0.121314	0.119856	18.17095
1236	EE3.2	37.98139	-75.9242	SALINITY	24.8	3	5/16/1990	10/12/1993	3.410959	1.41955	1.702915	0.86294	10.63777
1236	EE3.2	37.98139	-75.9242	SALINITY	25	463	4/30/1986	12/12/2023	37.64384	-0.00703	0.003955	-0.01287	17.87543
1236	EE3.2	37.98139	-75.9242	SALINITY	25.2	4	12/12/1990	9/29/2010	19.81096	0.24765	0.371727	0.042105	13.85526
1236	EE3.2	37.98139	-75.9242	SALINITY	25.3	9	6/6/1988	4/14/2004	15.86575	0.048118	0.246425	-0.19606	17.4309
1236	EE3.2	37.98139	-75.9242	SALINITY	25.4	4	5/22/1991	5/31/2001	10.03288	0.835494	0.946026	0.330843	5.683028
1236	EE3.2	37.98139	-75.9242	SALINITY	25.5	15	7/18/1990	11/14/2019	29.34521	0.086585	0.204758	-0.01634	15.79698
1236	EE3.2	37.98139	-75.9242	SALINITY	25.6	9	7/6/1987	5/6/2015	27.85205	-0.07949	0.051829	-0.12359	18.75592
1236	EE3.2	37.98139	-75.9242	SALINITY	25.7	16	12/14/1989	11/15/2023	33.94247	0.205664	0.267841	0.091319	16.70324
1236	EE3.2	37.98139	-75.9242	SALINITY	25.8	11	9/1/1988	8/14/2019	30.96986	-0.07161	-0.00496	-0.12188	18.77826
1236	EE3.2	37.98139	-75.9242	SALINITY	25.9	8	6/14/1989	10/17/2013	24.3589	0.250798	0.320566	0.048548	15.63583
1236	EE3.2	37.98139	-75.9242	SALINITY	26	85	4/30/1986	3/16/2023	36.90137	-0.04077	-0.01657	-0.06002	18.4045
1236	EE3.2	37.98139	-75.9242	SALINITY	26.1	15	8/29/1990	10/18/2023	33.1589	-0.01283	0.012793	-0.0423	18.0007
1236	EE3.2	37.98139	-75.9242	SALINITY	26.2	27	4/30/1986	9/12/2023	37.39452	0.011753	0.046231	-0.04594	17.56206
1236	EE3.2	37.98139	-75.9242	SALINITY	26.3	31	8/30/1989	9/20/2023	34.07945	-0.01011	0.032792	-0.05567	17.87797
1236	EE3.2	37.98139	-75.9242	SALINITY	26.4	25	12/8/1986	12/12/2023	37.03562	-0.0791	-0.0219	-0.11122	18.71961
1236	EE3.2	37.98139	-75.9242	SALINITY	26.5	43	2/24/1987	6/29/2023	36.36712	0.032874	0.099482	0.009728	15.25714

1246	ET1.1	39.56976	-75.9678	SALINITY	0.5	423	4/10/1986	12/13/2023	37.70137	0	0.006604	-0.01494	0
1246	ET1.1	39.56976	-75.9678	SALINITY	1	414	10/29/1985	12/13/2023	38.14795	0	0.003689	-0.01687	0
1246	ET1.1	39.56976	-75.9678	SALINITY	1.1	4	3/17/1988	3/16/2023	35.01918	0	NA	NA	0
1246	ET1.1	39.56976	-75.9678	SALINITY	1.2	5	5/12/1988	2/25/2014	25.80822	0	NA	NA	0
1246	ET1.1	39.56976	-75.9678	SALINITY	1.3	12	4/9/1987	8/11/2016	29.36164	0	NA	NA	0
1246	ET1.1	39.56976	-75.9678	SALINITY	1.4	24	7/31/1986	6/15/2017	30.89589	0	0.0016	-0.06447	0
1246	ET1.1	39.56976	-75.9678	SALINITY	1.5	46	8/14/1986	8/11/2023	37.01644	0	0.07024	-0.03811	0
1246	ET1.1	39.56976	-75.9678	SALINITY	1.6	40	11/18/1987	12/13/2023	36.09315	0	0.040787	-0.06413	0
1246	ET1.1	39.56976	-75.9678	SALINITY	1.7	38	4/10/1986	3/18/2021	34.96164	0	0.036067	-0.0387	0
1246	ET1.1	39.56976	-75.9678	SALINITY	1.8	42	10/29/1985	6/15/2023	37.65205	0	0.093646	-0.01425	0
1246	ET1.1	39.56976	-75.9678	SALINITY	1.9	40	10/8/1987	11/14/2023	36.12603	0	0.085608	-0.12114	0
1246	ET1.1	39.56976	-75.9678	SALINITY	2	140	11/20/1985	6/9/2022	36.57534	0	0.024822	-0.04662	0
1246	ET1.1	39.56976	-75.9678	SALINITY	2.1	29	3/12/1987	10/19/2023	36.63014	0	0.016984	-0.03931	0
1246	ET1.1	39.56976	-75.9678	SALINITY	2.2	33	12/12/1985	4/20/2022	36.37808	0	0.022601	-0.03588	0
1246	ET1.1	39.56976	-75.9678	SALINITY	2.3	21	5/15/1986	5/19/2022	36.03562	0	NA	NA	0
1246	ET1.1	39.56976	-75.9678	SALINITY	2.4	21	11/20/1985	6/9/2022	36.57534	0	0.000162	-0.17355	0
1246	ET1.1	39.56976	-75.9678	SALINITY	2.5	25	1/7/1987	9/18/2018	31.71781	0	0.016045	0.016045	0
1246	ET1.1	39.56976	-75.9678	SALINITY	2.6	7	3/23/1995	9/20/2017	22.51233	0	NA	NA	0
1246	ET1.1	39.56976	-75.9678	SALINITY	2.7	6	5/14/1992	5/7/2009	16.99178	0	NA	NA	0
1246	ET1.1	39.56976	-75.9678	SALINITY	2.8	6	5/22/2007	7/9/2009	2.134247	0	0.735944	0.735944	0
1246	ET1.1	39.56976	-75.9678	SALINITY	2.9	3	6/11/1992	9/16/2020	28.28493	0	NA	NA	0
1246	ET1.1	39.56976	-75.9678	SALINITY	3	7	6/27/1990	8/13/2019	29.14795	0	NA	NA	0
1247	ET10.1	38.07614	-75.5713	SALINITY	0	5	12/16/2003	2/14/2007	3.167123	0	NA	NA	0
1247	ET10.1	38.07614	-75.5713	SALINITY	0.5	447	1/8/1986	12/13/2023	37.95342	0	0.018894	-0.02956	0
1247	ET10.1	38.07614	-75.5713	SALINITY	1	439	3/24/1986	12/13/2023	37.74795	0	0.017151	-0.03432	0
1247	ET10.1	38.07614	-75.5713	SALINITY	2	438	3/24/1986	12/13/2023	37.74795	0	0.017429	-0.03421	0
1247	ET10.1	38.07614	-75.5713	SALINITY	2.9	6	7/14/1993	3/16/2016	22.68767	0	NA	NA	0
1247	ET10.1	38.07614	-75.5713	SALINITY	3	424	3/24/1986	12/13/2023	37.74795	0	0.017442	-0.03429	0
1247	ET10.1	38.07614	-75.5713	SALINITY	3.2	6	7/18/1990	6/8/2023	32.91233	0	NA	NA	0
1247	ET10.1	38.07614	-75.5713	SALINITY	3.4	6	9/14/1987	7/15/2013	25.85205	0	-0.0461	-0.0461	0
1247	ET10.1	38.07614	-75.5713	SALINITY	3.5	15	12/8/1986	11/16/2016	29.96164	0	NA	NA	0
1247	ET10.1	38.07614	-75.5713	SALINITY	3.6	5	3/7/1990	12/16/2003	13.7863	0	NA	NA	0
1247	ET10.1	38.07614	-75.5713	SALINITY	3.7	3	7/12/1989	7/13/1994	5.005479	0	NA	NA	0
1247	ET10.1	38.07614	-75.5713	SALINITY	3.8	3	10/19/1987	9/19/2007	19.93151	-0.24965	0.08485	-0.31844	1.292696
1247	ET10.1	38.07614	-75.5713	SALINITY	3.9	6	11/17/1986	8/11/1993	6.736986	0	NA	NA	0
1247	ET10.1	38.07614	-75.5713	SALINITY	4	369	1/8/1986	12/13/2023	37.95342	0	0.024451	-0.03321	0
1247	ET10.1	38.07614	-75.5713	SALINITY	4.1	6	8/7/1991	10/21/2015	24.22192	0	NA	NA	0
1247	ET10.1	38.07614	-75.5713	SALINITY	4.2	3	1/19/1989	2/11/1993	4.065753	0	NA	NA	0
1247	ET10.1	38.07614	-75.5713	SALINITY	4.4	3	2/14/1991	10/18/2017	26.69315	0	NA	NA	0
1247	ET10.1	38.07614	-75.5713	SALINITY	4.5	7	5/16/1990	12/11/2013	23.58904	0	NA	NA	0
1247	ET10.1	38.07614	-75.5713	SALINITY	4.6	6	11/6/1991	5/10/2017	25.52603	0	NA	NA	0
1247	ET10.1	38.07614	-75.5713	SALINITY	4.8	7	9/20/1995	7/8/2015	19.81096	0	NA	NA	0

1252	ET4.1	39.2437	-75.9249	SALINITY	5	163	3/5/1985	12/11/2023	38.79452	0	-0.00361	-0.0188	0
1252	ET4.1	39.2437	-75.9249	SALINITY	5.1	17	7/10/1985	11/9/2022	37.3589	0	0.049391	-0.03093	0
1252	ET4.1	39.2437	-75.9249	SALINITY	5.2	15	9/25/1996	12/11/2023	27.2274	-0.03107	-0.01475	-0.07726	1.422302
1252	ET4.1	39.2437	-75.9249	SALINITY	5.3	13	1/8/1991	12/6/2021	30.93151	0	0.03075	-0.12426	0
1252	ET4.1	39.2437	-75.9249	SALINITY	5.4	21	4/25/1985	5/10/2022	37.06575	-0.01866	-0.01744	-0.05121	0.695056
1252	ET4.1	39.2437	-75.9249	SALINITY	5.5	9	7/22/1985	6/3/2015	29.88493	-0.03448	-0.00788	-0.06346	1.173708
1252	ET4.1	39.2437	-75.9249	SALINITY	5.6	9	8/29/1990	8/12/2019	28.9726	0	0.1314	-0.06628	0
1252	ET4.1	39.2437	-75.9249	SALINITY	5.7	14	6/16/2004	2/13/2023	18.67397	0	0.072137	-0.04081	0
1252	ET4.1	39.2437	-75.9249	SALINITY	5.8	9	10/23/1996	7/13/2023	26.73699	0.044349	0.080316	0.030887	-0.72281
1252	ET4.1	39.2437	-75.9249	SALINITY	5.9	5	6/5/1985	5/12/2021	35.9589	-0.03338	-0.01682	-0.95906	1.175278
1252	ET4.1	39.2437	-75.9249	SALINITY	6	7	5/21/1991	7/13/2021	30.16712	0.002472	0.010095	0.002525	0
1253	ET4.2	38.99233	-76.2151	SALINITY	0.3	19	2/19/1985	12/11/1985	0.808219	8.895152	9.600036	5.992232	4.867808
1253	ET4.2	38.99233	-76.2151	SALINITY	0.5	589	10/16/1984	12/14/2023	39.1863	-0.03291	-0.01516	-0.03484	9.786585
1253	ET4.2	38.99233	-76.2151	SALINITY	1	594	10/16/1984	12/14/2023	39.1863	-0.04015	-0.02466	-0.04317	10.13172
1253	ET4.2	38.99233	-76.2151	SALINITY	10	540	10/16/1984	12/14/2023	39.1863	-0.01945	-0.00824	-0.02685	11.97231
1253	ET4.2	38.99233	-76.2151	SALINITY	10.1	5	4/8/1986	5/15/2023	37.12603	-0.16113	-0.05848	-0.41132	18.42015
1253	ET4.2	38.99233	-76.2151	SALINITY	10.3	8	8/9/1988	12/14/2023	35.36986	0.020814	0.109817	-0.19168	10.39092
1253	ET4.2	38.99233	-76.2151	SALINITY	10.4	7	8/8/1985	4/18/2011	25.70959	-0.15917	-0.1486	-0.2093	12.70221
1253	ET4.2	38.99233	-76.2151	SALINITY	10.5	11	9/11/1985	12/20/2022	37.29863	0.008812	0.089097	-0.06049	11.23005
1253	ET4.2	38.99233	-76.2151	SALINITY	10.6	7	2/20/1986	3/23/2017	31.10685	-0.1333	-0.11134	-0.1333	15.01633
1253	ET4.2	38.99233	-76.2151	SALINITY	10.7	6	9/25/1985	3/19/2018	32.50137	0.046368	0.133868	-0.08002	14.33215
1253	ET4.2	38.99233	-76.2151	SALINITY	10.8	12	7/15/1986	6/9/2020	33.92603	-0.23514	-0.09039	-0.39454	16.59408
1253	ET4.2	38.99233	-76.2151	SALINITY	11	483	11/14/1984	8/7/2023	38.75342	-0.01149	-0.00359	-0.02657	12.21967
1253	ET4.2	38.99233	-76.2151	SALINITY	11.1	12	10/15/1997	3/13/2023	25.42466	-0.0132	0.266747	-0.15843	13.53172
1253	ET4.2	38.99233	-76.2151	SALINITY	11.2	26	11/18/1986	4/7/2022	35.40822	-0.11305	-0.08793	-0.14503	14.6903
1253	ET4.2	38.99233	-76.2151	SALINITY	11.3	14	7/7/1987	6/10/2021	33.95068	0.01286	0.057716	-0.05198	10.11
1253	ET4.2	38.99233	-76.2151	SALINITY	11.4	13	3/11/1993	6/12/2019	26.27123	-0.27666	-0.15543	-0.35237	18.28378
1253	ET4.2	38.99233	-76.2151	SALINITY	11.5	25	2/25/1987	8/7/2023	36.47123	-0.01843	0.031343	-0.06717	12.5153
1253	ET4.2	38.99233	-76.2151	SALINITY	11.6	20	3/11/1986	3/19/2013	27.0411	-0.0648	0.002138	-0.17175	13.40043
1253	ET4.2	38.99233	-76.2151	SALINITY	11.7	17	10/22/1985	6/7/2022	36.64932	-0.07849	-0.04003	-0.1611	12.40818
1253	ET4.2	38.99233	-76.2151	SALINITY	11.8	12	10/18/1995	1/20/2016	20.27123	-0.68968	0.015927	-1.49807	18.80283
1253	ET4.2	38.99233	-76.2151	SALINITY	11.9	8	6/21/1988	4/13/2021	32.83288	-0.09284	0.046103	-0.19442	11.38784
1253	ET4.2	38.99233	-76.2151	SALINITY	12	301	5/7/1985	8/13/2020	35.29315	-1.43E-05	0.018294	-0.02023	12.4097
1253	ET4.2	38.99233	-76.2151	SALINITY	12.1	6	9/15/1998	9/14/2022	24.0137	0.166103	0.238061	-0.071	3.916088
1253	ET4.2	38.99233	-76.2151	SALINITY	12.2	12	8/6/1996	4/7/2015	18.67945	0.364786	0.539978	0.016691	6.945789
1253	ET4.2	38.99233	-76.2151	SALINITY	12.3	8	12/5/1991	8/24/2010	18.73151	-0.29061	0.122708	-0.41487	12.70029
1253	ET4.2	38.99233	-76.2151	SALINITY	12.4	4	3/9/1987	7/21/2005	18.38082	-0.67723	-0.34272	-0.75239	14.16287
1253	ET4.2	38.99233	-76.2151	SALINITY	12.5	16	10/11/1989	9/16/2014	24.94795	-0.05479	0.033957	-0.13537	12.48481
1253	ET4.2	38.99233	-76.2151	SALINITY	12.6	7	11/17/1987	6/2/2015	27.5589	-0.16778	0.108346	-0.3974	12.857
1253	ET4.2	38.99233	-76.2151	SALINITY	12.7	14	11/15/1988	9/15/2015	26.84932	-0.06177	0.120164	-0.15699	11.32349
1253	ET4.2	38.99233	-76.2151	SALINITY	12.8	11	8/14/1989	5/15/2012	22.76712	-0.24053	-0.22066	-0.44209	19.01679
1253	ET4.2	38.99233	-76.2151	SALINITY	13	238	3/25/1987	8/13/2020	33.41096	0.019342	0.033788	-0.00981	12.78939

1258	ET6.1	38.54833	-75.7031	SALINITY	5	16	1/8/1986	5/10/2017	31.35616	0	0.024797	-0.10681	0
1258	ET6.1	38.54833	-75.7031	SALINITY	6	4	9/8/1986	6/15/1994	7.772603	-1.63176	-0.86595	-1.88051	2.159294
1259	ET6.2	38.34133	-75.8883	SALINITY	0.5	453	1/8/1986	12/13/2023	37.95342	-0.02068	-0.0122	-0.03899	8.28226
1259	ET6.2	38.34133	-75.8883	SALINITY	1	453	1/8/1986	12/13/2023	37.95342	-0.01941	-0.01076	-0.03736	8.348873
1259	ET6.2	38.34133	-75.8883	SALINITY	2	440	1/8/1986	12/13/2023	37.95342	-0.02082	-0.01244	-0.03958	8.570125
1259	ET6.2	38.34133	-75.8883	SALINITY	2.1	6	5/10/2005	12/13/2023	18.60548	-0.06285	0.29858	-0.3834	12.7038
1259	ET6.2	38.34133	-75.8883	SALINITY	2.2	5	8/17/1994	5/18/2022	27.76986	-0.0578	0.026891	-0.17303	8.121697
1259	ET6.2	38.34133	-75.8883	SALINITY	2.3	16	6/10/1986	7/15/2021	35.12055	-0.1397	-0.00135	-0.18993	8.296746
1259	ET6.2	38.34133	-75.8883	SALINITY	2.4	12	7/26/1989	8/12/2021	32.06849	-0.10019	-0.03736	-0.25331	12.92333
1259	ET6.2	38.34133	-75.8883	SALINITY	2.5	34	3/10/1986	8/10/2022	36.44384	-0.08402	0.011747	-0.11149	9.500705
1259	ET6.2	38.34133	-75.8883	SALINITY	2.6	18	4/7/1986	4/12/2023	37.03836	-0.0467	0.009826	-0.14353	7.644626
1259	ET6.2	38.34133	-75.8883	SALINITY	2.7	33	5/13/1986	11/15/2023	37.53425	0.008755	0.090691	-0.03096	9.689952
1259	ET6.2	38.34133	-75.8883	SALINITY	2.8	36	11/18/1986	10/18/2023	36.93973	-0.00412	0.091628	-0.02225	9.340897
1259	ET6.2	38.34133	-75.8883	SALINITY	2.9	35	12/13/1990	9/14/2022	31.77534	-0.07273	-0.01697	-0.11458	8.741907
1259	ET6.2	38.34133	-75.8883	SALINITY	3	194	1/8/1986	5/18/2023	37.38082	-0.06435	-0.04697	-0.08863	11.23429
1259	ET6.2	38.34133	-75.8883	SALINITY	3.1	19	8/4/1987	6/13/2018	30.87945	-0.08755	-0.04087	-0.17388	10.07307
1259	ET6.2	38.34133	-75.8883	SALINITY	3.2	31	10/20/1987	6/15/2023	35.67671	0.066305	0.109428	-0.00532	6.050097
1259	ET6.2	38.34133	-75.8883	SALINITY	3.3	19	9/13/1990	7/14/2022	31.85479	-0.18132	-0.13812	-0.2312	14.02024
1259	ET6.2	38.34133	-75.8883	SALINITY	3.4	9	11/18/1987	8/6/2012	24.73425	-0.09483	0.182172	-0.51523	13.86207
1259	ET6.2	38.34133	-75.8883	SALINITY	3.5	18	1/5/1987	9/16/2020	33.72055	-0.03954	0.005289	-0.11466	12.42983
1259	ET6.2	38.34133	-75.8883	SALINITY	3.6	3	4/15/1993	5/15/2019	26.09863	-0.22673	-0.02901	-0.39121	15.30869
1259	ET6.2	38.34133	-75.8883	SALINITY	3.7	5	10/23/2008	9/20/2018	9.915068	-0.90788	6.214463	-1.70352	34.74952
1259	ET6.2	38.34133	-75.8883	SALINITY	3.8	3	12/15/1994	3/19/2008	13.26849	-0.29263	-0.2178	-0.30394	17.59892
1259	ET6.2	38.34133	-75.8883	SALINITY	4	4	2/11/1998	6/19/2014	16.36164	0.335269	0.417623	0.24257	0.18279
1260	ET7.1	38.26783	-75.7879	SALINITY	0	33	1/14/1999	2/16/2023	24.10685	0.049646	0.144273	-0.07223	4.538242
1260	ET7.1	38.26783	-75.7879	SALINITY	0.5	410	1/8/1986	12/13/2023	37.95342	-0.01386	-0.00687	-0.03176	7.018584
1260	ET7.1	38.26783	-75.7879	SALINITY	1	399	1/8/1986	12/13/2023	37.95342	-0.01575	-0.0074	-0.03279	7.199511
1260	ET7.1	38.26783	-75.7879	SALINITY	1.5	3	4/4/1990	2/12/1996	5.863014	-0.44728	-0.16386	-0.59555	9.407481
1260	ET7.1	38.26783	-75.7879	SALINITY	1.6	3	10/4/1995	1/7/2002	6.265753	-4.7168	0.920291	-6.17493	53.24692
1260	ET7.1	38.26783	-75.7879	SALINITY	1.8	3	10/18/1990	12/12/2000	10.1589	0.419906	1.165991	-0.81007	-4.13153
1260	ET7.1	38.26783	-75.7879	SALINITY	2	377	1/8/1986	12/13/2023	37.95342	-0.01232	-0.00639	-0.03291	7.42747
1260	ET7.1	38.26783	-75.7879	SALINITY	2.1	4	2/11/1998	2/13/2018	20.01918	0.318411	0.411031	0.169422	0.518283
1260	ET7.1	38.26783	-75.7879	SALINITY	2.4	5	11/20/1989	5/30/2001	11.53151	0.335741	0.722178	-0.19153	4.692888
1260	ET7.1	38.26783	-75.7879	SALINITY	3	353	1/8/1986	12/13/2023	37.95342	-0.00622	-0.00513	-0.0333	7.377771
1260	ET7.1	38.26783	-75.7879	SALINITY	4	345	2/25/1986	12/13/2023	37.82192	-0.00857	-0.00699	-0.03648	7.695964
1260	ET7.1	38.26783	-75.7879	SALINITY	4.2	4	10/20/1988	5/17/2018	29.59178	-0.16658	-0.02589	-0.2575	12.92368
1260	ET7.1	38.26783	-75.7879	SALINITY	4.5	5	2/24/1987	12/9/2021	34.8137	0.149624	0.343099	0.077091	1.862133
1260	ET7.1	38.26783	-75.7879	SALINITY	5	327	2/25/1986	12/13/2023	37.82192	-0.01399	-0.01204	-0.0413	7.90765
1260	ET7.1	38.26783	-75.7879	SALINITY	5.2	5	4/6/1988	4/12/2023	35.03836	0.084604	0.132607	-0.1165	3.435581
1260	ET7.1	38.26783	-75.7879	SALINITY	5.4	5	4/11/2002	8/9/2023	21.34247	0.550018	1.125477	-0.16939	-14.8538
1260	ET7.1	38.26783	-75.7879	SALINITY	5.5	8	2/22/1988	12/13/2023	35.83014	0.104136	0.20455	0.079057	5.43889
1260	ET7.1	38.26783	-75.7879	SALINITY	5.6	7	2/25/1986	6/15/2023	37.32603	0.031021	0.48759	-0.08114	4.645921

1308	LE1.2	38.37887	-76.5113	SALINITY	6	544	7/12/1990	12/20/2023	33.46301	0.00812	0.016273	-0.00882	12.32993
1308	LE1.2	38.37887	-76.5113	SALINITY	6.1	112	1/9/1985	6/20/1990	5.446575	-0.28426	-0.21044	-0.45285	14.50084
1308	LE1.2	38.37887	-76.5113	SALINITY	9	543	7/12/1990	12/20/2023	33.46301	-0.00272	0.011292	-0.01239	12.91468
1308	LE1.2	38.37887	-76.5113	SALINITY	9.1	112	1/9/1985	6/20/1990	5.446575	-0.31192	-0.22428	-0.44781	14.8637
1309	LE1.3	38.3398	-76.4849	SALINITY	0.3	19	2/20/1985	12/16/1985	0.819178	4.913223	5.616108	3.613996	11.27184
1309	LE1.3	38.3398	-76.4849	SALINITY	0.5	669	1/9/1985	12/20/2023	38.96986	-0.0265	-0.00951	-0.02852	11.99476
1309	LE1.3	38.3398	-76.4849	SALINITY	1	266	7/5/1994	12/20/2023	29.47945	0.091529	0.108532	0.06367	9.710097
1309	LE1.3	38.3398	-76.4849	SALINITY	1.5	7	11/16/2004	7/5/2006	1.632877	-0.67805	0.686497	-4.09559	25.3409
1309	LE1.3	38.3398	-76.4849	SALINITY	12	547	7/12/1990	12/20/2023	33.46301	0.007749	0.019991	-0.00469	12.74048
1309	LE1.3	38.3398	-76.4849	SALINITY	12.2	112	1/9/1985	6/20/1990	5.446575	-0.33345	-0.25324	-0.50376	14.94784
1309	LE1.3	38.3398	-76.4849	SALINITY	15	543	7/12/1990	12/20/2023	33.46301	0.007122	0.019589	-0.00495	12.92236
1309	LE1.3	38.3398	-76.4849	SALINITY	15.2	112	1/9/1985	6/20/1990	5.446575	-0.30384	-0.20944	-0.46349	15.28182
1309	LE1.3	38.3398	-76.4849	SALINITY	18	545	7/12/1990	12/20/2023	33.46301	0	0.016272	-0.00783	13.14025
1309	LE1.3	38.3398	-76.4849	SALINITY	18.3	109	2/20/1985	6/20/1990	5.331507	-0.34865	-0.24548	-0.49885	15.73587
1309	LE1.3	38.3398	-76.4849	SALINITY	2	266	7/5/1994	12/20/2023	29.47945	0.091831	0.108024	0.062792	9.770485
1309	LE1.3	38.3398	-76.4849	SALINITY	2.5	7	11/16/2004	7/5/2006	1.632877	-0.00081	0.860443	-3.4737	11.62726
1309	LE1.3	38.3398	-76.4849	SALINITY	20	9	3/26/1986	9/9/2010	24.47397	0.43439	1.839625	-0.00726	3.434907
1309	LE1.3	38.3398	-76.4849	SALINITY	21	176	8/12/1985	12/20/2023	38.38082	0.026534	0.07302	0.012942	13.75295
1309	LE1.3	38.3398	-76.4849	SALINITY	21.5	12	4/22/1985	7/12/1990	5.224658	0.516399	1.274603	-0.05044	14.09604
1309	LE1.3	38.3398	-76.4849	SALINITY	22	403	3/26/1985	10/24/2023	38.60548	0	0.022775	-0.01213	13.23724
1309	LE1.3	38.3398	-76.4849	SALINITY	22.5	74	4/8/1985	2/24/1997	11.89041	0.136195	0.316879	0.065432	13.77404
1309	LE1.3	38.3398	-76.4849	SALINITY	22.9	14	2/20/1985	9/26/1988	3.6	0.382259	0.791835	0.00192	14.26487
1309	LE1.3	38.3398	-76.4849	SALINITY	23	84	1/19/1987	9/18/2023	36.68767	0.012319	0.028417	-0.00427	12.96377
1309	LE1.3	38.3398	-76.4849	SALINITY	23.5	7	12/3/1986	11/7/2019	32.95068	-0.11519	0.262284	-0.52795	15.85807
1309	LE1.3	38.3398	-76.4849	SALINITY	24	23	5/7/1986	11/20/2023	37.56438	0.040362	0.112731	0.008153	13.12032
1309	LE1.3	38.3398	-76.4849	SALINITY	25	5	4/9/1986	4/5/2022	36.0137	-0.23965	0.108542	-0.36677	22.99381
1309	LE1.3	38.3398	-76.4849	SALINITY	3	677	1/9/1985	12/20/2023	38.96986	-0.02954	-0.0115	-0.02958	12.58554
1309	LE1.3	38.3398	-76.4849	SALINITY	4	9	7/13/2004	10/18/2006	2.265753	0.236055	1.760067	-0.54993	6.19723
1309	LE1.3	38.3398	-76.4849	SALINITY	5	8	7/13/2004	7/12/2005	0.99726	-0.55781	0.181144	-3.92546	22.01498
1309	LE1.3	38.3398	-76.4849	SALINITY	6	553	7/12/1990	12/20/2023	33.46301	0.013848	0.020864	-0.00427	12.18398
1309	LE1.3	38.3398	-76.4849	SALINITY	6.1	112	1/9/1985	6/20/1990	5.446575	-0.28591	-0.21169	-0.47766	14.58506
1309	LE1.3	38.3398	-76.4849	SALINITY	7	5	7/13/2004	7/12/2005	0.99726	-1.36332	1.084266	-3.59444	37.76731
1309	LE1.3	38.3398	-76.4849	SALINITY	8	6	7/13/2004	7/12/2005	0.99726	-0.74724	0.28251	-4.68158	23.71063
1309	LE1.3	38.3398	-76.4849	SALINITY	9	549	7/12/1990	12/20/2023	33.46301	0.010873	0.021032	-0.00377	12.41713
1309	LE1.3	38.3398	-76.4849	SALINITY	9.1	112	1/9/1985	6/20/1990	5.446575	-0.26575	-0.21146	-0.49184	14.63055
1310	LE1.4	38.312	-76.4215	SALINITY	0.3	19	2/20/1985	12/16/1985	0.819178	4.864875	6.314113	2.897908	11.9038
1310	LE1.4	38.312	-76.4215	SALINITY	0.5	661	1/9/1985	12/20/2023	38.96986	-0.02859	-0.00919	-0.02923	12.77434
1310	LE1.4	38.312	-76.4215	SALINITY	1	255	7/17/1995	12/20/2023	28.44658	0.064923	0.09825	0.042424	11.38416
1310	LE1.4	38.312	-76.4215	SALINITY	10	12	7/13/2004	10/13/2005	1.252055	9.598148	15.23279	4.532258	-182.999
1310	LE1.4	38.312	-76.4215	SALINITY	11	12	7/13/2004	10/13/2005	1.252055	10.40926	14.59362	4.857109	-199.787
1310	LE1.4	38.312	-76.4215	SALINITY	12	557	7/12/1990	12/20/2023	33.46301	-0.0121	0.008499	-0.01592	13.88242
1310	LE1.4	38.312	-76.4215	SALINITY	12.2	113	1/9/1985	6/20/1990	5.446575	-0.28603	-0.21404	-0.4593	15.77183

1323	LE3.7	37.53069	-76.3066	SALINITY	8	5	12/15/1987	4/17/1989	1.339726	4.616403	5.830371	-1.84928	-0.99918
1324	LE4.1	37.41883	-76.6913	SALINITY	1	483	9/4/1984	1/24/2024	39.4137	0.000987	0.012145	-0.01567	13.18142
1324	LE4.1	37.41883	-76.6913	SALINITY	10	74	9/4/1984	12/20/2022	38.31781	0.028024	0.0663	-0.11928	15.23755
1324	LE4.1	37.41883	-76.6913	SALINITY	11	5	10/2/2001	6/22/2016	14.73151	-0.38925	-0.24819	-0.52225	27.08373
1324	LE4.1	37.41883	-76.6913	SALINITY	2	161	4/23/2007	1/24/2024	16.76712	0.052051	0.085867	-0.02962	11.52038
1324	LE4.1	37.41883	-76.6913	SALINITY	3	486	9/4/1984	1/24/2024	39.4137	0.02045	0.042766	0.015726	13.35936
1324	LE4.1	37.41883	-76.6913	SALINITY	4	161	4/23/2007	1/24/2024	16.76712	0.039021	0.085332	-0.01942	13.50462
1324	LE4.1	37.41883	-76.6913	SALINITY	5	485	9/4/1984	1/24/2024	39.4137	0	0.012563	-0.01101	14.83608
1324	LE4.1	37.41883	-76.6913	SALINITY	6	160	4/23/2007	1/24/2024	16.76712	0.053515	0.091708	-0.00084	14.155
1324	LE4.1	37.41883	-76.6913	SALINITY	7	482	9/4/1984	1/24/2024	39.4137	0.012504	0.020378	-0.00226	15.26358
1324	LE4.1	37.41883	-76.6913	SALINITY	8	282	9/24/1984	10/18/2023	39.09041	0.011576	0.021248	-0.00335	15.52971
1324	LE4.1	37.41883	-76.6913	SALINITY	9	312	9/4/1984	10/18/2023	39.14521	0.009563	0.022344	-0.01122	15.19015
1325	LE4.2	37.29044	-76.5781	SALINITY	1	485	9/4/1984	1/24/2024	39.4137	-0.02556	-0.0145	-0.03722	18.01053
1325	LE4.2	37.29044	-76.5781	SALINITY	10	178	3/26/1985	1/24/2024	38.85753	0.034216	0.060741	0.011268	18.9711
1325	LE4.2	37.29044	-76.5781	SALINITY	11	421	9/4/1984	1/24/2024	39.4137	-0.01666	-0.00959	-0.02945	20.0501
1325	LE4.2	37.29044	-76.5781	SALINITY	12	180	3/27/1986	1/24/2024	37.85479	0.048017	0.066367	0.024343	18.49768
1325	LE4.2	37.29044	-76.5781	SALINITY	13	342	9/4/1984	1/24/2024	39.4137	-0.03494	-0.02223	-0.04491	20.57021
1325	LE4.2	37.29044	-76.5781	SALINITY	14	169	8/6/1985	1/24/2024	38.49315	0.020576	0.039359	0.001281	19.55104
1325	LE4.2	37.29044	-76.5781	SALINITY	15	240	9/4/1984	1/24/2024	39.4137	-0.03787	-0.02299	-0.05085	20.83043
1325	LE4.2	37.29044	-76.5781	SALINITY	16	145	9/4/1984	1/24/2024	39.4137	0.005562	0.039143	-0.00937	19.88027
1325	LE4.2	37.29044	-76.5781	SALINITY	17	147	9/24/1984	2/15/2022	37.41918	-0.02318	-0.00347	-0.04303	20.51912
1325	LE4.2	37.29044	-76.5781	SALINITY	18	40	2/13/1986	10/25/2018	32.71781	0.006204	0.049981	-0.06962	20.30848
1325	LE4.2	37.29044	-76.5781	SALINITY	19	4	3/23/1987	3/21/2017	30.01644	0.000466	0.090474	-0.01743	18.08826
1325	LE4.2	37.29044	-76.5781	SALINITY	2	162	4/23/2007	1/24/2024	16.76712	0.025567	0.061292	-0.04133	16.8189
1325	LE4.2	37.29044	-76.5781	SALINITY	3	486	9/4/1984	1/24/2024	39.4137	0.013727	0.033421	0.009256	17.75664
1325	LE4.2	37.29044	-76.5781	SALINITY	4	162	4/23/2007	1/24/2024	16.76712	0.043797	0.078246	-0.00768	17.99046
1325	LE4.2	37.29044	-76.5781	SALINITY	5	485	9/4/1984	1/24/2024	39.4137	-0.01273	-0.00596	-0.02621	18.89284
1325	LE4.2	37.29044	-76.5781	SALINITY	6	165	10/2/2001	1/24/2024	22.32603	0.042746	0.061764	-0.00947	18.4089
1325	LE4.2	37.29044	-76.5781	SALINITY	7	483	9/4/1984	1/24/2024	39.4137	-0.01263	-0.00457	-0.02343	19.43685
1325	LE4.2	37.29044	-76.5781	SALINITY	8	172	7/3/1985	1/24/2024	38.5863	0.030184	0.058778	0.001082	18.77767
1325	LE4.2	37.29044	-76.5781	SALINITY	9	465	9/4/1984	1/24/2024	39.4137	-0.01648	-0.00658	-0.0258	19.83351
1328	LE4.3	37.23392	-76.4309	SALINITY	1	480	9/4/1984	1/24/2024	39.4137	-0.03732	-0.03214	-0.05161	20.12631
1328	LE4.3	37.23392	-76.4309	SALINITY	10	171	11/15/1984	1/24/2024	39.21644	0.028524	0.057138	0.011936	20.3779
1328	LE4.3	37.23392	-76.4309	SALINITY	11	467	9/4/1984	1/24/2024	39.4137	-0.02374	-0.01829	-0.03568	21.82673
1328	LE4.3	37.23392	-76.4309	SALINITY	12	169	10/17/1985	1/24/2024	38.29589	0.000866	0.03354	-0.01829	21.58751
1328	LE4.3	37.23392	-76.4309	SALINITY	13	392	9/4/1984	8/16/2023	38.9726	-0.0257	-0.01281	-0.0348	22.60703
1328	LE4.3	37.23392	-76.4309	SALINITY	14	108	12/13/1984	8/16/2023	38.69863	-0.05069	-0.01611	-0.06333	23.0434
1328	LE4.3	37.23392	-76.4309	SALINITY	15	144	4/25/1985	5/17/2022	37.08493	0	0.026437	-0.07233	22.57434
1328	LE4.3	37.23392	-76.4309	SALINITY	16	19	9/24/1986	10/21/2014	28.09315	-0.02051	0.06201	-0.36008	22.19358
1328	LE4.3	37.23392	-76.4309	SALINITY	17	102	4/25/1985	10/21/2014	29.50959	-0.03745	-0.00917	-0.11391	23.3857
1328	LE4.3	37.23392	-76.4309	SALINITY	18	18	6/4/1987	10/21/2014	27.4	-0.02645	0.04559	-0.25577	23.13621
1328	LE4.3	37.23392	-76.4309	SALINITY	19	74	4/25/1985	10/21/2014	29.50959	0	0.037455	-0.11791	23.67608

1360	MKB0016	38.21389	-75.6714	SALINITY	0.4	57	1/22/2012	1/26/2023	11.01918	0	NA	NA	0
1360	MKB0016	38.21389	-75.6714	SALINITY	0.5	7	10/31/2012	7/30/2018	5.747945	0	NA	NA	0
1361	MNK0146	38.175	-75.7236	SALINITY	0	44	1/6/2020	12/13/2023	3.936986	1.149578	1.691194	0.929943	-20.1667
1361	MNK0146	38.175	-75.7236	SALINITY	0.5	120	4/14/2003	12/11/2013	10.66849	0.350442	0.387289	0.233833	0.704627
1361	MNK0146	38.175	-75.7236	SALINITY	1	119	4/14/2003	12/11/2013	10.66849	0.340613	0.387099	0.228597	0.732556
1361	MNK0146	38.175	-75.7236	SALINITY	1.5	6	5/23/2012	9/25/2013	1.342466	-0.33606	0.586254	-4.4057	5.213223
1361	MNK0146	38.175	-75.7236	SALINITY	2	116	4/14/2003	12/11/2013	10.66849	0.332231	0.38792	0.215103	1.078639
1361	MNK0146	38.175	-75.7236	SALINITY	2.3	4	5/22/2008	8/26/2013	5.265753	0.164269	0.328538	0.328538	-0.83935
1361	MNK0146	38.175	-75.7236	SALINITY	2.4	3	5/23/2012	11/14/2013	1.479452	11.32152	14.24683	1.08293	-116.214
1361	MNK0146	38.175	-75.7236	SALINITY	2.5	3	11/9/2004	6/18/2013	8.610959	-0.90185	-0.07374	-0.97559	11.5915
1361	MNK0146	38.175	-75.7236	SALINITY	2.7	4	12/17/2008	4/24/2013	4.353425	0	-1.0779	-1.0779	4.38
1361	MNK0146	38.175	-75.7236	SALINITY	2.8	5	3/17/2010	10/29/2013	3.621918	-1.68107	4.986464	-1.98225	18.89391
1361	MNK0146	38.175	-75.7236	SALINITY	2.9	3	9/26/2011	5/22/2013	1.654795	-5.06019	-3.12932	-6.14287	53.73189
1361	MNK0146	38.175	-75.7236	SALINITY	3	87	4/14/2003	12/11/2013	10.66849	0.471404	0.53806	0.334365	0.057307
1361	MNK0146	38.175	-75.7236	SALINITY	3.2	6	9/19/2007	6/5/2013	5.715068	-0.35024	0.090949	-0.98126	9.363532
1361	MNK0146	38.175	-75.7236	SALINITY	3.4	3	6/20/2007	11/20/2008	1.421918	3.762443	5.337445	3.544844	-12.3201
1361	MNK0146	38.175	-75.7236	SALINITY	3.5	5	6/18/2003	10/15/2013	10.33425	0.580622	1.028616	0.352726	-0.26425
1361	MNK0146	38.175	-75.7236	SALINITY	3.6	6	5/7/2003	8/14/2013	10.27945	0.101824	0.865259	-0.23463	2.82244
1361	MNK0146	38.175	-75.7236	SALINITY	3.7	6	7/9/2003	4/30/2012	8.816438	0.030905	0.302565	-0.05134	-0.0508
1361	MNK0146	38.175	-75.7236	SALINITY	3.8	5	3/16/2005	7/15/2009	4.334247	0.08001	1.159567	-0.02602	1.074318
1361	MNK0146	38.175	-75.7236	SALINITY	3.9	6	9/29/2003	6/6/2011	7.690411	1.396798	2.024179	0.14851	-3.41996
1361	MNK0146	38.175	-75.7236	SALINITY	4	40	4/14/2003	4/17/2013	10.01644	0.636945	0.770945	0.304078	0
1361	MNK0146	38.175	-75.7236	SALINITY	4.1	5	3/17/2004	4/18/2012	8.093151	0.243507	0.275416	-0.56076	0.525206
1361	MNK0146	38.175	-75.7236	SALINITY	4.2	8	6/23/2005	9/17/2012	7.241096	0.166883	0.636718	-0.5297	3.152641
1361	MNK0146	38.175	-75.7236	SALINITY	4.3	6	10/7/2004	4/13/2011	6.517808	0.443977	0.459344	0.102328	-0.16886
1361	MNK0146	38.175	-75.7236	SALINITY	4.4	6	4/14/2003	7/12/2011	8.249315	1.262925	1.765707	1.122998	0
1361	MNK0146	38.175	-75.7236	SALINITY	4.5	8	10/8/2003	12/13/2012	9.189041	0.510195	0.673462	0.208639	2.651604
1364	MON0020	39.27171	-77.4416	SALINITY	0	419	1/7/1986	9/7/2022	36.69041	0	NA	NA	0
1365	MON0155	39.38778	-77.3811	SALINITY	0	439	1/27/1986	12/6/2023	37.88219	0	NA	NA	0
1366	MON0269	39.48027	-77.3894	SALINITY	0	438	1/27/1986	12/6/2023	37.88219	0	NA	NA	0
1367	MON0528	39.67917	-77.2349	SALINITY	0	433	1/27/1986	12/6/2023	37.88219	0	0.037207	0.037207	0
1368	MON0546	39.69645	-77.2395	SALINITY	0	6	7/19/2005	1/23/2018	12.52329	0	NA	NA	0
1368	MON0546	39.69645	-77.2395	SALINITY	0.1	7	11/8/2005	9/6/2023	17.83836	0	NA	NA	0
1368	MON0546	39.69645	-77.2395	SALINITY	0.2	111	3/22/2006	8/14/2023	17.40822	0	-0.00232	-0.005	0
1368	MON0546	39.69645	-77.2395	SALINITY	0.3	58	10/9/2005	3/7/2022	16.41918	0	NA	NA	0
1368	MON0546	39.69645	-77.2395	SALINITY	0.4	61	1/26/2006	9/25/2023	17.67397	0	NA	NA	0
1368	MON0546	39.69645	-77.2395	SALINITY	0.5	19	12/5/2005	5/2/2023	17.41644	0	NA	NA	0
1368	MON0546	39.69645	-77.2395	SALINITY	0.6	23	10/26/2005	4/1/2022	16.4411	0	NA	NA	0
1368	MON0546	39.69645	-77.2395	SALINITY	0.7	17	1/18/2006	4/29/2023	17.28767	0	NA	NA	0
1368	MON0546	39.69645	-77.2395	SALINITY	0.8	13	7/9/2005	4/8/2022	16.7589	0	NA	NA	0
1368	MON0546	39.69645	-77.2395	SALINITY	0.9	7	4/21/2008	9/9/2021	13.39452	0	NA	NA	0
1368	MON0546	39.69645	-77.2395	SALINITY	1	12	1/3/2006	2/4/2022	16.09863	0	NA	NA	0

1469	PXT0972	39.23931	-77.0562	SALINITY	0.6	5	5/12/2008	9/6/2022	14.32877	0	NA	NA	0
1469	PXT0972	39.23931	-77.0562	SALINITY	0.7	4	3/2/2007	1/24/2019	11.90685	0	NA	NA	0
1470	RCM0111	38.99302	-77.063	SALINITY	0	433	2/3/1986	12/6/2023	37.86301	0	0.017525	0.002517	0
1471	RCR01	38.9865	-77.0636	SALINITY	0.1	25	3/9/2010	11/8/2016	6.673973	-0.01349	0.008297	-0.02085	0.179861
1474	RCR09	38.92844	-77.0494	SALINITY	0.1	24	3/9/2010	11/8/2016	6.673973	0	0.016228	-0.02622	0.185324
1475	RET1.1	38.4909	-76.6643	SALINITY	0.3	19	2/20/1985	12/16/1985	0.819178	3.802179	5.264461	2.580985	9.24477
1475	RET1.1	38.4909	-76.6643	SALINITY	0.5	637	1/9/1985	12/20/2023	38.96986	-0.02996	-0.01295	-0.0339	9.411308
1475	RET1.1	38.4909	-76.6643	SALINITY	1	244	9/19/1994	12/20/2023	29.27123	0.074721	0.092429	0.037336	7.290791
1475	RET1.1	38.4909	-76.6643	SALINITY	10	458	9/16/1985	11/20/2023	38.20274	-0.02268	1.62E-05	-0.02684	11.15137
1475	RET1.1	38.4909	-76.6643	SALINITY	10.5	43	2/20/1985	2/24/1997	12.01918	-0.48353	-0.37088	-0.63626	13.16859
1475	RET1.1	38.4909	-76.6643	SALINITY	11	65	4/22/1985	10/24/2023	38.53151	-0.00985	0.033271	-0.02717	11.60719
1475	RET1.1	38.4909	-76.6643	SALINITY	11.5	3	7/13/1987	7/12/1990	3	-1.0986	-0.67402	-1.14458	16.35776
1475	RET1.1	38.4909	-76.6643	SALINITY	11.6	4	3/26/1985	9/21/1987	2.490411	0.224166	0.509956	-1.04545	13.54767
1475	RET1.1	38.4909	-76.6643	SALINITY	12	10	12/16/1996	5/11/2023	26.41644	0.266476	0.33013	0.244581	1.774135
1475	RET1.1	38.4909	-76.6643	SALINITY	2	243	7/17/1995	12/20/2023	28.44658	0.077065	0.099576	0.043598	7.277335
1475	RET1.1	38.4909	-76.6643	SALINITY	3	657	1/9/1985	12/20/2023	38.96986	-0.03077	-0.01571	-0.0343	10.19928
1475	RET1.1	38.4909	-76.6643	SALINITY	6	544	7/12/1990	12/20/2023	33.46301	0.017748	0.026895	0.001049	10.0582
1475	RET1.1	38.4909	-76.6643	SALINITY	6.1	113	1/9/1985	3/4/1991	6.150685	-0.29865	-0.22265	-0.45395	12.43165
1475	RET1.1	38.4909	-76.6643	SALINITY	9	546	5/6/1985	12/20/2023	38.64932	0.005953	0.02175	-0.00347	10.64162
1475	RET1.1	38.4909	-76.6643	SALINITY	9.1	108	1/9/1985	3/4/1991	6.150685	-0.29104	-0.21585	-0.41755	13.14886
1475	RET1.1	38.4909	-76.6643	SALINITY	9.5	6	12/3/1986	1/16/1990	3.123288	-1.44825	-0.89755	-4.09723	17.14968
1476	RET2.1	38.4035	-77.2691	SALINITY	0.3	93	1/6/1986	9/11/2007	21.69315	-0.27932	-0.27216	-0.47378	1.510528
1476	RET2.1	38.4035	-77.2691	SALINITY	0.5	563	10/15/1990	12/12/2023	33.18082	0	0.015749	0.000353	0.278284
1476	RET2.1	38.4035	-77.2691	SALINITY	1	20	4/11/2006	10/14/2008	2.512329	0	0.767248	-0.20382	0.123191
1476	RET2.1	38.4035	-77.2691	SALINITY	2	20	4/11/2006	10/14/2008	2.512329	0.007176	0.803632	-0.17877	0
1476	RET2.1	38.4035	-77.2691	SALINITY	3	20	4/11/2006	10/14/2008	2.512329	0	0.769315	-0.20319	0
1476	RET2.1	38.4035	-77.2691	SALINITY	4	21	10/5/1999	10/14/2008	9.032877	0	0.585708	-0.16632	0
1476	RET2.1	38.4035	-77.2691	SALINITY	4.6	632	1/6/1986	12/12/2023	37.95616	0	0.006729	-0.00582	0.8394
1476	RET2.1	38.4035	-77.2691	SALINITY	5	29	6/13/1994	6/6/2022	28	-0.01627	0.019953	-0.04732	1.696295
1476	RET2.1	38.4035	-77.2691	SALINITY	5.2	3	5/13/1996	12/12/2023	27.6	0.540357	0.576742	0.148035	-16.4241
1476	RET2.1	38.4035	-77.2691	SALINITY	5.4	5	11/17/1997	8/8/2022	24.73973	-0.02179	0.125379	-0.06747	2.357694
1476	RET2.1	38.4035	-77.2691	SALINITY	5.5	8	9/22/1999	9/11/2023	23.9863	0.050943	1.349336	0.008947	0
1476	RET2.1	38.4035	-77.2691	SALINITY	5.6	14	7/22/1991	1/10/2023	31.49315	-0.02934	9.25E-05	-0.05403	2.649797
1476	RET2.1	38.4035	-77.2691	SALINITY	5.7	16	7/8/1991	7/10/2023	32.0274	0.062248	0.216622	0.011973	2.02254
1476	RET2.1	38.4035	-77.2691	SALINITY	5.8	18	3/30/1992	3/6/2023	30.95342	0.015113	0.023696	-0.03571	2.492214
1476	RET2.1	38.4035	-77.2691	SALINITY	5.9	12	1/4/1993	7/15/2019	26.54247	-0.00401	0.038718	-0.11784	0.133317
1476	RET2.1	38.4035	-77.2691	SALINITY	6	85	4/22/1991	8/7/2023	32.31507	0.003819	0.058085	0.005162	0
1476	RET2.1	38.4035	-77.2691	SALINITY	6.1	52	3/12/1991	7/12/2021	30.35616	-0.05922	-0.01913	-0.07807	3.45052
1476	RET2.1	38.4035	-77.2691	SALINITY	6.2	59	6/8/1992	10/11/2022	30.36164	-0.00148	-0.02191	-0.0925	2.15676
1476	RET2.1	38.4035	-77.2691	SALINITY	6.3	58	10/29/1990	12/9/2019	29.13151	-0.00295	0.016601	-0.05323	0.922742
1476	RET2.1	38.4035	-77.2691	SALINITY	6.4	68	6/16/1986	4/10/2023	36.8411	0.036357	0.101922	0.034636	-0.03427
1476	RET2.1	38.4035	-77.2691	SALINITY	6.5	61	4/1/1986	5/9/2022	36.12877	0.016098	0.070563	-0.00257	0.093819

1491	RET5.2	37.20294	-76.7822	SALINITY	2	179	5/12/1992	2/8/2024	31.76438	-0.02942	-0.01385	-0.05756	1.67988
1491	RET5.2	37.20294	-76.7822	SALINITY	3	503	7/31/1984	2/8/2024	39.55068	0.016935	0.024887	0.014549	0.660691
1491	RET5.2	37.20294	-76.7822	SALINITY	4	178	7/12/1999	2/8/2024	24.59452	-0.03328	-0.01724	-0.06413	1.977096
1491	RET5.2	37.20294	-76.7822	SALINITY	5	503	7/31/1984	2/8/2024	39.55068	0.019095	0.024233	0.013329	0.835402
1491	RET5.2	37.20294	-76.7822	SALINITY	6	182	10/2/1985	2/8/2024	38.37808	-0.02521	-0.01493	-0.05919	2.009774
1491	RET5.2	37.20294	-76.7822	SALINITY	7	500	7/31/1984	2/8/2024	39.55068	0.024187	0.02833	0.01692	0.809436
1491	RET5.2	37.20294	-76.7822	SALINITY	8	279	10/11/1984	2/8/2024	39.35342	0.006405	0.013704	-0.00206	1.2
1491	RET5.2	37.20294	-76.7822	SALINITY	9	335	7/31/1984	2/8/2024	39.55068	0.031725	0.038386	0.022302	0.462492
1495	RPP001.99	37.61415	-76.3203	SALINITY	0.1	40	4/2/2007	10/16/2023	16.55068	0.136274	0.227611	0.101709	13.83564
1495	RPP001.99	37.61415	-76.3203	SALINITY	0.25	42	4/2/2007	10/16/2023	16.55068	0.111012	0.210304	0.085303	13.99975
1495	RPP001.99	37.61415	-76.3203	SALINITY	0.5	42	4/2/2007	10/16/2023	16.55068	0.111872	0.20943	0.084623	13.99053
1495	RPP001.99	37.61415	-76.3203	SALINITY	0.75	41	4/2/2007	10/16/2023	16.55068	0.132593	0.223644	0.107399	13.94846
1495	RPP001.99	37.61415	-76.3203	SALINITY	1	42	4/2/2007	10/16/2023	16.55068	0.116135	0.20881	0.086393	14.03362
1495	RPP001.99	37.61415	-76.3203	SALINITY	1.25	34	4/2/2007	10/16/2023	16.55068	0.239145	0.361643	0.184927	13.15
1495	RPP001.99	37.61415	-76.3203	SALINITY	1.5	42	4/2/2007	10/16/2023	16.55068	0.113412	0.204465	0.081504	14.1799
1495	RPP001.99	37.61415	-76.3203	SALINITY	1.75	33	5/1/2007	10/16/2023	16.47123	0.276	0.472582	0.188138	12.8635
1495	RPP001.99	37.61415	-76.3203	SALINITY	2	42	4/2/2007	10/16/2023	16.55068	0.097963	0.200791	0.075296	14.36575
1495	RPP001.99	37.61415	-76.3203	SALINITY	2.25	11	6/6/2007	9/7/2021	14.26575	0.261408	0.769772	-0.35207	16.37369
1495	RPP001.99	37.61415	-76.3203	SALINITY	2.5	42	4/2/2007	10/16/2023	16.55068	0.086334	0.194774	0.069833	14.28311
1495	RPP001.99	37.61415	-76.3203	SALINITY	2.75	11	6/6/2007	9/7/2021	14.26575	0.063135	0.755272	-0.50626	16.77938
1495	RPP001.99	37.61415	-76.3203	SALINITY	3	41	4/2/2007	10/16/2023	16.55068	0.098054	0.182861	0.057174	14.66849
1495	RPP001.99	37.61415	-76.3203	SALINITY	3.25	10	6/6/2007	9/7/2021	14.26575	0.075981	0.411506	-0.89528	16.5619
1495	RPP001.99	37.61415	-76.3203	SALINITY	3.5	32	4/2/2007	10/16/2023	16.55068	0.292213	0.601688	0.227229	12.68818
1495	RPP001.99	37.61415	-76.3203	SALINITY	3.75	4	10/22/2007	5/22/2023	15.59178	-0.26962	-0.10038	-0.27066	20.67995
1495	RPP001.99	37.61415	-76.3203	SALINITY	4	9	4/12/2021	10/16/2023	2.512329	2.092811	3.147541	0.554142	-15.1475
1496	RPP006.96	37.59248	-76.4117	SALINITY	0.1	42	4/2/2007	10/16/2023	16.55068	0.162073	0.224536	0.107456	13.37927
1496	RPP006.96	37.59248	-76.4117	SALINITY	0.25	42	4/2/2007	10/16/2023	16.55068	0.162073	0.224611	0.10794	13.37927
1496	RPP006.96	37.59248	-76.4117	SALINITY	0.5	42	4/2/2007	10/16/2023	16.55068	0.164732	0.226616	0.108895	13.3788
1496	RPP006.96	37.59248	-76.4117	SALINITY	0.75	42	4/2/2007	10/16/2023	16.55068	0.165081	0.227336	0.109346	13.37839
1496	RPP006.96	37.59248	-76.4117	SALINITY	1	42	4/2/2007	10/16/2023	16.55068	0.165081	0.226507	0.109361	13.37833
1496	RPP006.96	37.59248	-76.4117	SALINITY	1.25	36	4/2/2007	10/16/2023	16.55068	0.24928	0.337717	0.170717	12.42
1496	RPP006.96	37.59248	-76.4117	SALINITY	1.5	24	4/2/2007	10/16/2023	16.55068	0.511396	0.966166	0.405377	9.245083
1496	RPP006.96	37.59248	-76.4117	SALINITY	1.75	5	9/24/2007	10/16/2023	16.07123	0.543545	1.561826	-0.99403	7.938925
1497	RPP010.60	37.63189	-76.4449	SALINITY	1	42	4/2/2007	10/16/2023	16.55068	0.168568	0.244538	0.117936	12.91069
1497	RPP010.60	37.63189	-76.4449	SALINITY	10	42	4/2/2007	10/16/2023	16.55068	0.092476	0.160118	0.056641	14.97762
1497	RPP010.60	37.63189	-76.4449	SALINITY	11	27	4/2/2007	10/16/2023	16.55068	0.130246	0.277456	-0.03463	15.699
1497	RPP010.60	37.63189	-76.4449	SALINITY	12	17	4/2/2007	8/10/2009	2.358904	1.057841	1.58723	-0.07396	14.58483
1497	RPP010.60	37.63189	-76.4449	SALINITY	13	8	4/2/2007	4/13/2009	2.032877	2.52025	8.579895	1.702606	13.59439
1497	RPP010.60	37.63189	-76.4449	SALINITY	14	4	4/2/2007	4/13/2009	2.032877	13.02769	14.24542	6.915483	13.51
1497	RPP010.60	37.63189	-76.4449	SALINITY	2	42	4/2/2007	10/16/2023	16.55068	0.171209	0.248761	0.120171	12.91024
1497	RPP010.60	37.63189	-76.4449	SALINITY	3	42	4/2/2007	10/16/2023	16.55068	0.167883	0.247082	0.115125	13.08386
1497	RPP010.60	37.63189	-76.4449	SALINITY	4	42	4/2/2007	10/16/2023	16.55068	0.115812	0.213365	0.09031	13.62041

1521	SBE2	36.8134	-76.2903	SALINITY	9	356	2/28/1989	1/23/2024	34.92329	0.028572	0.042796	0.01942	19.78122
1524	SBE5	36.76987	-76.2961	SALINITY	1	384	2/28/1989	2/21/2024	35.00274	0.005658	0.029463	-0.00791	16.74533
1524	SBE5	36.76987	-76.2961	SALINITY	10	61	3/23/1989	4/21/2005	16.09041	0.517778	0.591388	0.257757	17.33591
1524	SBE5	36.76987	-76.2961	SALINITY	10.5	9	10/30/1989	6/10/1992	2.613699	1.238307	1.780952	-1.13533	17.09552
1524	SBE5	36.76987	-76.2961	SALINITY	11	24	5/10/1989	11/14/1995	6.517808	0.827674	1.010546	0.397443	15.35689
1524	SBE5	36.76987	-76.2961	SALINITY	11.5	3	11/19/1990	1/24/1992	1.180822	0.604061	2.10498	-1.16217	19.33135
1524	SBE5	36.76987	-76.2961	SALINITY	12	6	9/27/1989	11/14/1995	6.134247	1.215492	9.085185	0.646013	14.71132
1524	SBE5	36.76987	-76.2961	SALINITY	2	384	2/28/1989	2/21/2024	35.00274	0.011429	0.034649	-0.00029	17.14712
1524	SBE5	36.76987	-76.2961	SALINITY	3	368	2/28/1989	2/21/2024	35.00274	0.012518	0.033094	0.001922	17.19514
1524	SBE5	36.76987	-76.2961	SALINITY	4	327	2/28/1989	2/21/2024	35.00274	0.037005	0.049439	0.020123	16.72521
1524	SBE5	36.76987	-76.2961	SALINITY	5	262	2/28/1989	2/21/2024	35.00274	0.028152	0.045145	0.015713	17.35007
1524	SBE5	36.76987	-76.2961	SALINITY	6	185	2/28/1989	9/13/2023	34.56164	0.022905	0.044441	0.01146	17.71276
1524	SBE5	36.76987	-76.2961	SALINITY	7	126	2/28/1989	7/13/2022	33.39178	0.01436	0.061596	-0.00814	18.31389
1524	SBE5	36.76987	-76.2961	SALINITY	8	105	2/28/1989	7/17/2019	30.4	0.073084	0.173504	0.036429	18.64553
1524	SBE5	36.76987	-76.2961	SALINITY	9	88	2/28/1989	4/12/2017	28.13699	0.061112	0.252535	0.042947	18.49454
1524	SBE5	36.76987	-76.2961	SALINITY	9.5	4	7/18/1990	11/18/1992	2.339726	-2.07659	-1.42503	-2.37147	26.27309
1525	SEN0008	39.07958	-77.3396	SALINITY	0	439	1/7/1986	12/6/2023	37.93699	0	0.018151	0.001069	0
1581	TBK01	38.91789	-77.1205	SALINITY	0.1	7	4/6/2010	10/16/2012	2.531507	0.039731	0.133254	-0.01682	0.149138
1582	TBR01	38.94528	-77.0511	SALINITY	0.1	9	4/6/2010	10/21/2014	4.545205	-0.01111	0.077063	-0.03035	0.29813
1583	TCO01	38.89456	-77.0747	SALINITY	0.1	18	6/15/2010	3/3/2015	4.717808	0	0.040365	-0.01465	0.16
1584	TCO06	38.92761	-77.1014	SALINITY	0.1	21	6/15/2010	3/3/2015	4.717808	0.002692	0.016286	3.44E-05	0.156133
1585	TDA01	38.92872	-77.1219	SALINITY	0.1	9	4/6/2010	7/15/2014	4.276712	0	0.106456	-0.0296	0.32
1586	TDO01	38.91556	-77.0597	SALINITY	0.1	8	4/6/2010	7/15/2014	4.276712	-0.03218	-0.0112	-0.0386	0.324716
1587	TDU01	38.88345	-76.9764	SALINITY	0.1	7	4/5/2010	10/2/2012	2.49589	-0.0189	0.034263	-0.06559	0.225886
1588	TF1.0	38.95557	-76.6941	SALINITY	0	849	1/9/1985	12/20/2023	38.96986	0	0.010182	0.000562	0
1588	TF1.0	38.95557	-76.6941	SALINITY	0.1	3	3/8/1999	3/22/1999	0.038356	0	NA	NA	0
1589	TF1.2	38.8143	-76.7509	SALINITY	0	636	1/9/1985	12/20/2023	38.96986	0	0.013457	-0.00054	0
1589	TF1.2	38.8143	-76.7509	SALINITY	0.1	12	3/8/1999	9/17/2015	16.53973	0	NA	NA	0
1589	TF1.2	38.8143	-76.7509	SALINITY	0.2	117	11/7/2005	9/18/2023	17.87397	0	0.073858	-0.02898	0
1589	TF1.2	38.8143	-76.7509	SALINITY	0.3	59	12/7/2005	5/22/2023	17.46575	0	-0.00423	-0.11085	0
1589	TF1.2	38.8143	-76.7509	SALINITY	0.4	50	1/23/2006	1/23/2023	17.01096	0	NA	NA	0
1589	TF1.2	38.8143	-76.7509	SALINITY	0.5	14	10/26/2005	11/17/2022	17.07123	0	0.0104	0.0104	0
1589	TF1.2	38.8143	-76.7509	SALINITY	0.6	26	5/12/2006	4/23/2023	16.9589	0	0.01743	-0.01408	0
1589	TF1.2	38.8143	-76.7509	SALINITY	0.7	10	2/21/2007	7/26/2023	16.43562	0	NA	NA	0
1589	TF1.2	38.8143	-76.7509	SALINITY	0.8	13	10/8/2005	4/29/2023	17.56712	0	NA	NA	0
1589	TF1.2	38.8143	-76.7509	SALINITY	0.9	12	1/7/2009	10/26/2021	12.80822	0	NA	NA	0
1589	TF1.2	38.8143	-76.7509	SALINITY	1	16	7/6/2006	7/1/2023	16.99726	0	0.077851	-0.01342	0
1589	TF1.2	38.8143	-76.7509	SALINITY	1.1	14	7/9/2005	7/9/2022	17.01096	0	NA	NA	0
1589	TF1.2	38.8143	-76.7509	SALINITY	1.2	5	3/13/2010	4/7/2022	12.07671	0	NA	NA	0
1589	TF1.2	38.8143	-76.7509	SALINITY	1.3	6	9/2/2006	4/1/2021	14.58904	0	NA	NA	0
1589	TF1.2	38.8143	-76.7509	SALINITY	1.4	4	7/12/2013	10/30/2021	8.306849	0	NA	NA	0
1589	TF1.2	38.8143	-76.7509	SALINITY	1.5	5	6/5/2008	10/12/2018	10.3589	0	NA	NA	0

1598	TF2.3	38.6082	-77.1739	SALINITY	9	22	2/26/1985	10/14/2008	23.64658	0	0.177786	0.177786	0
1598	TF2.3	38.6082	-77.1739	SALINITY	9.1	653	8/6/1984	12/12/2023	39.37534	0	0.009276	-0.00511	0
1599	TF2.4	38.5301	-77.2654	SALINITY	0.3	93	1/6/1986	9/24/1990	4.717808	0	-0.15247	-0.48446	0
1599	TF2.4	38.5301	-77.2654	SALINITY	0.5	565	10/15/1990	12/12/2023	33.18082	0	0.012132	-0.00769	0
1599	TF2.4	38.5301	-77.2654	SALINITY	1	21	4/11/2006	10/14/2008	2.512329	0	0.812428	-0.00666	0
1599	TF2.4	38.5301	-77.2654	SALINITY	2	21	4/11/2006	10/14/2008	2.512329	0	0.921917	0.00194	0
1599	TF2.4	38.5301	-77.2654	SALINITY	3	21	4/11/2006	10/14/2008	2.512329	0	0.929627	0.010269	0
1599	TF2.4	38.5301	-77.2654	SALINITY	4	21	4/11/2006	10/14/2008	2.512329	0	0.979071	0.013021	0
1599	TF2.4	38.5301	-77.2654	SALINITY	4.6	637	1/6/1986	12/12/2023	37.95616	0	0.00728	-0.009	0
1599	TF2.4	38.5301	-77.2654	SALINITY	5	21	6/15/1998	10/14/2008	10.33973	0	0.860998	0.028695	0
1599	TF2.4	38.5301	-77.2654	SALINITY	6	22	6/15/1998	5/9/2022	23.91507	0	0.745357	-0.02459	0
1599	TF2.4	38.5301	-77.2654	SALINITY	6.6	6	8/7/2000	5/23/2005	4.794521	0	NA	NA	0
1599	TF2.4	38.5301	-77.2654	SALINITY	6.9	5	6/1/1998	12/9/2013	15.53425	-0.00195	0.072542	-0.02372	0.194701
1599	TF2.4	38.5301	-77.2654	SALINITY	7	29	7/8/1991	11/9/2022	31.36164	0	0.143733	-0.00452	0
1599	TF2.4	38.5301	-77.2654	SALINITY	7.2	10	8/10/1992	10/10/2023	31.1863	0	0.448368	0.058146	0
1599	TF2.4	38.5301	-77.2654	SALINITY	7.3	12	11/15/1993	4/12/2021	27.42466	-0.01209	0.027073	-0.05391	0.271643
1599	TF2.4	38.5301	-77.2654	SALINITY	7.4	22	10/29/1990	9/7/2021	30.87945	0	0.085802	-0.09587	0
1599	TF2.4	38.5301	-77.2654	SALINITY	7.5	29	5/11/1992	12/12/2023	31.60822	0	0.237545	0.011852	0
1599	TF2.4	38.5301	-77.2654	SALINITY	7.6	48	3/25/1991	6/13/2023	32.2411	0	0.039482	-0.05357	0
1599	TF2.4	38.5301	-77.2654	SALINITY	7.7	49	4/8/1991	3/6/2023	31.93151	0	0.06487	-0.02007	0
1599	TF2.4	38.5301	-77.2654	SALINITY	7.8	51	10/5/1987	7/12/2021	33.79178	0	0.014936	-0.03639	0
1599	TF2.4	38.5301	-77.2654	SALINITY	7.9	46	10/15/1990	6/10/2019	28.67123	0	0.068795	-0.08052	0
1599	TF2.4	38.5301	-77.2654	SALINITY	8	89	1/6/1986	4/10/2023	37.28219	0	0.062869	-0.02168	0
1599	TF2.4	38.5301	-77.2654	SALINITY	8.1	60	2/25/1987	7/10/2023	36.39452	0	0.044162	-0.01395	0
1599	TF2.4	38.5301	-77.2654	SALINITY	8.2	51	5/2/1989	10/12/2021	32.46849	0	0.028986	-0.08524	0
1599	TF2.4	38.5301	-77.2654	SALINITY	8.3	49	6/2/1986	10/10/2019	33.37808	0	0.064226	-0.05372	0
1599	TF2.4	38.5301	-77.2654	SALINITY	8.4	28	3/3/1986	9/8/2020	34.54247	0	0.081871	-0.03398	0
1599	TF2.4	38.5301	-77.2654	SALINITY	8.5	25	4/1/1986	11/7/2023	37.6274	0	0.025914	-0.09354	0
1599	TF2.4	38.5301	-77.2654	SALINITY	8.6	21	4/28/1986	1/15/2020	33.73973	0	-0.09205	-0.60011	0
1599	TF2.4	38.5301	-77.2654	SALINITY	8.7	25	5/6/1987	9/12/2018	31.37534	0	0.076568	-0.0229	0
1599	TF2.4	38.5301	-77.2654	SALINITY	8.8	8	5/12/1986	1/17/1990	3.687671	-0.52212	0.913669	-1.08975	1.033154
1599	TF2.4	38.5301	-77.2654	SALINITY	8.9	5	3/11/1987	9/23/2008	21.55342	0.001276	0.049502	-0.31039	-0.00587
1599	TF2.4	38.5301	-77.2654	SALINITY	9	11	3/23/1987	10/2/2007	20.54247	0.046519	1.067116	0.019024	-0.13263
1599	TF2.4	38.5301	-77.2654	SALINITY	9.2	3	9/10/1990	8/14/2007	16.93699	0.886969	0.932495	0.097357	-17.5242
1600	TF3.0	38.32236	-77.5181	SALINITY	1	9	8/16/1988	8/6/1990	1.972603	0	NA	NA	0
1603	TF3.1B	38.24628	-77.2334	SALINITY	1	334	5/3/1988	2/21/2024	35.8274	0.001607	0.001615	0.001568	-0.0022
1603	TF3.1B	38.24628	-77.2334	SALINITY	2	189	5/6/2004	2/21/2024	19.80822	0	0.001506	0.000863	0.04
1603	TF3.1B	38.24628	-77.2334	SALINITY	3	322	6/1/1988	2/21/2024	35.74795	6.47E-05	0.000289	0.000226	0.038162
1603	TF3.1B	38.24628	-77.2334	SALINITY	4	34	5/3/1988	7/12/2016	28.21096	0.001649	0.002554	0.00172	0
1603	TF3.1B	38.24628	-77.2334	SALINITY	5	15	5/18/1989	10/16/2018	29.43288	0.00087	0.003408	0.000764	0.018298
1606	TF3.1E	38.24469	-77.3251	SALINITY	1	307	2/21/1991	2/21/2024	33.02192	0.001675	0.001717	0.001567	0.004236
1606	TF3.1E	38.24469	-77.3251	SALINITY	2	188	2/8/1993	2/21/2024	31.05479	0	0.001491	0.000855	0.04

1656	TSO01	38.9449	-77.0517	SALINITY	0.1	10	3/9/2010	3/3/2015	4.986301	0.027534	0.211253	-0.04009	0.339802
1657	TTX27	38.86845	-76.9691	SALINITY	0.1	8	3/8/2010	12/1/2014	4.736986	-0.02712	-0.01202	-0.0751	0.404044
1659	TUK0181	38.96713	-75.9431	SALINITY	0	5	7/18/2005	10/30/2012	7.290411	0	NA	NA	0
1659	TUK0181	38.96713	-75.9431	SALINITY	0.9	10	4/28/2006	7/9/2011	5.2	0	NA	NA	0
1659	TUK0181	38.96713	-75.9431	SALINITY	1	40	10/3/2005	9/7/2023	17.93973	0	NA	NA	0
1659	TUK0181	38.96713	-75.9431	SALINITY	1.1	31	10/27/2005	9/11/2023	17.88493	0	NA	NA	0
1659	TUK0181	38.96713	-75.9431	SALINITY	1.2	90	4/25/2006	8/15/2023	17.31781	0	NA	NA	0
1659	TUK0181	38.96713	-75.9431	SALINITY	1.3	48	3/14/2006	12/8/2022	16.74795	0	NA	NA	0
1659	TUK0181	38.96713	-75.9431	SALINITY	1.4	51	1/24/2006	4/7/2023	17.21096	0	NA	NA	0
1659	TUK0181	38.96713	-75.9431	SALINITY	1.5	24	8/22/2009	1/7/2021	11.3863	0	NA	NA	0
1659	TUK0181	38.96713	-75.9431	SALINITY	1.6	39	1/4/2006	4/30/2023	17.32877	0	NA	NA	0
1659	TUK0181	38.96713	-75.9431	SALINITY	1.7	8	10/13/2013	4/8/2022	8.490411	0	NA	NA	0
1659	TUK0181	38.96713	-75.9431	SALINITY	1.8	8	2/16/2006	8/4/2020	14.47397	0	NA	NA	0
1659	TUK0181	38.96713	-75.9431	SALINITY	1.9	3	2/24/2010	2/11/2018	7.969863	0	NA	NA	0
1659	TUK0181	38.96713	-75.9431	SALINITY	2	4	2/3/2011	10/30/2020	9.745205	0	NA	NA	0
1662	TWB01	38.90345	-76.9455	SALINITY	0.1	25	2/1/2010	11/7/2016	6.769863	-0.00527	0.003319	-0.01155	0.246237
1666	TWB05	38.894	-76.9147	SALINITY	0.1	27	2/1/2010	11/7/2016	6.769863	0.00311	0.012413	-0.00192	0.204667
1667	TWB06	38.89372	-76.9319	SALINITY	0.1	26	2/1/2010	11/7/2016	6.769863	-0.00763	-0.00127	-0.01641	0.244526
1667	TWB06	38.89372	-76.9319	SALINITY	0.14	3	2/1/2016	6/13/2016	0.364384	-0.299	-0.22601	-0.33269	2.13751
1671	WBB05	36.82917	-76.3958	SALINITY	0.3	7	2/2/2000	5/21/2002	2.29863	0.700146	2.936572	-0.77328	14.88251
1671	WBB05	36.82917	-76.3958	SALINITY	1	279	1/22/1998	1/3/2024	25.96438	-0.00817	0.025362	-0.02876	16.278
1671	WBB05	36.82917	-76.3958	SALINITY	2	173	4/27/1998	1/3/2024	25.70411	-0.06648	-0.0147	-0.1042	17.20911
1671	WBB05	36.82917	-76.3958	SALINITY	3	276	1/22/1998	1/3/2024	25.96438	-0.00436	0.035472	-0.01657	16.481
1671	WBB05	36.82917	-76.3958	SALINITY	4	188	3/19/1998	1/3/2024	25.81096	0.037832	0.085797	0.020843	15.69142
1671	WBB05	36.82917	-76.3958	SALINITY	5	51	10/22/1998	6/13/2023	24.65753	-0.09658	-0.04872	-0.14665	18.40864
1672	WBE1	36.8432	-76.3594	SALINITY	1	378	2/27/1989	1/3/2024	34.87123	0.032238	0.043534	0.01517	17.57269
1672	WBE1	36.8432	-76.3594	SALINITY	2	377	2/27/1989	1/3/2024	34.87123	0.035665	0.045491	0.01838	17.82532
1672	WBE1	36.8432	-76.3594	SALINITY	3	335	2/27/1989	1/3/2024	34.87123	0.024327	0.036622	0.009811	18.04527
1672	WBE1	36.8432	-76.3594	SALINITY	3.5	9	4/18/1989	1/27/2005	15.78904	0.400545	1.673544	-0.46106	16.15308
1672	WBE1	36.8432	-76.3594	SALINITY	4	122	2/27/1989	10/26/2023	34.68219	-0.00749	0.036684	-0.01941	17.89371
1672	WBE1	36.8432	-76.3594	SALINITY	4.5	4	5/10/1989	5/13/1992	3.010959	2.331812	2.549937	1.269953	13.22177
1672	WBE1	36.8432	-76.3594	SALINITY	5	26	2/27/1989	1/27/2016	26.93151	-0.1295	-0.06343	-0.16644	19.17154
1673	WCK0001	39.48172	-76.3405	SALINITY	0.1	154	10/27/2011	9/20/2023	11.90685	0	-0.00619	-0.08112	0
1673	WCK0001	39.48172	-76.3405	SALINITY	0.2	80	10/14/2011	7/21/2023	11.77534	0	0.171996	-0.02757	0
1674	WE4.1	37.31181	-76.3463	SALINITY	0	24	6/8/1988	3/19/1992	3.780822	-2.38031	-0.06924	-3.28041	32.60971
1674	WE4.1	37.31181	-76.3463	SALINITY	1	605	7/11/1984	2/19/2024	39.63562	-0.00953	-0.00435	-0.01827	19.60731
1674	WE4.1	37.31181	-76.3463	SALINITY	2	565	6/27/1984	2/19/2024	39.67397	0.003559	0.006639	-0.00872	19.38881
1674	WE4.1	37.31181	-76.3463	SALINITY	3	605	7/11/1984	2/19/2024	39.63562	0.083962	0.090953	0.073408	17.33258
1674	WE4.1	37.31181	-76.3463	SALINITY	4	555	6/27/1984	2/19/2024	39.67397	0	0.00465	-0.01033	19.72336
1674	WE4.1	37.31181	-76.3463	SALINITY	5	379	7/11/1984	2/19/2024	39.63562	-0.0106	-0.01166	-0.03629	20.50899
1674	WE4.1	37.31181	-76.3463	SALINITY	6	32	6/27/1984	8/7/2018	34.13425	-0.27997	-0.21347	-0.48215	22.81353
1675	WE4.2	37.24181	-76.3863	SALINITY	0	25	6/8/1988	3/19/1992	3.780822	-3.89707	-2.17478	-4.60346	39.538

1729	WT6.1	39.07851	-76.5101	SALINITY	0.5	436	11/8/1984	12/12/2023	39.11781	-0.02602	-0.01756	-0.04141	8.085668
1729	WT6.1	39.07851	-76.5101	SALINITY	1	446	11/8/1984	12/12/2023	39.11781	-0.03429	-0.02295	-0.04619	8.169274
1729	WT6.1	39.07851	-76.5101	SALINITY	2	446	11/8/1984	12/12/2023	39.11781	-0.0356	-0.02319	-0.04631	8.306354
1729	WT6.1	39.07851	-76.5101	SALINITY	3	445	11/8/1984	12/12/2023	39.11781	-0.02983	-0.02083	-0.04385	8.465067
1729	WT6.1	39.07851	-76.5101	SALINITY	3.5	6	7/10/1985	8/7/1991	6.079452	-0.52903	0.500725	-0.77208	9.651383
1729	WT6.1	39.07851	-76.5101	SALINITY	3.7	7	3/9/1987	8/9/2022	35.44384	-0.39907	0.014275	-0.50998	9.244802
1729	WT6.1	39.07851	-76.5101	SALINITY	4	405	12/3/1984	12/12/2023	39.04932	-0.00842	-0.00452	-0.03374	8.749611
1729	WT6.1	39.07851	-76.5101	SALINITY	4.1	8	3/21/1989	1/10/2002	12.81644	0.45625	0.641137	-0.263	3.50375
1729	WT6.1	39.07851	-76.5101	SALINITY	4.2	12	4/24/1985	11/13/2013	28.57534	0.111992	0.354918	0.017278	9.036642
1729	WT6.1	39.07851	-76.5101	SALINITY	4.3	20	9/8/1988	1/13/2020	31.36712	0.037799	0.174614	-0.07263	10.31779
1729	WT6.1	39.07851	-76.5101	SALINITY	4.4	24	12/11/1985	6/2/2022	36.49863	-0.00196	0.133277	-0.08157	10.85199
1729	WT6.1	39.07851	-76.5101	SALINITY	4.5	39	3/27/1985	11/15/2016	31.66027	0.011628	0.049445	-0.07231	8.5
1729	WT6.1	39.07851	-76.5101	SALINITY	4.6	44	12/10/1986	12/14/2020	34.03562	-0.00368	0.0692	-0.06349	9.787076
1729	WT6.1	39.07851	-76.5101	SALINITY	4.7	46	4/8/1986	12/12/2023	37.70411	0.069741	0.162026	0.05035	9.029425
1729	WT6.1	39.07851	-76.5101	SALINITY	4.8	42	5/22/1985	11/16/2023	38.51233	-0.05663	-0.02518	-0.10992	9.933301
1729	WT6.1	39.07851	-76.5101	SALINITY	4.9	40	8/8/1985	8/8/2023	38.02466	-0.02514	0.071765	-0.07365	10.48736
1729	WT6.1	39.07851	-76.5101	SALINITY	5	90	12/3/1984	10/17/2023	38.89589	0.046157	0.073316	-0.01114	7.145657
1729	WT6.1	39.07851	-76.5101	SALINITY	5.1	31	5/14/1986	9/13/2023	37.3589	-0.06591	0.036949	-0.12485	10.29265
1729	WT6.1	39.07851	-76.5101	SALINITY	5.2	19	8/10/1995	2/8/2021	25.51781	-0.02202	0.110958	-0.29253	9.994436
1729	WT6.1	39.07851	-76.5101	SALINITY	5.3	10	7/28/2003	7/11/2023	19.96712	-0.1724	0.404249	-0.24435	14.17016
1729	WT6.1	39.07851	-76.5101	SALINITY	5.5	6	11/18/1987	10/15/2019	31.92877	0.238165	0.288771	-0.06553	2.694671
1730	WT7.1	39.00764	-76.5035	SALINITY	0.3	5	8/8/1985	12/11/1985	0.342466	-1.54256	11.49245	-30.2323	14.96012
1730	WT7.1	39.00764	-76.5035	SALINITY	0.5	447	12/3/1984	12/12/2023	39.04932	-0.02178	-0.01146	-0.03602	9.505326
1730	WT7.1	39.00764	-76.5035	SALINITY	1	451	12/3/1984	12/12/2023	39.04932	-0.02471	-0.01648	-0.04018	9.619888
1730	WT7.1	39.00764	-76.5035	SALINITY	10	21	2/14/1989	12/12/2023	34.84658	1.125053	1.493237	0.737636	-29.1401
1730	WT7.1	39.00764	-76.5035	SALINITY	11	12	10/14/2020	10/17/2023	3.008219	1.759821	2.573258	-0.5584	-53.5144
1730	WT7.1	39.00764	-76.5035	SALINITY	11.8	3	10/14/2020	6/6/2023	2.643836	-0.92142	0.142674	-1.74493	44.82059
1730	WT7.1	39.00764	-76.5035	SALINITY	2	451	12/3/1984	12/12/2023	39.04932	-0.02288	-0.01463	-0.03812	9.707862
1730	WT7.1	39.00764	-76.5035	SALINITY	3	452	12/3/1984	12/12/2023	39.04932	-0.01927	-0.01444	-0.03836	9.901922
1730	WT7.1	39.00764	-76.5035	SALINITY	4	448	12/3/1984	12/12/2023	39.04932	-0.01748	-0.01248	-0.03559	10.12882
1730	WT7.1	39.00764	-76.5035	SALINITY	5	450	8/8/1985	12/12/2023	38.36986	-0.01776	-0.009	-0.0323	10.34829
1730	WT7.1	39.00764	-76.5035	SALINITY	6	437	8/8/1985	12/12/2023	38.36986	-0.02399	-0.01186	-0.03477	10.76142
1730	WT7.1	39.00764	-76.5035	SALINITY	6.5	3	12/11/1985	6/7/1988	2.490411	-3.12491	-0.67073	-3.44197	20.71532
1730	WT7.1	39.00764	-76.5035	SALINITY	7	419	8/8/1985	12/12/2023	38.36986	-0.01326	0.000873	-0.02644	10.76168
1730	WT7.1	39.00764	-76.5035	SALINITY	7.4	3	4/8/1987	9/24/1997	10.47123	-0.47813	0.420899	-0.58293	10.84398
1730	WT7.1	39.00764	-76.5035	SALINITY	7.5	5	11/20/1986	12/12/2013	27.07945	-0.41659	0.196839	-0.96166	6.479088
1730	WT7.1	39.00764	-76.5035	SALINITY	7.8	6	5/7/1987	12/13/2007	20.61644	-0.02567	0.089049	-0.13679	11.53223
1730	WT7.1	39.00764	-76.5035	SALINITY	7.9	3	12/7/1999	11/14/2018	18.95068	-5.07949	-0.25198	-5.27709	180.5042
1730	WT7.1	39.00764	-76.5035	SALINITY	8	367	6/11/1986	12/12/2023	37.52877	0.01767	0.022343	-0.0104	9.989336
1730	WT7.1	39.00764	-76.5035	SALINITY	8.1	9	1/6/1987	1/13/2020	33.0411	0.033393	0.211861	-0.18462	6.578406
1730	WT7.1	39.00764	-76.5035	SALINITY	8.2	13	11/15/1989	6/9/2016	26.58356	0.086203	0.125855	-1.43E-05	11.45424
1730	WT7.1	39.00764	-76.5035	SALINITY	8.3	23	9/8/1988	5/14/2019	30.69863	-0.0575	-0.02325	-0.15722	13.09884

1796	XCI4078	38.23379	-75.8696	SALINITY	2.1	5	10/17/2007	3/16/2023	15.42192	1.188444	1.787996	-0.29101	-13.3601
1796	XCI4078	38.23379	-75.8696	SALINITY	2.3	5	6/20/2007	7/13/2023	16.07397	0.267313	0.501097	0.081398	10.61583
1796	XCI4078	38.23379	-75.8696	SALINITY	2.4	4	11/14/2006	9/23/2010	3.860274	1.029126	1.909955	1.012881	7.233379
1796	XCI4078	38.23379	-75.8696	SALINITY	2.5	8	4/27/2004	8/9/2023	19.29589	0.481158	1.29896	0.106679	8.513358
1796	XCI4078	38.23379	-75.8696	SALINITY	2.6	5	4/20/2006	3/16/2022	15.91507	0.100757	0.344803	-0.09658	12.17477
1796	XCI4078	38.23379	-75.8696	SALINITY	2.7	6	5/10/2006	1/13/2022	15.69041	-0.09969	0.091399	-0.15232	13.37547
1796	XCI4078	38.23379	-75.8696	SALINITY	2.8	5	5/10/2005	4/13/2022	16.93699	0.362728	0.845851	0.087133	7.693491
1796	XCI4078	38.23379	-75.8696	SALINITY	3	101	3/12/2003	12/13/2023	20.76986	0.136199	0.187947	0.095297	9.725838
1796	XCI4078	38.23379	-75.8696	SALINITY	3.1	8	7/9/2003	12/21/2020	17.46575	-0.15392	0.231692	-0.42384	10.98843
1796	XCI4078	38.23379	-75.8696	SALINITY	3.3	8	5/22/2003	5/16/2013	9.991781	0.75324	1.069218	0.146353	8.914318
1796	XCI4078	38.23379	-75.8696	SALINITY	3.4	3	9/17/2003	8/22/2012	8.936986	3.630196	3.953039	0.205299	-23.218
1796	XCI4078	38.23379	-75.8696	SALINITY	3.5	11	3/12/2003	6/8/2022	19.25479	0.07505	0.197006	-0.49075	10.0443
1796	XCI4078	38.23379	-75.8696	SALINITY	3.6	7	10/7/2004	12/13/2023	19.19452	0.248451	0.343156	0.083606	9.038579
1796	XCI4078	38.23379	-75.8696	SALINITY	3.7	13	10/8/2003	9/12/2023	19.94247	0.152258	0.284673	0.081568	8.079098
1796	XCI4078	38.23379	-75.8696	SALINITY	3.8	9	9/22/2004	11/15/2023	19.1589	0.266627	0.383058	0.081846	9.122745
1796	XCI4078	38.23379	-75.8696	SALINITY	3.9	4	9/19/2007	7/13/2020	12.82466	-1.98692	-0.97844	-2.78036	25.14284
1796	XCI4078	38.23379	-75.8696	SALINITY	4	29	5/12/2004	6/15/2023	19.10411	-0.10562	0.172601	-0.3149	12.99233
1796	XCI4078	38.23379	-75.8696	SALINITY	4.1	3	11/24/2008	12/11/2013	5.049315	-0.14666	-0.05035	-0.53795	15.4216
1819	XDJ9007	38.48375	-75.821	SALINITY	0	174	3/12/2003	12/11/2023	20.76438	0.059266	0.083027	0.057442	0.017654
1819	XDJ9007	38.48375	-75.821	SALINITY	0.5	4	3/18/2010	9/20/2010	0.509589	0	NA	NA	0
1819	XDJ9007	38.48375	-75.821	SALINITY	2	3	3/18/2010	9/20/2010	0.509589	6.802273	11.62969	4.827419	-48.7714
1821	XDN6921	38.44902	-75.1325	SALINITY	0.1	36	8/6/2008	7/28/2020	11.98356	-0.39305	0.171344	-0.68849	19.01759
1821	XDN6921	38.44902	-75.1325	SALINITY	0.2	30	6/11/2008	11/5/2020	12.41096	-0.42528	0.328469	-0.73418	21.9819
1821	XDN6921	38.44902	-75.1325	SALINITY	0.3	56	4/30/2008	10/7/2020	12.44658	-0.12275	0.124977	-0.35271	23.1315
1821	XDN6921	38.44902	-75.1325	SALINITY	0.4	29	11/12/2008	7/20/2020	11.69315	-0.45506	-0.14479	-0.59987	24.9416
1821	XDN6921	38.44902	-75.1325	SALINITY	0.5	37	3/19/2008	9/20/2019	11.51233	0.012137	0.157893	-0.3166	21.52034
1821	XDN6921	38.44902	-75.1325	SALINITY	0.6	10	10/1/2008	6/18/2015	6.715068	-1.04751	-0.55276	-1.67255	24.99775
1821	XDN6921	38.44902	-75.1325	SALINITY	0.7	5	11/24/2009	10/21/2020	10.91507	1.06627	1.472301	-0.3467	19.83134
1830	XEF3551	38.5563	-76.4147	SALINITY	0.5	111	3/3/2011	5/24/2023	12.23288	0.147916	0.213805	0.02594	11.98676
1830	XEF3551	38.5563	-76.4147	SALINITY	1	99	7/27/2010	5/24/2023	12.83288	0.063949	0.181105	-0.06175	12.65972
1830	XEF3551	38.5563	-76.4147	SALINITY	10	121	7/27/2010	5/24/2023	12.83288	-0.0571	0.038462	-0.1023	16.5783
1830	XEF3551	38.5563	-76.4147	SALINITY	10.7	3	2/9/2011	5/14/2013	2.260274	-31.7534	0.803485	-32.6454	35.27205
1830	XEF3551	38.5563	-76.4147	SALINITY	10.8	4	8/11/2010	1/14/2021	10.43562	0.066364	0.257047	-0.3759	13.13473
1830	XEF3551	38.5563	-76.4147	SALINITY	10.9	16	9/23/2010	10/25/2022	12.09589	-0.28049	-0.00063	-0.38211	17.89884
1830	XEF3551	38.5563	-76.4147	SALINITY	11	62	7/27/2010	3/27/2023	12.67397	-0.09421	0.014789	-0.14551	17.33901
1830	XEF3551	38.5563	-76.4147	SALINITY	11.1	12	10/6/2010	5/24/2023	12.63836	0.095233	0.215281	-0.24482	13.74341
1830	XEF3551	38.5563	-76.4147	SALINITY	11.2	15	6/7/2011	11/10/2022	11.43562	0.228701	0.372259	-0.04049	15.32024
1830	XEF3551	38.5563	-76.4147	SALINITY	11.3	13	9/13/2010	1/30/2023	12.38904	-0.16099	0.009282	-0.37545	17.05845
1830	XEF3551	38.5563	-76.4147	SALINITY	11.4	13	1/16/2013	8/18/2022	9.591781	-0.15019	0.172075	-0.41505	17.55465
1830	XEF3551	38.5563	-76.4147	SALINITY	11.5	6	8/25/2010	8/7/2018	7.956164	-0.46007	0.872541	-3.05248	21.60655
1830	XEF3551	38.5563	-76.4147	SALINITY	2	123	7/27/2010	5/24/2023	12.83288	0.003204	0.093819	-0.06694	12.97685
1830	XEF3551	38.5563	-76.4147	SALINITY	3	123	7/27/2010	5/24/2023	12.83288	0.00863	0.094313	-0.06413	13.04853

46465	LIC0042	39.67628	-78.0419	SALINITY	0.4	18	10/6/2014	4/30/2023	8.569863	0	NA	NA	0
46465	LIC0042	39.67628	-78.0419	SALINITY	0.5	13	12/7/2014	12/16/2022	8.030137	0	NA	NA	0
46465	LIC0042	39.67628	-78.0419	SALINITY	0.6	14	4/20/2015	5/1/2023	8.035616	0	NA	NA	0
46465	LIC0042	39.67628	-78.0419	SALINITY	0.7	7	5/5/2017	1/25/2020	2.726027	0	NA	NA	0
46465	LIC0042	39.67628	-78.0419	SALINITY	0.8	3	2/4/2016	5/6/2017	1.252055	0	NA	NA	0
46465	LIC0042	39.67628	-78.0419	SALINITY	0.9	3	6/28/2015	3/22/2019	3.734247	0	NA	NA	0
46465	LIC0042	39.67628	-78.0419	SALINITY	1	4	2/16/2018	3/4/2023	5.046575	0	NA	NA	0
46466	SID0015	39.64935	-78.3442	SALINITY	0.3	13	10/9/2014	8/22/2023	8.873973	0	NA	NA	0
46466	SID0015	39.64935	-78.3442	SALINITY	0.4	46	11/24/2014	8/8/2023	8.709589	0	NA	NA	0
46466	SID0015	39.64935	-78.3442	SALINITY	0.5	35	10/17/2014	9/27/2023	8.950685	0	NA	NA	0
46466	SID0015	39.64935	-78.3442	SALINITY	0.6	33	12/7/2014	12/20/2022	8.041096	0	NA	NA	0
46466	SID0015	39.64935	-78.3442	SALINITY	0.7	15	1/10/2016	5/1/2023	7.309589	0	NA	NA	0
46466	SID0015	39.64935	-78.3442	SALINITY	0.8	12	2/4/2016	3/4/2023	7.082192	0	NA	NA	0
46466	SID0015	39.64935	-78.3442	SALINITY	0.9	9	4/20/2015	5/6/2019	4.046575	0	NA	NA	0
46467	TOC0037	39.70636	-78.1528	SALINITY	0.2	9	12/16/2015	2/17/2022	6.178082	0	NA	NA	0
46467	TOC0037	39.70636	-78.1528	SALINITY	0.3	39	11/5/2014	9/18/2023	8.873973	0	NA	NA	0
46467	TOC0037	39.70636	-78.1528	SALINITY	0.4	48	10/6/2014	9/27/2023	8.980822	0	NA	NA	0
46467	TOC0037	39.70636	-78.1528	SALINITY	0.5	26	10/15/2014	1/24/2023	8.282192	0	NA	NA	0
46467	TOC0037	39.70636	-78.1528	SALINITY	0.6	15	3/22/2015	3/24/2021	6.010959	0	NA	NA	0
46467	TOC0037	39.70636	-78.1528	SALINITY	0.7	10	12/7/2014	2/28/2023	8.232877	0	NA	NA	0
46467	TOC0037	39.70636	-78.1528	SALINITY	0.8	4	5/6/2017	5/1/2023	5.989041	0	NA	NA	0
46467	TOC0037	39.70636	-78.1528	SALINITY	0.9	7	10/30/2017	11/12/2022	5.038356	0	NA	NA	0
46467	TOC0037	39.70636	-78.1528	SALINITY	1	5	1/10/2016	2/4/2022	6.073973	0	NA	NA	0
46467	TOC0037	39.70636	-78.1528	SALINITY	1.1	3	1/25/2020	3/4/2023	3.106849	0	NA	NA	0
46470	XIE7136	39.28547	-76.608	SALINITY	0.5	132	5/25/2016	5/23/2023	6.99726	0.258996	0.363068	0.088929	6.222906
46470	XIE7136	39.28547	-76.608	SALINITY	1	135	5/25/2016	5/23/2023	6.99726	0.179018	0.282921	0.008006	6.649638
46470	XIE7136	39.28547	-76.608	SALINITY	1.5	4	6/14/2018	10/21/2021	3.356164	2.266067	2.69951	-3.6205	-0.30159
46470	XIE7136	39.28547	-76.608	SALINITY	2	135	5/25/2016	5/23/2023	6.99726	0.159514	0.304168	0.032644	6.96274
46470	XIE7136	39.28547	-76.608	SALINITY	3	135	5/25/2016	5/23/2023	6.99726	0.141609	0.29412	0.019436	7.339187
46470	XIE7136	39.28547	-76.608	SALINITY	4	135	5/25/2016	5/23/2023	6.99726	0.147446	0.266019	-0.004	7.77632
46470	XIE7136	39.28547	-76.608	SALINITY	5	132	5/25/2016	5/23/2023	6.99726	0.144037	0.26205	-0.0034	7.862879
46470	XIE7136	39.28547	-76.608	SALINITY	5.4	4	4/15/2019	10/20/2022	3.517808	1.840285	2.970971	1.616911	2.027052
46470	XIE7136	39.28547	-76.608	SALINITY	5.5	7	11/9/2016	1/25/2023	6.213699	-0.96065	0.123404	-1.18173	15.01119
46470	XIE7136	39.28547	-76.608	SALINITY	5.6	9	3/9/2017	9/20/2022	5.536986	0.129778	1.574268	-0.41812	7.677867
46470	XIE7136	39.28547	-76.608	SALINITY	5.7	16	2/8/2017	4/25/2023	6.210959	0.469778	0.887207	-0.13722	5.949671
46470	XIE7136	39.28547	-76.608	SALINITY	5.8	18	1/11/2017	10/6/2022	5.736986	0.272897	0.518107	-0.40374	4.894237
46470	XIE7136	39.28547	-76.608	SALINITY	5.9	22	8/30/2016	5/23/2023	6.731507	0.123131	0.567545	-0.18442	10.27783
46470	XIE7136	39.28547	-76.608	SALINITY	6	36	5/25/2016	5/9/2023	6.958904	0.049434	0.212704	-0.37628	7.592575
46470	XIE7136	39.28547	-76.608	SALINITY	6.1	12	6/7/2016	4/11/2023	6.846575	-0.50245	-0.07167	-1.00081	10.22061
46470	XIE7136	39.28547	-76.608	SALINITY	6.2	10	6/21/2016	8/6/2020	4.128767	0.812582	1.351737	-0.0527	4.636711
46470	XIE7136	39.28547	-76.608	SALINITY	6.3	9	5/25/2016	9/23/2021	5.334247	-1.0639	-0.4712	-1.78467	10.93588
46470	XIE7136	39.28547	-76.608	SALINITY	6.4	4	8/16/2016	6/16/2022	5.835616	0.04324	0.162536	-0.77668	6.877833

46521	XDA8236	38.46978	-77.2728	SALINITY	5	40	4/19/2018	5/23/2023	5.09589	0	0.728763	0.313856	0
46521	XDA8236	38.46978	-77.2728	SALINITY	5.1	8	8/12/2020	9/2/2021	1.057534	0	0.803791	-0.38501	0
46521	XDA8236	38.46978	-77.2728	SALINITY	5.2	12	5/3/2018	5/9/2023	5.019178	0	1.468171	0.610095	0
46521	XDA8236	38.46978	-77.2728	SALINITY	5.3	8	4/19/2018	5/23/2023	5.09589	0	0.246209	0.246209	0
46521	XDA8236	38.46978	-77.2728	SALINITY	5.4	5	5/23/2018	9/2/2022	4.282192	0.63435	0.93599	0.302415	-0.20983
46521	XDA8236	38.46978	-77.2728	SALINITY	5.5	6	7/25/2018	10/21/2020	2.243836	0.061603	1.317356	0.038624	-0.04253
46521	XDA8236	38.46978	-77.2728	SALINITY	5.7	4	9/27/2018	12/22/2022	4.238356	0.63186	1.139631	0.587492	-0.49099
46522	XDF0255	38.33645	-76.4083	SALINITY	0.1	7	7/18/2018	8/6/2020	2.054795	0.635309	2.316793	-3.78263	8.085255
46522	XDF0255	38.33645	-76.4083	SALINITY	0.2	3	7/3/2018	9/3/2020	2.172603	7.515923	7.998293	2.610694	-4.81621
46522	XDF0255	38.33645	-76.4083	SALINITY	0.3	9	4/30/2018	9/30/2020	2.421918	3.536419	4.648889	-0.19059	4.715153
46522	XDF0255	38.33645	-76.4083	SALINITY	0.4	5	4/11/2018	6/10/2020	2.167123	1.763823	4.511708	-2.6696	6.628663
46522	XDF0255	38.33645	-76.4083	SALINITY	0.5	6	4/24/2019	7/8/2020	1.208219	11.11485	19.28364	6.192498	-3.57072
46522	XDF0255	38.33645	-76.4083	SALINITY	0.6	5	11/6/2018	10/15/2020	1.942466	4.049792	5.476907	3.425136	3.795163
46523	XDG2324	38.3715	-76.2932	SALINITY	0.5	20	4/11/2018	10/6/2020	2.490411	2.328979	2.862919	0.858777	7.02313
46523	XDG2324	38.3715	-76.2932	SALINITY	1	20	4/11/2018	10/6/2020	2.490411	2.37171	3.114695	0.689659	6.908276
46523	XDG2324	38.3715	-76.2932	SALINITY	2	20	4/11/2018	10/6/2020	2.490411	2.366042	3.127004	0.685468	6.919825
46523	XDG2324	38.3715	-76.2932	SALINITY	3	20	4/11/2018	10/6/2020	2.490411	2.328979	3.035297	0.706681	7.077123
46523	XDG2324	38.3715	-76.2932	SALINITY	4	19	4/11/2018	10/6/2020	2.490411	2.830409	3.422981	1.355635	6.897985
46523	XDG2324	38.3715	-76.2932	SALINITY	4.4	3	9/26/2018	7/14/2020	1.8	2.811383	3.178254	2.477574	7.115136
46523	XDG2324	38.3715	-76.2932	SALINITY	4.6	3	8/15/2018	9/24/2020	2.112329	4.524396	4.612377	3.401059	6.184
46523	XDG2324	38.3715	-76.2932	SALINITY	5	4	6/4/2019	6/25/2020	1.060274	19.07955	20.20707	7.702082	-12.965
46524	XDG5922	38.43147	-76.2971	SALINITY	0.5	33	7/31/2018	10/30/2019	1.249315	5.253788	9.912229	3.599506	3.676667
46524	XDG5922	38.43147	-76.2971	SALINITY	1	25	8/13/2018	10/30/2019	1.213699	16.0959	17.78295	9.437465	-5.86049
46524	XDG5922	38.43147	-76.2971	SALINITY	1.1	4	7/31/2018	4/23/2019	0.728767	-2.41706	1.494065	-2.5797	10.62
46524	XDG5922	38.43147	-76.2971	SALINITY	1.2	5	8/13/2018	6/18/2019	0.846575	0.283243	1.767511	0.122278	7.569437
46524	XDG5922	38.43147	-76.2971	SALINITY	1.3	7	9/27/2018	10/30/2019	1.090411	4.99289	13.14779	0.309804	2.529838
46524	XDG5922	38.43147	-76.2971	SALINITY	1.4	4	11/1/2018	9/11/2019	0.860274	14.5131	18.74635	9.990842	-4.1
46524	XDG5922	38.43147	-76.2971	SALINITY	1.5	6	9/20/2018	10/22/2019	1.087671	14.82813	19.87624	5.62261	-3.045
46524	XDG5922	38.43147	-76.2971	SALINITY	1.6	3	8/6/2019	10/9/2019	0.175342	35.90268	37.2787	30.50445	-27.0094
46524	XDG5922	38.43147	-76.2971	SALINITY	0.5	33	7/31/2018	10/30/2019	1.249315	5.253788	9.912229	3.599506	3.676667
46524	XDG5922	38.43147	-76.2971	SALINITY	1	25	8/13/2018	10/30/2019	1.213699	16.0959	17.78295	9.437465	-5.86049
46524	XDG5922	38.43147	-76.2971	SALINITY	1.1	4	7/31/2018	4/23/2019	0.728767	-2.41706	1.494065	-2.5797	10.62
46524	XDG5922	38.43147	-76.2971	SALINITY	1.2	5	8/13/2018	6/18/2019	0.846575	0.283243	1.767511	0.122278	7.569437
46524	XDG5922	38.43147	-76.2971	SALINITY	1.3	7	9/27/2018	10/30/2019	1.090411	4.99289	13.14779	0.309804	2.529838
46524	XDG5922	38.43147	-76.2971	SALINITY	1.4	4	11/1/2018	9/11/2019	0.860274	14.5131	18.74635	9.990842	-4.1
46524	XDG5922	38.43147	-76.2971	SALINITY	1.5	6	9/20/2018	10/22/2019	1.087671	14.82813	19.87624	5.62261	-3.045
46524	XDG5922	38.43147	-76.2971	SALINITY	1.6	3	8/6/2019	10/9/2019	0.175342	35.90268	37.2787	30.50445	-27.0094
46529	NWA.COBR1	38.6422	-75.6068	SALINITY	0.2	6	4/8/2018	11/3/2019	1.572603	0	NA	NA	0.1
46529	NWA.COBR1	38.6422	-75.6068	SALINITY	0.3	12	3/25/2018	7/19/2021	3.320548	0	NA	NA	0.1
46529	NWA.COBR1	38.6422	-75.6068	SALINITY	0.5	78	5/20/2018	11/7/2022	4.471233	0	0.001967	0.001967	0.1
48017	MDE.0306006	38.93556	-76.5231	SALINITY	0.5	17	4/4/2019	10/20/2021	2.547945	0.228094	1.252451	-0.6195	7.486386
48017	MDE.0306006	38.93556	-76.5231	SALINITY	1	17	4/4/2019	10/20/2021	2.547945	0.175717	1.108522	-0.8007	7.740766

48080	NWA.MANA5	38.3096	-75.8929	SALINITY	0.7	3	11/5/2017	10/10/2021	3.931507	-0.45784	NA	NA	13.67972
48082	NWA.QUCR1	38.3664	-75.7722	SALINITY	0.3	3	10/10/2022	10/24/2022	0.038356	-5.21429	NA	NA	29.31429
48082	NWA.QUCR1	38.3664	-75.7722	SALINITY	0.5	115	3/26/2017	11/7/2022	5.621918	0	0.23737	0.044979	0.1
48083	NWA.RECR1	38.4016	-75.7905	SALINITY	0.5	122	3/27/2017	11/6/2022	5.616438	0.289829	0.368203	0.239827	0.200862
48083	NWA.RECR1	38.4016	-75.7905	SALINITY	0.9	3	5/8/2017	5/24/2021	4.046575	-0.07595	-0.06035	-0.08974	0.57187
48083	NWA.RECR1	38.4016	-75.7905	SALINITY	1	10	3/27/2017	8/15/2021	4.389041	0	0.162429	-0.03503	0.221477
48083	NWA.RECR1	38.4016	-75.7905	SALINITY	1.1	11	6/17/2019	8/15/2022	3.164384	0.248299	0.696415	-0.44622	0.831236
48083	NWA.RECR1	38.4016	-75.7905	SALINITY	1.2	15	8/27/2018	10/10/2022	4.123288	0.152242	0.537999	0.077223	-0.13942
48083	NWA.RECR1	38.4016	-75.7905	SALINITY	1.3	13	10/23/2017	6/5/2022	4.619178	-0.52958	-0.04659	-0.71562	2.904688
48083	NWA.RECR1	38.4016	-75.7905	SALINITY	1.4	14	5/22/2017	11/6/2022	5.463014	0.023488	0.962612	-0.237	0.053153
48083	NWA.RECR1	38.4016	-75.7905	SALINITY	1.5	18	7/3/2017	10/24/2022	5.312329	0.397018	0.527337	0.007081	0.211062
48083	NWA.RECR1	38.4016	-75.7905	SALINITY	1.6	14	8/14/2017	8/29/2022	5.043836	1.142235	2.479159	0.802163	-0.34615
48083	NWA.RECR1	38.4016	-75.7905	SALINITY	1.7	16	7/17/2017	9/25/2022	5.194521	0.391451	1.042126	0.100931	3.121026
48083	NWA.RECR1	38.4016	-75.7905	SALINITY	1.8	5	6/19/2017	7/18/2022	5.082192	0.231664	0.606755	0.092122	3.144712
48084	NWA.RECR2c	38.4106	-75.7544	SALINITY	0.2	3	4/20/2020	3/28/2022	1.936986	0	NA	NA	0.1
48084	NWA.RECR2c	38.4106	-75.7544	SALINITY	0.3	6	4/21/2019	10/24/2022	3.512329	0	0.089811	0.089811	0.1
48084	NWA.RECR2c	38.4106	-75.7544	SALINITY	0.5	83	3/25/2018	11/7/2022	4.624658	0	0.090693	-0.09114	0.1
48087	NWA.TRAN2	38.51269	-75.9683	SALINITY	0.5	78	6/4/2018	11/7/2021	3.430137	0	-0.06531	-0.41742	0.1
48087	NWA.TRAN2	38.51269	-75.9683	SALINITY	0.7	18	7/16/2018	10/24/2021	3.276712	0	-0.09177	-0.47035	0.1
48087	NWA.TRAN2	38.51269	-75.9683	SALINITY	0.8	24	6/4/2018	11/7/2021	3.430137	0	NA	NA	0.1
48087	NWA.TRAN2	38.51269	-75.9683	SALINITY	0.9	8	8/13/2018	9/26/2021	3.123288	0.434524	1.91779	0.434524	-0.35
48087	NWA.TRAN2	38.51269	-75.9683	SALINITY	1	6	4/23/2019	11/2/2020	1.531507	0	NA	NA	0.1
48088	NWA.TYCR1	38.32006	-75.8591	SALINITY	0.3	9	7/15/2019	8/14/2022	3.084932	5.188127	9.477317	3.082916	-17.8871
48088	NWA.TYCR1	38.32006	-75.8591	SALINITY	0.5	99	3/27/2017	11/7/2022	5.619178	0.741731	0.972175	0.613805	4.966667
48088	NWA.TYCR1	38.32006	-75.8591	SALINITY	0.6	6	7/3/2017	5/7/2018	0.843836	-0.60421	4.068214	-1.58132	7.197247
48088	NWA.TYCR1	38.32006	-75.8591	SALINITY	0.8	3	6/19/2017	11/5/2017	0.380822	-1.11991	7.801899	-4.98296	7.586598
48089	NWA.WECR1	38.3267	-75.8665	SALINITY	0.5	109	4/10/2017	11/7/2022	5.580822	0.457204	0.754337	0.416855	5.466406
48089	NWA.WECR1	38.3267	-75.8665	SALINITY	1	105	4/10/2017	10/23/2022	5.539726	0.268778	0.553628	0.219281	5.9
48089	NWA.WECR1	38.3267	-75.8665	SALINITY	2	105	4/10/2017	10/23/2022	5.539726	0.284498	0.536792	0.196942	6.022169
48089	NWA.WECR1	38.3267	-75.8665	SALINITY	2.3	3	6/3/2018	11/7/2021	3.432877	2.068236	NA	NA	-1.57422
48089	NWA.WECR1	38.3267	-75.8665	SALINITY	2.6	3	9/25/2017	7/4/2022	4.775342	-1.26365	0.478546	-1.65843	6.896402
48089	NWA.WECR1	38.3267	-75.8665	SALINITY	2.8	3	6/4/2017	6/19/2022	5.043836	0.753627	1.009918	-0.19681	2.192578
48089	NWA.WECR1	38.3267	-75.8665	SALINITY	2.9	4	3/26/2018	6/7/2021	3.20274	-0.52143	-0.20695	-0.92471	7.5
48089	NWA.WECR1	38.3267	-75.8665	SALINITY	3	95	4/10/2017	10/23/2022	5.539726	0.32196	0.571337	0.214984	6.315908
48089	NWA.WECR1	38.3267	-75.8665	SALINITY	3.1	3	7/3/2017	4/12/2021	3.778082	-0.70517	-0.32649	-1.14626	6.395801
48089	NWA.WECR1	38.3267	-75.8665	SALINITY	3.2	3	4/8/2018	8/2/2021	3.320548	5.348791	5.968787	-0.74046	-16.4391
48089	NWA.WECR1	38.3267	-75.8665	SALINITY	3.3	8	6/17/2019	7/17/2022	3.084932	0.583764	2.282945	-2.67678	6.163941
48089	NWA.WECR1	38.3267	-75.8665	SALINITY	3.4	10	9/11/2017	9/25/2022	5.041096	-0.07562	0.973959	-0.6896	5.551
48089	NWA.WECR1	38.3267	-75.8665	SALINITY	3.5	20	7/31/2017	5/22/2022	4.810959	1.097288	1.513336	-0.12682	2.399743
48089	NWA.WECR1	38.3267	-75.8665	SALINITY	3.6	8	4/22/2019	6/6/2022	3.126027	0.86202	4.429884	-0.38417	4.002923
48089	NWA.WECR1	38.3267	-75.8665	SALINITY	3.7	4	5/20/2019	10/23/2022	3.430137	1.273719	3.796605	0.798932	5.143948
48089	NWA.WECR1	38.3267	-75.8665	SALINITY	3.8	4	8/26/2019	9/8/2020	1.038356	0.551511	1.672125	1.672125	6.588462

48421	CHE006.36	36.92375	-76.0518	SALINITY	1.75	4	7/22/2019	5/13/2020	0.810959	3.401136	8.620394	-0.5876	21.8925
48423	WES002.98	36.85732	-76.1203	SALINITY	0.1	27	3/12/2019	11/9/2021	2.665753	2.563022	3.205697	1.745417	12.97401
48423	WES002.98	36.85732	-76.1203	SALINITY	0.25	27	3/12/2019	11/9/2021	2.665753	2.55813	3.169791	1.766287	13.07403
48423	WES002.98	36.85732	-76.1203	SALINITY	0.5	27	3/12/2019	11/9/2021	2.665753	2.594752	3.14632	1.815288	13.13096
48423	WES002.98	36.85732	-76.1203	SALINITY	0.75	25	3/12/2019	11/9/2021	2.665753	2.557736	3.099872	1.666337	12.98871
48423	WES002.98	36.85732	-76.1203	SALINITY	1	21	4/8/2019	11/9/2021	2.591781	1.691446	2.887235	1.468011	13.22351
48423	WES002.98	36.85732	-76.1203	SALINITY	1.25	15	7/18/2019	11/9/2021	2.315068	1.28958	2.684371	0.199378	13.71305
48423	WES002.98	36.85732	-76.1203	SALINITY	1.5	6	7/18/2019	9/21/2021	2.180822	2.259102	3.537753	1.563398	15.53141
48423	WES002.98	36.85732	-76.1203	SALINITY	0.1	27	3/12/2019	11/9/2021	2.665753	2.563022	3.205697	1.745417	12.97401
48423	WES002.98	36.85732	-76.1203	SALINITY	0.25	27	3/12/2019	11/9/2021	2.665753	2.55813	3.169791	1.766287	13.07403
48423	WES002.98	36.85732	-76.1203	SALINITY	0.5	27	3/12/2019	11/9/2021	2.665753	2.594752	3.14632	1.815288	13.13096
48423	WES002.98	36.85732	-76.1203	SALINITY	0.75	25	3/12/2019	11/9/2021	2.665753	2.557736	3.099872	1.666337	12.98871
48423	WES002.98	36.85732	-76.1203	SALINITY	1	21	4/8/2019	11/9/2021	2.591781	1.691446	2.887235	1.468011	13.22351
48423	WES002.98	36.85732	-76.1203	SALINITY	1.25	15	7/18/2019	11/9/2021	2.315068	1.28958	2.684371	0.199378	13.71305
48423	WES002.98	36.85732	-76.1203	SALINITY	1.5	6	7/18/2019	9/21/2021	2.180822	2.259102	3.537753	1.563398	15.53141
48424	WES001.66	36.87361	-76.107	SALINITY	0.1	27	3/12/2019	11/9/2021	2.665753	1.786966	2.349285	1.09632	16.23444
48424	WES001.66	36.87361	-76.107	SALINITY	0.25	27	3/12/2019	11/9/2021	2.665753	1.724793	2.348508	1.087005	16.19757
48424	WES001.66	36.87361	-76.107	SALINITY	0.5	27	3/12/2019	11/9/2021	2.665753	2.05424	2.367097	1.109857	16.17353
48424	WES001.66	36.87361	-76.107	SALINITY	0.75	27	3/12/2019	11/9/2021	2.665753	1.978802	2.419914	1.169205	16.14807
48424	WES001.66	36.87361	-76.107	SALINITY	1	23	3/12/2019	11/9/2021	2.665753	2.107938	2.594515	1.102419	16.40286
48424	WES001.66	36.87361	-76.107	SALINITY	1.25	20	3/12/2019	11/9/2021	2.665753	1.287272	2.423698	0.244342	19.20895
48424	WES001.66	36.87361	-76.107	SALINITY	1.5	9	3/12/2019	9/21/2021	2.531507	-0.92836	3.762353	-2.039	19.78449
48425	EBL000.00	36.88651	-76.0746	SALINITY	0.1	27	3/12/2019	11/9/2021	2.665753	1.790788	2.130907	0.755067	17.6459
48425	EBL000.00	36.88651	-76.0746	SALINITY	0.25	27	3/12/2019	11/9/2021	2.665753	1.836294	2.164816	0.754502	17.52967
48425	EBL000.00	36.88651	-76.0746	SALINITY	0.5	27	3/12/2019	11/9/2021	2.665753	1.819519	2.157744	0.732473	17.58217
48425	EBL000.00	36.88651	-76.0746	SALINITY	0.75	27	3/12/2019	11/9/2021	2.665753	1.616505	1.931236	0.625182	18.76698
48425	EBL000.00	36.88651	-76.0746	SALINITY	1	26	3/12/2019	11/9/2021	2.665753	1.463671	1.951866	0.741946	18.82138
48425	EBL000.00	36.88651	-76.0746	SALINITY	1.25	23	3/12/2019	11/9/2021	2.665753	1.211477	1.707409	0.541206	19.30796
48425	EBL000.00	36.88651	-76.0746	SALINITY	1.5	14	4/8/2019	11/9/2021	2.591781	0.858532	1.705776	0.183672	19.41287
48425	EBL000.00	36.88651	-76.0746	SALINITY	1.75	4	6/5/2019	4/9/2020	0.846575	0.973333	7.500862	-1.67098	23.72067
48426	EBL002.52	36.8553	-76.0627	SALINITY	0.1	27	3/12/2019	11/9/2021	2.665753	2.130813	3.374108	1.638442	12.90254
48426	EBL002.52	36.8553	-76.0627	SALINITY	0.25	27	3/12/2019	11/9/2021	2.665753	1.918432	3.250209	1.501872	13.54514
48426	EBL002.52	36.8553	-76.0627	SALINITY	0.5	27	3/12/2019	11/9/2021	2.665753	2.234875	3.208356	1.567781	13.60484
48426	EBL002.52	36.8553	-76.0627	SALINITY	0.75	25	3/12/2019	11/9/2021	2.665753	2.256571	3.291395	1.570807	14.226
48426	EBL002.52	36.8553	-76.0627	SALINITY	1	20	3/12/2019	11/9/2021	2.665753	2.099098	3.176931	0.772782	14.94466
48426	EBL002.52	36.8553	-76.0627	SALINITY	1.25	13	3/12/2019	11/9/2021	2.665753	-0.02248	2.782152	-0.9146	17.20529
48426	EBL002.52	36.8553	-76.0627	SALINITY	1.5	7	3/12/2019	3/15/2021	2.010959	-2.93712	4.928909	-3.38465	21.286
48426	EBL002.52	36.8553	-76.0627	SALINITY	1.75	4	10/18/2019	3/15/2021	1.408219	-3.1059	-2.82987	-4.52284	22.06584
48427	LYN001.98	36.89796	-76.0852	SALINITY	0.1	27	3/12/2019	11/9/2021	2.665753	1.309786	1.774433	0.538913	19.06701
48427	LYN001.98	36.89796	-76.0852	SALINITY	0.25	27	3/12/2019	11/9/2021	2.665753	1.364798	1.784809	0.534283	19.06701
48427	LYN001.98	36.89796	-76.0852	SALINITY	0.5	27	3/12/2019	11/9/2021	2.665753	1.411936	1.783565	0.54336	19.0712
48427	LYN001.98	36.89796	-76.0852	SALINITY	0.75	27	3/12/2019	11/9/2021	2.665753	1.415794	1.784856	0.551668	19.0654

56959	BWB.BWB-PATMH-07	39.25454	-76.5738	SALINITY	2	20	4/18/2018	11/2/2022	4.545205	1.214105	2.129212	0.631346	2.031179
56959	BWB.BWB-PATMH-07	39.25454	-76.5738	SALINITY	2.99	3	4/18/2018	8/21/2019	1.342466	-7.60066	1.023394	-8.71344	4.886129
56959	BWB.BWB-PATMH-07	39.25454	-76.5738	SALINITY	3	16	6/12/2019	11/2/2022	3.394521	1.070779	3.026661	-0.18682	2.954079
56959	BWB.BWB-PATMH-07	39.25454	-76.5738	SALINITY	4	17	4/25/2019	11/2/2022	3.526027	1.113961	2.967484	-0.04018	3.106158
56959	BWB.BWB-PATMH-07	39.25454	-76.5738	SALINITY	4.01	3	6/13/2018	8/21/2019	1.189041	-3.79243	3.46342	-4.86737	3.473704
56959	BWB.BWB-PATMH-07	39.25454	-76.5738	SALINITY	4.98	3	6/12/2019	10/9/2019	0.326027	4.032381	20.9328	1.778992	5.96
56959	BWB.BWB-PATMH-07	39.25454	-76.5738	SALINITY	5	15	4/18/2018	11/2/2022	4.545205	0.752925	3.11295	-0.11749	4.601983
56959	BWB.BWB-PATMH-07	39.25454	-76.5738	SALINITY	5.05	3	8/8/2018	7/24/2019	0.958904	5.866071	5.928643	5.537571	0.83
56959	BWB.BWB-PATMH-07	39.25454	-76.5738	SALINITY	5.97	3	6/13/2018	10/9/2019	1.323288	8.320443	10.19253	2.594064	-3.57421
56959	BWB.BWB-PATMH-07	39.25454	-76.5738	SALINITY	6	14	7/11/2018	11/2/2022	4.315068	0.923469	3.251485	0.415013	5.565601
56959	BWB.BWB-PATMH-07	39.25454	-76.5738	SALINITY	6.03	3	8/8/2018	9/25/2019	1.131507	8.837447	8.929464	8.25084	0.092353
56959	BWB.BWB-PATMH-07	39.25454	-76.5738	SALINITY	7	12	11/6/2019	10/6/2022	2.917808	0.135862	3.003064	-1.3325	8.316486
56959	BWB.BWB-PATMH-07	39.25454	-76.5738	SALINITY	8	12	4/25/2019	8/3/2022	3.276712	1.602074	2.428154	0.464913	4.699416
56959	BWB.BWB-PATMH-07	39.25454	-76.5738	SALINITY	9	8	8/7/2019	8/3/2022	2.991781	2.778413	3.785837	0.188185	-0.20797
56959	BWB.BWB-PATMH-07	39.25454	-76.5738	SALINITY	9.05	3	9/12/2018	11/6/2019	1.150685	-5.99643	-0.44321	-6.61345	21.365
56960	BWB.BWB-PATMH-08	39.23534	-76.556	SALINITY	0.5	22	4/18/2018	11/2/2022	4.545205	1.054914	1.401646	0.775334	2.92744
56960	BWB.BWB-PATMH-08	39.23534	-76.556	SALINITY	0.97	3	8/8/2018	10/9/2019	1.169863	12.47699	15.46209	4.750848	-11.3471
56960	BWB.BWB-PATMH-08	39.23534	-76.556	SALINITY	1	28	9/12/2018	11/2/2022	4.142466	1.282212	2.199862	1.153106	1.99275
56960	BWB.BWB-PATMH-08	39.23534	-76.556	SALINITY	1.01	3	4/18/2018	7/18/2018	0.249315	-0.6583	8.338846	-15.0537	3.151
56960	BWB.BWB-PATMH-08	39.23534	-76.556	SALINITY	1.5	12	5/4/2021	11/2/2022	1.49863	6.159191	10.5977	3.624579	-14.76
56960	BWB.BWB-PATMH-08	39.23534	-76.556	SALINITY	2	19	10/3/2018	11/2/2022	4.084932	1.746275	4.660765	1.233487	0.480613
56960	BWB.BWB-PATMH-08	39.23534	-76.556	SALINITY	2.01	4	5/9/2018	7/24/2019	1.208219	0.521429	0.669029	-1.25233	5.21
56960	BWB.BWB-PATMH-08	39.23534	-76.556	SALINITY	2.04	3	5/15/2019	11/6/2019	0.479452	16.31136	17.38202	15.32305	-15.6818
56960	BWB.BWB-PATMH-08	39.23534	-76.556	SALINITY	2.5	12	5/9/2018	11/2/2022	4.487671	3.558837	5.30175	0.910941	-4.18814
56960	BWB.BWB-PATMH-08	39.23534	-76.556	SALINITY	2.99	4	4/18/2018	7/24/2019	1.265753	-1.13161	0.260658	-1.5783	5.712128
56960	BWB.BWB-PATMH-08	39.23534	-76.556	SALINITY	3	21	6/13/2018	11/2/2022	4.391781	1.462446	3.443382	0.763401	2.430439
56960	BWB.BWB-PATMH-08	39.23534	-76.556	SALINITY	3.5	11	5/4/2021	11/2/2022	1.49863	5.232661	7.542293	3.009858	-10.3735
56960	BWB.BWB-PATMH-08	39.23534	-76.556	SALINITY	3.52	3	6/13/2018	11/6/2019	1.4	7.245467	10.21414	1.54561	-5.13143
56960	BWB.BWB-PATMH-08	39.23534	-76.556	SALINITY	3.98	4	7/18/2018	11/6/2019	1.30411	-3.91071	0.29334	-5.64887	16.825
56960	BWB.BWB-PATMH-08	39.23534	-76.556	SALINITY	4	20	4/25/2019	11/2/2022	3.526027	1.927848	3.895219	1.173124	0.36518
56960	BWB.BWB-PATMH-08	39.23534	-76.556	SALINITY	4.5	12	7/18/2018	11/2/2022	4.29589	1.21184	5.250544	0.658912	4.189325
56960	BWB.BWB-PATMH-08	39.23534	-76.556	SALINITY	5	17	6/13/2018	11/2/2022	4.391781	2.755722	6.352992	1.780795	-2.89536
56960	BWB.BWB-PATMH-08	39.23534	-76.556	SALINITY	5.01	3	7/18/2018	4/25/2019	0.769863	-5.80043	-3.47289	-6.67896	6.419786
56960	BWB.BWB-PATMH-08	39.23534	-76.556	SALINITY	5.5	6	5/4/2021	10/20/2022	1.463014	1.440789	5.218907	-1.03983	2.382632
56960	BWB.BWB-PATMH-08	39.23534	-76.556	SALINITY	6	10	4/25/2019	5/18/2022	3.065753	1.200627	1.795875	-0.75037	4.988199
56960	BWB.BWB-PATMH-08	39.23534	-76.556	SALINITY	6.06	3	5/9/2018	8/7/2019	1.246575	-2.87237	1.671124	-5.04086	5.312078
56961	BWB.BWB-PATMH-09	39.21309	-76.5225	SALINITY	0.5	20	6/6/2018	11/9/2022	4.430137	1.985146	2.279506	1.477468	1.284117
56961	BWB.BWB-PATMH-09	39.21309	-76.5225	SALINITY	0.99	4	7/26/2018	9/18/2019	1.147945	2.429701	10.94821	2.393674	1.110985
56961	BWB.BWB-PATMH-09	39.21309	-76.5225	SALINITY	1	20	4/18/2018	11/9/2022	4.564384	1.268853	1.815648	1.01132	2.598227
56961	BWB.BWB-PATMH-09	39.21309	-76.5225	SALINITY	1.5	12	5/22/2019	11/9/2022	3.471233	3.900071	4.922045	2.517333	-6.10447
56961	BWB.BWB-PATMH-09	39.21309	-76.5225	SALINITY	1.52	3	7/26/2018	9/18/2019	1.147945	5.779528	5.970339	5.340811	0.781443
56961	BWB.BWB-PATMH-09	39.21309	-76.5225	SALINITY	2	12	6/26/2019	11/9/2022	3.375342	4.07562	4.725506	2.29374	-6.25328

56971	BWB.BWB-PATMH-19	39.25579	-76.6101	SALINITY	2	15	9/12/2018	11/2/2022	4.142466	1.451058	2.169961	0.422162	2.471504
56971	BWB.BWB-PATMH-19	39.25579	-76.6101	SALINITY	2.02	3	5/9/2018	5/30/2019	1.057534	-2.70844	-0.88016	-3.83294	4.158565
56971	BWB.BWB-PATMH-19	39.25579	-76.6101	SALINITY	2.5	13	7/18/2018	11/2/2022	4.29589	0.776834	2.787324	0.157318	4.012195
56971	BWB.BWB-PATMH-19	39.25579	-76.6101	SALINITY	2.52	4	5/9/2018	5/30/2019	1.057534	-3.76587	-2.52866	-4.96185	5.678889
56971	BWB.BWB-PATMH-19	39.25579	-76.6101	SALINITY	3	8	5/15/2019	10/6/2022	3.39726	1.65226	4.676527	0.875798	2.09066
56971	BWB.BWB-PATMH-19	39.25579	-76.6101	SALINITY	3.5	9	4/25/2019	10/6/2022	3.452055	2.202389	3.93432	1.56048	1.265929
56971	BWB.BWB-PATMH-19	39.25579	-76.6101	SALINITY	4	7	5/9/2018	10/6/2022	4.413699	1.167332	3.95791	-0.54316	3.223727
56971	BWB.BWB-PATMH-19	39.25579	-76.6101	SALINITY	4.5	5	5/9/2018	10/13/2021	3.432877	0.506566	1.486275	-14.4766	5.337317
56971	BWB.BWB-PATMH-19	39.25579	-76.6101	SALINITY	4.53	3	9/12/2018	8/7/2019	0.90137	-19.3711	0.773267	-20.2664	32.3405
56971	BWB.BWB-PATMH-19	39.25579	-76.6101	SALINITY	5	4	5/9/2018	10/13/2021	3.432877	0.261146	0.32917	-2.41524	6.329967
56971	BWB.BWB-PATMH-19	39.25579	-76.6101	SALINITY	5.03	4	7/18/2018	8/7/2019	1.054795	0.762466	2.823112	2.823112	5.735283
56971	BWB.BWB-PATMH-19	39.25579	-76.6101	SALINITY	5.06	3	6/13/2018	10/9/2019	1.323288	6.486342	6.964588	4.426205	3.533297
56971	BWB.BWB-PATMH-19	39.25579	-76.6101	SALINITY	5.5	5	5/9/2018	10/13/2021	3.432877	0.29607	0.574995	-1.9865	6.067442
56972	BWB.BWB-PATMH-20	39.25467	-76.5946	SALINITY	0.5	22	4/11/2018	11/2/2022	4.564384	1.412774	1.875682	0.914963	0.991182
56972	BWB.BWB-PATMH-20	39.25467	-76.5946	SALINITY	1	20	6/13/2018	11/2/2022	4.391781	2.234661	2.563247	1.626895	0.279626
56972	BWB.BWB-PATMH-20	39.25467	-76.5946	SALINITY	1.5	14	5/9/2018	11/2/2022	4.487671	2.077609	3.454912	1.217158	-0.38736
56972	BWB.BWB-PATMH-20	39.25467	-76.5946	SALINITY	1.99	3	7/18/2018	10/9/2019	1.227397	6.628211	10.22718	0.572458	-4.24244
56972	BWB.BWB-PATMH-20	39.25467	-76.5946	SALINITY	2	13	7/24/2019	11/2/2022	3.279452	1.580087	3.975707	0.423125	0.899697
56972	BWB.BWB-PATMH-20	39.25467	-76.5946	SALINITY	2.03	4	4/11/2018	8/21/2019	1.361644	-0.23865	1.514175	-3.39465	3.110692
56972	BWB.BWB-PATMH-20	39.25467	-76.5946	SALINITY	2.5	11	5/4/2021	11/2/2022	1.49863	1.495263	5.428103	0.263666	1.899739
56972	BWB.BWB-PATMH-20	39.25467	-76.5946	SALINITY	3	13	7/18/2018	11/2/2022	4.29589	1.027881	3.705469	0.375191	3.677679
56972	BWB.BWB-PATMH-20	39.25467	-76.5946	SALINITY	3.03	3	4/11/2018	9/25/2019	1.457534	10.78657	12.34962	-0.84952	-6.64
56972	BWB.BWB-PATMH-20	39.25467	-76.5946	SALINITY	3.05	3	9/12/2018	11/6/2019	1.150685	-5.97356	5.854179	-6.59607	6.164035
56972	BWB.BWB-PATMH-20	39.25467	-76.5946	SALINITY	3.5	9	8/8/2018	10/6/2022	4.164384	1.715628	5.107264	0.511458	0.775522
56972	BWB.BWB-PATMH-20	39.25467	-76.5946	SALINITY	4	6	10/9/2019	8/3/2022	2.819178	-0.93523	0.362389	-1.73143	11.753
56972	BWB.BWB-PATMH-20	39.25467	-76.5946	SALINITY	4.5	3	9/25/2019	6/1/2022	2.684932	-5.10475	-0.80925	-6.37749	30.81347
56973	BWB.BWB-PATMH-21	39.19738	-76.5746	SALINITY	0.5	15	4/25/2018	11/9/2022	4.545205	1.827096	2.140617	1.303827	2.544191
56973	BWB.BWB-PATMH-21	39.19738	-76.5746	SALINITY	1	24	6/6/2018	11/9/2022	4.430137	1.823359	2.097302	1.659036	1.790208
56973	BWB.BWB-PATMH-21	39.19738	-76.5746	SALINITY	1.5	9	5/23/2018	11/9/2022	4.468493	3.202314	4.238323	2.434349	-0.68669
56973	BWB.BWB-PATMH-21	39.19738	-76.5746	SALINITY	1.99	3	4/17/2019	11/6/2019	0.556164	11.1532	11.50157	10.9073	-7.085
56973	BWB.BWB-PATMH-21	39.19738	-76.5746	SALINITY	2	13	5/23/2018	11/9/2022	4.468493	3.505036	4.12076	2.660686	-4.35374
56973	BWB.BWB-PATMH-21	39.19738	-76.5746	SALINITY	2.03	3	5/8/2019	10/9/2019	0.421918	10.98911	19.55713	10.13231	-9.122
56973	BWB.BWB-PATMH-21	39.19738	-76.5746	SALINITY	2.5	10	5/23/2018	11/9/2022	4.468493	2.873837	3.629281	2.295934	0.861667
56973	BWB.BWB-PATMH-21	39.19738	-76.5746	SALINITY	3	14	5/23/2018	11/9/2022	4.468493	2.897484	3.455027	2.21812	-0.93886
56973	BWB.BWB-PATMH-21	39.19738	-76.5746	SALINITY	3.01	5	10/25/2018	11/6/2019	1.032877	10.56567	11.7669	0.981607	-5.93034
56973	BWB.BWB-PATMH-21	39.19738	-76.5746	SALINITY	3.05	4	6/6/2018	9/18/2019	1.284932	16.33641	16.4425	6.881728	-13.901
56973	BWB.BWB-PATMH-21	39.19738	-76.5746	SALINITY	3.5	8	4/27/2021	11/9/2022	1.536986	2.992564	4.109535	2.426171	0.087711
56973	BWB.BWB-PATMH-21	39.19738	-76.5746	SALINITY	4	15	5/23/2018	11/9/2022	4.468493	2.735682	3.220348	2.261621	-0.40501
56973	BWB.BWB-PATMH-21	39.19738	-76.5746	SALINITY	4.01	4	9/21/2018	8/14/2019	0.89589	4.453062	4.531804	3.40286	2.55272
56973	BWB.BWB-PATMH-21	39.19738	-76.5746	SALINITY	4.03	4	4/17/2019	10/9/2019	0.479452	15.52136	16.38532	13.86372	-12.7099
56973	BWB.BWB-PATMH-21	39.19738	-76.5746	SALINITY	4.5	9	5/23/2018	11/9/2022	4.468493	2.179637	2.756869	1.914214	3.021951
56973	BWB.BWB-PATMH-21	39.19738	-76.5746	SALINITY	4.51	3	4/17/2019	10/9/2019	0.479452	11.52976	15.1861	10.02595	-8.05806

57109	ARF.LittleAberdeen	38.9474	-76.5273	SALINITY	2.25	19	4/23/2019	10/9/2019	0.463014	19.63179	20.29231	15.74138	1.66377
57109	ARF.LittleAberdeen	38.9474	-76.5273	SALINITY	2.5	19	4/23/2019	10/9/2019	0.463014	19.51012	20.39253	15.85386	1.64504
57109	ARF.LittleAberdeen	38.9474	-76.5273	SALINITY	2.75	9	5/8/2019	10/9/2019	0.421918	19.39063	21.41642	15.39976	1.86875
57110	ARF.MS1	38.89568	-76.4736	SALINITY	0.3	23	5/4/2022	10/12/2022	0.441096	18.97421	19.45906	17.75864	-51.2288
57110	ARF.MS1	38.89568	-76.4736	SALINITY	0.5	42	4/23/2019	9/8/2022	3.380822	1.508706	1.753753	0.998021	5.41252
57110	ARF.MS1	38.89568	-76.4736	SALINITY	1	62	4/23/2019	10/12/2022	3.473973	1.619121	1.935706	1.37304	4.18216
57110	ARF.MS1	38.89568	-76.4736	SALINITY	1.5	42	4/23/2019	9/8/2022	3.380822	1.509685	1.691663	0.951637	5.639054
57110	ARF.MS1	38.89568	-76.4736	SALINITY	2	63	4/23/2019	10/12/2022	3.473973	1.607673	1.927312	1.41756	4.590381
57110	ARF.MS1	38.89568	-76.4736	SALINITY	2.5	42	4/23/2019	6/15/2022	3.147945	1.198874	1.454796	0.751052	6.601323
57110	ARF.MS1	38.89568	-76.4736	SALINITY	3	63	4/23/2019	10/12/2022	3.473973	1.556639	1.932485	1.39285	5.22546
57110	ARF.MS1	38.89568	-76.4736	SALINITY	3.5	42	4/23/2019	9/8/2022	3.380822	1.499974	1.754625	1.039145	6.42098
57110	ARF.MS1	38.89568	-76.4736	SALINITY	4	62	4/23/2019	10/12/2022	3.473973	1.516612	1.921783	1.34773	5.401677
57110	ARF.MS1	38.89568	-76.4736	SALINITY	4.5	40	4/23/2019	5/4/2022	3.032877	1.297337	1.681853	0.941247	6.896901
57110	ARF.MS1	38.89568	-76.4736	SALINITY	5	57	4/23/2019	10/12/2022	3.473973	1.703957	1.942784	1.399826	5.780446
57110	ARF.MS1	38.89568	-76.4736	SALINITY	5.1	3	8/9/2022	10/12/2022	0.175342	3.749071	6.066984	3.100056	0.6894
57110	ARF.MS1	38.89568	-76.4736	SALINITY	5.2	6	5/10/2022	9/28/2022	0.386301	20.53125	21.64507	18.39252	-55.74
57110	ARF.MS1	38.89568	-76.4736	SALINITY	5.4	5	6/21/2022	10/7/2022	0.29589	14.96737	18.63412	12.00283	-38.0548
57110	ARF.MS1	38.89568	-76.4736	SALINITY	5.5	30	4/23/2019	8/16/2022	3.317808	1.763427	2.04961	1.157722	5.440023
57111	ARF.MS1A	38.91958	-76.5009	SALINITY	0.3	12	8/23/2019	10/12/2022	3.139726	3.101692	3.628749	2.434906	2.13107
57111	ARF.MS1A	38.91958	-76.5009	SALINITY	0.5	45	4/23/2019	10/20/2021	2.49589	1.43716	1.596755	1.122861	5.374786
57111	ARF.MS1A	38.91958	-76.5009	SALINITY	0.75	5	8/23/2019	7/13/2021	1.890411	2.793367	9.188157	-0.0745	3.082857
57111	ARF.MS1A	38.91958	-76.5009	SALINITY	1	52	4/23/2019	10/12/2022	3.473973	1.890937	2.019511	1.623337	4.476513
57111	ARF.MS1A	38.91958	-76.5009	SALINITY	1.25	5	8/23/2019	7/13/2021	1.890411	4.078316	9.327869	-0.0664	0.303571
57111	ARF.MS1A	38.91958	-76.5009	SALINITY	1.5	45	4/23/2019	10/20/2021	2.49589	1.529148	1.603155	1.109241	5.345158
57111	ARF.MS1A	38.91958	-76.5009	SALINITY	1.75	5	8/23/2019	7/13/2021	1.890411	5.493622	9.577764	-0.05269	-2.76071
57111	ARF.MS1A	38.91958	-76.5009	SALINITY	2	52	4/23/2019	10/12/2022	3.473973	1.841689	2.002552	1.559932	4.832971
57111	ARF.MS1A	38.91958	-76.5009	SALINITY	2.25	5	8/23/2019	7/13/2021	1.890411	6.015051	10.02615	-0.00928	-3.76071
57111	ARF.MS1A	38.91958	-76.5009	SALINITY	2.5	45	4/23/2019	10/20/2021	2.49589	1.359053	1.554652	1.025416	5.898911
57111	ARF.MS1A	38.91958	-76.5009	SALINITY	2.75	5	8/23/2019	7/13/2021	1.890411	5.903316	10.4822	0.026024	-3.39643
57111	ARF.MS1A	38.91958	-76.5009	SALINITY	3	52	4/23/2019	10/12/2022	3.473973	1.929118	2.074969	1.554936	4.78833
57111	ARF.MS1A	38.91958	-76.5009	SALINITY	3.25	5	8/23/2019	7/13/2021	1.890411	7.206888	10.73209	-0.01325	-6.23643
57111	ARF.MS1A	38.91958	-76.5009	SALINITY	3.5	45	4/23/2019	10/20/2021	2.49589	1.095976	1.521781	0.950003	6.39134
57111	ARF.MS1A	38.91958	-76.5009	SALINITY	3.75	4	8/23/2019	7/13/2021	1.890411	7.531746	9.07687	3.518428	-6.91556
57111	ARF.MS1A	38.91958	-76.5009	SALINITY	4	48	4/23/2019	10/12/2022	3.473973	1.739072	2.074834	1.526421	4.961086
57111	ARF.MS1A	38.91958	-76.5009	SALINITY	4.25	3	8/23/2019	7/13/2021	1.890411	4.70824	5.502757	-0.65698	-2.06619
57111	ARF.MS1A	38.91958	-76.5009	SALINITY	4.5	41	4/23/2019	10/20/2021	2.49589	1.097009	1.46551	0.81084	7.212831
57111	ARF.MS1A	38.91958	-76.5009	SALINITY	4.75	3	8/23/2019	7/13/2021	1.890411	4.693808	5.484896	-0.64826	-1.9876
57111	ARF.MS1A	38.91958	-76.5009	SALINITY	5	43	4/23/2019	10/12/2022	3.473973	1.741213	2.11032	1.571566	5.598745
57111	ARF.MS1A	38.91958	-76.5009	SALINITY	5.5	37	4/23/2019	9/14/2022	3.39726	1.718143	2.159419	1.380154	7.234312
57111	ARF.MS1A	38.91958	-76.5009	SALINITY	6	35	4/23/2019	10/12/2022	3.473973	1.612897	1.985795	1.329114	6.662851
57111	ARF.MS1A	38.91958	-76.5009	SALINITY	6.5	23	4/23/2019	8/23/2022	3.336986	11.48062	15.03462	7.8841	3.902436
57111	ARF.MS1A	38.91958	-76.5009	SALINITY	7	14	4/23/2019	7/28/2021	2.265753	14.53953	17.00163	9.295467	3.802333

57359	FB.FOBBT06	39.45933	-75.8801	SALINITY	1	15	4/28/2018	9/25/2019	1.410959	-0.0296	0.565298	-0.0748	0.311212
57359	FB.FOBBT06	39.45933	-75.8801	SALINITY	2	17	4/28/2018	9/25/2019	1.410959	-0.01956	0.187623	-0.07796	0.335679
57359	FB.FOBBT06	39.45933	-75.8801	SALINITY	2.4	3	5/24/2018	9/25/2019	1.339726	5.018876	5.973339	0.844413	-5.65015
57359	FB.FOBBT06	39.45933	-75.8801	SALINITY	3	14	4/28/2018	9/25/2019	1.410959	-0.02827	0.803782	-0.09523	0.32742
57359	FB.FOBBT06	39.45933	-75.8801	SALINITY	3.1	4	8/9/2018	9/25/2019	1.128767	1.787755	1.96978	0.79721	-0.15449
57359	FB.FOBBT06	39.45933	-75.8801	SALINITY	3.3	3	6/28/2018	5/23/2019	0.90137	-0.27992	0.018069	-0.34236	0.330494
57359	FB.FOBBT06	39.45933	-75.8801	SALINITY	4	3	6/13/2018	5/23/2019	0.942466	0.347564	0.380916	-0.00152	0.257631
57467	MDE.0102003	39.30306	-76.3231	SALINITY	0.5	16	4/10/2019	8/11/2021	2.339726	0.487745	1.140997	0.021715	1.284028
57467	MDE.0102003	39.30306	-76.3231	SALINITY	1	16	4/10/2019	8/11/2021	2.339726	0.546244	1.189397	-0.09549	1.183833
57467	MDE.0102003	39.30306	-76.3231	SALINITY	2	16	4/10/2019	8/11/2021	2.339726	0.520135	1.30302	-0.09926	1.318731
57467	MDE.0102003	39.30306	-76.3231	SALINITY	3	9	7/16/2019	8/11/2021	2.073973	0.095274	0.658886	-1.52996	1.213889
57468	MDE.0102016	39.24167	-76.3978	SALINITY	0.5	16	4/10/2019	8/11/2021	2.339726	0.118337	0.968201	-0.39388	2.301118
57468	MDE.0102016	39.24167	-76.3978	SALINITY	1	16	4/10/2019	8/11/2021	2.339726	0.118337	1.125366	-0.51448	2.629356
57468	MDE.0102016	39.24167	-76.3978	SALINITY	2	15	4/10/2019	8/11/2021	2.339726	0.122074	0.943309	-0.53967	1.854515
57468	MDE.0102016	39.24167	-76.3978	SALINITY	2.7	3	6/12/2019	8/11/2021	2.167123	3.443306	3.936977	1.167517	-5.12624
57468	MDE.0102016	39.24167	-76.3978	SALINITY	3	5	9/30/2019	7/20/2021	1.805479	-1.4102	2.271939	-3.51755	5.235854
57469	MDE.0102017	39.25861	-76.4433	SALINITY	0.5	16	4/10/2019	8/11/2021	2.339726	0.101108	0.605729	-0.714	1.539751
57469	MDE.0102017	39.25861	-76.4433	SALINITY	1	16	4/10/2019	8/11/2021	2.339726	-0.24415	0.521398	-0.78263	2.31548
57469	MDE.0102017	39.25861	-76.4433	SALINITY	2	3	9/30/2019	6/14/2021	1.706849	-2.04529	-1.94856	-2.67457	5.995313
57470	MDE.0102018	39.29	-76.4672	SALINITY	0.5	16	4/10/2019	8/11/2021	2.339726	-0.16714	0.151136	-0.60783	1.105255
57470	MDE.0102018	39.29	-76.4672	SALINITY	1	16	4/10/2019	8/11/2021	2.339726	-0.30332	0.099589	-0.7144	1.198975
57471	MDE.0103008	39.47693	-75.9432	SALINITY	0.5	12	4/10/2019	11/3/2022	3.569863	0.021172	0.968807	-0.43635	0.181787
57471	MDE.0103008	39.47693	-75.9432	SALINITY	1	12	4/10/2019	11/3/2022	3.569863	0.021172	0.968807	-0.43635	0.181787
57471	MDE.0103008	39.47693	-75.9432	SALINITY	2	12	4/10/2019	11/3/2022	3.569863	0.021172	0.968841	-0.45991	0.181787
57471	MDE.0103008	39.47693	-75.9432	SALINITY	3	5	4/10/2019	6/29/2022	3.221918	3.147292	7.021334	-0.30302	-0.02805
57472	MDE.0103013	39.38261	-76.0681	SALINITY	0.5	12	4/10/2019	11/3/2022	3.569863	0.057937	1.01753	-0.82421	0.1
57472	MDE.0103013	39.38261	-76.0681	SALINITY	1	12	4/10/2019	11/3/2022	3.569863	0	1.210883	-1.00014	0.1
57472	MDE.0103013	39.38261	-76.0681	SALINITY	2	12	4/10/2019	11/3/2022	3.569863	0	1.247773	-1.16017	0.1
57472	MDE.0103013	39.38261	-76.0681	SALINITY	3	10	4/10/2019	4/13/2022	3.010959	0	0.051393	-1.27977	0.1
57472	MDE.0103013	39.38261	-76.0681	SALINITY	4	5	4/10/2019	7/14/2021	2.263014	1.814663	13.16039	-0.31831	0.01561
57472	MDE.0103013	39.38261	-76.0681	SALINITY	0.5	12	4/10/2019	11/3/2022	3.569863	0.057937	1.01753	-0.82421	0.1
57472	MDE.0103013	39.38261	-76.0681	SALINITY	1	12	4/10/2019	11/3/2022	3.569863	0	1.210883	-1.00014	0.1
57472	MDE.0103013	39.38261	-76.0681	SALINITY	2	12	4/10/2019	11/3/2022	3.569863	0	1.247773	-1.16017	0.1
57472	MDE.0103013	39.38261	-76.0681	SALINITY	3	10	4/10/2019	4/13/2022	3.010959	0	0.051393	-1.27977	0.1
57472	MDE.0103013	39.38261	-76.0681	SALINITY	4	5	4/10/2019	7/14/2021	2.263014	1.814663	13.16039	-0.31831	0.01561
57473	MDE.0301003	39.0692	-76.4806	SALINITY	0.5	21	4/9/2019	10/12/2021	2.512329	0.352906	1.323394	-0.31004	5.623897
57473	MDE.0301003	39.0692	-76.4806	SALINITY	1	20	4/9/2019	10/12/2021	2.512329	0.661189	1.855229	-0.18455	5.972839
57473	MDE.0301003	39.0692	-76.4806	SALINITY	2	9	4/9/2019	7/26/2021	2.29863	3.773424	7.633135	0.552433	5.33
57473	MDE.0301003	39.0692	-76.4806	SALINITY	3	6	4/9/2019	7/26/2021	2.29863	4.034072	12.39198	0.15488	3.8
57473	MDE.0301003	39.0692	-76.4806	SALINITY	3.5	4	4/9/2019	7/26/2021	2.29863	1.804926	7.048244	1.479142	3.9
57474	MDE.0301005C	39.0875	-76.4608	SALINITY	0.5	21	4/9/2019	10/12/2021	2.512329	0.189006	1.278807	-0.43602	5.825538
57474	MDE.0301005C	39.0875	-76.4608	SALINITY	1	20	4/9/2019	10/12/2021	2.512329	-0.34274	0.821086	-1.05229	6.612877

57506	MDE.1803010	38.12028	-75.9089	SALINITY	2	13	2/18/2020	12/20/2022	2.838356	0.227486	1.697001	-0.60374	15.03779
57506	MDE.1803010	38.12028	-75.9089	SALINITY	3	4	2/18/2020	10/14/2021	1.654795	-1.55687	-0.59262	-2.36012	17.72844
57507	MDE.1803017	38.09806	-75.8817	SALINITY	0.5	24	5/6/2019	12/20/2022	3.627397	1.187253	1.388644	0.196721	12.55877
57507	MDE.1803017	38.09806	-75.8817	SALINITY	1	24	5/6/2019	12/20/2022	3.627397	1.010769	1.344903	0.159944	12.61297
57507	MDE.1803017	38.09806	-75.8817	SALINITY	2	22	5/6/2019	10/24/2022	3.471233	0.801465	0.968062	-0.1272	13.05639
57507	MDE.1803017	38.09806	-75.8817	SALINITY	3	7	6/18/2019	5/13/2021	1.90411	-2.52024	1.293432	-4.02514	20.14548
57508	MDE.1804001	38.07583	-75.7839	SALINITY	0.5	18	5/14/2019	9/22/2022	3.361644	1.907797	2.403879	1.45273	10.36105
57508	MDE.1804001	38.07583	-75.7839	SALINITY	1	18	5/14/2019	9/22/2022	3.361644	1.90379	2.230297	1.408506	10.43075
57508	MDE.1804001	38.07583	-75.7839	SALINITY	2	10	5/14/2019	8/22/2022	3.276712	2.417801	2.908027	-1.02676	8.785206
57509	MDE.1804007	38.0525	-75.87	SALINITY	0.5	18	5/14/2019	9/22/2022	3.361644	1.270934	1.653154	0.183005	11.13847
57509	MDE.1804007	38.0525	-75.87	SALINITY	1	18	5/14/2019	9/22/2022	3.361644	1.295042	1.694192	0.182992	11.19335
57509	MDE.1804007	38.0525	-75.87	SALINITY	2	15	5/14/2019	9/22/2022	3.361644	1.458458	1.715464	0.0917	10.99445
57509	MDE.1804007	38.0525	-75.87	SALINITY	3	9	2/25/2020	5/26/2022	2.249315	-0.15354	0.30838	-1.57504	15.32572
57510	MDE.1806011	37.92972	-75.775	SALINITY	0.5	17	6/17/2019	11/15/2022	3.416438	1.4904	1.910893	-0.37267	13.85494
57510	MDE.1806011	37.92972	-75.775	SALINITY	1	17	6/17/2019	11/15/2022	3.416438	1.525117	2.168021	-0.12098	13.84335
57510	MDE.1806011	37.92972	-75.775	SALINITY	2	15	8/14/2019	11/15/2022	3.257534	1.235323	1.889403	-0.54252	15.23063
57510	MDE.1806011	37.92972	-75.775	SALINITY	3	3	6/8/2020	10/19/2021	1.364384	-1.01644	2.716862	-3.8066	15.08732
57511	MDE.1807012	37.97958	-75.6371	SALINITY	0.5	17	6/17/2019	11/15/2022	3.416438	2.064169	2.826532	1.036287	2.664754
57511	MDE.1807012	37.97958	-75.6371	SALINITY	1	17	6/17/2019	11/15/2022	3.416438	1.950271	3.066804	0.86719	3.031557
57511	MDE.1807012	37.97958	-75.6371	SALINITY	2	17	6/17/2019	11/15/2022	3.416438	2.028653	2.823678	1.009912	3.384981
57511	MDE.1807012	37.97958	-75.6371	SALINITY	3	17	6/17/2019	11/15/2022	3.416438	2.028653	2.972183	0.985385	2.747951
57511	MDE.1807012	37.97958	-75.6371	SALINITY	4	17	6/17/2019	11/15/2022	3.416438	1.978789	3.022608	1.047954	2.964754
57511	MDE.1807012	37.97958	-75.6371	SALINITY	5	17	6/17/2019	11/15/2022	3.416438	1.893234	2.897514	0.88851	3.465164
57511	MDE.1807012	37.97958	-75.6371	SALINITY	6	16	6/17/2019	11/15/2022	3.416438	1.633206	2.349289	0.439203	5.440477
57511	MDE.1807012	37.97958	-75.6371	SALINITY	7	14	6/17/2019	10/20/2022	3.345205	1.788984	2.21205	-0.19746	5.382842
57511	MDE.1807012	37.97958	-75.6371	SALINITY	8	9	6/17/2019	10/20/2022	3.345205	1.970683	2.365794	-0.42355	6.49898
57512	MDE.1807111	37.95692	-75.665	SALINITY	0.5	17	6/17/2019	11/15/2022	3.416438	2.326628	2.823195	0.799734	7.25122
57512	MDE.1807111	37.95692	-75.665	SALINITY	1	8	6/17/2019	10/20/2022	3.345205	1.216667	3.041667	-0.78187	10.10904
57521	NWA.MANA7	38.25951	-75.9122	SALINITY	0.5	77	3/25/2019	11/7/2022	3.624658	0.736961	1.303612	0.516187	10.62697
57521	NWA.MANA7	38.25951	-75.9122	SALINITY	0.6	5	5/5/2019	10/10/2021	2.435616	-1.11373	4.448286	-1.1391	16.23773
57771	SCC.BCC1	38.9563	-76.4867	SALINITY	0.2	4	6/6/2019	8/1/2019	0.153425	21.72619	23.46429	19.55357	3.4
57771	SCC.BCC1	38.9563	-76.4867	SALINITY	0.6	3	6/6/2019	8/1/2019	0.153425	26.50595	28.35268	20.96577	2.65
57772	SCC.BCC2	38.95816	-76.4868	SALINITY	0.2	4	6/6/2019	8/1/2019	0.153425	22.48661	24.11607	20.40089	3.5
57773	SCC.BCC3	38.96124	-76.4809	SALINITY	0.2	4	6/6/2019	8/1/2019	0.153425	23.00804	24.11607	21.61746	3.7
57774	SCC.BCC4	38.96236	-76.4791	SALINITY	0.2	4	6/6/2019	8/1/2019	0.153425	23.13839	25.41964	20.85712	3.6
57774	SCC.BCC4	38.96236	-76.4791	SALINITY	0.4	3	6/27/2019	8/1/2019	0.09589	23.89881	29.98214	14.77381	3.15
57774	SCC.BCC4	38.96236	-76.4791	SALINITY	0.8	3	6/27/2019	8/1/2019	0.09589	21.72619	27.63571	12.8619	3.4
57775	SCC.BCC5	38.96316	-76.4841	SALINITY	0.2	4	6/6/2019	8/1/2019	0.153425	22.8125	24.76786	20.85711	3.6
57776	SCC.BCC6	38.96598	-76.4831	SALINITY	0.2	4	6/6/2019	8/1/2019	0.153425	24.33333	25.72386	22.5952	3.6
57776	SCC.BCC6	38.96598	-76.4831	SALINITY	1.2	3	6/27/2019	8/1/2019	0.09589	27.80952	33.37143	19.46667	2.8
57777	SRA.BC1	39.0004	-76.4512	SALINITY	0.5	51	6/27/2019	10/27/2022	3.336986	0.789018	1.551159	0.544054	6.434503
57777	SRA.BC1	39.0004	-76.4512	SALINITY	1	51	6/27/2019	10/27/2022	3.336986	0.779418	1.535379	0.529766	6.690483

57801	SRA.RBE	39.0582	-76.5481	SALINITY	1	94	5/29/2019	10/28/2022	3.419178	0.839806	1.008987	0.483881	6.307189
57801	SRA.RBE	39.0582	-76.5481	SALINITY	1.5	92	6/5/2019	10/28/2022	3.4	0.676333	0.90557	0.367557	6.519602
57801	SRA.RBE	39.0582	-76.5481	SALINITY	2	91	5/29/2019	10/28/2022	3.419178	0.859166	1.022082	0.493429	6.205887
57801	SRA.RBE	39.0582	-76.5481	SALINITY	2.5	62	6/5/2019	10/19/2022	3.375342	0.969591	1.086325	0.37555	6.163408
57801	SRA.RBE	39.0582	-76.5481	SALINITY	3	4	5/29/2019	10/29/2021	2.421918	1.927423	2.231029	1.895738	4.063036
57802	SRA.RBN	39.0599	-76.5618	SALINITY	0.5	104	7/18/2018	10/28/2022	4.282192	1.094005	1.18079	0.898743	4.668158
57802	SRA.RBN	39.0599	-76.5618	SALINITY	1	105	7/18/2018	10/28/2022	4.282192	1.119117	1.197494	0.940807	4.732471
57802	SRA.RBN	39.0599	-76.5618	SALINITY	2	105	7/18/2018	10/28/2022	4.282192	1.12502	1.248495	0.990412	4.760811
57802	SRA.RBN	39.0599	-76.5618	SALINITY	3	105	7/18/2018	10/28/2022	4.282192	1.043234	1.175211	0.853663	4.939962
57802	SRA.RBN	39.0599	-76.5618	SALINITY	4	105	7/18/2018	10/28/2022	4.282192	1.018691	1.189462	0.844687	5.166218
57802	SRA.RBN	39.0599	-76.5618	SALINITY	5	105	7/18/2018	10/28/2022	4.282192	0.992346	1.187001	0.812901	5.427256
57802	SRA.RBN	39.0599	-76.5618	SALINITY	6	104	7/18/2018	10/28/2022	4.282192	0.841154	1.16484	0.766469	6.041322
57802	SRA.RBN	39.0599	-76.5618	SALINITY	7	71	7/18/2018	10/28/2022	4.282192	0.293081	0.69333	0.099727	7.624355
57802	SRA.RBN	39.0599	-76.5618	SALINITY	8	8	6/3/2020	10/6/2022	2.342466	0.693206	1.646918	-0.29508	8.055041
57803	SRA.RBP	39.0412	-76.5723	SALINITY	0.5	95	8/29/2018	9/1/2022	4.010959	1.037605	1.024382	0.558686	5.380868
57803	SRA.RBP	39.0412	-76.5723	SALINITY	1	95	8/29/2018	9/1/2022	4.010959	1.035098	1.016477	0.534777	5.407143
57803	SRA.RBP	39.0412	-76.5723	SALINITY	1.5	26	7/15/2021	8/17/2022	1.090411	-0.06505	0.645971	-0.48307	8.435043
57803	SRA.RBP	39.0412	-76.5723	SALINITY	2	94	8/29/2018	9/1/2022	4.010959	1.021639	1.070412	0.607971	5.38423
57803	SRA.RBP	39.0412	-76.5723	SALINITY	2.5	25	7/15/2021	8/17/2022	1.090411	0.021354	0.800839	-0.29102	8.187334
57803	SRA.RBP	39.0412	-76.5723	SALINITY	3	94	8/29/2018	9/1/2022	4.010959	1.002302	1.05254	0.580153	5.430024
57803	SRA.RBP	39.0412	-76.5723	SALINITY	3.5	24	7/15/2021	8/17/2022	1.090411	-0.39189	0.513362	-0.62925	9.803626
57803	SRA.RBP	39.0412	-76.5723	SALINITY	4	90	8/29/2018	9/1/2022	4.010959	1.025884	1.029584	0.518093	5.466657
57803	SRA.RBP	39.0412	-76.5723	SALINITY	4.5	3	6/9/2022	9/1/2022	0.230137	18.70444	24.81586	12.87727	-64.4792
57804	SRA.RBS	39.0354	-76.5429	SALINITY	0.5	112	7/25/2018	10/28/2022	4.263014	1.238412	1.303746	1.013841	4.537698
57804	SRA.RBS	39.0354	-76.5429	SALINITY	1	115	7/18/2018	10/28/2022	4.282192	1.26403	1.339385	1.074272	4.52657
57804	SRA.RBS	39.0354	-76.5429	SALINITY	1.5	4	9/15/2022	10/28/2022	0.117808	7.75625	11.92165	6.880505	-19.8438
57804	SRA.RBS	39.0354	-76.5429	SALINITY	2	115	7/18/2018	10/28/2022	4.282192	1.244498	1.341321	1.067372	4.520203
57804	SRA.RBS	39.0354	-76.5429	SALINITY	2.5	4	9/15/2022	10/28/2022	0.117808	7.75625	11.98925	6.998233	-19.8438
57804	SRA.RBS	39.0354	-76.5429	SALINITY	3	114	7/18/2018	10/28/2022	4.282192	1.26574	1.363581	1.105041	4.525044
57804	SRA.RBS	39.0354	-76.5429	SALINITY	3.5	4	9/15/2022	10/28/2022	0.117808	7.528125	12.34833	7.060825	-18.8669
57804	SRA.RBS	39.0354	-76.5429	SALINITY	4	113	7/18/2018	10/28/2022	4.282192	1.220827	1.334787	1.027725	4.548096
57804	SRA.RBS	39.0354	-76.5429	SALINITY	5	107	7/18/2018	10/28/2022	4.282192	1.147576	1.244284	0.932614	4.912689
57804	SRA.RBS	39.0354	-76.5429	SALINITY	6	107	7/18/2018	10/28/2022	4.282192	0.945284	1.157995	0.800159	5.83748
57804	SRA.RBS	39.0354	-76.5429	SALINITY	7	106	7/18/2018	10/28/2022	4.282192	0.867256	1.245164	0.821558	6.271556
57804	SRA.RBS	39.0354	-76.5429	SALINITY	8	89	7/18/2018	10/19/2022	4.257534	0.949178	1.319041	0.929838	6.52636
57804	SRA.RBS	39.0354	-76.5429	SALINITY	9	3	7/2/2021	8/19/2021	0.131507	7.510152	13.45177	-5.56141	-14.7308
57805	SRA.RBW	39.0374	-76.5601	SALINITY	0.5	105	7/18/2018	10/28/2022	4.282192	1.163586	1.171739	0.882649	4.730993
57805	SRA.RBW	39.0374	-76.5601	SALINITY	1	103	8/15/2018	10/28/2022	4.205479	1.176062	1.200159	0.851155	4.629853
57805	SRA.RBW	39.0374	-76.5601	SALINITY	1.5	3	7/18/2018	8/1/2018	0.038356	-34.1536	-25.1589	-43.1482	7.075
57805	SRA.RBW	39.0374	-76.5601	SALINITY	2	102	8/15/2018	10/28/2022	4.205479	1.150989	1.174425	0.837275	4.674864
57805	SRA.RBW	39.0374	-76.5601	SALINITY	2.5	3	7/18/2018	8/1/2018	0.038356	-34.4143	-18.25	-50.5786	7.35
57805	SRA.RBW	39.0374	-76.5601	SALINITY	3	103	8/15/2018	10/28/2022	4.205479	1.195713	1.2099	0.870192	4.630252

64643	XCH3277	38.22027	-76.0386	SALINITY	0.8	3	4/20/2023	8/14/2023	0.317808	2.328292	3.062943	1.845521	11.48862
64643	XCH3277	38.22027	-76.0386	SALINITY	0.9	4	7/20/2021	9/21/2022	1.172603	10.22	12.06574	5.202007	1.408
64643	XCH3277	38.22027	-76.0386	SALINITY	1	10	6/23/2021	10/12/2023	2.30411	1.505422	2.919973	-0.68764	11.89835
64643	XCH3277	38.22027	-76.0386	SALINITY	0.1	3	9/28/2021	10/12/2022	1.038356	-3.24286	2.673087	-3.46977	22.55658
64643	XCH3277	38.22027	-76.0386	SALINITY	0.2	4	8/17/2021	8/30/2022	1.035616	19.81429	20.92232	10.28317	-11.5757
64643	XCH3277	38.22027	-76.0386	SALINITY	0.3	5	7/22/2021	10/25/2022	1.260274	4.307736	5.240735	2.706127	9.552268
64643	XCH3277	38.22027	-76.0386	SALINITY	0.4	4	7/6/2022	5/24/2023	0.882192	2.228277	2.365574	1.627965	11.57566
64643	XCH3277	38.22027	-76.0386	SALINITY	0.5	33	4/14/2021	10/12/2023	2.49589	1.576869	1.915307	1.435834	12.31131
64643	XCH3277	38.22027	-76.0386	SALINITY	0.6	3	10/13/2021	5/2/2023	1.550685	0.151844	2.942155	-1.64888	11.75193
64643	XCH3277	38.22027	-76.0386	SALINITY	0.7	7	4/27/2021	5/10/2023	2.035616	1.569847	1.945103	0.79839	12.58839
64643	XCH3277	38.22027	-76.0386	SALINITY	0.8	3	4/20/2023	8/14/2023	0.317808	2.328292	3.062943	1.845521	11.48862
64643	XCH3277	38.22027	-76.0386	SALINITY	0.9	4	7/20/2021	9/21/2022	1.172603	10.22	12.06574	5.202007	1.408
64643	XCH3277	38.22027	-76.0386	SALINITY	1	10	6/23/2021	10/12/2023	2.30411	1.505422	2.919973	-0.68764	11.89835
64644	XCI0132	38.16864	-75.9471	SALINITY	0.5	56	4/27/2021	10/11/2023	2.457534	1.978523	2.221103	1.818539	12.18845
64644	XCI0132	38.16864	-75.9471	SALINITY	1	50	4/27/2021	10/11/2023	2.457534	1.864446	2.106321	1.744745	12.23974
64644	XCI0132	38.16864	-75.9471	SALINITY	1.1	4	8/3/2021	9/20/2023	2.131507	1.987301	2.365441	1.60919	13.1662
64644	XCI0132	38.16864	-75.9471	SALINITY	1.2	3	8/31/2021	8/17/2022	0.961644	2.27675	4.357958	-2.1644	9.45325
64644	XCI0132	38.16864	-75.9471	SALINITY	1.3	6	4/27/2021	10/11/2023	2.457534	2.117836	4.131735	1.913497	12.24535
64644	XCI0132	38.16864	-75.9471	SALINITY	1.4	7	5/20/2021	5/17/2023	1.991781	0.538157	1.890268	-0.13914	14.47395
64644	XCI0132	38.16864	-75.9471	SALINITY	1.5	23	7/6/2021	7/17/2023	2.030137	1.761772	2.062793	1.44015	12.64859
64644	XCI0132	38.16864	-75.9471	SALINITY	1.6	7	5/17/2021	11/9/2022	1.482192	2.376227	3.649777	0.535349	11.51769
64644	XCI0132	38.16864	-75.9471	SALINITY	1.7	3	4/26/2022	7/17/2023	1.224658	4.725881	6.143992	1.831777	9.813767
64644	XCI0132	38.16864	-75.9471	SALINITY	1.8	4	10/28/2021	5/10/2023	1.531507	0.882375	1.327279	0.437478	13.88519
64644	XCI0132	38.16864	-75.9471	SALINITY	2	6	7/20/2021	10/21/2022	1.254795	0.798927	3.545667	0.798927	13.74043
64644	XCI0132	38.16864	-75.9471	SALINITY	0.5	56	4/27/2021	10/11/2023	2.457534	1.978523	2.221103	1.818539	12.18845
64644	XCI0132	38.16864	-75.9471	SALINITY	1	50	4/27/2021	10/11/2023	2.457534	1.864446	2.106321	1.744745	12.23974
64644	XCI0132	38.16864	-75.9471	SALINITY	1.1	4	8/3/2021	9/20/2023	2.131507	1.987301	2.365441	1.60919	13.1662
64644	XCI0132	38.16864	-75.9471	SALINITY	1.2	3	8/31/2021	8/17/2022	0.961644	2.27675	4.357958	-2.1644	9.45325
64644	XCI0132	38.16864	-75.9471	SALINITY	1.3	6	4/27/2021	10/11/2023	2.457534	2.117836	4.131735	1.913497	12.24535
64644	XCI0132	38.16864	-75.9471	SALINITY	1.4	7	5/20/2021	5/17/2023	1.991781	0.538157	1.890268	-0.13914	14.47395
64644	XCI0132	38.16864	-75.9471	SALINITY	1.5	23	7/6/2021	7/17/2023	2.030137	1.761772	2.062793	1.44015	12.64859
64644	XCI0132	38.16864	-75.9471	SALINITY	1.6	7	5/17/2021	11/9/2022	1.482192	2.376227	3.649777	0.535349	11.51769
64644	XCI0132	38.16864	-75.9471	SALINITY	1.7	3	4/26/2022	7/17/2023	1.224658	4.725881	6.143992	1.831777	9.813767
64644	XCI0132	38.16864	-75.9471	SALINITY	1.8	4	10/28/2021	5/10/2023	1.531507	0.882375	1.327279	0.437478	13.88519
64644	XCI0132	38.16864	-75.9471	SALINITY	2	6	7/20/2021	10/21/2022	1.254795	0.798927	3.545667	0.798927	13.74043
64645	ARF.Locust	38.8834	-76.5226	SALINITY	0.3	48	4/8/2021	10/10/2022	1.506849	1.886312	2.453004	1.648604	7.416434
64645	ARF.Locust	38.8834	-76.5226	SALINITY	0.5	44	4/8/2021	10/10/2022	1.506849	1.695921	2.235316	1.41008	7.615113
64645	ARF.Locust	38.8834	-76.5226	SALINITY	0.75	26	4/8/2021	5/11/2022	1.090411	3.448784	3.841438	1.545339	7.826601
64645	ARF.Locust	38.8834	-76.5226	SALINITY	1	47	4/8/2021	10/10/2022	1.506849	2.356068	2.642722	1.874411	7.35616
64645	ARF.Locust	38.8834	-76.5226	SALINITY	1.25	26	4/8/2021	5/11/2022	1.090411	3.392958	3.758542	1.515281	7.834085
64645	ARF.Locust	38.8834	-76.5226	SALINITY	1.5	44	4/8/2021	10/10/2022	1.506849	1.929835	2.339255	1.484818	7.750248
64645	ARF.Locust	38.8834	-76.5226	SALINITY	1.75	25	4/8/2021	5/11/2022	1.090411	3.227815	3.854652	1.707313	7.95303

64661	ARF.Popham	38.86037	-76.5408	SALINITY	1	41	4/8/2021	10/10/2022	1.506849	2.157951	2.507335	1.49415	7.542402
64661	ARF.Popham	38.86037	-76.5408	SALINITY	1.1	6	6/1/2022	9/19/2022	0.30137	17.36748	23.53621	12.72286	-11.7842
64661	ARF.Popham	38.86037	-76.5408	SALINITY	1.2	4	5/5/2022	10/6/2022	0.421918	15.65444	16.85696	14.41426	-10.0224
64661	ARF.Popham	38.86037	-76.5408	SALINITY	1.25	22	4/8/2021	5/11/2022	1.090411	3.386361	4.416091	1.963031	8.419918
64661	ARF.Popham	38.86037	-76.5408	SALINITY	1.4	3	5/11/2022	7/20/2022	0.191781	19.85774	20.50952	19.42321	-14.5181
64661	ARF.Popham	38.86037	-76.5408	SALINITY	1.5	20	4/8/2021	8/17/2022	1.358904	2.103095	3.734946	0.877211	8.845833
64661	ARF.Popham	38.86037	-76.5408	SALINITY	1.75	10	4/8/2021	10/13/2021	0.515068	4.856868	7.448677	3.085278	7.560566
64662	ARF.RedMarker6	38.8444	-76.5344	SALINITY	0.3	45	4/8/2021	10/10/2022	1.506849	3.500453	3.950674	2.923983	7.276992
64662	ARF.RedMarker6	38.8444	-76.5344	SALINITY	0.5	44	4/8/2021	10/10/2022	1.506849	1.939836	2.298128	1.432449	7.91492
64662	ARF.RedMarker6	38.8444	-76.5344	SALINITY	0.75	23	4/8/2021	5/11/2022	1.090411	3.332008	4.270839	1.22747	8.154103
64662	ARF.RedMarker6	38.8444	-76.5344	SALINITY	1	47	4/8/2021	10/10/2022	1.506849	2.443891	2.67311	1.837196	7.561552
64662	ARF.RedMarker6	38.8444	-76.5344	SALINITY	1.25	23	4/8/2021	5/11/2022	1.090411	3.793972	4.582849	1.441689	8.073534
64662	ARF.RedMarker6	38.8444	-76.5344	SALINITY	1.5	44	4/8/2021	10/10/2022	1.506849	1.856769	2.32493	1.452079	7.844131
64662	ARF.RedMarker6	38.8444	-76.5344	SALINITY	1.75	23	4/8/2021	5/11/2022	1.090411	3.729348	4.450866	1.321068	8.275365
64662	ARF.RedMarker6	38.8444	-76.5344	SALINITY	2	47	4/8/2021	10/10/2022	1.506849	2.239322	2.706623	1.863799	7.595621
64662	ARF.RedMarker6	38.8444	-76.5344	SALINITY	2.25	22	4/8/2021	5/11/2022	1.090411	3.170578	4.35755	1.189797	8.36627
64662	ARF.RedMarker6	38.8444	-76.5344	SALINITY	2.5	42	4/8/2021	10/10/2022	1.506849	1.947368	2.338854	1.449392	7.699933
64662	ARF.RedMarker6	38.8444	-76.5344	SALINITY	2.75	19	4/14/2021	5/5/2022	1.057534	3.171174	4.397951	1.517246	8.421821
64662	ARF.RedMarker6	38.8444	-76.5344	SALINITY	3	39	4/14/2021	10/10/2022	1.490411	2.42459	2.799865	1.996889	7.921847
64662	ARF.RedMarker6	38.8444	-76.5344	SALINITY	3.2	3	7/6/2022	8/31/2022	0.153425	10.02881	22.27152	2.683185	-0.56586
64662	ARF.RedMarker6	38.8444	-76.5344	SALINITY	3.25	18	4/14/2021	10/13/2021	0.49863	4.940404	5.595565	2.197995	8.265974
64662	ARF.RedMarker6	38.8444	-76.5344	SALINITY	3.3	4	7/27/2022	10/10/2022	0.205479	1.782558	5.800822	1.667859	10.24349
64662	ARF.RedMarker6	38.8444	-76.5344	SALINITY	3.4	3	5/18/2022	9/19/2022	0.339726	19.36289	20.46958	13.93484	-12.9989
64662	ARF.RedMarker6	38.8444	-76.5344	SALINITY	3.5	19	4/14/2021	8/17/2022	1.342466	1.734954	2.360396	0.734296	8.920666
64662	ARF.RedMarker6	38.8444	-76.5344	SALINITY	3.75	6	4/14/2021	10/6/2021	0.479452	5.911972	6.643265	-2.43341	7.705915
64663	ARF.Scaffold	38.8656	-76.5336	SALINITY	0.3	46	4/8/2021	10/10/2022	1.506849	2.168438	2.749169	1.830784	7.36
64663	ARF.Scaffold	38.8656	-76.5336	SALINITY	0.5	42	4/8/2021	10/10/2022	1.506849	1.848397	2.419031	1.448682	7.567822
64663	ARF.Scaffold	38.8656	-76.5336	SALINITY	0.75	24	4/8/2021	5/11/2022	1.090411	2.899425	3.962737	1.088912	8.350381
64663	ARF.Scaffold	38.8656	-76.5336	SALINITY	0.8	3	5/18/2022	9/26/2022	0.358904	11.16097	19.06075	9.820832	-2.77883
64663	ARF.Scaffold	38.8656	-76.5336	SALINITY	0.9	5	7/6/2022	10/10/2022	0.263014	14.09823	16.50104	6.51525	-6.40777
64663	ARF.Scaffold	38.8656	-76.5336	SALINITY	1	37	4/8/2021	10/6/2022	1.49589	1.799597	2.278963	1.174292	8.00078
64663	ARF.Scaffold	38.8656	-76.5336	SALINITY	1.1	3	6/1/2022	10/6/2022	0.347945	7.792779	18.52635	1.049896	-2.52184
64663	ARF.Scaffold	38.8656	-76.5336	SALINITY	1.2	4	5/5/2022	8/3/2022	0.246575	10.34167	14.86617	10.02657	-3.01667
64663	ARF.Scaffold	38.8656	-76.5336	SALINITY	1.25	19	4/8/2021	5/11/2022	1.090411	4.061129	5.015517	1.695163	7.937178
64663	ARF.Scaffold	38.8656	-76.5336	SALINITY	1.5	11	4/8/2021	8/17/2022	1.358904	2.769268	3.368588	0.672364	8.042233
64664	ARF.Smith	38.8333	-76.549	SALINITY	0.3	46	4/8/2021	10/10/2022	1.506849	2.28543	2.809997	1.818382	6.768219
64664	ARF.Smith	38.8333	-76.549	SALINITY	0.5	43	4/8/2021	10/10/2022	1.506849	1.91075	2.329765	1.484976	7.136374
64664	ARF.Smith	38.8333	-76.549	SALINITY	0.7	3	7/6/2022	8/24/2022	0.134247	-4.51905	14.40136	-7.67245	14.89762
64664	ARF.Smith	38.8333	-76.549	SALINITY	0.75	24	4/8/2021	5/11/2022	1.090411	1.392464	2.730155	-0.14484	8.063813
64664	ARF.Smith	38.8333	-76.549	SALINITY	0.8	5	6/13/2022	10/10/2022	0.326027	14.95804	16.69383	9.979734	-9.68848
64664	ARF.Smith	38.8333	-76.549	SALINITY	0.9	6	5/5/2022	9/16/2022	0.367123	18.58138	21.95508	16.07247	-13.4514
64664	ARF.Smith	38.8333	-76.549	SALINITY	1	27	4/8/2021	10/6/2022	1.49589	1.477688	2.570635	0.633056	8.019003

65080	BWB.BWB-PATMH-SC	39.28242	-76.6111	SALINITY	3.5	6	4/13/2022	11/2/2022	0.556164	15.77656	18.68556	13.12055	5.850256
65080	BWB.BWB-PATMH-SC	39.28242	-76.6111	SALINITY	4	5	4/13/2022	10/20/2022	0.520548	12.98435	28.05678	9.370277	7.111026
65080	BWB.BWB-PATMH-SC	39.28242	-76.6111	SALINITY	4.5	5	4/13/2022	10/20/2022	0.520548	12.80135	27.87532	9.43254	7.206282
65080	BWB.BWB-PATMH-SC	39.28242	-76.6111	SALINITY	5	3	8/3/2022	10/6/2022	0.175342	32.69067	49.31936	19.75725	-1.47889
65093	MDE.1404100	38.36439	-75.9351	SALINITY	0.5	8	1/21/2022	12/13/2022	0.893151	5.348388	8.858342	3.775874	7.268247
65093	MDE.1404100	38.36439	-75.9351	SALINITY	1	7	1/21/2022	12/13/2022	0.893151	5.22104	8.108637	3.353243	6.890047
65093	MDE.1404100	38.36439	-75.9351	SALINITY	1.8	3	7/21/2022	11/7/2022	0.29863	6.054706	8.854155	-0.12055	6.153412
65094	MDE.1404200	38.31851	-76.0882	SALINITY	0.5	8	1/21/2022	12/13/2022	0.893151	7.12672	9.842299	1.042874	6.393013
65094	MDE.1404200	38.31851	-76.0882	SALINITY	1	6	1/21/2022	12/13/2022	0.893151	6.153967	9.415389	1.674324	4.4
65095	NWA.DECR5	38.64487	-75.5799	SALINITY	0.5	19	3/27/2022	11/6/2022	0.613699	0	NA	NA	0.1
65095	NWA.DECR5	38.64487	-75.5799	SALINITY	1	3	3/27/2022	7/3/2022	0.268493	0	NA	NA	0.1
65095	NWA.DECR5	38.64487	-75.5799	SALINITY	1.1	4	5/9/2022	7/17/2022	0.189041	0.675926	0.675926	0.675926	-0.02963
65095	NWA.DECR5	38.64487	-75.5799	SALINITY	1.2	3	4/24/2022	9/11/2022	0.383562	0	NA	NA	0.1
65096	NWA.MAHO6	38.69391	-75.7723	SALINITY	0.5	11	7/4/2022	11/7/2022	0.345205	0	NA	NA	0.1
65112	XCH7886	38.2968	-76.0234	SALINITY	1	3	4/13/2023	5/22/2023	0.106849	-8.20274	4.005216	-17.6362	15.53441
